# The Importance of Atomic Charges for Predicting Site-Selective Ir-, Ru-, and Rh-Catalyzed C–H Borylations

**DOI:** 10.1021/acs.joc.5c00343

**Published:** 2025-04-23

**Authors:** Shannon M. Stephens, Kyle M. Lambert

**Affiliations:** Department of Chemistry and Biochemistry, Old Dominion University, 4501 Elkhorn Ave, Norfolk, Virginia 23529, United States

## Abstract

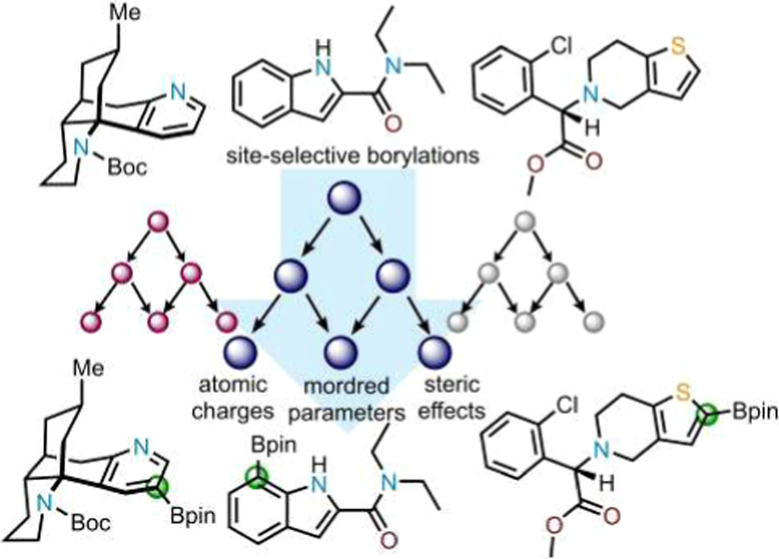

A supervised machine learning model has been developed that allows for the prediction of site selectivity in late-stage C–H borylations. Model development was accomplished using literature data for the site-selective (≥95%) C–H borylation of 189 unique arene, heteroarene, and aliphatic substrates that feature a total of 971 possible sp^2^ or sp^3^ C–H borylation sites. The reported experimental data was supplemented with additional chemoinformatic descriptors, computed atomic charges at the C–H borylation sites, and data from parameterization of catalytically active tris-boryl complexes resulting from the combination of seven different Ir-, Ru-, and Rh-based precatalysts with eight different ligands. Of the over 1600 parameters investigated, the computed atomic charges (e.g., Hirshfeld, ChelpG, and Mulliken charges) on the hydrogen and carbon atoms at the site of borylation were identified as the most important features that allow for the successful prediction of whether a particular C–H bond will undergo a site-selective borylation. The overall accuracy of the developed model was 88.9% ± 2.5% with precision, recall, and F1 scores of 92–95% for the nonborylating sites and 65–75% for the sites of borylation. The model was demonstrated to be generalizable to molecules outside of the training/test sets with an additional validation set of 12 electronically and structurally diverse systems.

## Introduction

**Figure 1:**
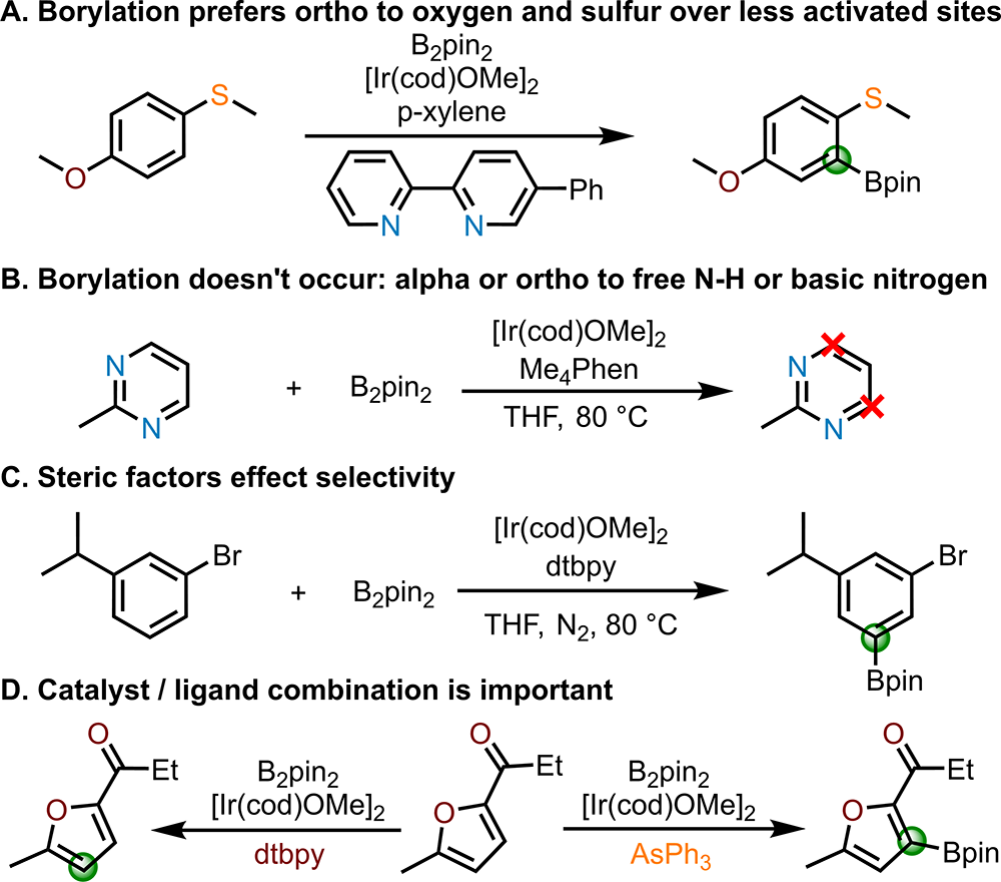
Empirical guidelines to predict sites of C–H borylation.

Synthetic methods that are able to accomplish selective, late-stage functionalization of advanced intermediates have found an increasing role in both the synthesis of complex natural products and expanding the molecular diversity of potential lead compounds in medicinal chemistry efforts.^1−3^ The advent of methods to perform C–H borylations and their advancement over the past three decades has continually increased the synthetic toolbox. This allowed for the functionalization of C–H bonds without prior halogenation, affording products well suited for rapid diversification via transition-metal-mediated cross-couplings.^4−13^ The lack of prior substrate functionalization is certainly advantageous from an atom-economic standpoint and can streamline approaches by eliminating the need to plan for undesired reactivity of the chosen functional handle; however, a noted barrier to advancing C–H borylations and other C–H functionalization reactions in late-stage synthetic planning is the ability to accurately predict the site selectivity of the reaction.^14−16^ As a result, the efforts of several groups have focused on developing protocols to control and understand the regiochemical outcomes in C–H borylations of aliphatic, arene, and heteroarene substrates.^17−19^ By leveraging transition metal catalyst/ligand combinations, substrates with metal-binding groups, as well as altering the steric and electronic properties of the substrate, modulation of the site selectivity is possible and has been used to effect ortho-,^20−25^ meta-,^26−29^ and para^30−32^ -selective arene C–H borylations. Furthermore, empirical guidelines for site selectivity in heteroarenes (e.g., C–H borylation occurs preferentially ortho to a ring oxygen or sulfur, but not next to a basic nitrogen) have been established by Hartwig and others, thereby assisting chemists in making informed decisions on the choice of substrate to use and to be able to consider limitations during synthetic planning when a late-stage C–H borylation is envisioned (Figure 1).^18^ While these guidelines are effective for simple systems with clear influences on selectivity (e.g., a single directing group), there are exceptions to the guidelines, and it can be difficult to extend these generalizations to more complex systems such as natural products or pharmaceuticals.^33^ The challenges associated with predicting whether or not a C–H borylation will occur in a regioselective manner present an opportunity to harness data analysis and classification machine learning algorithms to provide insights into inherent reaction, catalyst, or substrate features that can be utilized to aid in the prediction of site selectivity in C–H functionalization reactions.

Prediction of site selectivity is at the forefront of machine learning development in organic chemistry due to its potential to streamline synthetic planning and reaction optimizations.^34−47^ Models developed to predict the regioselectivity of catalytic difluorinations, electrophilic aromatic substitutions, and carbene-induced C–H insertions have employed neural network models, light gradient boosting, as well as logistic regression classification models, respectively, that were combined with quantum descriptor calculations and some used supplementary high-throughput experimentation (HTE).^48−51^

Complementing predictive models with HTE can be advantageous in allowing the generation of large quantities of experimental data to train the model. However, this approach can be limited in that the operational cost for HTE equipment is often high, such equipment requires trained operators for use, and is just beginning to become available beyond the industrial sector.^52^ On the contrary, the use of literature data alone to build and train machine learning models is cost-effective and widely available but not without its own limitations. This approach requires time to collate the necessary data, which may be incomplete due to the tendency of the literature to be limited to productive results. Literature data is also known to underreport unsuccessful results, causing an additional bias by merely training off of literature trends instead of creating generalizable models.^53^ With these considerations in mind, we opted to direct our investigation toward developing a supervised model to identify features that can contribute to the successful prediction of regioselective C–H borylations. Our initial investigations into this area identified the computed atomic charges at the individual borylation sites as important features to consider.^54^

Atomic charges have long been utilized in computational investigations using density functional theory (DFT) in combination with experimental data to evaluate possible reaction mechanisms and to provide insight into experimentally observed phenomena.^55−60^ Wiberg and Rablen recently conducted a thorough investigation at the MP2/6-311+G*, B3LYP/aug-cc-pVTZ, and B3LYP/6-311+G* levels of theory to evaluate several classic methods used to compute atomic charges at hydrogen.^61^ The computed hydrogen charges obtained from these methods were compared with experimental data, and several relationships to other parameters (e.g., hybridization, carbon acidity, known H···O bond energies, and high-resolution spectroscopic studies of deuterated alkanes) were identified. The high degree of correlation of atomic charges to several experimentally validated parameters prompted our investigation into atomic charges as potentially reliable predictors of the site of selective C–H functionalization.

Recently, three simultaneous reports of machine learning models aimed at investigating the site selectivity of C–H borylations were reported during the course of our work.^62−64^ Konrad, Grether, Martin, Schneider, and co-workers used HTE (ca. 950 reactions) and literature data (ca. 1300 reactions) combined with a geometric deep-learning program based on graphical neural networks (GNNs) to achieve predictive accuracies of 90% with F-scores of 60% for correct regioselective predictions of borylation sites.^62^ In a related study, Norrby, Hartwig, and co-workers collated 86 literature examples with 15 experimental examples of arene borylations and used a hybrid approach with computed transition state barriers and molecular fingerprints to develop a partial least-squares regression model (PLS) to predict the major sites of borylation in a 6-molecule validation set much better than chemists well-versed in C–H borylation.^63^ Goodman and co-workers shortly thereafter reported a nonquantum mechanical-based multitask language model trained on 1041 Ir-catalyzed borylations of aromatic C–H bonds acquired from literature databases and available in PubChem.^64^ The model was able to generate the correct product in 79% of cases by SMILES product prediction, and using site classification, the model achieved an F-score of 78% for correct regioselective predictions of borylation sites on the same data set^62^ used by Konrad, Grether, Martin, Schneider, and co-workers. Herein we report our complementary findings on how incorporation of experimental parameters available from the reported C–H borylation literature for transformations with a high degree of site selectivity (>95%) and supplementation of this data with quantum mechanical molecular and atomic descriptors of individual C–H borylation positions can be used to identify if a substrate-of-interest will undergo a site-selective borylation at the individual borylation site using a supervised, random forest classification model. This work further demonstrates that supplementing existing literature data with site-specific quantum mechanical information allows for the development of accurate models that can learn from smaller data sets and be used for the prediction of selectivity in C–H functionalization reactions.

## Results and Discussion

### Computational Methods and Model Development

Numerical molecular descriptors of reaction data were curated following a literature search of highly selective (≥95%) C–H borylation reactions, resulting in a database with 189 unique arene, heteroarene, and aliphatic substrates.^18−22,63,65−96^ The experimental parameters reported for site-selective borylations were extracted as model features and included: seven different organometallic catalysts [Ir, Ru, and Rh-based], eight different ligands [phosphine, phenanthroline, bipyridyl, cyclooctadiene, and arsine-based], were conducted neat or in solvent [e.g., octane, hexanes, THF], used either pinacolborane (HBpin) or bis(pinacolato)diboron (B_2_pin_2_) as the boronate ester source, and were conducted at a range of temperatures.

**Figure 2:**
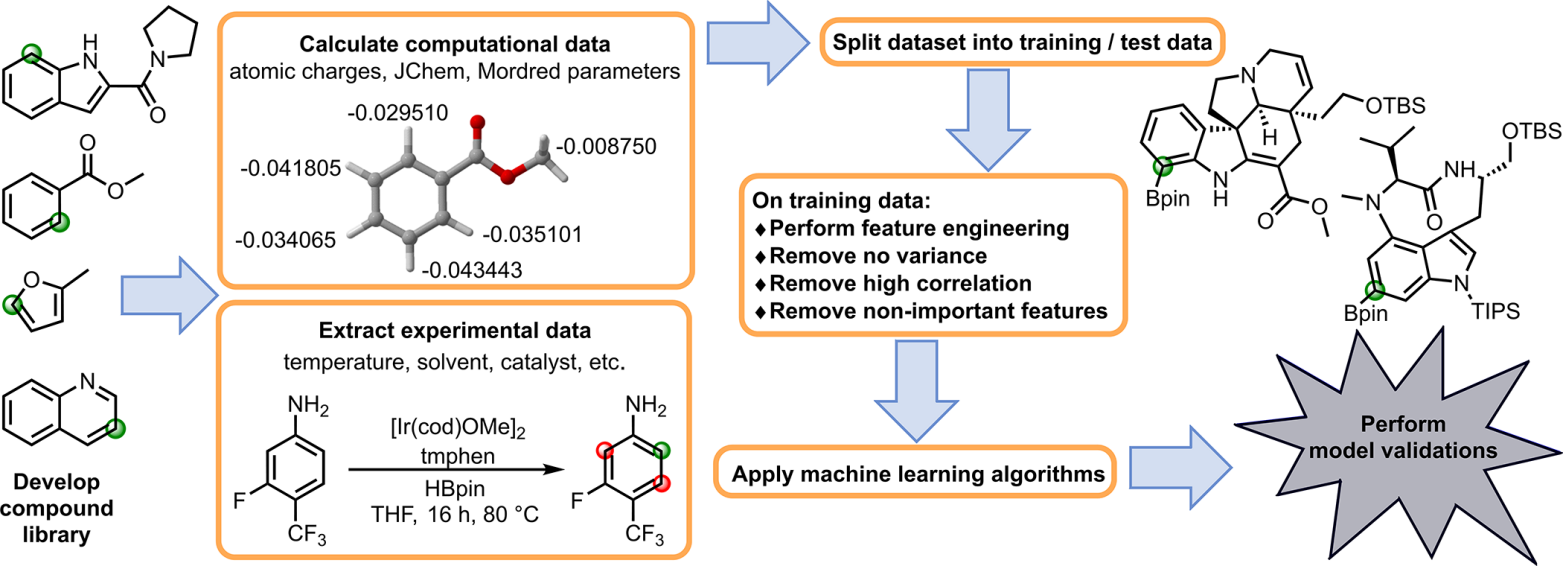
Flowchart of machine learning model workflow.

Each substrate was converted into an international chemical identifier (InChI) string, and the database was organized with each possible borylation site as a separate entry to allow for use of atomic descriptors. A general workflow for model development is shown in Figure 2.

Geometries were optimized without constraint at the B3LYP/6-311+G** level of theory using Gaussian 09 software and were used to compute a set of atomic charges for each potential site of borylation within the molecule. The charges on the hydrogen and carbon atoms at the potential C–H borylation site, as well as the carbon atom charge with the hydrogens summed (referred to as “heavy atom charges”) were computed using each of the following atomic charge calculation methods: Mulliken population analysis, natural population analysis (NPA), the minimal basis set (MBS) method, Hirshfeld population analysis, Charge Model-5 (CM-5), and the Charges from Electrostatic Potentials using a Grid-based method (ChelpG).^61,97−102^ Mordred descriptors (∼1600) were calculated for each substrate using the Python-based RDKit library package,^103,104^ and JChem for Excel by ChemAxon was used to calculate additional numerical descriptors,^105^ including Topological Steric Effect Index (TSEI) and atomic/molecular polarizability.^106^ Each of the chemoinformatic descriptors was compiled into a Pandas DataFrame, totaling 1680 columns. Model development also included meaningful feature engineering and a comparison of classification machine learning algorithms. Model training and accuracy testing were performed by randomly dividing the data set by substate entries such that 80% of the substrates were part of the training set and 20% were included in the test set. This method ensured that no substrate entry was included in both the training and test set.

### Feature Engineering and Model Training

Provided that a single borylation site is present in each substrate and that the possibility exists for more than one nonborylating site, there was a class imbalance within the data set (189 borylating sites to 798 nonborylating sites). Random oversampling was applied to the minority class of the training set such that the model was trained on an equal number of entries from each class (borylating and nonborylating).

To avoid overfitting caused by model overcomplexity, the number of features in the training set of each model type were reduced by removing zero variance features and those exhibiting a correlation greater than 95% to another feature. Further reduction of dimensionality was accomplished by applying a random forest classifier to the data set and selecting features greater than the mean importance using the SelectFromModel function available from the scikit-learn library. Subsequently, collinear features were clustered together using Ward’s linkage method of hierarchical clustering on the data set allowing for further feature reduction for model evaluation.^107,108^

The data set was screened against eight different machine learning algorithms using ten different random training/test sets (split at 80:20). Entries 1–7 were each evaluated using four different Ward’s linkage threshold settings. The accuracy scores of each model are reported in Table 1 alongside the standard deviations. The support vector machines, multilayer perception, Gaussian process, and logistic regression models were normalized using the scikit-learn function, normalize. Entry 8 consists of a graph neural network model using extended-connectivity fingerprints for each substrate.^109,110^ Of the algorithms explored, the random forest classification model yielded the highest average accuracy score of 88.9% ± 2.5% across ten model runs and was selected for further development to predict site-selective borylations (Table 1). Gaussian process and decision tree models were less effective with average accuracy scores across ten model runs of 79.1% ± 2.8% and 77.2% ± 1.1%, respectively. The Naïve Bayes model exhibited the lowest accuracy score evaluated across ten model runs of 59.1% ± 2.8%.

**Table 1:** Comparison of Model Accuracy

entry	classifier	average accuracy score^a^
1	decision tree	77.2 ± 1.1%
2	SVM	81.9 ± 0.0%
3	random forest	88.9 ± 2.5%
4	multilayer perception	80.4 ± 1.4%
5	Gaussian process	79.8 ± 0.9%
6	Naïve Bayes	59.1 ± 2.8%
7	logistic regression	81.9 ± 0.0%
8	graph neural network	80.6 ± 1.3%

a The accuracy scores reported are taken from the average of 10 model runs, and the associated standard deviations are reported.

Notably, of the over 1600 original parameters, 8 out of the final 35 important features in the developed random forest model to predict site-selective borylations were computed atomic charges. The specific atomic charge composition of the final features included the following computed charges: Hirshfeld heavy atom, Hirshfeld carbon, Hirshfeld hydrogen, ChelpG heavy atom, ChelpG hydrogen, NPA hydrogen, Mulliken heavy atom, and Mulliken hydrogen. The final features included four JChem for Excel functions including the steric effect index, a value of relative volume that substituents occupy around a reaction center; atomic polarizability of the potentially bonding carbon; distance degree, a topology calculation of bond distances; and Dreiding energy, a calculation of molecular energy of a molecular conformation using the Dreiding force field. A total of 23 Mordred descriptors were used as the final parameters in the developed model. Of the 23 Mordred parameters, 20 were calculated using topographical autocorrelation descriptors to include variations of the Moreau-Broto autocorrelation values (AATS 2d, AATS 1s, AATS2Z, AATS0v, AATS 4p, AATS1i, ATSC 1d, ATSC 2d, AATSC2dv, AATSC 4d, AATSC3Z, AATSC5v, AATSC1pe); Moran coefficient values (MATS1c, MATS 5s, MATS 1p); and Geary coefficient values (GATS3c, GATS4c, GATS1v, GATS2pe). The final three Mordred parameters used in the model are the topological descriptor BCUTZ-1l that describes the molecular graphs of the compound, the relative positive charge (RPCG), and FilterItLogS, an estimate of aqueous solubility.

**Figure 3:**
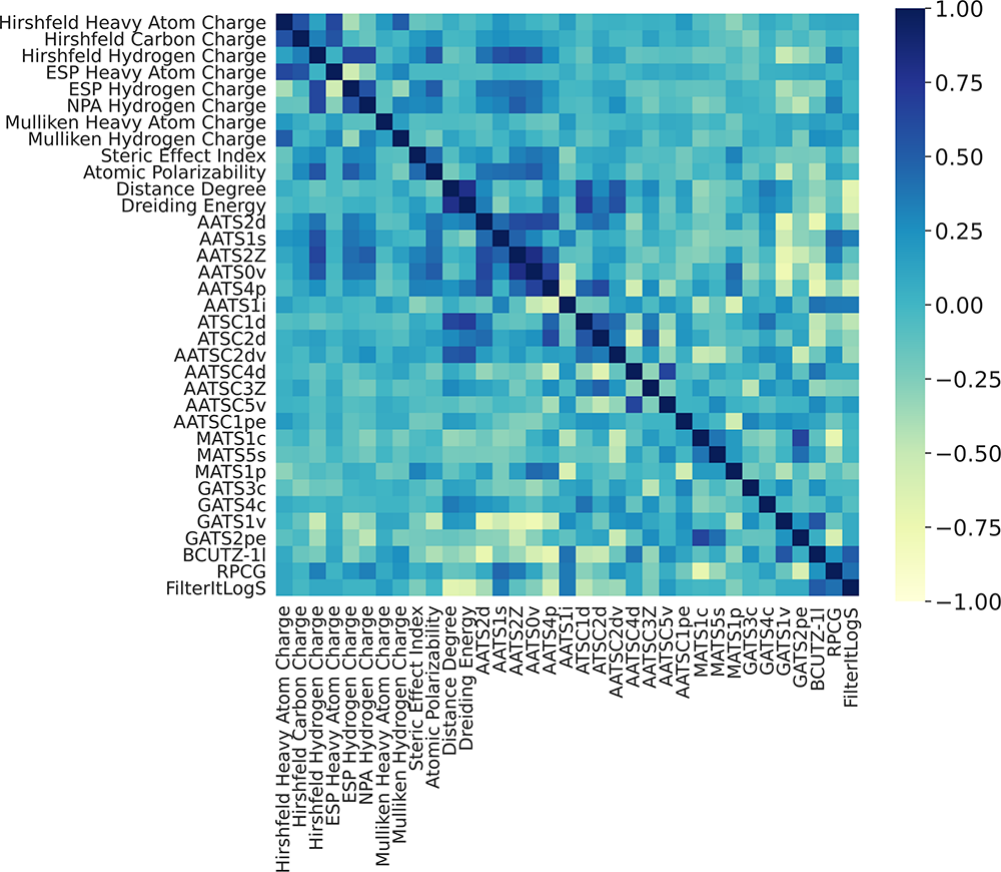
Spearman correlation matrix of final parameter set.

The degree of correlation between each of these 35 final parameters was examined via a Spearman correlation matrix revealing a moderate correlation (between −0.77 to 0.81) among the final features (Figure 3).

### Feature Importance and Exploration

**Figure 4:**
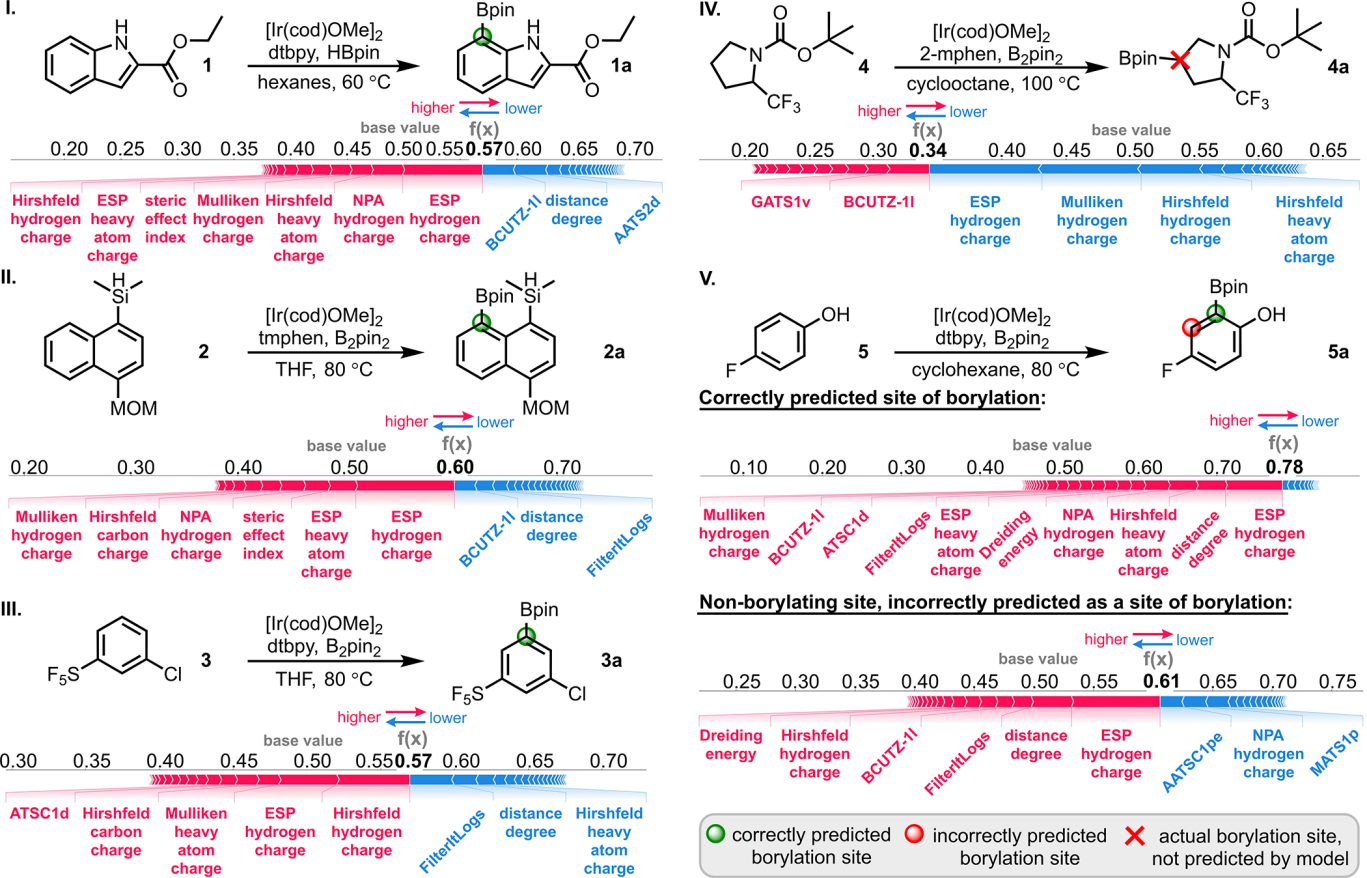
Shapley force plots of random forest model predictions.

The developed random forest classification model was trained by using a diverse set of chemoinformatic descriptors. Therefore, it was of interest to assess the importance of each of the features to successfully predict site-selective borylations.^111^ Shapley force plots were generated to gain insight into the effect features have on the model’s prediction (Figure 4).^112^ SHapley Additive exPlanations (SHAP) analysis plots increase model interpretability by calculating the contributions of each parameter to model prediction decisions. Features that are important contributions to the model’s prediction are shown in red, while those that contribute negatively are shown in blue. Entries I–III show feature contributions that led to the prediction of the correct site of borylation in compounds **1**-**3**. The correct predictions heavily relied upon charge parameters to form a decision value higher than the base value of 0.5. In entry IV, the model was unable to predict the correct site of borylation in **4** as the decision value of 0.34 was <0.5 leading to a prediction that no borylation occurs. In this case, the correct site of borylation was not predicted by the model as the computed charges for **4** were contributing against borylation, as seen in the blue colored entries. Entry V shows an example of where the model predicted both the correct site of borylation in **5** and an additional nonborylating site as the atomic charges and distance degree features lead to a borylation prediction at both sites.

Experimentally, the selection of a particular catalyst and ligand combination can affect both the site selectivity and the overall yield of the reaction, thus both features are likely to be important model attributes (e.g., Figure 1).^22,27,113^ Therefore, it was prudent to investigate how the model interprets different catalysts and ligands used for C–H borylations as part of the decision-making process. The majority of site-selective C–H borylations have been reported with Ir-based catalysts (e.g., 183 of 189 substrates in the training set) compared to Ru- and Rh-based catalysts. Several methods to characterize the reaction catalysts and ligands using the data set were investigated. In the first method explored, numerical variables were used to separately encode the catalyst (e.g., [Cp*RuCl_2_]_2_ = 1, [Cp*RuCl]_4_ = 2, ...) and ligand selection (e.g., no added ligand = 1, dtbpy = 2, ...).^114^ This rudimentary method failed to identify the catalyst or ligand as an important feature within the feature engineering sequence. To provide a set of discrete catalyst–ligand descriptors based on quantum mechanical calculations, the Rh, Ru, and Ir-based catalysts and relevant ligand combinations were parameterized utilizing methods developed by Gensch and co-workers for parameterizing organophosphorus ligands.^115^ To capture the influence of the ligand, the geometries of the active catalyst resultant from each of the ligand-catalyst combinations used were computed at the RB3LYP/LANL2DZ level of theory (See Figure S2 of the Supporting Information). The starting geometries for the active catalysts were appropriated and modified from atomic coordinates reported by Zhong and Sakaki during their computational investigations into Ir-, Ru-, and Rh-mediated C–H borylations of methane and cyclohexane, and correspond to the geometries preceding oxidative addition into the C–H bond of the substrate.^116^

Among the parameters computed were the percent buried volume of the metal center, Sterimol steric parameters, pyramidalization about the metal center, solvent accessible surface area (SASA) and volume, and additionally, separate column entries for numerical encoding of each catalyst, ligand, and the active catalyst were included.^115,117^ The active catalyst was added to express that many of the catalyst–ligand combinations ultimately result in the *in situ* formation of the same active catalyst.^118^ However, the additional parameterization features of the active catalyst resultant from the different catalyst–ligand combinations were not included within the final feature selection. This finding was not unexpected given the paucity of literature examples available for model training within the data set that feature the same model substrate with different catalyst–ligand combinations that lead to divergent site selectivity. Therefore, the catalyst and ligand would not be identified as important features during model development. Candidly, this is a result of how synthetic methodologies are optimized and ultimately reported in the literature. A representative model substrate is often chosen and screened against several catalyst–ligand combinations under differing conditions to identify an optimized protocol, which is then evaluated for generality and scope by extension to additional substrates. As a result, a single set of optimized conditions is evaluated against the additional substrate scope and reported. Thus, the data needed to determine the effect of differing catalyst–ligand combinations across different substrates is often not readily available from the literature or published high-throughput data sets.

**Figure 5:**
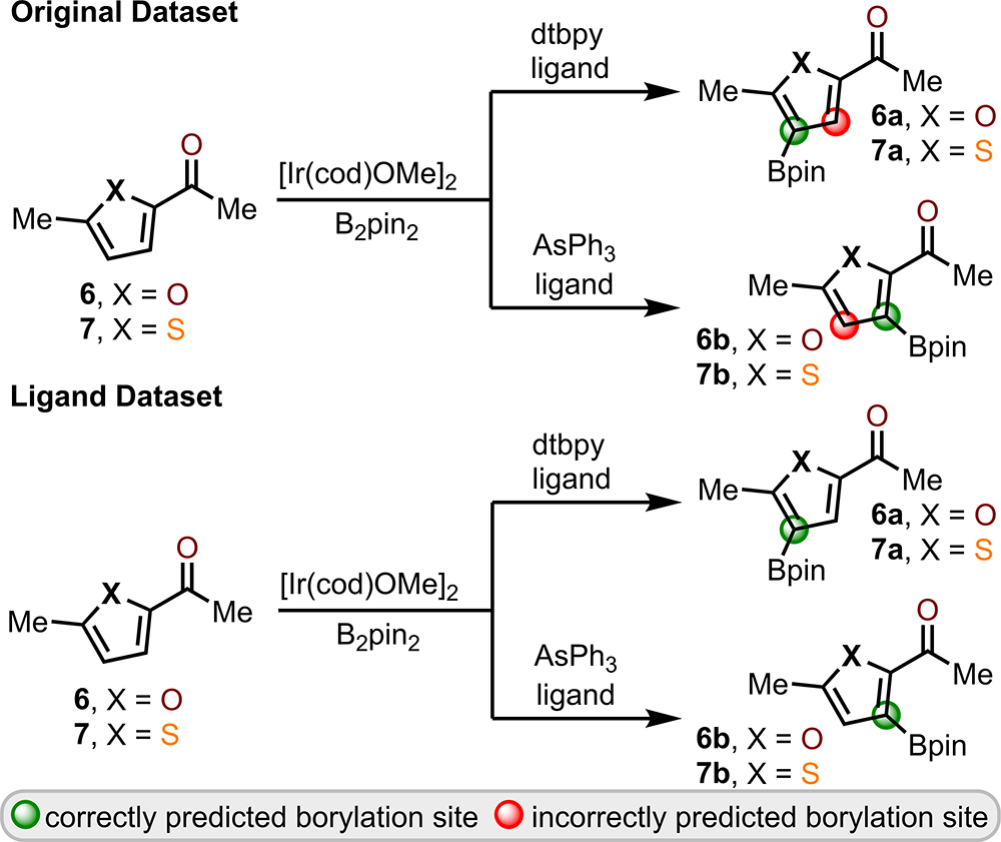
Validation set used to demonstrate model effectiveness when trained on the “ligand data set”.

To further probe the model’s ability to utilize the catalyst and ligand as part of the decision-making pathway, the model was trained and tested on a second, separate data set. This “ligand” data set included additional entries wherein substrates already within the data set were evaluated against different catalyst–ligand combinations that altered the regiochemical outcome of the C–H borylation.^70^ With this modification, the model was now able to train on multiple entries of a given substrate that afforded different regiochemical outcomes based on the catalyst–ligand selection. The original and ligand data set were compared against a validation set that included furan **6** and thiophene **7** (Figure 5). Prior to the data set modifications made with the ligand data set, the model was unable to identify that the use of a dtbpy ligand versus an AsPh_3_ ligand results in different site selectivity during C–H borylation of **6** or **7**. After retraining, model prediction of the nonborylating and borylating sites within substrates **6** and **7** improved with 16 of the 16 potential sites predicted correctly. This shows the ability of the model to account for the effect of the active catalyst–ligand combination on site selectivity but also signifies the need for more training data wherein different catalyst–ligand combinations are used on the same substrate than is currently available from literature examples alone.

**Figure 6:**
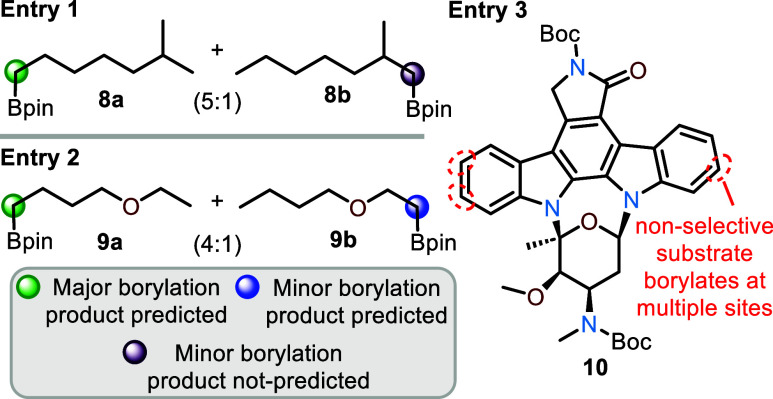
Model performance on substrates with <95% site selectivity for C–H borylations as well as nonselective substrates.

In addition to examining the effects of catalyst–ligand combinations, we wanted to determine how the model would perform when less selective (<95% regioselectivity for a single position) or nonselective (>than two sites) borylation reactions were used as inputs (Figure 6). The results indicate that while the model was trained on selective reactions (≥95% regioselectivity for a single position), it performs well on less selective borylations, which helps gauge the degree of selectivity predicted by the model. As shown in Figure 6, entry 1, 2-methylheptane has an experimentally measured site selectivity of 5:1 for **8a** over **8b**, and the model can distinguish the major product **8a** from the two potential borylating sites (Figure 6). In entry 2, 1-ethoxybutane is less selective with an experimentally measured selectivity of 4:1 favoring **9a** over **9b**, and in this example, the model indicates both are viable C–H borylation sites. Furthermore, when *tert*-butoxycarbonyl-protected staurosporine (**10**) is evaluated, which experimentally exhibits nonselective borylation at multiple sites (with several product combinations at the highlighted sites in Figure 6),^33^ the model does not indicate that a selective borylation will occur; thus, the model is able to limit false positives of nonselective molecules. Overall, the model indicates whether an ≥ 5:1 selectivity is expected experimentally with prediction of borylation at a single site, whereas when moderately selective (≤4:1) borylations are expected both borylation sites are predicted, and for nonselective systems, no selective borylation sites are predicted by the model.

**Figure 7:**
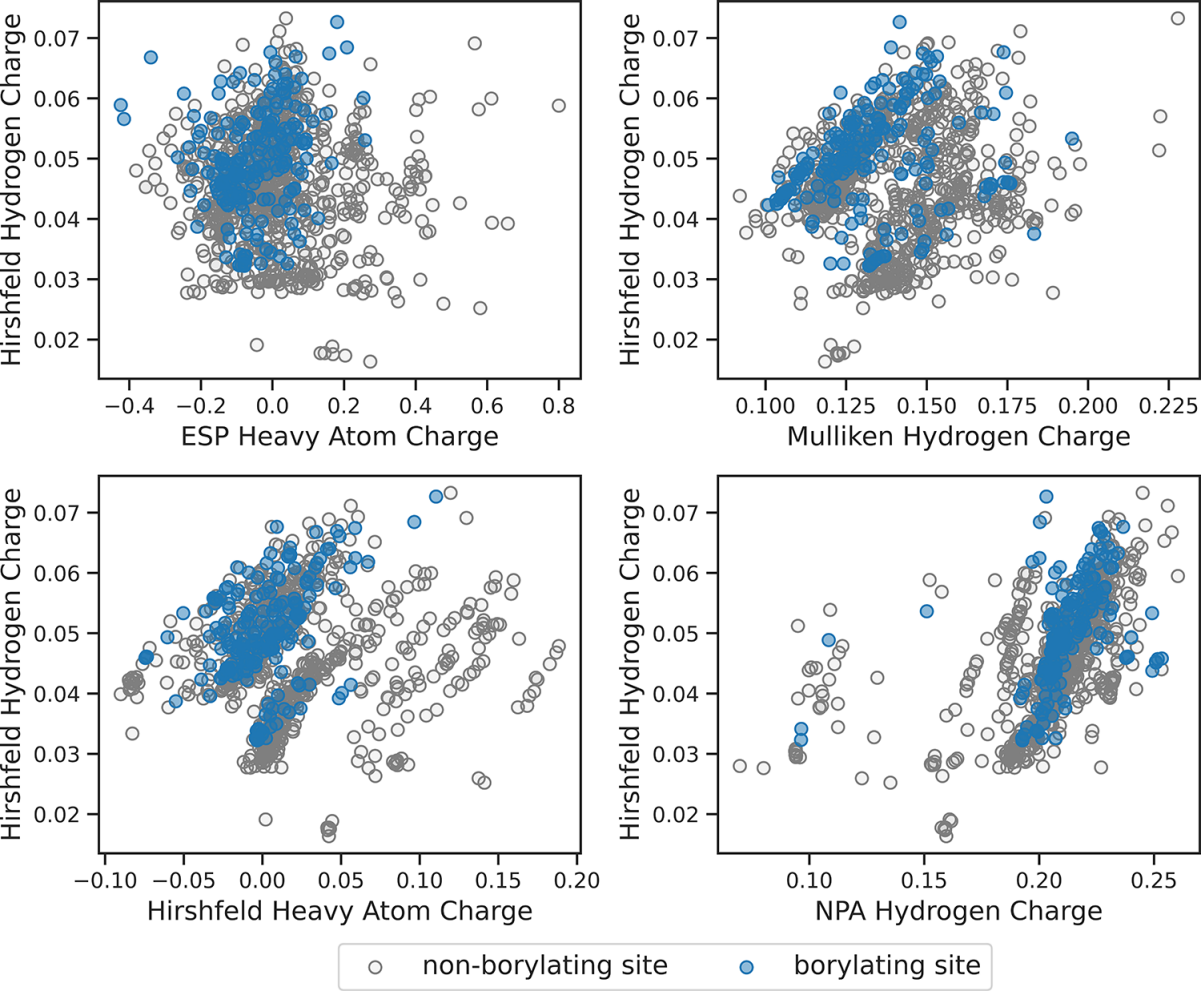
Scatter plots of data set atomic charges for each model entry. Sites of borylation are indicated by blue circles, and sites that do not undergo borylation are indicated by gray circles.

To further examine model performance using atomic charges, the Hirshfeld charge on the hydrogen atom of both borylating (blue circles) and nonborylating sites (gray circles) of individual entries from within the original data set were plotted against the ChelpG (ESP) charges on heavy atoms, Hirshfeld charges on the heavy atoms, the Mulliken hydrogen atom charges, and the NPA hydrogen atom charges (Figure 7). In each plot, the sites of borylation were overlaid upon the nonborylating sites of the same structures. As can be seen from these plots, varying degrees of clustering are observed for the sites of borylation; for example, those sites which have computed Hirshfeld hydrogen atom charges between 0.04 and 0.06 e and ESP heavy atom charges between −0.2 and 0.1 e tended to undergo site-selective borylation. A similar observation can be made with sites that had computed Hirshfeld hydrogen atom charges between 0.04 and 0.06 e and Mulliken hydrogen atom charges between 0.10 and 0.14 e. Several of the nonborylating sites had computed charges outside of these ranges. Further comparison of the Hirshfeld charges on the hydrogen atoms versus Hirshfeld charges on the heavy atoms (carbon) or natural population analysis (NPA) hydrogen atom charges reveals linear trends among the data. The random forest model makes informed decisions within these trends as with entry IV of Figure 4. Entry IV highlighted an example of a substrate in which the model failed to predict the correct site of borylation. Upon further probing of the atomic charges, the Hirshfeld hydrogen atom charge was 0.038 e and the Mulliken hydrogen atom charge was 0.18 e. This entry falls outside the charge ranges for typical site-selective borylations shown in Figure 7, preventing the model from making a correct prediction.

Both electronic factors and steric factors are experimentally important in Ir-catalyzed C–H borylations of arenes and heterocycles.^18,60,73^ Some time ago, Maleczka, Singleton, Smith, and co-workers studied the influence of ring electronics on the reactivity and regioselectivity in Ir-catalyzed arene C–H borylations of unencumbered substrates.^60^ They observed that the computed energy difference (Δ*E*) between ground-state C–H borylation reactants and intermediates was linearly correlated with both the computed transition state activation energies (*R*^2^ = 0.96) and the total natural population analysis (NPA) charge on the aryl and heteroaryl groups (*R*^2^ = 0.95). Thus, electronic changes in the arene influence reactivity in Ir-catalyzed C–H borylations, with increased steric interactions in arenes resulting in higher computed energies compared to the five-membered heterocycles evaluated. Our developed model relies heavily on atomic charges as revealed by the (SHAP) analysis plots (Figure 4) and scatter plots in Figure 7. However, incorporating the steric effect index, a relatively simple steric parameter, helps balance the predominantly charge-driven decision pathways by accounting for steric influences within the substrate, thereby improving the model’s accuracy in classifying borylation sites. A similar observation was made by Paci, Leitch, and co-workers who developed a multivariate linear regression model for catalytic cross-coupling reactions that used average molecular electrostatic potential (ESP) as electronic descriptors and substrate A-values as steric descriptors.^119^ Their reactivity model showed robust predictive power, despite ESP values at the carbon undergoing substitution being the most consequential descriptor (*ca*. 50% contribution) in predicting the activation energy of the oxidative addition, whereas steric descriptors, represented by A-values, contributed less than 10%.

Our model accuracy and validity were further probed by control studies, ablation studies split by feature attributes within the data set, and an additional data set of compounds that represent the chemical space. This methodology underpins the selectivity guidelines of the model and further points toward the reliability of the computed atomic charges.

### Model Generalizability and Ablation Studies

Control experiments were performed to gauge the robustness of the model. Each control experiment was conducted using the highest performing algorithm, which was random forest classification (Table 2). Entry 1 consists of the highest performing model with the final 35 parameters resulting in an overall 88.9 ± 2.5% accuracy. Model accuracy dropped to 85.5 ± 0.7% when no feature selection was performed (entry 2). This reduction in model accuracy is due to the tendency of random forest models to overfit the test set when given too many correlated parameters. An additional control experiment was conducted with each of the target parameter values randomly shuffled (entry 3). While model accuracy only dropped to 75.8 ± 2.8%, the F1 score for predicting sites of borylation dropped to only 16.6 ± 6.4%. A final control experiment was conducted in which the order of the final 35 parameters is shuffled, and the model accuracy did not change an appreciable amount (87.8 ± 0.7%, entry 4). While entries 1 and 4 exhibited the highest overall accuracy, when only given the charges and experimental conditions (entry 5), model accuracy was comparable at 85.0 ± 0.7%. This pattern was continued with each of the ablation study entries, with those consisting of primarily charges having high accuracy scores and better F1 scores for borylation predictions (entries 6 and 11–14). The retrained “ligand” data set was also tested against the additional substrates and had a slightly lower accuracy score than the top performing model (84.8 ± 1.0%).

**Table 2:** Examination of Model Generalizability on a Validation Set and Ablation Studies^a^

entry^a^	classifier	accuracy	precision (nonborylating/borylating)	recall (nonborylating/borylating)	F1 (nonborylating/borylating)
1	charges, exp., Mordred, and JChem w/top 35 parameters	88.9 ± 2.5%	93.9 ± 1.7%/68.0 ± 4.8%	92.4 ± 1.6%/72.8 ± 5.8%	93.1 ± 1.6%/70.3 ± 5.1%
2	charges, exp., Mordred, and JChem w/all features present	85.5 ± 0.7%	90.2 ± 0.3%/65.4 ± 2.3%	91.7 ± 0.7%/61.1 ± 1.4%	91.0 ± 0.5%/63.1 ± 1.6%
3	charges, exp., Mordred, and JChem w/target values shuffled	75.8 ± 2.8%	80.9 ± 2.4%/27.1 ± 13.2%	91.3 ± 3.1%/12.4 ± 4.9%	85.8 ± 1.9%/16.6 ± 6.4%
4	charges, exp., Mordred, and JChem w/order of top 35 features shuffled	87.8 ± 0.7%	93.9 ± 0.3%/61.6 ± 2.3%	91.3 ± 0.8%/70.0 ± 1.5%	92.6 ± 0.5%/65.5 ± 1.6%
5	charges and exp.	85.0 ± 0.7%	93.8 ± 0.3%/59.8 ± 1.6%	87.0 ± 0.9%/77.0 ± 1.4%	90.3 ± 0.5%/67.3 ± 1.1%
6	charges only	80.9 ± 2.9%	90.1 ± 2.4%/52.7 ± 8.5%	85.7 ± 4.3%/62.2 ± 8.8%	87.8 ± 2.0%/56.2 ± 5.0%
7	Mordred and exp.	62.1 ± 4.0%	84.5 ± 1.3%/27.0 ± 1.9%	64.4 ± 6.5%/52.3 ± 7.9%	72.9 ± 4.3%/35.5 ± 3.0%
8	Mordred only	64.8 ± 5.3%	84.6 ± 0.7%/26.3 ± 1.7%	68.7 ± 8.6%/46.6 ± 9.7%	75.6 ± 5.7%/33.4 ± 3.3%
9	JChem and exp.	78.7 ± 1.0%	85.4 ± 0.2%/40.1 ± 2.8%	89.2 ± 1.3%/32.2 ± 0.9%	87.2 ± 0.7%/35.7 ± 1.4%
10	JChem only	73.8 ± 2.2%	87.2 ± 0.9%/36.4 ± 2.6%	79.3 ± 2.9%/50.3 ± 4.4%	83.0 ± 1.8%/42.2 ± 2.9%
11	charges, JChem and exp.	87.4 ± 1.7%	93.6 ± 1.3%/66.7 ± 4.1%	90.3 ± 1.9%/75.8 ± 4.5%	91.9 ± 1.2%/70.8 ± 2.9%
12	charges and JChem	83.6 ± 0.5%	92.5 ± 0.6%/54.9 ± 1.2%	87.0 ± 0.7%/69.2 ± 2.9%	89.6 ± 0.3%/61.2 ± 1.6%
13	charges, Mordred and exp.	83.0 ± 0.7%	91.1 ± 0.4%/53.3 ± 1.6%	87.7 ± 0.6%/61.9 ± 2.0%	89.4 ± 0.4%/57.2 ± 1.6%
14	charges and Mordred	84.8 ± 2.1%	92.2 ± 1.4%/60.7 ± 6.3%	88.6 ± 2.8%/69.6 ± 5.9%	90.3 ± 1.4%/64.6 ± 4.6%
15	ligand data set	84.8 ± 1.0%	92.2 ± 0.6%/59.6 ± 2.4%	88.7 ± 0.9%/69.0 ± 2.7%	90.4 ± 0.6%/64.0 ± 2.2%

a Scores are reported as averages of 10 model runs alongside the corresponding standard deviation.

**Figure 8:**
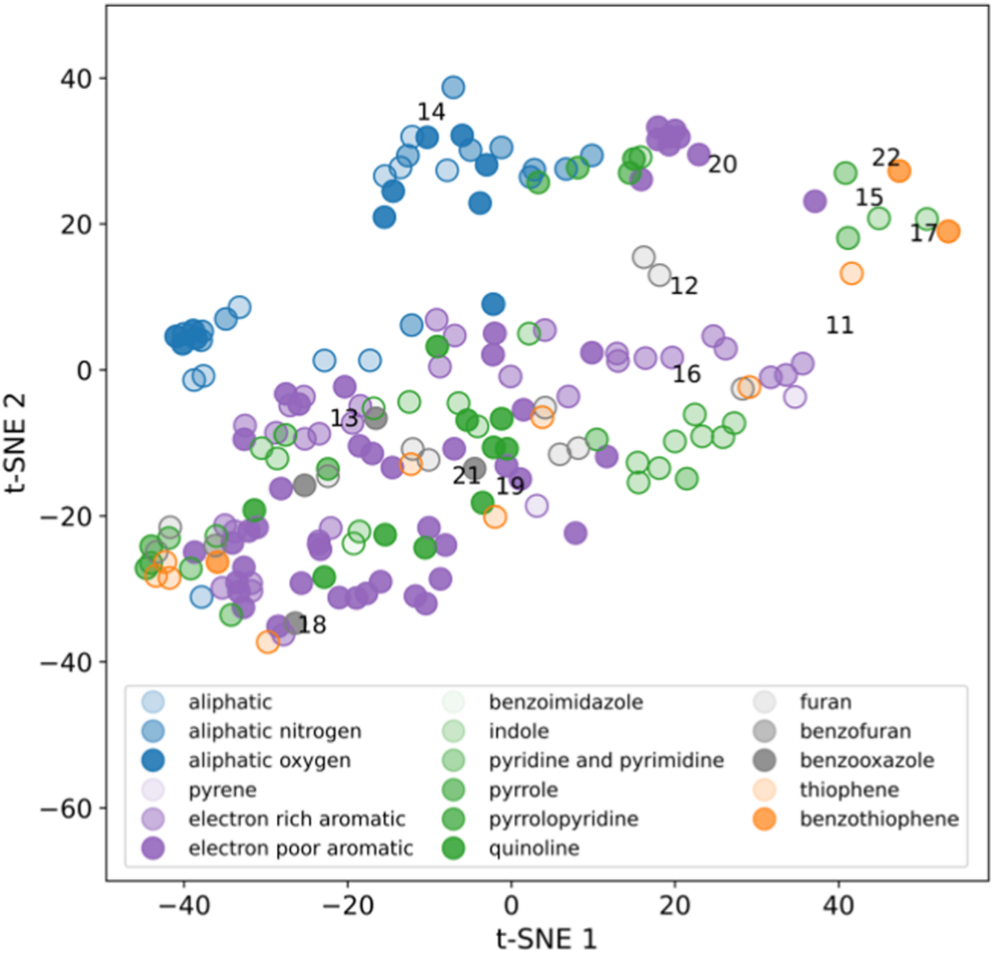
Projection of model data set using t-SNE clustering with compounds included in the additional data set labeled **11–22** illustrating their range of structural and electronic diversity.

The data set’s chemical space was visualized using the t-SNE clustering algorithm (Figure 8).^120^ The t-SNE plot graphically shows the diversity of compounds within the data set, which is representative of the overall chemical space of site-selective C–H borylation substrates in the literature including aliphatic, ethers, amines, nitrogen heterocycles, furans and thiophenes, and electronically varied aromatics.

**Figure 9:**
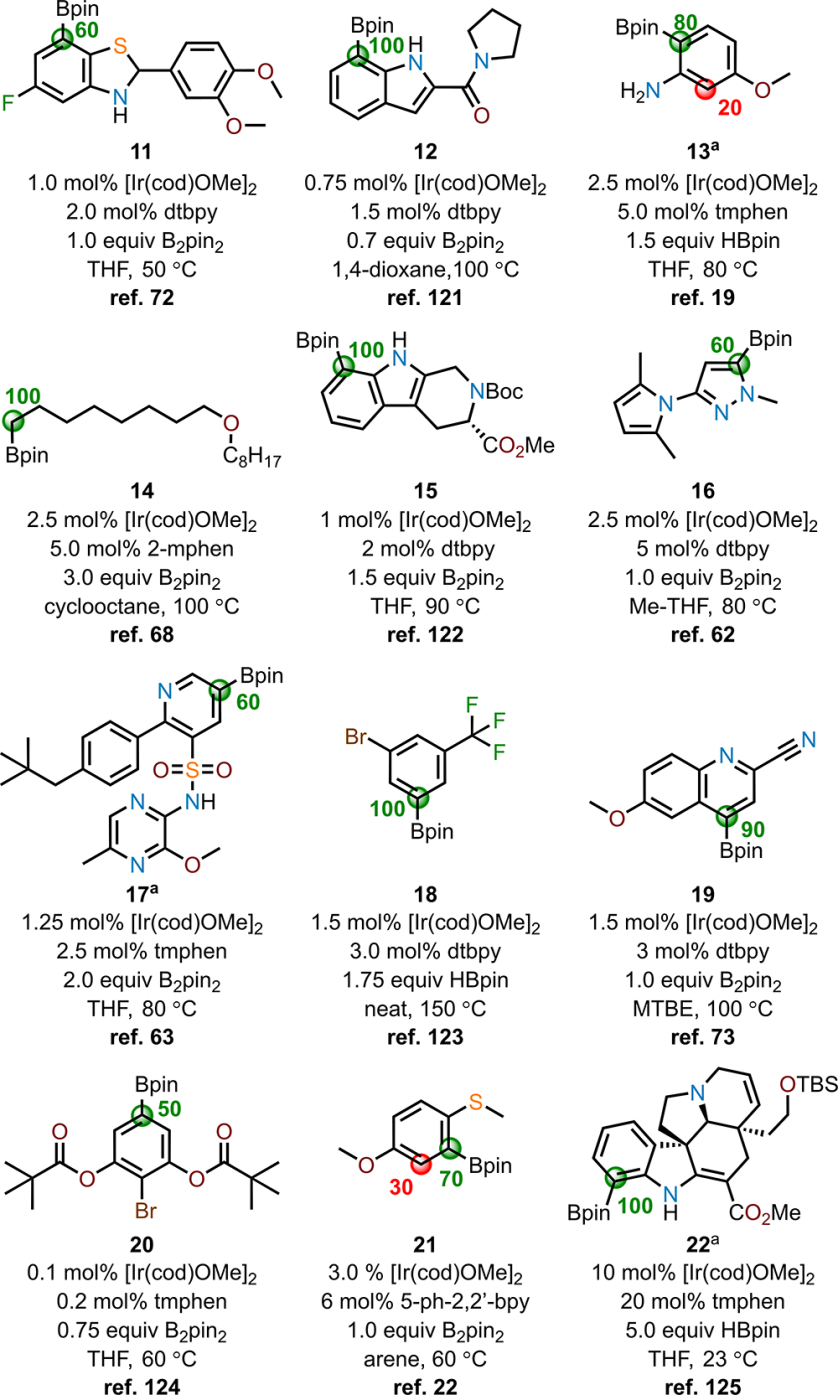
Compounds within the additional data set. Correctly predicted sites of borylation are indicated as green circles; improperly predicted sites of borylation are shown as red circles. Numerical values next to the circles indicate the percent of prediction when the model is run 10 times. ^[a]^These molecules had an additional site of borylation predicted 10% of the time that was removed for the sake of clarity.

To examine the model’s ability to correctly predict compounds from outside of the training and test set, we tested the model against an additional set of compounds (**11**-**22**) chosen for their range of structural and electronic diversity and that capture a wide-reaching portion of chemical space (Figure 9).^19,22,62,63,68,72,73,121−125^ The random forest model showed high and general performance in selecting the correct site of borylation, denoted by the site highlighted in green. The incorrectly predicted sites of borylation are labeled with red spheres. Each colored sphere is labeled with a numerical value representing the percentage of model predictions for that site of C–H functionalization with 100% representing 10 model predictions out of 10 model runs. This extra data set revealed that the model is particularly effective for predicting site-selective borylations on nitrogen heterocycles (e.g., indoles, indolines, pyridines, pyrazoles; **11–12**, **15–17**, **19**, and **22**), aliphatic systems (e.g., **14**), and hindered arenes (e.g., **18** and **20**). Lower performance was observed on electron-rich arene substrates that selectively borylate in the *ortho*-position (e.g., **13** and **21**), which were predicted correctly by the model as the major site of borylation but with an incorrect site prediction occurring in 2 or 3 of the 10 runs of the model. This result arises from **13** and **21** having similarities in the computed atomic charges and steric effect indices at both sites as well as the absence of the ligand parameters within selected features.

To provide a comparison of the developed random forest model to the graphical neural network and partial least-squares models recently reported, pyrazole **16** used by Konrad and co-workers^62^ and nitrogen heterocycle **17** reported by Hartwig and co-workers^63^ were included in our validation set. The correct site of borylation was predicted for both molecules using the random forest algorithm with 6/10 predictions for **16**, which falls well within the chemical space covered by the training set and 6/10 predictions for **17**, which populates the periphery of the chemical space used in model development.

**Figure 10:**
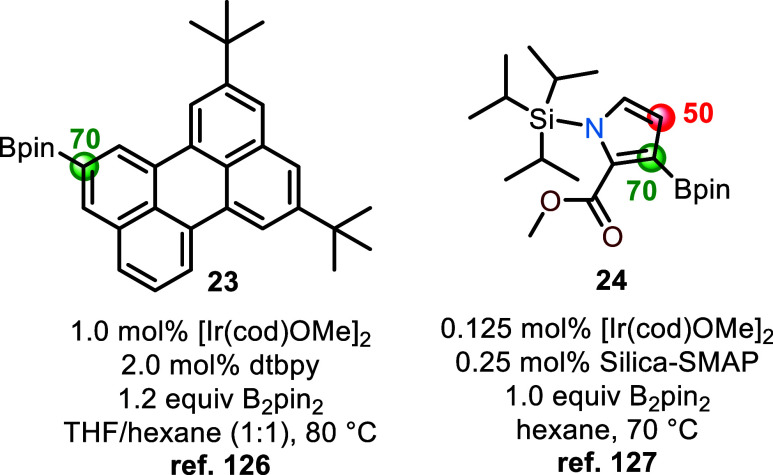
Evaluation of the developed random forest classifier model using only computed atomic charges and Mordred parameters against compounds **23** and **24**. Correctly predicted sites of borylation are indicated as green circles, improperly predicted sites of borylation are shown as red circles. Numerical values next to the circles indicate the percent of prediction when the model is run 10 times.

The performance of the developed random forest classifier model using only computed atomic charges and Mordred parameters (entry 14, Table 2) was evaluated against two unseen compounds, **23** and **24**, to gain insight into whether physically interpretable descriptors are indispensable for accurate site selectivity predictions (Figure 10). Compounds **23**(126) and **24**(127) are known to undergo site-selective borylation experimentally, but this reactivity is not predicted by nonquantum mechanical-based multitask language models,^64^ thereby enabling comparison of our atomic charge-based model to DFT-free machine learning approaches. The correct C–H borylation site in **23** was predicted by the model when the computed atomic charges alongside the Mordred molecular descriptors were used with 7/10 predictions. When compound **24** was evaluated, the major borylation site *ortho* to the ester was predicted in 7 out of 10 model runs, while an additional borylation site *meta* to the ester was predicted in 5 out of 10 runs. We chose to evaluate a model that excluded experimental data and atomic-level steric parameters (e.g., steric effect index at the reacting carbon) to better gauge the charge importance. While a complete consensus was not achieved in **24**, the model predicts C–H borylation at the sterically hindered, experimentally observed *ortho*-position more so than the less sterically demanding *meta*-position. This may be due to the fact that metal-catalyzed C–H borylations of heterocycles exhibit a greater degree of electronic regiocontrol than arenes, owing to the electronic activating effects of the heteroatom,^128^ which are accounted for by using computed atomic charges. When the atomic charges were removed as features and the DFT-free Mordred molecular descriptors were used alone, the model failed to predict the borylation sites in both **23** and **24**. This highlights how incorporating atomic charges at the carbon and hydrogen atoms undergoing oxidative addition to the low-valent metal as descriptors enhances the model’s predictive power.

**Figure 11:**
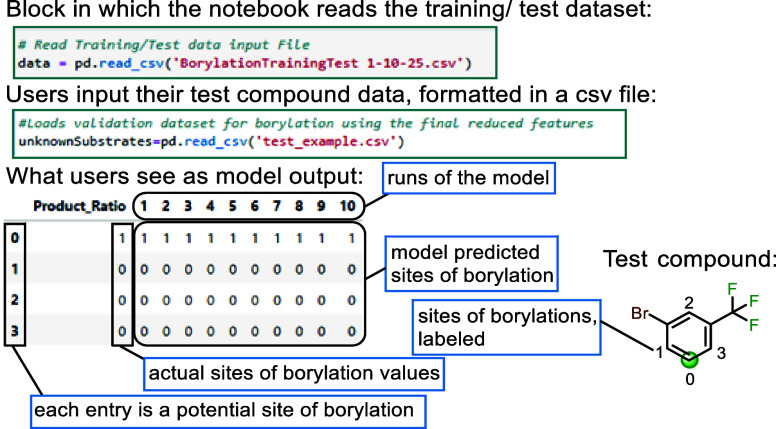
Example of the model output seen by the user.

Overall, the developed model sufficiently covers the known chemical space of site-selective C–H borylations reported in the literature to date, performs well across several classes of molecules, and can be used for the prediction of site selectivity of additional substrates. A general workflow for operation of the machine learning model is provided in the Supporting Information along with the associated Python code and instructions needed for users to run the model with their own substrate data set. This information is provided in a separate GitHub repository (see the Associated Content). To use the model, users enter the substrate of interest as an (InChI) string into a CSV file that contains the computed atomic charges at the possible borylation sites of interest, the Mordred and JChem for Excel parameters of interest, and a set of desired experimental conditions from those included in model development (see the Supporting Information, Table S2). Each entry in the output represents a possible borylation site within the molecule, and the result of the ten individual model runs are displayed as either a “0” = no borylation expected or a “1” = a site-selective C–H borylation is predicted to occur (Figure 11). This workflow requires the user to have a basic understanding of DFT calculations to appropriate the atomic charge data and the use of Python libraries in Jupyter Notebook to run the model.

## Conclusions

A supervised, random forest machine learning model has been developed that allows for the prediction of Ir-, Ru-, and Rh-catalyzed site-selective C–H borylations using standard experimental conditions. Model development was accomplished using experimental data reported in the literature for the site-selective C–H borylation (≥95%) of 189 diverse substrates (representing 971 possible C–H borylation sites) that was supplemented with data available from standard quantum mechanical calculations and chemoinformatic descriptor packages. Assessment of 1680 easily accessible chemoinformatic descriptors as possible model features reveals that Hirshfeld, ChelpG, and Mulliken atomic charges alongside an atomic steric effect index parameter are important features that allow for the successful prediction of site-selective C–H borylations. The influence of different Ir, Ru, and Rh-based precatalyst/ligand combinations on site selectivity was investigated through parametrization of the computed geometries of the active catalyst. The developed model was shown to learn from available data wherein using a different precatalyst/ligand combination can alter the site of C–H borylation within a given substrate and is able to make successful predictions based on a given precatalyst/ligand combination as an input. This evaluation also illuminates the limited availability of such data in current literature as many reported C–H borylation substrates tend to afford the same site selectivity as the employed precatalyst/ligand combinations result in geometrically similar active catalysts. Future use of HTE could be used to assist in the generation of such data for inclusion in the model. Ablation and validation studies further highlight the ability to use computed atomic charges and atomic-level steric effect indices as productive features in the development of generalizable machine learning models to predict site selectivity in late-stage C–H functionalization reactions and can serve as a cost-effective alternative when access to perform HTE is limited.

## Data Availability

The data underlying this study are available in the published article, in its Supporting Information, and openly available on GitHub at https://github.com/LambertGroupChemistry/CH-Borylation-Model.

## Supporting Information Available

Data associated with the computational methods, library of compounds, experimental parameters used, table of contents for GitHub repository, model operation guide, and computed structures data (PDF)
