# Supplementary material for: The Importance of Atomic Charges for Predicting Site-Selective Ir-, Ru-, and Rh-Catalyzed C–H Borylations
Source: J Org Chem. 2025 Apr 23;90(17):6000–12. doi: 10.1021/acs.joc.5c00343 (PMC12053941; doi:10.1021/acs.joc.5c00343)
Supplement: Supplementary file 1 — jo5c00343_si_001.pdf [file jo5c00343_si_001.pdf]

Supporting Information for

The Importance of Atomic Charges for Predicting Site-Selective Ir, Ru, and Rh-Catalyzed C-H Borylations

Shannon M. Stephens and Kyle M. Lambert*

Department of Chemistry and Biochemistry, Old Dominion University, 4501 Elkhorn Ave, Norfolk, VA 23529, United States

Table of Contents

1. Computational Methods S2
   1.1 Chart S1 Compounds used within the training/test library: S2
   1.2 Table S1. Compound experimental data and references S5
   1.3 Model feature selection S10
2. Random Forest Model Testing and Development S50
3. Table S10. Table of Contents for GitHub Repository S55
4. Model Operation Guide S55
5. Ablation study on the extra dataset S56
6. Compound library Cartesian Coordinates, total energies, and thermal energies S59
   6.1 Table S12. Calculated energies for dataset compounds. S59
   6.2 Cartesian coordinates of computed compounds S64
7. References for Supporting Information S141

1. Computational Methods

1.1 **Chart S1** Compounds used within the training/test library:

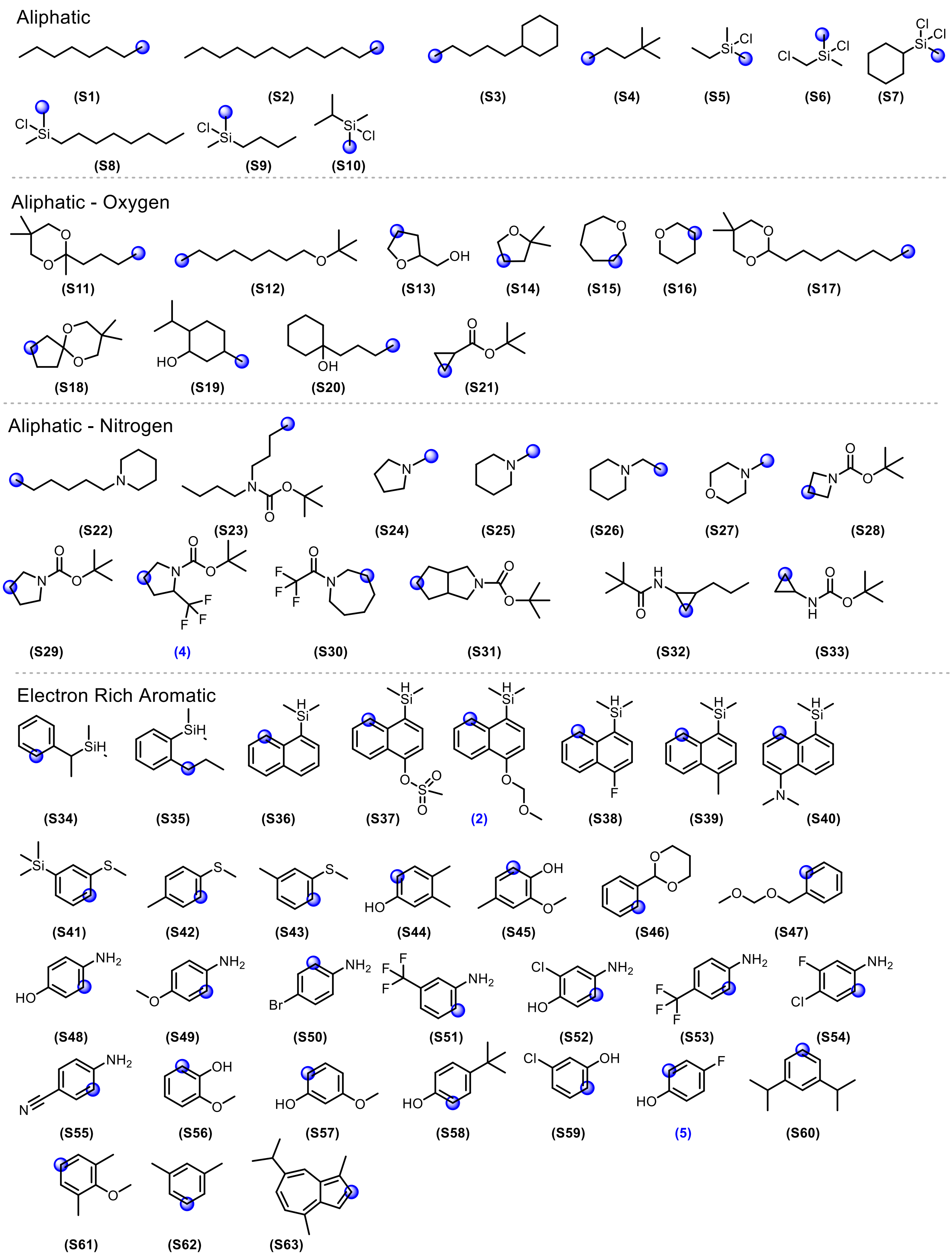

Electron Poor Aromatic

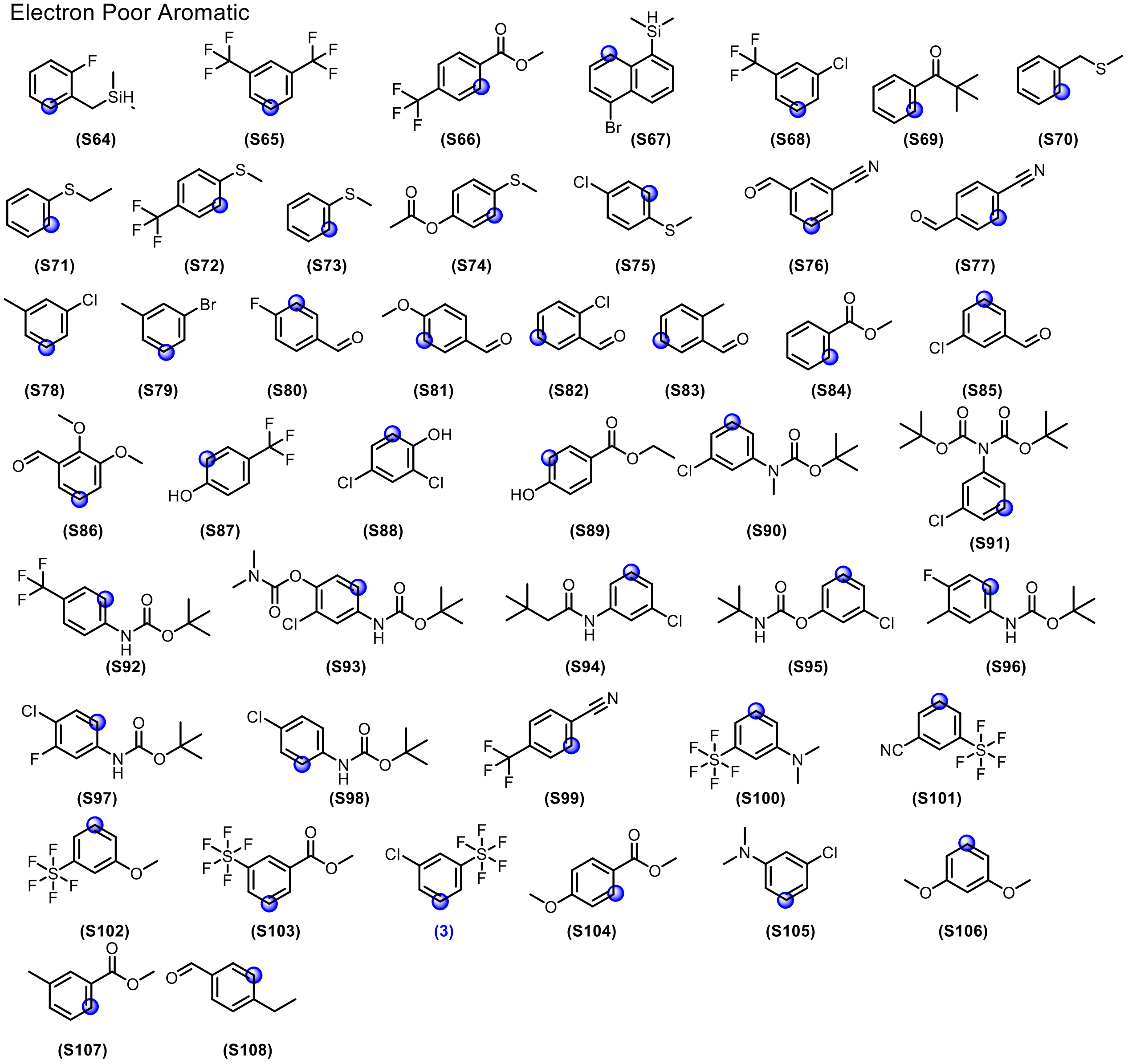

Aromatic

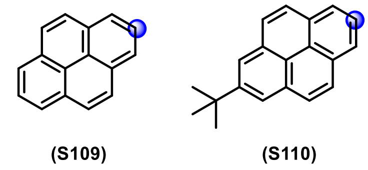

Thiophene

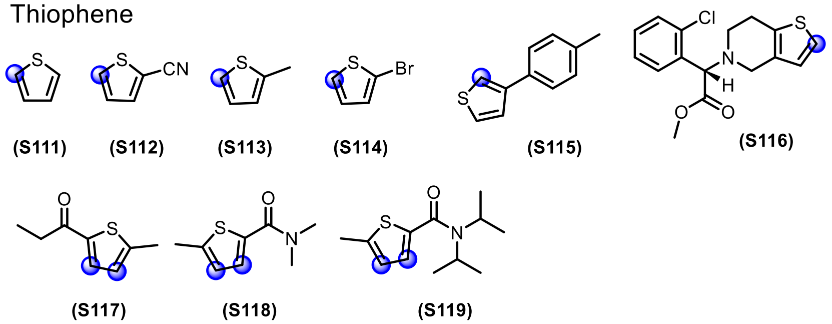

Benzofuran

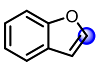

(S120)

Pyrrolopyridine

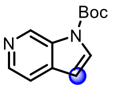

(S121)

Benzoimidazole

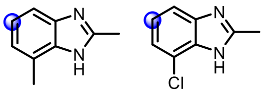

(S122) (S123)

Benzooxazole

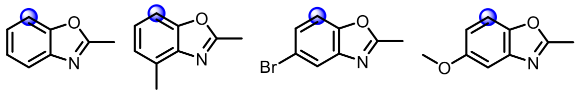

(S124) (S125) (S126) (S127)

Furan

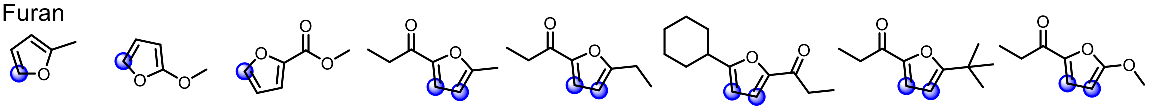

(S128) (S129) (S130) (S131) (S132) (S133) (S134) (S135)

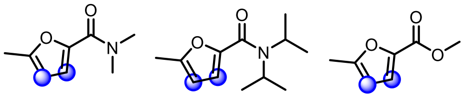

(S136) (S137) (S138)

Benzothiophene

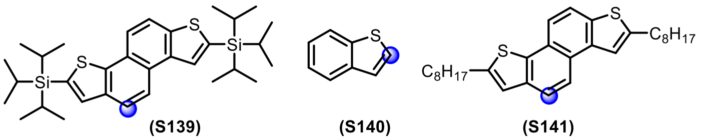

(S139) (S140) (S141)

Indole

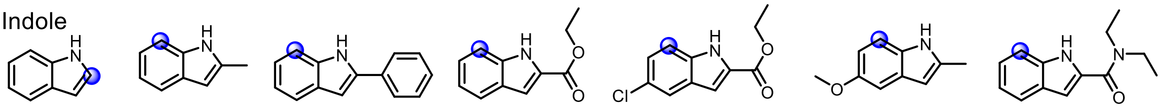

(S142) (S143) (S144) (1) (S145) (S146) (S147)

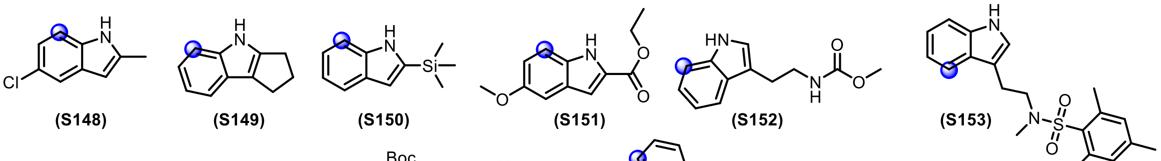

(S148) (S149) (S150) (S151) (S152) (S153)

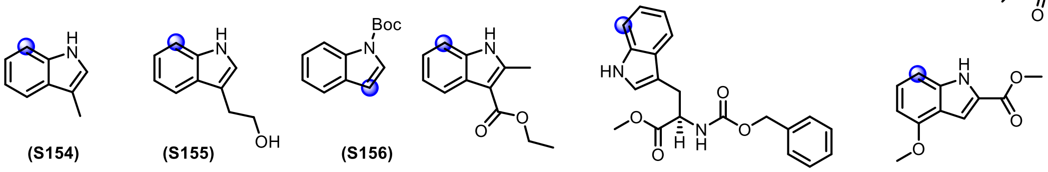

(S154) (S155) (S156) (S157) (S158) (S159)

Pyridine and pyrimidine

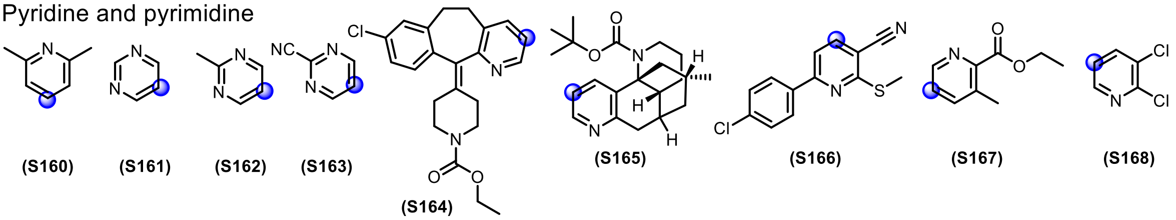

(S160) (S161) (S162) (S163) (S164) (S165) (S166) (S167) (S168)

Quinoline

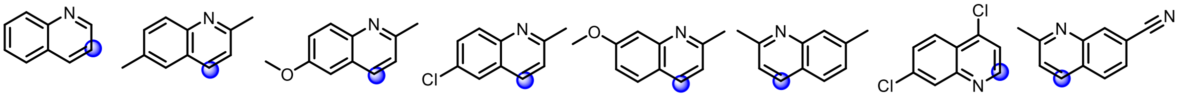

(S169) (S170) (S171) (S172) (S173) (S174) (S175) (S176)

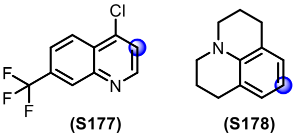

(S177) (S178)

Pyrrole

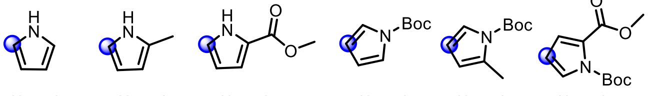

(S179) (S180) (S181) (S182) (S183) (S184)

1.2 **Table S1**. Compound experimental data and references

#	Catalyst/ ligand	Solvent	Temperature (°C)	Boronic ester	Ref. #
**1**	$[Ir(cod)(OMe)]_2$/ dtbpy	hexanes	60	HBpin	1
**2**	$[Ir(cod)(OMe)]_2$/ tmphen	THF	80	B_2pin_2	2
**3**	$[Ir(cod)(OMe)]_2$/ dtbpy	THF	80	B_2pin_2	3
**4**	$[Ir(cod)(OMe)]_2$/2-mphen	cyclooctane	100	B_2pin_2	4
**5**	$[Ir(cod)(OMe)]_2$/ dtbpy	cyclohexane	80	B_2pin_2	5
**6**	$[Ir(cod)(OMe)]_2$/ dtbpy and $[Ir(cod)(OMe)]_2$/ $AsPh_3$	octane	25 and 120	B_2pin_2	6
**7**	$[Ir(cod)(OMe)]_2$/ dtbpy and $[Ir(cod)(OMe)]_2$/ $AsPh_3$	octane	25 and 120	B_2pin_2	6
**8**	Cp*Rh(η^4-C_6Me_6)/ none	neat	150	B_2pin_2	7
**9**	Cp*Rh(η^4-C_6Me_6)/ none	neat	150	B_2pin_2	7
**10**	$[Ir(cod)(OMe)]_2$/ 1,10 phenanthroline	THF	100	B_2pin_2	8
**11**	$[Ir(cod)(OMe)]_2$/ dtbpy	THF	50	B_2pin_2	9
**12**	$[Ir(cod)(OMe)]_2$/ dtbpy	1,4-dioxane	100	B_2pin_2	10
**13**	$[Ir(cod)(OMe)]_2$/ tmphen	THF	80	HBpin	11
**14**	$[Ir(cod)(OMe)]_2$/2-mphen	cyclooctane	100	B_2pin_2	4
**15**	$[Ir(cod)(OMe)]_2$/ dtbpy	THF	90	B_2pin_2	12
**16**	$[Ir(cod)(OMe)]_2$/ dtbpy	Me-THF	80	B_2pin_2	13
**17**	$[Ir(cod)(OMe)]_2$/ tmphen	THF	80	B_2pin_2	14
**18**	$[Ir(cod)(OMe)]_2$/ dtbpy	neat	25	HBpin	15
**19**	$[Ir(cod)(OMe)]_2$/ dtbpy	MTBE	100	B_2pin_2	16
**20**	$[Ir(cod)(OMe)]_2$/ tmphen	THF	60	B_2pin_2	17
**21**	$[Ir(cod)(OMe)]_2$/ 5-phenyl-2,2'-bipyridine	arene	60	B_2pin_2	18
**22**	$[Ir(cod)(OMe)]_2$/ tmphen	THF	23	HBpin	19
**23**	$[Ir(cod)(OMe)]_2$/ dtbpy	THF/hexane	80	B_2pin_2	20
**24**	$[Ir(cod)(OMe)]_2$/ Silica – SMAP	hexane	70	B_2pin_2	21
**S1**	$[Cp*RuCl_2]_2$/none	octane	150	B_2pin_2	22
**S2**	$[Ir(cod)(OMe)]_2$/2-mphen	cyclooctane	100	B_2pin_2	4
**S3**	$[Ir(cod)(OMe)]_2$/2-mphen	cyclooctane	100	B_2pin_2	4
**S4**	$[Cp*RuCl_2]_2$/none	neat	150	B_2pin_2	22
**S5**	$[Ir(cod)(OMe)]_2$/ tmphen	cyclohexane	80	B_2pin_2	23
**S6**	$[Ir(cod)(OMe)]_2$/ tmphen	cyclohexane	80	B_2pin_2	23
**S7**	$[Ir(cod)(OMe)]_2$/ tmphen	cyclohexane	80	B_2pin_2	23
**S8**	$[Ir(cod)(OMe)]_2$/ tmphen	cyclohexane	80	B_2pin_2	23
**S9**	$[Ir(cod)(OMe)]_2$/ tmphen	cyclohexane	80	B_2pin_2	23
**S10**	$[Ir(cod)(OMe)]_2$/ tmphen	cyclohexane	80	B_2pin_2	23
**S11**	$[Ir(cod)(OMe)]_2$/2-mphen	cyclooctane	100	B_2pin_2	4
**S12**	$[Ir(cod)(OMe)]_2$/2-mphen	cyclooctane	100	B_2pin_2	4
**S13**	$[Ir(cod)(OMe)]_2$/2-mphen	cyclooctane	100	B_2pin_2	4
**S14**	(η^6-mes)Ir$(Bpin)_3$/ tmphen	neat	120	B_2pin_2	24
**S15**	(η^6-mes)Ir$(Bpin)_3$/ tmphen	neat	120	B_2pin_2	24
**S16**	(η^6-mes)Ir$(Bpin)_3$/ tmphen	neat	120	B_2pin_2	24
**S17**	$[Ir(cod)(OMe)]_2$/2-mphen	cyclooctane	100	B_2pin_2	4

**S18**	$[Ir(cod)(OMe)]_2$/2-mphen	cyclooctane	100	B_2pin_2	4
**S19**	$[Ir(cod)(OMe)]_2$/2-mphen	cyclooctane	100	B_2pin_2	4
**S20**	$[Ir(cod)(OMe)]_2$/2-mphen	cyclooctane	100	B_2pin_2	4
**S21**	$[Ir(cod)(OMe)]_2$/2-mphen	cyclooctane	100	B_2pin_2	4
**S22**	$[Ir(cod)(OMe)]_2$/2-mphen	cyclooctane	100	B_2pin_2	4
**S23**	$[Ir(cod)(OMe)]_2$/2-mphen	cyclooctane	100	B_2pin_2	4
**S24**	(η^6-mes)Ir(Bpin)$_3$/ tmphen	neat	120	B_2pin_2	25
**S25**	(η^6-mes)Ir(Bpin)$_3$/ tmphen	neat	120	B_2pin_2	25
**S26**	$[Cp^*RuCl_2]_2$/none	neat	150	B_2pin_2	22
**S27**	(η^6-mes)Ir(Bpin)$_3$/ tmphen	neat	120	B_2pin_2	25
**S28**	$[Ir(cod)(OMe)]_2$/2-mphen	cyclooctane	100	B_2pin_2	4
**S29**	$[Ir(cod)(OMe)]_2$/2-mphen	cyclooctane	100	B_2pin_2	4
**S30**	$[Ir(cod)(OMe)]_2$/2-mphen	cyclooctane	100	B_2pin_2	4
**S31**	$[Ir(cod)(OMe)]_2$/2-mphen	cyclooctane	100	B_2pin_2	4
**S32**	$[Ir(cod)(OMe)]_2$/ tmphen	cyclohexane	70	HBpin	26
**S33**	$[Ir(cod)(OMe)]_2$/ tmphen	cyclohexane	80	HBpin	26
**S34**	$[Ir(cod)(Cl]_2$/ dtbpy	THF	80	B_2pin_2	27
**S35**	$[Ir(cod)(OMe)]_2$/ dtbpy	THF	80	B_2pin_2	28
**S36**	$[Ir(cod)(OMe)]_2$/ tmphen	THF	80	B_2pin_2	2
**S37**	$[Ir(cod)(OMe)]_2$/ tmphen	THF	80	B_2pin_2	2
**S38**	$[Ir(cod)(OMe)]_2$/ tmphen	THF	80	B_2pin_2	2
**S39**	$[Ir(cod)(OMe)]_2$/ tmphen	THF	80	B_2pin_2	2
**S40**	$[Ir(cod)(OMe)]_2$/ tmphen	THF	80	B_2pin_2	2
**S41**	$[Ir(cod)(OMe)]_2$/ 5-phenyl-2,2'-bipyridine	arene	60	B_2pin_2	18
**S42**	$[Ir(cod)(OMe)]_2$/ 5-phenyl-2,2'-bipyridine	arene	60	B_2pin_2	18
**S43**	$[Ir(cod)(OMe)]_2$/ 5-phenyl-2,2'-bipyridine	arene	60	B_2pin_2	18
**S44**	$[Ir(cod)(OMe)]_2$/ dtbpy	cyclohexane	80	B_2pin_2	5
**S45**	$[Ir(cod)(OMe)]_2$/ dtbpy	cyclohexane	80	B_2pin_2	5
**S46**	$[Ir(cod)(OMe)]_2$/ Silica – SMAP	hexane	25	B_2pin_2	29
**S47**	$[Ir(cod)(OMe)]_2$/ Silica – SMAP	hexane	25	B_2pin_2	29
**S48**	$[Ir(cod)(OMe)]_2$/ tmphen	THF	80	HBpin	11
**S49**	$[Ir(cod)(OMe)]_2$/ tmphen	THF	80	HBpin	11
**S50**	$[Ir(cod)(OMe)]_2$/ tmphen	THF	80	HBpin	11
**S51**	$[Ir(cod)(OMe)]_2$/ tmphen	THF	80	HBpin	11
**S52**	$[Ir(cod)(OMe)]_2$/ tmphen	THF	80	HBpin	11
**S53**	$[Ir(cod)(OMe)]_2$/ tmphen	THF	80	HBpin	11
**S54**	$[Ir(cod)(OMe)]_2$/ tmphen	THF	80	HBpin	11
**S55**	$[Ir(cod)(OMe)]_2$/ tmphen	THF	80	HBpin	11
**S56**	$[Ir(cod)(OMe)]_2$/ dtbpy	cyclohexane	80	B_2pin_2	5
**S57**	$[Ir(cod)(OMe)]_2$/ dtbpy	cyclohexane	80	B_2pin_2	5
**S58**	$[Ir(cod)(OMe)]_2$/ dtbpy	cyclohexane	80	B_2pin_2	5
**S59**	$[Ir(cod)(OMe)]_2$/ dtbpy	cyclohexane	80	B_2pin_2	5
**S60**	$[Ir(cod)(OMe)]_2$/ dtbpy	CPME	70	B_2pin_2	30
**S61**	$[Ir(cod)(OMe)]_2$/ tmphen	THF	80	B_2pin_2	30
**S62**	Cp*Rh(η^4-C_6Me_6)/ none	neat	150	HBpin	31

**S63**	$[Ir(cod)(OMe)]_2$/ tmphen	THF	25	B_2pin_2	9
**S64**	$[Ir(cod)(Cl]_2$/ dtbpy	THF	80	B_2pin_2	27
**S65**	Cp*Rh(η^4-C_6Me_6)/ none	neat	150	HBpin	31
**S66**	$[Ir(cod)(OMe)]_2$/ Silica – SMAP	hexane	25	B_2pin_2	29
**S67**	$[Ir(cod)(OMe)]_2$/ tmphen	THF	80	B_2pin_2	2
**S68**	(Ind)Ir(cod)/ dppe	neat	150	HBpin	32
**S69**	$[Ir(cod)(OMe)]_2$/ Silica – SMAP	hexane	25	B_2pin_2	29
**S70**	$[Ir(cod)(OMe)]_2$/ 5-phenyl-2,2'-bipyridine	arene	60	B_2pin_2	18
**S71**	$[Ir(cod)(OMe)]_2$/ 5-phenyl-2,2'-bipyridine	arene	60	B_2pin_2	18
**S72**	$[Ir(cod)(OMe)]_2$/ 5-phenyl-2,2'-bipyridine	arene	60	B_2pin_2	18
**S73**	$[Ir(cod)(OMe)]_2$/ 5-phenyl-2,2'-bipyridine	arene	60	B_2pin_2	18
**S74**	$[Ir(cod)(OMe)]_2$/ 5-phenyl-2,2'-bipyridine	arene	60	B_2pin_2	18
**S75**	$[Ir(cod)(OMe)]_2$/ 5-phenyl-2,2'-bipyridine	arene	60	B_2pin_2	18
**S76**	$[Ir(cod)(OMe)]_2$/ tmphen	THF	90	HBpin	33
**S77**	$[Ir(cod)(OMe)]_2$/ tmphen	THF	90	HBpin	33
**S78**	(Ind)Ir(cod)/ dppe	neat	150	HBpin	32
**S79**	(Ind)Ir(cod)/ dmpe	neat	150	HBpin	32
**S80**	$[Ir(cod)(OMe)]_2$/ tmphen	THF	90	HBpin	33
**S81**	$[Ir(cod)(OMe)]_2$/ tmphen	THF	90	HBpin	33
**S82**	$[Ir(cod)(OMe)]_2$/ tmphen	THF	90	HBpin	33
**S83**	$[Ir(cod)(OMe)]_2$/ tmphen	THF	90	HBpin	33
**S84**	$[Ir(cod)(OMe)]_2$/ Silica – SMAP	hexane	25	B_2pin_2	29
**S85**	$[Ir(cod)(OMe)]_2$/ tmphen	THF	90	HBpin	33
**S86**	$[Ir(cod)(OMe)]_2$/ tmphen	THF	90	HBpin	33
**S87**	$[Ir(cod)(OMe)]_2$/ dtbpy	cyclohexane	80	B_2pin_2	5
**S88**	$[Ir(cod)(OMe)]_2$/ dtbpy	cyclohexane	80	B_2pin_2	5
**S89**	$[Ir(cod)(OMe)]_2$/ dtbpy	cyclohexane	80	B_2pin_2	5
**S90**	$[Ir(cod)(OMe)]_2$/ dtbpy	MTBE	50	B_2pin_2	34
**S91**	$[Ir(cod)(OMe)]_2$/ dtbpy	MTBE	50	B_2pin_2	34
**S92**	$[Ir(cod)(OMe)]_2$/ dtbpy	MTBE	50	B_2pin_2	34
**S93**	$[Ir(cod)(OMe)]_2$/ dtbpy	MTBE	50	B_2pin_2	34
**S94**	$[Ir(cod)(OMe)]_2$/ dtbpy	MTBE	50	B_2pin_2	34
**S95**	$[Ir(cod)(OMe)]_2$/ dtbpy	MTBE	50	B_2pin_2	34
**S96**	$[Ir(cod)(OMe)]_2$/ dtbpy	MTBE	50	B_2pin_2	34
**S97**	$[Ir(cod)(OMe)]_2$/ dtbpy	MTBE	50	B_2pin_2	34
**S98**	$[Ir(cod)(OMe)]_2$/ dtbpy	MTBE	50	B_2pin_2	34
**S99**	$[Ir(cod)(OMe)]_2$/ tmphen	THF	90	HBpin	33
**S100**	$[Ir(cod)(OMe)]_2$/ dtbpy	THF	80	B_2pin_2	3
**S101**	$[Ir(cod)(OMe)]_2$/ dtbpy	THF	80	B_2pin_2	3
**S102**	$[Ir(cod)(OMe)]_2$/ dtbpy	THF	80	B_2pin_2	3
**S103**	$[Ir(cod)(OMe)]_2$/ dtbpy	THF	80	B_2pin_2	3
**S104**	$[Ir(cod)(OMe)]_2$/ Silica – SMAP	hexane	25	B_2pin_2	29
**S105**	(Ind)Ir(cod)/ dmpe	neat	150	HBpin	32
**S106**	$[Ir(cod)(OMe)]_2$/ dtbpy	THF	80	B_2pin_2	35
**S107**	$[Ir(cod)(OMe)]_2$/ Silica – SMAP	hexane	25	B_2pin_2	29

**S108**	$[Ir(cod)(OMe)]_2$/ tmphen	THF	90	HBpin	33
**S109**	$[Ir(cod)(OMe)]_2$/ dtbpy	hexane	80	B_2pin_2	36
**S110**	$[Ir(cod)(OMe)]_2$/ dtbpy	hexane	80	B_2pin_2	36
**S111**	$[Ir(cod)(Cl]_2$/ dtbpy	octane	80	B_2pin_2	37
**S112**	$[Ir(cod)(OMe)]_2$/ dtbpy	hexane	25	B_2pin_2	38
**S113**	$[Ir(cod)(Cl]_2$/ dtbpy	octane	80	B_2pin_2	37
**S114**	$[Ir(cod)(OMe)]_2$/ dtbpy	hexane	25	B_2pin_2	38
**S115**	$[Ir(cod)(OMe)]_2$/ dtbpy	hexanes	25	HBpin	39
**S116**	$[Ir(cod)(OMe)]_2$/ tmphen	THF	25	B_2pin_2	9
**S117**	$[Ir(cod)(OMe)]_2$/ dtbpy and $[Ir(cod)(OMe)]_2$/ $AsPh_3$	octane	25 and 120	B_2pin_2	6
**S118**	$[Ir(cod)(OMe)]_2$/ dtbpy and $[Ir(cod)(OMe)]_2$/ $AsPh_3$	octane	25 and 120	B_2pin_2	6
**S119**	$[Ir(cod)(OMe)]_2$/ dtbpy and $[Ir(cod)(OMe)]_2$/ $AsPh_3$	octane	25 and 120	B_2pin_2	6
**S120**	$[Ir(cod)(Cl]_2$/ dtbpy	octane	80	B_2pin_2	37
**S121**	$[Ir(cod)(OMe)]_2$/ dtbpy	THF	55	HBpin	40
**S122**	$[Ir(cod)(OMe)]_2$/ tmphen	THF	25	B_2pin_2	41
**S123**	$[Ir(cod)(OMe)]_2$/ tmphen	THF	25	B_2pin_2	41
**S124**	$[Ir(cod)(OMe)]_2$/ tmphen	THF	25	B_2pin_2	41
**S125**	$[Ir(cod)(OMe)]_2$/ tmphen	THF	25	B_2pin_2	41
**S126**	$[Ir(cod)(OMe)]_2$/ tmphen	THF	25	B_2pin_2	41
**S127**	$[Ir(cod)(OMe)]_2$/ tmphen	THF	25	B_2pin_2	41
**S128**	$[Ir(cod)(OMe)]_2$/ dtbpy	hexane	25	B_2pin_2	38
**S129**	$[Ir(cod)(OMe)]_2$/ dtbpy	hexane	25	B_2pin_2	38
**S130**	$[Ir(cod)(OMe)]_2$/ dtbpy	hexane	25	B_2pin_2	38
**S131**	$[Ir(cod)(OMe)]_2$/ dtbpy and $[Ir(cod)(OMe)]_2$/ $AsPh_3$	octane	25 and 120	B_2pin_2	6
**S132**	$[Ir(cod)(OMe)]_2$/ dtbpy and $[Ir(cod)(OMe)]_2$/ $AsPh_3$	octane	25 and 120	B_2pin_2	6
**S133**	$[Ir(cod)(OMe)]_2$/ dtbpy and $[Ir(cod)(OMe)]_2$/ $AsPh_3$	octane	25 and 120	B_2pin_2	6
**S134**	$[Ir(cod)(OMe)]_2$/ dtbpy and $[Ir(cod)(OMe)]_2$/ $AsPh_3$	octane	25 and 120	B_2pin_2	6
**S135**	$[Ir(cod)(OMe)]_2$/ dtbpy and $[Ir(cod)(OMe)]_2$/ $AsPh_3$	octane	25 and 120	B_2pin_2	6
**S136**	$[Ir(cod)(OMe)]_2$/ dtbpy and $[Ir(cod)(OMe)]_2$/ $AsPh_3$	octane	25 and 120	B_2pin_2	6
**S137**	$[Ir(cod)(OMe)]_2$/ dtbpy and $[Ir(cod)(OMe)]_2$/ $AsPh_3$	octane	25 and 120	B_2pin_2	6
**S138**	$[Ir(cod)(OMe)]_2$/ dtbpy and $[Ir(cod)(OMe)]_2$/ $AsPh_3$	octane	25 and 120	B_2pin_2	6
**S139**	$[Ir(cod)(OMe)]_2$/ dtbpy	cyclohexane	80	B_2pin_2	42
**S140**	$[Ir(cod)(Cl]_2$/ dtbpy	octane	80	B_2pin_2	37
**S141**	$[Ir(cod)(OMe)]_2$/ dtbpy	cyclohexane	80	B_2pin_2	42
**S142**	$[Ir(cod)(Cl]_2$/ dtbpy	octane	80	B_2pin_2	37
**S143**	$[Ir(cod)(OMe)]_2$/ dtbpy	hexanes	60	HBpin	1

**S144**	$[Ir(cod)(OMe)]_2$/ dtbpy	hexanes	60	HBpin	1
**S145**	$[Ir(cod)(OMe)]_2$/ dtbpy	hexanes	60	HBpin	1
**S146**	$[Ir(cod)(OMe)]_2$/ dtbpy	hexanes	60	HBpin	1
**S147**	$[Ir(cod)(OMe)]_2$/ dtbpy	hexanes	60	HBpin	1
**S148**	$[Ir(cod)(OMe)]_2$/ dtbpy	hexanes	60	HBpin	1
**S149**	$[Ir(cod)(OMe)]_2$/ dtbpy	hexanes	60	HBpin	1
**S150**	$[Ir(cod)(OMe)]_2$/ dtbpy	hexanes	60	HBpin	1
**S151**	$[Ir(cod)(OMe)]_2$/ dtbpy	hexanes	60	HBpin	1
**S152**	$[Ir(cod)(OMe)]_2$/ dtbpy	THF	60	HBpin	43
**S153**	$[Ir(cod)(OMe)]_2$/ dtbpy	THF	60	HBpin	43
**S154**	$[Ir(cod)(OMe)]_2$/ dtbpy	THF	60	HBpin	43
**S155**	$[Ir(cod)(OMe)]_2$/ dtbpy	THF	60	HBpin	43
**S156**	$[Ir(cod)(OMe)]_2$/ dtbpy	THF	55	HBpin	40
**S157**	$[Ir(cod)(OMe)]_2$/ dtbpy	hexanes	60	HBpin	1
**S158**	$[Ir(cod)(OMe)]_2$/ dtbpy	THF	60	HBpin	43
**S159**	$[Ir(cod)(OMe)]_2$/ dtbpy	hexanes	60	HBpin	1
**S160**	$Cp^*Rh(\eta^4-C_6Me_6)$/ none	neat	150	HBpin	31
**S161**	$[Ir(cod)(OMe)]_2$/ tmphen	THF	25	B_2pin_2	41
**S162**	$[Ir(cod)(OMe)]_2$/ tmphen	THF	25	B_2pin_2	41
**S163**	$[Ir(cod)(OMe)]_2$/ tmphen	THF	25	B_2pin_2	41
**S164**	$[Ir(cod)(OMe)]_2$/ tmphen	THF	25	B_2pin_2	9
**S165**	$[Ir(cod)(OMe)]_2$/ dtbpy	THF	80	B_2pin_2	44
**S166**	$[Ir(cod)(OMe)]_2$/ tmphen	THF	80	B_2pin_2	14
**S167**	$[Ir(cod)(OMe)]_2$/ dtbpy	THF	80	B_2pin_2	45
**S168**	$[Ir(cod)(OMe)]_2$/ dtbpy	MTBE	25	B_2pin_2	46
**S169**	$[Ir(cod)(Cl]_2$/ dtbpy	octane	80	B_2pin_2	37
**S170**	$[Ir(cod)(OMe)]_2$/ dtbpy	MTBE	100	B_2pin_2	16
**S171**	$[Ir(cod)(OMe)]_2$/ dtbpy	MTBE	100	B_2pin_2	16
**S172**	$[Ir(cod)(OMe)]_2$/ dtbpy	MTBE	100	B_2pin_2	16
**S173**	$[Ir(cod)(OMe)]_2$/ dtbpy	MTBE	100	B_2pin_2	16
**S174**	$[Ir(cod)(OMe)]_2$/ dtbpy	MTBE	100	B_2pin_2	16
**S175**	$[Ir(cod)(OMe)]_2$/ dtbpy	MTBE	100	B_2pin_2	16
**S176**	$[Ir(cod)(OMe)]_2$/ dtbpy	MTBE	100	B_2pin_2	16
**S177**	$[Ir(cod)(OMe)]_2$/ dtbpy	MTBE	100	B_2pin_2	16
**S178**	$(\eta^6$-mes)$Ir(Bpin)_3$/ dtbpy	THF	100	B_2pin_2	47
**S179**	$[Ir(cod)(Cl]_2$/ dtbpy	octane	80	B_2pin_2	37
**S180**	$[Ir(cod)(OMe)]_2$/ dtbpy	hexane	25	B_2pin_2	38
**S181**	$[Ir(cod)(OMe)]_2$/ dtbpy	hexane	25	B_2pin_2	38
**S182**	$[Ir(cod)(OMe)]_2$/ dtbpy	THF	55	HBpin	40
**S183**	$[Ir(cod)(OMe)]_2$/ dtbpy	THF	55	HBpin	40
**S184**	$[Ir(cod)(OMe)]_2$/ dtbpy	THF	55	HBpin	40

1.3 Model feature selection

Table S2. Experimental conditions from literature reactions

**Catalysts**	**#**	**Added Ligands**	**#**	**Solvents**	**#**	**Boronic Ester**	**#**
$[Cp^{*}RuCl_2]_2$	1	No added ligand	1	THF	1	HBpin	1
$[Cp^{*}RuCl]_4$	2	dtbpy	2	octane	2	B_2pin_2	2
$Cp^{*}Rh(\eta^4\text{-}C_6Me_6)$	3	dppe	3	neat	3		
(Ind)Ir(cod)	4	Silica – SMAP	4	cyclohexane	4		
$[Ir(cod)(Cl]_2$	5	2-mphen	5	hexane	5		
$[Ir(cod)(OMe)]_2$	6	tmphen	6	hexanes	6		
$(\eta^6\text{-mes})Ir(Bpin)_3$	7	5-phenyl-2,2'-bipyridine	7	toluene	7		
		$AsPh_3$	8	cyclooctane	8		
		1,10 phenanthroline	9	arene	9		
		dmpe	11	MTBE	10		
				1,4-dioxane	11		
				Me-THF	12		
				CPME	13		

Table S3. Each active catalyst was parameterized using the Morfeus library developed by the Sigman group.[48] Catalyst coordinates derived from Zhong and Sakaki and optimized at the b3lyp/lanl2dz level of theory are given in our GitHub repository.[49–51]

**Descriptor**
buried volume
pyramidalization according to Garvish
pyramidalization according to Agranat and Radhakrishnan
SASA area
SASA volume
sterimol L
sterimol B_1
sterimol B_5
buried sterimol L
buried sterimol B_1
buried sterimol B_5

Table S4. Experimental parameters taken from literature reactions.

**Feature Index**	**If on atom (Y/N)**	**Feature Name**	**Description**
**0**	N	Boronic Ester	Either Bpin or B_2pin_2
**1**	N	Active Catalyst	Active catalyst-ligand combination
**2**	N	Catalyst	Reaction catalyst used
**3**	N	Ligand	Reaction ligand used
**4**	N	Solvent	Solvent used in reaction
**5**	N	Temperature	Temperature used in reaction

Table S5. Gaussian 09 point charges were computed at the B3LYP/6-311+g** level of theory.[52–55]

**Feature Index**	**If on atom**	**Feature Name**	**Description**

	(Y/N)		
**6**	Y	Hirshfeld Heavy Atom Charge	Charge distribution based upon bonded-atom fragment deformation densities minus free atom densities[56], summed into heavy atoms
**7**	Y	Hirshfeld Carbon Charge	Charge distribution based upon bonded-atom fragment deformation densities minus free atom densities for carbon atoms
**8**	Y	Hirshfeld Hydrogen Charge	Charge distribution based upon bonded-atom fragment deformation densities minus free atom densities for hydrogen atoms
**9**	Y	CM5 Charge	Class IV (defined through parametrization) partial atomic charges mapped from Hirshfeld population analysis[57]
**10**	Y	ESP Heavy Atom Charge	Charges from electrostatic potentials using a grid-based method, summed into heavy atoms[58]
**11**	Y	ESP Carbon Charge	Charges from electrostatic potentials using a grid-based method for carbon atoms
**12**	Y	ESP Hydrogen Charge	Charges from electrostatic potentials using a grid-based method for hydrogen
**13**	Y	NPA Carbon Charge	Charges based on "natural atomic orbitals" for an arbitrary basis set for carbon atoms[59]
**14**	Y	NPA Hydrogen Charge	Charges based on "natural atomic orbitals" for an arbitrary basis set for hydrogen atoms
**15**	Y	MBS Heavy Atom Charge	Charges derived from Mulliken populations with a minimal basis set, summed into heavy atoms
**16**	Y	MBS Carbon Charge	Charges derived from Mulliken populations with a minimal basis set for carbon atoms
**17**	Y	MBS Hydrogen Charge	Charges derived from Mulliken populations with a minimal basis set for carbon atoms
**18**	Y	Mulliken Heavy Charge	Charges based upon LCAO-MO molecular wave functions, summed into heavy atoms[60]
**19**	Y	Mulliken Carbon Charge	Charges based upon LCAO-MO molecular wave functions for carbon atoms
**20**	Y	Mulliken Hydrogen Charge	Charges based upon LCAO-MO molecular wave functions for hydrogen atoms

Table S6. JChem for Excel features.[61]

Feature Index	If on atom	Feature Name	Description

	(Y/N)		
**21**	N	Aliphatic Atom Count	Calculates the number of aliphatic atoms in a molecule
**22**	N	Aliphatic Bond Count	Calculates the number of aliphatic bonds in the molecule
**23**	N	Aliphatic Ring Count	Calculates the number of aliphatic rings in the molecule
**24**	N	Aromatic Atom Count	Calculates the number of aromatic atoms in the molecule
**25**	N	Aromatic Bond Count	Calculates the number of aromatic bonds in the molecule
**26**	Y	Steric Effect Index	Calculates the steric effect index of an atom
**27**	Y	Atomic_Polarizability	Calculates the atomic polarizability
**28**	N	Balaban Index	Calculates the Balaban index
**29**	N	Chain Atom Count	Calculates the number of chain atoms in the molecule
**30**	Y	Distance Degree	Calculates the distance degree of an atom
**31**	N	Dreiding Energy	Returns the Dreiding forcefield energy of the input molecule (for the input if it is a 3D molecule or a generated lowest energy conformer)
**32**	Y	Eccentricity	Calculates the eccentricity of an atom
**33**	N	Harary Index	Calculates the Harary index for the graph of input the molecule
**34**	N	Hydrogen Acceptor Count	Calculates the hydrogen acceptor atom count in molecule
**35**	N	Hydrogen Acceptor Site Count	Calculates hydrogen bond acceptor multiplicity in molecule
**36**	N	Heteroatom Aromatic Ring Count	Calculates the number of aromatic heterocyclic rings (the number of rings in the smallest set of smallest aromatic heterocyclic rings, SSSR) in the input molecule
**37**	N	Hydrogen Donor Count	Calculates hydrogen bond donor atom count in molecule
**38**	N	Hyper Wiener Index	Calculates a variant of the average topological atom distance (half of the sum of all atom distances) in the molecule
**39**	N	Largest Ring Size	Calculates the number of atoms in the largest ring of the input molecule
**40**	N	Max Projection Area	Calculates the maximal projection area of the molecule
**41**	N	Max Projection Radius	Calculates the maximal projection radius of the molecule
**42**	N	MaxZ	Returns the maximum z coordinate of the bounding box
**43**	N	Molecular Polarizability	Calculates the molecular polarizability of the molecule
**44**	N	Platt Index	Calculates the Platt index of the molecule
**45**	N	Refractivity	Calculates the molar refractivity of the molecule
**46**	N	Ring Atom Count	Calculates the number of ring atoms in the molecule
**47**	N	Rot Bond Count	Calculates the number of ring bonds in the molecule
**48**	Y	Sigma Orbital Electronegativity	Calculates atomic sigma orbital electronegativity
**49**	N	Wiener Index	Calculates the Wiener index of the molecule

Table S7. Mordred features.[62]

Feature Index	If on atom	Feature Name	Description

	(Y/N)		
**50**	N	ABC	atom-bond connectivity index
**51**	N	ABCGG	Graovac-Ghorbani atom-bond connectivity index
**52**	N	nAcid	acidic group count
**53**	N	nBase	basic group count
**54**	N	SpAbs_A	SpAbs of adjacency matrix
**55**	N	SpMax_A	SpMax of adjacency matrix
**56**	N	SpDiam_A	SpDiam of adjacency matrix
**57**	N	SpAD_A	SpAD of adjacency matrix
**58**	N	SpMAD_A	SpMAD of adjacency matrix
**59**	N	LogEE_A	LogEE of adjacency matrix
**60**	N	SM1_A	SM1 of adjacency matrix
**61**	N	VE1_A	VE1 of adjacency matrix
**62**	N	VE2_A	VE2 of adjacency matrix
**63**	N	VE3_A	VE3 of adjacency matrix
**64**	N	VR1_A	VR1 of adjacency matrix
**65**	N	VR2_A	VR2 of adjacency matrix
**66**	N	VR3_A	VR3 of adjacency matrix
**67**	N	nAromAtom	aromatic atoms count
**68**	N	nAromBond	aromatic bonds count
**69**	N	nAtom	number of all atoms
**70**	N	nHeavyAtom	number of heavy atoms
**71**	N	nSpiro	number of spiro atoms
**72**	N	nBridgehead	number of bridgehead atoms
**73**	N	nH	number of H atoms
**74**	N	nB	number of B atoms
**75**	N	nC	number of C atoms
**76**	N	nN	number of N atoms
**77**	N	nO	number of O atoms
**78**	N	nS	number of S atoms
**79**	N	nP	number of P atoms
**80**	N	nF	number of F atoms
**81**	N	nCl	number of Cl atoms
**82**	N	nBr	number of Br atoms
**83**	N	nI	number of I atoms
**84**	N	nX	number of halogen atoms
**85**	N	ATS0dv	moreau-broto autocorrelation of lag 0 weighted by valence electrons
**86**	N	ATS1dv	moreau-broto autocorrelation of lag 1 weighted by valence electrons
**87**	N	ATS2dv	moreau-broto autocorrelation of lag 2 weighted by valence electrons
**88**	N	ATS3dv	moreau-broto autocorrelation of lag 3 weighted by valence electrons
**89**	N	ATS4dv	moreau-broto autocorrelation of lag 4 weighted by valence electrons
**90**	N	ATS5dv	moreau-broto autocorrelation of lag 5 weighted by valence electrons
**91**	N	ATS6dv	moreau-broto autocorrelation of lag 6 weighted by valence electrons
**92**	N	ATS7dv	moreau-broto autocorrelation of lag 7 weighted by valence electrons
**93**	N	ATS8dv	moreau-broto autocorrelation of lag 8 weighted by valence electrons

**94**	N	ATS0d	moreau-broto autocorrelation of lag 0 weighted by sigma electrons
**95**	N	ATS1d	moreau-broto autocorrelation of lag 1 weighted by sigma electrons
**96**	N	ATS2d	moreau-broto autocorrelation of lag 2 weighted by sigma electrons
**97**	N	ATS3d	moreau-broto autocorrelation of lag 3 weighted by sigma electrons
**98**	N	ATS4d	moreau-broto autocorrelation of lag 4 weighted by sigma electrons
**99**	N	ATS5d	moreau-broto autocorrelation of lag 5 weighted by sigma electrons
**100**	N	ATS6d	moreau-broto autocorrelation of lag 6 weighted by sigma electrons
**101**	N	ATS7d	moreau-broto autocorrelation of lag 7 weighted by sigma electrons
**102**	N	ATS8d	moreau-broto autocorrelation of lag 8 weighted by sigma electrons
**103**	N	ATS0s	moreau-broto autocorrelation of lag 0 weighted by intrinsic state
**104**	N	ATS1s	moreau-broto autocorrelation of lag 1 weighted by intrinsic state
**105**	N	ATS2s	moreau-broto autocorrelation of lag 2 weighted by intrinsic state
**106**	N	ATS3s	moreau-broto autocorrelation of lag 3 weighted by intrinsic state
**107**	N	ATS4s	moreau-broto autocorrelation of lag 4 weighted by intrinsic state
**108**	N	ATS5s	moreau-broto autocorrelation of lag 5 weighted by intrinsic state
**109**	N	ATS6s	moreau-broto autocorrelation of lag 6 weighted by intrinsic state
**110**	N	ATS7s	moreau-broto autocorrelation of lag 7 weighted by intrinsic state
**111**	N	ATS8s	moreau-broto autocorrelation of lag 8 weighted by intrinsic state
**112**	N	ATS0Z	moreau-broto autocorrelation of lag 0 weighted by atomic number
**113**	N	ATS1Z	moreau-broto autocorrelation of lag 1 weighted by atomic number
**114**	N	ATS2Z	moreau-broto autocorrelation of lag 2 weighted by atomic number
**115**	N	ATS3Z	moreau-broto autocorrelation of lag 3 weighted by atomic number
**116**	N	ATS4Z	moreau-broto autocorrelation of lag 4 weighted by atomic number
**117**	N	ATS5Z	moreau-broto autocorrelation of lag 5 weighted by atomic number
**118**	N	ATS6Z	moreau-broto autocorrelation of lag 6 weighted by atomic number
**119**	N	ATS7Z	moreau-broto autocorrelation of lag 7 weighted by atomic number
**120**	N	ATS8Z	moreau-broto autocorrelation of lag 8 weighted by atomic number
**121**	N	ATS0m	moreau-broto autocorrelation of lag 0 weighted by mass
**122**	N	ATS1m	moreau-broto autocorrelation of lag 1 weighted by mass
**123**	N	ATS2m	moreau-broto autocorrelation of lag 2 weighted by mass
**124**	N	ATS3m	moreau-broto autocorrelation of lag 3 weighted by mass
**125**	N	ATS4m	moreau-broto autocorrelation of lag 4 weighted by mass
**126**	N	ATS5m	moreau-broto autocorrelation of lag 5 weighted by mass
**127**	N	ATS6m	moreau-broto autocorrelation of lag 6 weighted by mass
**128**	N	ATS7m	moreau-broto autocorrelation of lag 7 weighted by mass
**129**	N	ATS8m	moreau-broto autocorrelation of lag 8 weighted by mass
**130**	N	ATS0v	moreau-broto autocorrelation of lag 0 weighted by vdw volume
**131**	N	ATS1v	moreau-broto autocorrelation of lag 1 weighted by vdw volume
**132**	N	ATS2v	moreau-broto autocorrelation of lag 2 weighted by vdw volume
**133**	N	ATS3v	moreau-broto autocorrelation of lag 3 weighted by vdw volume
**134**	N	ATS4v	moreau-broto autocorrelation of lag 4 weighted by vdw volume
**135**	N	ATS5v	moreau-broto autocorrelation of lag 5 weighted by vdw volume
**136**	N	ATS6v	moreau-broto autocorrelation of lag 6 weighted by vdw volume
**137**	N	ATS7v	moreau-broto autocorrelation of lag 7 weighted by vdw volume
**138**	N	ATS8v	moreau-broto autocorrelation of lag 8 weighted by vdw volume

**139**	N	ATS0se	moreau-broto autocorrelation of lag 0 weighted by sanderson EN
**140**	N	ATS1se	moreau-broto autocorrelation of lag 1 weighted by sanderson EN
**141**	N	ATS2se	moreau-broto autocorrelation of lag 2 weighted by sanderson EN
**142**	N	ATS3se	moreau-broto autocorrelation of lag 3 weighted by sanderson EN
**143**	N	ATS4se	moreau-broto autocorrelation of lag 4 weighted by sanderson EN
**144**	N	ATS5se	moreau-broto autocorrelation of lag 5 weighted by sanderson EN
**145**	N	ATS6se	moreau-broto autocorrelation of lag 6 weighted by sanderson EN
**146**	N	ATS7se	moreau-broto autocorrelation of lag 7 weighted by sanderson EN
**147**	N	ATS8se	moreau-broto autocorrelation of lag 8 weighted by sanderson EN
**148**	N	ATS0pe	moreau-broto autocorrelation of lag 0 weighted by pauling EN
**149**	N	ATS1pe	moreau-broto autocorrelation of lag 1 weighted by pauling EN
**150**	N	ATS2pe	moreau-broto autocorrelation of lag 2 weighted by pauling EN
**151**	N	ATS3pe	moreau-broto autocorrelation of lag 3 weighted by pauling EN
**152**	N	ATS4pe	moreau-broto autocorrelation of lag 4 weighted by pauling EN
**153**	N	ATS5pe	moreau-broto autocorrelation of lag 5 weighted by pauling EN
**154**	N	ATS6pe	moreau-broto autocorrelation of lag 6 weighted by pauling EN
**155**	N	ATS7pe	moreau-broto autocorrelation of lag 7 weighted by pauling EN
**156**	N	ATS8pe	moreau-broto autocorrelation of lag 8 weighted by pauling EN
**157**	N	ATS0are	moreau-broto autocorrelation of lag 0 weighted by allred-rocow EN
**158**	N	ATS1are	moreau-broto autocorrelation of lag 1 weighted by allred-rocow EN
**159**	N	ATS2are	moreau-broto autocorrelation of lag 2 weighted by allred-rocow EN
**160**	N	ATS3are	moreau-broto autocorrelation of lag 3 weighted by allred-rocow EN
**161**	N	ATS4are	moreau-broto autocorrelation of lag 4 weighted by allred-rocow EN
**162**	N	ATS5are	moreau-broto autocorrelation of lag 5 weighted by allred-rocow EN
**163**	N	ATS6are	moreau-broto autocorrelation of lag 6 weighted by allred-rocow EN
**164**	N	ATS7are	moreau-broto autocorrelation of lag 7 weighted by allred-rocow EN
**165**	N	ATS8are	moreau-broto autocorrelation of lag 8 weighted by allred-rocow EN
**166**	N	ATS0p	moreau-broto autocorrelation of lag 0 weighted by polarizability
**167**	N	ATS1p	moreau-broto autocorrelation of lag 1 weighted by polarizability
**168**	N	ATS2p	moreau-broto autocorrelation of lag 2 weighted by polarizability
**169**	N	ATS3p	moreau-broto autocorrelation of lag 3 weighted by polarizability
**170**	N	ATS4p	moreau-broto autocorrelation of lag 4 weighted by polarizability
**171**	N	ATS5p	moreau-broto autocorrelation of lag 5 weighted by polarizability
**172**	N	ATS6p	moreau-broto autocorrelation of lag 6 weighted by polarizability
**173**	N	ATS7p	moreau-broto autocorrelation of lag 7 weighted by polarizability
**174**	N	ATS8p	moreau-broto autocorrelation of lag 8 weighted by polarizability
**175**	N	ATS0i	moreau-broto autocorrelation of lag 0 weighted by ionization potential
**176**	N	ATS1i	moreau-broto autocorrelation of lag 1 weighted by ionization potential
**177**	N	ATS2i	moreau-broto autocorrelation of lag 2 weighted by ionization potential
**178**	N	ATS3i	moreau-broto autocorrelation of lag 3 weighted by ionization potential
**179**	N	ATS4i	moreau-broto autocorrelation of lag 4 weighted by ionization potential
**180**	N	ATS5i	moreau-broto autocorrelation of lag 5 weighted by ionization potential
**181**	N	ATS6i	moreau-broto autocorrelation of lag 6 weighted by ionization potential
**182**	N	ATS7i	moreau-broto autocorrelation of lag 7 weighted by ionization potential
**183**	N	ATS8i	moreau-broto autocorrelation of lag 8 weighted by ionization potential

**184**	N	AATS0dv	averaged moreau-broto autocorrelation of lag 0 weighted by valence electrons
**185**	N	AATS1dv	averaged moreau-broto autocorrelation of lag 1 weighted by valence electrons
**186**	N	AATS2dv	averaged moreau-broto autocorrelation of lag 2 weighted by valence electrons
**187**	N	AATS3dv	averaged moreau-broto autocorrelation of lag 3 weighted by valence electrons
**188**	N	AATS4dv	averaged moreau-broto autocorrelation of lag 4 weighted by valence electrons
**189**	N	AATS5dv	averaged moreau-broto autocorrelation of lag 5 weighted by valence electrons
**190**	N	AATS6dv	averaged moreau-broto autocorrelation of lag 6 weighted by valence electrons
**191**	N	AATS7dv	averaged moreau-broto autocorrelation of lag 7 weighted by valence electrons
**192**	N	AATS8dv	averaged moreau-broto autocorrelation of lag 8 weighted by valence electrons
**193**	N	AATS0d	averaged moreau-broto autocorrelation of lag 0 weighted by sigma electrons
**194**	N	AATS1d	averaged moreau-broto autocorrelation of lag 1 weighted by sigma electrons
**195**	N	AATS2d	averaged moreau-broto autocorrelation of lag 2 weighted by sigma electrons
**196**	N	AATS3d	averaged moreau-broto autocorrelation of lag 3 weighted by sigma electrons
**197**	N	AATS4d	averaged moreau-broto autocorrelation of lag 4 weighted by sigma electrons
**198**	N	AATS5d	averaged moreau-broto autocorrelation of lag 5 weighted by sigma electrons
**199**	N	AATS6d	averaged moreau-broto autocorrelation of lag 6 weighted by sigma electrons
**200**	N	AATS7d	averaged moreau-broto autocorrelation of lag 7 weighted by sigma electrons
**201**	N	AATS8d	averaged moreau-broto autocorrelation of lag 8 weighted by sigma electrons
**202**	N	AATS0s	averaged moreau-broto autocorrelation of lag 0 weighted by intrinsic state
**203**	N	AATS1s	averaged moreau-broto autocorrelation of lag 1 weighted by intrinsic state
**204**	N	AATS2s	averaged moreau-broto autocorrelation of lag 2 weighted by intrinsic state
**205**	N	AATS3s	averaged moreau-broto autocorrelation of lag 3 weighted by intrinsic state
**206**	N	AATS4s	averaged moreau-broto autocorrelation of lag 4 weighted by intrinsic state
**207**	N	AATS5s	averaged moreau-broto autocorrelation of lag 5 weighted by intrinsic state
**208**	N	AATS6s	averaged moreau-broto autocorrelation of lag 6 weighted by intrinsic state
**209**	N	AATS7s	averaged moreau-broto autocorrelation of lag 7 weighted by intrinsic state
**210**	N	AATS8s	averaged moreau-broto autocorrelation of lag 8 weighted by intrinsic state
**211**	N	AATS0Z	averaged moreau-broto autocorrelation of lag 0 weighted by atomic number
**212**	N	AATS1Z	averaged moreau-broto autocorrelation of lag 1 weighted by atomic number
**213**	N	AATS2Z	averaged moreau-broto autocorrelation of lag 2 weighted by atomic number
**214**	N	AATS3Z	averaged moreau-broto autocorrelation of lag 3 weighted by atomic number
**215**	N	AATS4Z	averaged moreau-broto autocorrelation of lag 4 weighted by atomic number
**216**	N	AATS5Z	averaged moreau-broto autocorrelation of lag 5 weighted by atomic number
**217**	N	AATS6Z	averaged moreau-broto autocorrelation of lag 6 weighted by atomic number
**218**	N	AATS7Z	averaged moreau-broto autocorrelation of lag 7 weighted by atomic number
**219**	N	AATS8Z	averaged moreau-broto autocorrelation of lag 8 weighted by atomic number
**220**	N	AATS0m	averaged moreau-broto autocorrelation of lag 0 weighted by mass
**221**	N	AATS1m	averaged moreau-broto autocorrelation of lag 1 weighted by mass
**222**	N	AATS2m	averaged moreau-broto autocorrelation of lag 2 weighted by mass
**223**	N	AATS3m	averaged moreau-broto autocorrelation of lag 3 weighted by mass
**224**	N	AATS4m	averaged moreau-broto autocorrelation of lag 4 weighted by mass
**225**	N	AATS5m	averaged moreau-broto autocorrelation of lag 5 weighted by mass
**226**	N	AATS6m	averaged moreau-broto autocorrelation of lag 6 weighted by mass
**227**	N	AATS7m	averaged moreau-broto autocorrelation of lag 7 weighted by mass
**228**	N	AATS8m	averaged moreau-broto autocorrelation of lag 8 weighted by mass

**229**	N	AATS0v	averaged moreau-broto autocorrelation of lag 0 weighted by vdw volume
**230**	N	AATS1v	averaged moreau-broto autocorrelation of lag 1 weighted by vdw volume
**231**	N	AATS2v	averaged moreau-broto autocorrelation of lag 2 weighted by vdw volume
**232**	N	AATS3v	averaged moreau-broto autocorrelation of lag 3 weighted by vdw volume
**233**	N	AATS4v	averaged moreau-broto autocorrelation of lag 4 weighted by vdw volume
**234**	N	AATS5v	averaged moreau-broto autocorrelation of lag 5 weighted by vdw volume
**235**	N	AATS6v	averaged moreau-broto autocorrelation of lag 6 weighted by vdw volume
**236**	N	AATS7v	averaged moreau-broto autocorrelation of lag 7 weighted by vdw volume
**237**	N	AATS8v	averaged moreau-broto autocorrelation of lag 8 weighted by vdw volume
**238**	N	AATS0se	averaged moreau-broto autocorrelation of lag 0 weighted by sanderson EN
**239**	N	AATS1se	averaged moreau-broto autocorrelation of lag 1 weighted by sanderson EN
**240**	N	AATS2se	averaged moreau-broto autocorrelation of lag 2 weighted by sanderson EN
**241**	N	AATS3se	averaged moreau-broto autocorrelation of lag 3 weighted by sanderson EN
**242**	N	AATS4se	averaged moreau-broto autocorrelation of lag 4 weighted by sanderson EN
**243**	N	AATS5se	averaged moreau-broto autocorrelation of lag 5 weighted by sanderson EN
**244**	N	AATS6se	averaged moreau-broto autocorrelation of lag 6 weighted by sanderson EN
**245**	N	AATS7se	averaged moreau-broto autocorrelation of lag 7 weighted by sanderson EN
**246**	N	AATS8se	averaged moreau-broto autocorrelation of lag 8 weighted by sanderson EN
**247**	N	AATS0pe	averaged moreau-broto autocorrelation of lag 0 weighted by pauling EN
**248**	N	AATS1pe	averaged moreau-broto autocorrelation of lag 1 weighted by pauling EN
**249**	N	AATS2pe	averaged moreau-broto autocorrelation of lag 2 weighted by pauling EN
**250**	N	AATS3pe	averaged moreau-broto autocorrelation of lag 3 weighted by pauling EN
**251**	N	AATS4pe	averaged moreau-broto autocorrelation of lag 4 weighted by pauling EN
**252**	N	AATS5pe	averaged moreau-broto autocorrelation of lag 5 weighted by pauling EN
**253**	N	AATS6pe	averaged moreau-broto autocorrelation of lag 6 weighted by pauling EN
**254**	N	AATS7pe	averaged moreau-broto autocorrelation of lag 7 weighted by pauling EN
**255**	N	AATS8pe	averaged moreau-broto autocorrelation of lag 8 weighted by pauling EN
**256**	N	AATS0are	averaged moreau-broto autocorrelation of lag 0 weighted by allred-rocow EN
**257**	N	AATS1are	averaged moreau-broto autocorrelation of lag 1 weighted by allred-rocow EN
**258**	N	AATS2are	averaged moreau-broto autocorrelation of lag 2 weighted by allred-rocow EN
**259**	N	AATS3are	averaged moreau-broto autocorrelation of lag 3 weighted by allred-rocow EN
**260**	N	AATS4are	averaged moreau-broto autocorrelation of lag 4 weighted by allred-rocow EN
**261**	N	AATS5are	averaged moreau-broto autocorrelation of lag 5 weighted by allred-rocow EN
**262**	N	AATS6are	averaged moreau-broto autocorrelation of lag 6 weighted by allred-rocow EN
**263**	N	AATS7are	averaged moreau-broto autocorrelation of lag 7 weighted by allred-rocow EN
**264**	N	AATS8are	averaged moreau-broto autocorrelation of lag 8 weighted by allred-rocow EN
**265**	N	AATS0p	averaged moreau-broto autocorrelation of lag 0 weighted by polarizability
**266**	N	AATS1p	averaged moreau-broto autocorrelation of lag 1 weighted by polarizability
**267**	N	AATS2p	averaged moreau-broto autocorrelation of lag 2 weighted by polarizability
**268**	N	AATS3p	averaged moreau-broto autocorrelation of lag 3 weighted by polarizability
**269**	N	AATS4p	averaged moreau-broto autocorrelation of lag 4 weighted by polarizability
**270**	N	AATS5p	averaged moreau-broto autocorrelation of lag 5 weighted by polarizability
**271**	N	AATS6p	averaged moreau-broto autocorrelation of lag 6 weighted by polarizability
**272**	N	AATS7p	averaged moreau-broto autocorrelation of lag 7 weighted by polarizability
**273**	N	AATS8p	averaged moreau-broto autocorrelation of lag 8 weighted by polarizability

**274**	N	AATS0i	averaged moreau-broto autocorrelation of lag 0 weighted by ionization potential
**275**	N	AATS1i	averaged moreau-broto autocorrelation of lag 1 weighted by ionization potential
**276**	N	AATS2i	averaged moreau-broto autocorrelation of lag 2 weighted by ionization potential
**277**	N	AATS3i	averaged moreau-broto autocorrelation of lag 3 weighted by ionization potential
**278**	N	AATS4i	averaged moreau-broto autocorrelation of lag 4 weighted by ionization potential
**279**	N	AATS5i	averaged moreau-broto autocorrelation of lag 5 weighted by ionization potential
**280**	N	AATS6i	averaged moreau-broto autocorrelation of lag 6 weighted by ionization potential
**281**	N	AATS7i	averaged moreau-broto autocorrelation of lag 7 weighted by ionization potential
**282**	N	AATS8i	averaged moreau-broto autocorrelation of lag 8 weighted by ionization potential
**283**	N	ATSC0c	centered moreau-broto autocorrelation of lag 0 weighted by gasteiger charge
**284**	N	ATSC1c	centered moreau-broto autocorrelation of lag 1 weighted by gasteiger charge
**285**	N	ATSC2c	centered moreau-broto autocorrelation of lag 2 weighted by gasteiger charge
**286**	N	ATSC3c	centered moreau-broto autocorrelation of lag 3 weighted by gasteiger charge
**287**	N	ATSC4c	centered moreau-broto autocorrelation of lag 4 weighted by gasteiger charge
**288**	N	ATSC5c	centered moreau-broto autocorrelation of lag 5 weighted by gasteiger charge
**289**	N	ATSC6c	centered moreau-broto autocorrelation of lag 6 weighted by gasteiger charge
**290**	N	ATSC7c	centered moreau-broto autocorrelation of lag 7 weighted by gasteiger charge
**291**	N	ATSC8c	centered moreau-broto autocorrelation of lag 8 weighted by gasteiger charge
**292**	N	ATSC0dv	centered moreau-broto autocorrelation of lag 0 weighted by valence electrons
**293**	N	ATSC1dv	centered moreau-broto autocorrelation of lag 1 weighted by valence electrons
**294**	N	ATSC2dv	centered moreau-broto autocorrelation of lag 2 weighted by valence electrons
**295**	N	ATSC3dv	centered moreau-broto autocorrelation of lag 3 weighted by valence electrons
**296**	N	ATSC4dv	centered moreau-broto autocorrelation of lag 4 weighted by valence electrons
**297**	N	ATSC5dv	centered moreau-broto autocorrelation of lag 5 weighted by valence electrons
**298**	N	ATSC6dv	centered moreau-broto autocorrelation of lag 6 weighted by valence electrons
**299**	N	ATSC7dv	centered moreau-broto autocorrelation of lag 7 weighted by valence electrons
**300**	N	ATSC8dv	centered moreau-broto autocorrelation of lag 8 weighted by valence electrons
**301**	N	ATSC0d	centered moreau-broto autocorrelation of lag 0 weighted by sigma electrons
**302**	N	ATSC1d	centered moreau-broto autocorrelation of lag 1 weighted by sigma electrons
**303**	N	ATSC2d	centered moreau-broto autocorrelation of lag 2 weighted by sigma electrons
**304**	N	ATSC3d	centered moreau-broto autocorrelation of lag 3 weighted by sigma electrons
**305**	N	ATSC4d	centered moreau-broto autocorrelation of lag 4 weighted by sigma electrons
**306**	N	ATSC5d	centered moreau-broto autocorrelation of lag 5 weighted by sigma electrons
**307**	N	ATSC6d	centered moreau-broto autocorrelation of lag 6 weighted by sigma electrons
**308**	N	ATSC7d	centered moreau-broto autocorrelation of lag 7 weighted by sigma electrons
**309**	N	ATSC8d	centered moreau-broto autocorrelation of lag 8 weighted by sigma electrons
**310**	N	ATSC0s	centered moreau-broto autocorrelation of lag 0 weighted by intrinsic state
**311**	N	ATSC1s	centered moreau-broto autocorrelation of lag 1 weighted by intrinsic state
**312**	N	ATSC2s	centered moreau-broto autocorrelation of lag 2 weighted by intrinsic state
**313**	N	ATSC3s	centered moreau-broto autocorrelation of lag 3 weighted by intrinsic state
**314**	N	ATSC4s	centered moreau-broto autocorrelation of lag 4 weighted by intrinsic state
**315**	N	ATSC5s	centered moreau-broto autocorrelation of lag 5 weighted by intrinsic state
**316**	N	ATSC6s	centered moreau-broto autocorrelation of lag 6 weighted by intrinsic state
**317**	N	ATSC7s	centered moreau-broto autocorrelation of lag 7 weighted by intrinsic state
**318**	N	ATSC8s	centered moreau-broto autocorrelation of lag 8 weighted by intrinsic state

**319**	N	ATSC0Z	centered moreau-broto autocorrelation of lag 0 weighted by atomic number
**320**	N	ATSC1Z	centered moreau-broto autocorrelation of lag 1 weighted by atomic number
**321**	N	ATSC2Z	centered moreau-broto autocorrelation of lag 2 weighted by atomic number
**322**	N	ATSC3Z	centered moreau-broto autocorrelation of lag 3 weighted by atomic number
**323**	N	ATSC4Z	centered moreau-broto autocorrelation of lag 4 weighted by atomic number
**324**	N	ATSC5Z	centered moreau-broto autocorrelation of lag 5 weighted by atomic number
**325**	N	ATSC6Z	centered moreau-broto autocorrelation of lag 6 weighted by atomic number
**326**	N	ATSC7Z	centered moreau-broto autocorrelation of lag 7 weighted by atomic number
**327**	N	ATSC8Z	centered moreau-broto autocorrelation of lag 8 weighted by atomic number
**328**	N	ATSC0m	centered moreau-broto autocorrelation of lag 0 weighted by mass
**329**	N	ATSC1m	centered moreau-broto autocorrelation of lag 1 weighted by mass
**330**	N	ATSC2m	centered moreau-broto autocorrelation of lag 2 weighted by mass
**331**	N	ATSC3m	centered moreau-broto autocorrelation of lag 3 weighted by mass
**332**	N	ATSC4m	centered moreau-broto autocorrelation of lag 4 weighted by mass
**333**	N	ATSC5m	centered moreau-broto autocorrelation of lag 5 weighted by mass
**334**	N	ATSC6m	centered moreau-broto autocorrelation of lag 6 weighted by mass
**335**	N	ATSC7m	centered moreau-broto autocorrelation of lag 7 weighted by mass
**336**	N	ATSC8m	centered moreau-broto autocorrelation of lag 8 weighted by mass
**337**	N	ATSC0v	centered moreau-broto autocorrelation of lag 0 weighted by vdw volume
**338**	N	ATSC1v	centered moreau-broto autocorrelation of lag 1 weighted by vdw volume
**339**	N	ATSC2v	centered moreau-broto autocorrelation of lag 2 weighted by vdw volume
**340**	N	ATSC3v	centered moreau-broto autocorrelation of lag 3 weighted by vdw volume
**341**	N	ATSC4v	centered moreau-broto autocorrelation of lag 4 weighted by vdw volume
**342**	N	ATSC5v	centered moreau-broto autocorrelation of lag 5 weighted by vdw volume
**343**	N	ATSC6v	centered moreau-broto autocorrelation of lag 6 weighted by vdw volume
**344**	N	ATSC7v	centered moreau-broto autocorrelation of lag 7 weighted by vdw volume
**345**	N	ATSC8v	centered moreau-broto autocorrelation of lag 8 weighted by vdw volume
**346**	N	ATSC0se	centered moreau-broto autocorrelation of lag 0 weighted by sanderson EN
**347**	N	ATSC1se	centered moreau-broto autocorrelation of lag 1 weighted by sanderson EN
**348**	N	ATSC2se	centered moreau-broto autocorrelation of lag 2 weighted by sanderson EN
**349**	N	ATSC3se	centered moreau-broto autocorrelation of lag 3 weighted by sanderson EN
**350**	N	ATSC4se	centered moreau-broto autocorrelation of lag 4 weighted by sanderson EN
**351**	N	ATSC5se	centered moreau-broto autocorrelation of lag 5 weighted by sanderson EN
**352**	N	ATSC6se	centered moreau-broto autocorrelation of lag 6 weighted by sanderson EN
**353**	N	ATSC7se	centered moreau-broto autocorrelation of lag 7 weighted by sanderson EN
**354**	N	ATSC8se	centered moreau-broto autocorrelation of lag 8 weighted by sanderson EN
**355**	N	ATSC0pe	centered moreau-broto autocorrelation of lag 0 weighted by pauling EN
**356**	N	ATSC1pe	centered moreau-broto autocorrelation of lag 1 weighted by pauling EN
**357**	N	ATSC2pe	centered moreau-broto autocorrelation of lag 2 weighted by pauling EN
**358**	N	ATSC3pe	centered moreau-broto autocorrelation of lag 3 weighted by pauling EN
**359**	N	ATSC4pe	centered moreau-broto autocorrelation of lag 4 weighted by pauling EN
**360**	N	ATSC5pe	centered moreau-broto autocorrelation of lag 5 weighted by pauling EN
**361**	N	ATSC6pe	centered moreau-broto autocorrelation of lag 6 weighted by pauling EN
**362**	N	ATSC7pe	centered moreau-broto autocorrelation of lag 7 weighted by pauling EN
**363**	N	ATSC8pe	centered moreau-broto autocorrelation of lag 8 weighted by pauling EN

**364**	N	ATSC0are	centered moreau-broto autocorrelation of lag 0 weighted by allred-rocow EN
**365**	N	ATSC1are	centered moreau-broto autocorrelation of lag 1 weighted by allred-rocow EN
**366**	N	ATSC2are	centered moreau-broto autocorrelation of lag 2 weighted by allred-rocow EN
**367**	N	ATSC3are	centered moreau-broto autocorrelation of lag 3 weighted by allred-rocow EN
**368**	N	ATSC4are	centered moreau-broto autocorrelation of lag 4 weighted by allred-rocow EN
**369**	N	ATSC5are	centered moreau-broto autocorrelation of lag 5 weighted by allred-rocow EN
**370**	N	ATSC6are	centered moreau-broto autocorrelation of lag 6 weighted by allred-rocow EN
**371**	N	ATSC7are	centered moreau-broto autocorrelation of lag 7 weighted by allred-rocow EN
**372**	N	ATSC8are	centered moreau-broto autocorrelation of lag 8 weighted by allred-rocow EN
**373**	N	ATSC0p	centered moreau-broto autocorrelation of lag 0 weighted by polarizability
**374**	N	ATSC1p	centered moreau-broto autocorrelation of lag 1 weighted by polarizability
**375**	N	ATSC2p	centered moreau-broto autocorrelation of lag 2 weighted by polarizability
**376**	N	ATSC3p	centered moreau-broto autocorrelation of lag 3 weighted by polarizability
**377**	N	ATSC4p	centered moreau-broto autocorrelation of lag 4 weighted by polarizability
**378**	N	ATSC5p	centered moreau-broto autocorrelation of lag 5 weighted by polarizability
**379**	N	ATSC6p	centered moreau-broto autocorrelation of lag 6 weighted by polarizability
**380**	N	ATSC7p	centered moreau-broto autocorrelation of lag 7 weighted by polarizability
**381**	N	ATSC8p	centered moreau-broto autocorrelation of lag 8 weighted by polarizability
**382**	N	ATSC0i	centered moreau-broto autocorrelation of lag 0 weighted by ionization potential
**383**	N	ATSC1i	centered moreau-broto autocorrelation of lag 1 weighted by ionization potential
**384**	N	ATSC2i	centered moreau-broto autocorrelation of lag 2 weighted by ionization potential
**385**	N	ATSC3i	centered moreau-broto autocorrelation of lag 3 weighted by ionization potential
**386**	N	ATSC4i	centered moreau-broto autocorrelation of lag 4 weighted by ionization potential
**387**	N	ATSC5i	centered moreau-broto autocorrelation of lag 5 weighted by ionization potential
**388**	N	ATSC6i	centered moreau-broto autocorrelation of lag 6 weighted by ionization potential
**389**	N	ATSC7i	centered moreau-broto autocorrelation of lag 7 weighted by ionization potential
**390**	N	ATSC8i	centered moreau-broto autocorrelation of lag 8 weighted by ionization potential
**391**	N	AATSC0c	averaged and centered moreau-broto autocorrelation of lag 0 weighted by gasteiger charge
**392**	N	AATSC1c	averaged and centered moreau-broto autocorrelation of lag 1 weighted by gasteiger charge
**393**	N	AATSC2c	averaged and centered moreau-broto autocorrelation of lag 2 weighted by gasteiger charge
**394**	N	AATSC3c	averaged and centered moreau-broto autocorrelation of lag 3 weighted by gasteiger charge
**395**	N	AATSC4c	averaged and centered moreau-broto autocorrelation of lag 4 weighted by gasteiger charge
**396**	N	AATSC5c	averaged and centered moreau-broto autocorrelation of lag 5 weighted by gasteiger charge
**397**	N	AATSC6c	averaged and centered moreau-broto autocorrelation of lag 6 weighted by gasteiger charge
**398**	N	AATSC7c	averaged and centered moreau-broto autocorrelation of lag 7 weighted by gasteiger charge
**399**	N	AATSC8c	averaged and centered moreau-broto autocorrelation of lag 8 weighted by gasteiger charge
**400**	N	AATSC0dv	averaged and centered moreau-broto autocorrelation of lag 0 weighted by valence electrons
**401**	N	AATSC1dv	averaged and centered moreau-broto autocorrelation of lag 1 weighted by valence electrons
**402**	N	AATSC2dv	averaged and centered moreau-broto autocorrelation of lag 2 weighted by valence electrons
**403**	N	AATSC3dv	averaged and centered moreau-broto autocorrelation of lag 3 weighted by valence electrons
**404**	N	AATSC4dv	averaged and centered moreau-broto autocorrelation of lag 4 weighted by valence electrons
**405**	N	AATSC5dv	averaged and centered moreau-broto autocorrelation of lag 5 weighted by valence electrons
**406**	N	AATSC6dv	averaged and centered moreau-broto autocorrelation of lag 6 weighted by valence electrons
**407**	N	AATSC7dv	averaged and centered moreau-broto autocorrelation of lag 7 weighted by valence electrons
**408**	N	AATSC8dv	averaged and centered moreau-broto autocorrelation of lag 8 weighted by valence electrons

**409**	N	AATSC0d	averaged and centered moreau-broto autocorrelation of lag 0 weighted by sigma electrons
**410**	N	AATSC1d	averaged and centered moreau-broto autocorrelation of lag 1 weighted by sigma electrons
**411**	N	AATSC2d	averaged and centered moreau-broto autocorrelation of lag 2 weighted by sigma electrons
**412**	N	AATSC3d	averaged and centered moreau-broto autocorrelation of lag 3 weighted by sigma electrons
**413**	N	AATSC4d	averaged and centered moreau-broto autocorrelation of lag 4 weighted by sigma electrons
**414**	N	AATSC5d	averaged and centered moreau-broto autocorrelation of lag 5 weighted by sigma electrons
**415**	N	AATSC6d	averaged and centered moreau-broto autocorrelation of lag 6 weighted by sigma electrons
**416**	N	AATSC7d	averaged and centered moreau-broto autocorrelation of lag 7 weighted by sigma electrons
**417**	N	AATSC8d	averaged and centered moreau-broto autocorrelation of lag 8 weighted by sigma electrons
**418**	N	AATSC0s	averaged and centered moreau-broto autocorrelation of lag 0 weighted by intrinsic state
**419**	N	AATSC1s	averaged and centered moreau-broto autocorrelation of lag 1 weighted by intrinsic state
**420**	N	AATSC2s	averaged and centered moreau-broto autocorrelation of lag 2 weighted by intrinsic state
**421**	N	AATSC3s	averaged and centered moreau-broto autocorrelation of lag 3 weighted by intrinsic state
**422**	N	AATSC4s	averaged and centered moreau-broto autocorrelation of lag 4 weighted by intrinsic state
**423**	N	AATSC5s	averaged and centered moreau-broto autocorrelation of lag 5 weighted by intrinsic state
**424**	N	AATSC6s	averaged and centered moreau-broto autocorrelation of lag 6 weighted by intrinsic state
**425**	N	AATSC7s	averaged and centered moreau-broto autocorrelation of lag 7 weighted by intrinsic state
**426**	N	AATSC8s	averaged and centered moreau-broto autocorrelation of lag 8 weighted by intrinsic state
**427**	N	AATSC0Z	averaged and centered moreau-broto autocorrelation of lag 0 weighted by atomic number
**428**	N	AATSC1Z	averaged and centered moreau-broto autocorrelation of lag 1 weighted by atomic number
**429**	N	AATSC2Z	averaged and centered moreau-broto autocorrelation of lag 2 weighted by atomic number
**430**	N	AATSC3Z	averaged and centered moreau-broto autocorrelation of lag 3 weighted by atomic number
**431**	N	AATSC4Z	averaged and centered moreau-broto autocorrelation of lag 4 weighted by atomic number
**432**	N	AATSC5Z	averaged and centered moreau-broto autocorrelation of lag 5 weighted by atomic number
**433**	N	AATSC6Z	averaged and centered moreau-broto autocorrelation of lag 6 weighted by atomic number
**434**	N	AATSC7Z	averaged and centered moreau-broto autocorrelation of lag 7 weighted by atomic number
**435**	N	AATSC8Z	averaged and centered moreau-broto autocorrelation of lag 8 weighted by atomic number
**436**	N	AATSC0m	averaged and centered moreau-broto autocorrelation of lag 0 weighted by mass
**437**	N	AATSC1m	averaged and centered moreau-broto autocorrelation of lag 1 weighted by mass
**438**	N	AATSC2m	averaged and centered moreau-broto autocorrelation of lag 2 weighted by mass
**439**	N	AATSC3m	averaged and centered moreau-broto autocorrelation of lag 3 weighted by mass
**440**	N	AATSC4m	averaged and centered moreau-broto autocorrelation of lag 4 weighted by mass
**441**	N	AATSC5m	averaged and centered moreau-broto autocorrelation of lag 5 weighted by mass
**442**	N	AATSC6m	averaged and centered moreau-broto autocorrelation of lag 6 weighted by mass
**443**	N	AATSC7m	averaged and centered moreau-broto autocorrelation of lag 7 weighted by mass
**444**	N	AATSC8m	averaged and centered moreau-broto autocorrelation of lag 8 weighted by mass
**445**	N	AATSC0v	averaged and centered moreau-broto autocorrelation of lag 0 weighted by vdw volume
**446**	N	AATSC1v	averaged and centered moreau-broto autocorrelation of lag 1 weighted by vdw volume
**447**	N	AATSC2v	averaged and centered moreau-broto autocorrelation of lag 2 weighted by vdw volume
**448**	N	AATSC3v	averaged and centered moreau-broto autocorrelation of lag 3 weighted by vdw volume
**449**	N	AATSC4v	averaged and centered moreau-broto autocorrelation of lag 4 weighted by vdw volume
**450**	N	AATSC5v	averaged and centered moreau-broto autocorrelation of lag 5 weighted by vdw volume
**451**	N	AATSC6v	averaged and centered moreau-broto autocorrelation of lag 6 weighted by vdw volume
**452**	N	AATSC7v	averaged and centered moreau-broto autocorrelation of lag 7 weighted by vdw volume
**453**	N	AATSC8v	averaged and centered moreau-broto autocorrelation of lag 8 weighted by vdw volume

**454**	N	AATSC0se	averaged and centered moreau-broto autocorrelation of lag 0 weighted by sanderson EN
**455**	N	AATSC1se	averaged and centered moreau-broto autocorrelation of lag 1 weighted by sanderson EN
**456**	N	AATSC2se	averaged and centered moreau-broto autocorrelation of lag 2 weighted by sanderson EN
**457**	N	AATSC3se	averaged and centered moreau-broto autocorrelation of lag 3 weighted by sanderson EN
**458**	N	AATSC4se	averaged and centered moreau-broto autocorrelation of lag 4 weighted by sanderson EN
**459**	N	AATSC5se	averaged and centered moreau-broto autocorrelation of lag 5 weighted by sanderson EN
**460**	N	AATSC6se	averaged and centered moreau-broto autocorrelation of lag 6 weighted by sanderson EN
**461**	N	AATSC7se	averaged and centered moreau-broto autocorrelation of lag 7 weighted by sanderson EN
**462**	N	AATSC8se	averaged and centered moreau-broto autocorrelation of lag 8 weighted by sanderson EN
**463**	N	AATSC0pe	averaged and centered moreau-broto autocorrelation of lag 0 weighted by pauling EN
**464**	N	AATSC1pe	averaged and centered moreau-broto autocorrelation of lag 1 weighted by pauling EN
**465**	N	AATSC2pe	averaged and centered moreau-broto autocorrelation of lag 2 weighted by pauling EN
**466**	N	AATSC3pe	averaged and centered moreau-broto autocorrelation of lag 3 weighted by pauling EN
**467**	N	AATSC4pe	averaged and centered moreau-broto autocorrelation of lag 4 weighted by pauling EN
**468**	N	AATSC5pe	averaged and centered moreau-broto autocorrelation of lag 5 weighted by pauling EN
**469**	N	AATSC6pe	averaged and centered moreau-broto autocorrelation of lag 6 weighted by pauling EN
**470**	N	AATSC7pe	averaged and centered moreau-broto autocorrelation of lag 7 weighted by pauling EN
**471**	N	AATSC8pe	averaged and centered moreau-broto autocorrelation of lag 8 weighted by pauling EN
**472**	N	AATSC0are	averaged and centered moreau-broto autocorrelation of lag 0 weighted by allred-rocow EN
**473**	N	AATSC1are	averaged and centered moreau-broto autocorrelation of lag 1 weighted by allred-rocow EN
**474**	N	AATSC2are	averaged and centered moreau-broto autocorrelation of lag 2 weighted by allred-rocow EN
**475**	N	AATSC3are	averaged and centered moreau-broto autocorrelation of lag 3 weighted by allred-rocow EN
**476**	N	AATSC4are	averaged and centered moreau-broto autocorrelation of lag 4 weighted by allred-rocow EN
**477**	N	AATSC5are	averaged and centered moreau-broto autocorrelation of lag 5 weighted by allred-rocow EN
**478**	N	AATSC6are	averaged and centered moreau-broto autocorrelation of lag 6 weighted by allred-rocow EN
**479**	N	AATSC7are	averaged and centered moreau-broto autocorrelation of lag 7 weighted by allred-rocow EN
**480**	N	AATSC8are	averaged and centered moreau-broto autocorrelation of lag 8 weighted by allred-rocow EN
**481**	N	AATSC0p	averaged and centered moreau-broto autocorrelation of lag 0 weighted by polarizability
**482**	N	AATSC1p	averaged and centered moreau-broto autocorrelation of lag 1 weighted by polarizability
**483**	N	AATSC2p	averaged and centered moreau-broto autocorrelation of lag 2 weighted by polarizability
**484**	N	AATSC3p	averaged and centered moreau-broto autocorrelation of lag 3 weighted by polarizability
**485**	N	AATSC4p	averaged and centered moreau-broto autocorrelation of lag 4 weighted by polarizability
**486**	N	AATSC5p	averaged and centered moreau-broto autocorrelation of lag 5 weighted by polarizability
**487**	N	AATSC6p	averaged and centered moreau-broto autocorrelation of lag 6 weighted by polarizability
**488**	N	AATSC7p	averaged and centered moreau-broto autocorrelation of lag 7 weighted by polarizability
**489**	N	AATSC8p	averaged and centered moreau-broto autocorrelation of lag 8 weighted by polarizability
**490**	N	AATSC0i	averaged and centered moreau-broto autocorrelation of lag 0 weighted by ionization potential
**491**	N	AATSC1i	averaged and centered moreau-broto autocorrelation of lag 1 weighted by ionization potential
**492**	N	AATSC2i	averaged and centered moreau-broto autocorrelation of lag 2 weighted by ionization potential
**493**	N	AATSC3i	averaged and centered moreau-broto autocorrelation of lag 3 weighted by ionization potential
**494**	N	AATSC4i	averaged and centered moreau-broto autocorrelation of lag 4 weighted by ionization potential

**495**	N	AATSC5i	averaged and centered moreau-broto autocorrelation of lag 5 weighted by ionization potential
**496**	N	AATSC6i	averaged and centered moreau-broto autocorrelation of lag 6 weighted by ionization potential
**497**	N	AATSC7i	averaged and centered moreau-broto autocorrelation of lag 7 weighted by ionization potential
**498**	N	AATSC8i	averaged and centered moreau-broto autocorrelation of lag 8 weighted by ionization potential
**499**	N	MATS1c	moran coefficient of lag 1 weighted by gasteiger charge
**500**	N	MATS2c	moran coefficient of lag 2 weighted by gasteiger charge
**501**	N	MATS3c	moran coefficient of lag 3 weighted by gasteiger charge
**502**	N	MATS4c	moran coefficient of lag 4 weighted by gasteiger charge
**503**	N	MATS5c	moran coefficient of lag 5 weighted by gasteiger charge
**504**	N	MATS6c	moran coefficient of lag 6 weighted by gasteiger charge
**505**	N	MATS7c	moran coefficient of lag 7 weighted by gasteiger charge
**506**	N	MATS8c	moran coefficient of lag 8 weighted by gasteiger charge
**507**	N	MATS1dv	moran coefficient of lag 1 weighted by valence electrons
**508**	N	MATS2dv	moran coefficient of lag 2 weighted by valence electrons
**509**	N	MATS3dv	moran coefficient of lag 3 weighted by valence electrons
**510**	N	MATS4dv	moran coefficient of lag 4 weighted by valence electrons
**511**	N	MATS5dv	moran coefficient of lag 5 weighted by valence electrons
**512**	N	MATS6dv	moran coefficient of lag 6 weighted by valence electrons
**513**	N	MATS7dv	moran coefficient of lag 7 weighted by valence electrons
**514**	N	MATS8dv	moran coefficient of lag 8 weighted by valence electrons
**515**	N	MATS1d	moran coefficient of lag 1 weighted by sigma electrons
**516**	N	MATS2d	moran coefficient of lag 2 weighted by sigma electrons
**517**	N	MATS3d	moran coefficient of lag 3 weighted by sigma electrons
**518**	N	MATS4d	moran coefficient of lag 4 weighted by sigma electrons
**519**	N	MATS5d	moran coefficient of lag 5 weighted by sigma electrons
**520**	N	MATS6d	moran coefficient of lag 6 weighted by sigma electrons
**521**	N	MATS7d	moran coefficient of lag 7 weighted by sigma electrons
**522**	N	MATS8d	moran coefficient of lag 8 weighted by sigma electrons
**523**	N	MATS1s	moran coefficient of lag 1 weighted by intrinsic state
**524**	N	MATS2s	moran coefficient of lag 2 weighted by intrinsic state
**525**	N	MATS3s	moran coefficient of lag 3 weighted by intrinsic state
**526**	N	MATS4s	moran coefficient of lag 4 weighted by intrinsic state
**527**	N	MATS5s	moran coefficient of lag 5 weighted by intrinsic state
**528**	N	MATS6s	moran coefficient of lag 6 weighted by intrinsic state
**529**	N	MATS7s	moran coefficient of lag 7 weighted by intrinsic state
**530**	N	MATS8s	moran coefficient of lag 8 weighted by intrinsic state
**531**	N	MATS1Z	moran coefficient of lag 1 weighted by atomic number
**532**	N	MATS2Z	moran coefficient of lag 2 weighted by atomic number
**533**	N	MATS3Z	moran coefficient of lag 3 weighted by atomic number
**534**	N	MATS4Z	moran coefficient of lag 4 weighted by atomic number
**535**	N	MATS5Z	moran coefficient of lag 5 weighted by atomic number
**536**	N	MATS6Z	moran coefficient of lag 6 weighted by atomic number

**537**	N	MATS7Z	moran coefficient of lag 7 weighted by atomic number
**538**	N	MATS8Z	moran coefficient of lag 8 weighted by atomic number
**539**	N	MATS1m	moran coefficient of lag 1 weighted by mass
**540**	N	MATS2m	moran coefficient of lag 2 weighted by mass
**541**	N	MATS3m	moran coefficient of lag 3 weighted by mass
**542**	N	MATS4m	moran coefficient of lag 4 weighted by mass
**543**	N	MATS5m	moran coefficient of lag 5 weighted by mass
**544**	N	MATS6m	moran coefficient of lag 6 weighted by mass
**545**	N	MATS7m	moran coefficient of lag 7 weighted by mass
**546**	N	MATS8m	moran coefficient of lag 8 weighted by mass
**547**	N	MATS1v	moran coefficient of lag 1 weighted by vdw volume
**548**	N	MATS2v	moran coefficient of lag 2 weighted by vdw volume
**549**	N	MATS3v	moran coefficient of lag 3 weighted by vdw volume
**550**	N	MATS4v	moran coefficient of lag 4 weighted by vdw volume
**551**	N	MATS5v	moran coefficient of lag 5 weighted by vdw volume
**552**	N	MATS6v	moran coefficient of lag 6 weighted by vdw volume
**553**	N	MATS7v	moran coefficient of lag 7 weighted by vdw volume
**554**	N	MATS8v	moran coefficient of lag 8 weighted by vdw volume
**555**	N	MATS1se	moran coefficient of lag 1 weighted by sanderson EN
**556**	N	MATS2se	moran coefficient of lag 2 weighted by sanderson EN
**557**	N	MATS3se	moran coefficient of lag 3 weighted by sanderson EN
**558**	N	MATS4se	moran coefficient of lag 4 weighted by sanderson EN
**559**	N	MATS5se	moran coefficient of lag 5 weighted by sanderson EN
**560**	N	MATS6se	moran coefficient of lag 6 weighted by sanderson EN
**561**	N	MATS7se	moran coefficient of lag 7 weighted by sanderson EN
**562**	N	MATS8se	moran coefficient of lag 8 weighted by sanderson EN
**563**	N	MATS1pe	moran coefficient of lag 1 weighted by pauling EN
**564**	N	MATS2pe	moran coefficient of lag 2 weighted by pauling EN
**565**	N	MATS3pe	moran coefficient of lag 3 weighted by pauling EN
**566**	N	MATS4pe	moran coefficient of lag 4 weighted by pauling EN
**567**	N	MATS5pe	moran coefficient of lag 5 weighted by pauling EN
**568**	N	MATS6pe	moran coefficient of lag 6 weighted by pauling EN
**569**	N	MATS7pe	moran coefficient of lag 7 weighted by pauling EN
**570**	N	MATS8pe	moran coefficient of lag 8 weighted by pauling EN
**571**	N	MATS1are	moran coefficient of lag 1 weighted by allred-rocow EN
**572**	N	MATS2are	moran coefficient of lag 2 weighted by allred-rocow EN
**573**	N	MATS3are	moran coefficient of lag 3 weighted by allred-rocow EN
**574**	N	MATS4are	moran coefficient of lag 4 weighted by allred-rocow EN
**575**	N	MATS5are	moran coefficient of lag 5 weighted by allred-rocow EN
**576**	N	MATS6are	moran coefficient of lag 6 weighted by allred-rocow EN
**577**	N	MATS7are	moran coefficient of lag 7 weighted by allred-rocow EN
**578**	N	MATS8are	moran coefficient of lag 8 weighted by allred-rocow EN
**579**	N	MATS1p	moran coefficient of lag 1 weighted by polarizability
**580**	N	MATS2p	moran coefficient of lag 2 weighted by polarizability
**581**	N	MATS3p	moran coefficient of lag 3 weighted by polarizability

**582**	N	MATS4p	moran coefficient of lag 4 weighted by polarizability
**583**	N	MATS5p	moran coefficient of lag 5 weighted by polarizability
**584**	N	MATS6p	moran coefficient of lag 6 weighted by polarizability
**585**	N	MATS7p	moran coefficient of lag 7 weighted by polarizability
**586**	N	MATS8p	moran coefficient of lag 8 weighted by polarizability
**587**	N	MATS1i	moran coefficient of lag 1 weighted by ionization potential
**588**	N	MATS2i	moran coefficient of lag 2 weighted by ionization potential
**589**	N	MATS3i	moran coefficient of lag 3 weighted by ionization potential
**590**	N	MATS4i	moran coefficient of lag 4 weighted by ionization potential
**591**	N	MATS5i	moran coefficient of lag 5 weighted by ionization potential
**592**	N	MATS6i	moran coefficient of lag 6 weighted by ionization potential
**593**	N	MATS7i	moran coefficient of lag 7 weighted by ionization potential
**594**	N	MATS8i	moran coefficient of lag 8 weighted by ionization potential
**595**	N	GATS1c	geary coefficient of lag 1 weighted by gasteiger charge
**596**	N	GATS2c	geary coefficient of lag 2 weighted by gasteiger charge
**597**	N	GATS3c	geary coefficient of lag 3 weighted by gasteiger charge
**598**	N	GATS4c	geary coefficient of lag 4 weighted by gasteiger charge
**599**	N	GATS5c	geary coefficient of lag 5 weighted by gasteiger charge
**600**	N	GATS6c	geary coefficient of lag 6 weighted by gasteiger charge
**601**	N	GATS7c	geary coefficient of lag 7 weighted by gasteiger charge
**602**	N	GATS8c	geary coefficient of lag 8 weighted by gasteiger charge
**603**	N	GATS1dv	geary coefficient of lag 1 weighted by valence electrons
**604**	N	GATS2dv	geary coefficient of lag 2 weighted by valence electrons
**605**	N	GATS3dv	geary coefficient of lag 3 weighted by valence electrons
**606**	N	GATS4dv	geary coefficient of lag 4 weighted by valence electrons
**607**	N	GATS5dv	geary coefficient of lag 5 weighted by valence electrons
**608**	N	GATS6dv	geary coefficient of lag 6 weighted by valence electrons
**609**	N	GATS7dv	geary coefficient of lag 7 weighted by valence electrons
**610**	N	GATS8dv	geary coefficient of lag 8 weighted by valence electrons
**611**	N	GATS1d	geary coefficient of lag 1 weighted by sigma electrons
**612**	N	GATS2d	geary coefficient of lag 2 weighted by sigma electrons
**613**	N	GATS3d	geary coefficient of lag 3 weighted by sigma electrons
**614**	N	GATS4d	geary coefficient of lag 4 weighted by sigma electrons
**615**	N	GATS5d	geary coefficient of lag 5 weighted by sigma electrons
**616**	N	GATS6d	geary coefficient of lag 6 weighted by sigma electrons
**617**	N	GATS7d	geary coefficient of lag 7 weighted by sigma electrons
**618**	N	GATS8d	geary coefficient of lag 8 weighted by sigma electrons
**619**	N	GATS1s	geary coefficient of lag 1 weighted by intrinsic state
**620**	N	GATS2s	geary coefficient of lag 2 weighted by intrinsic state
**621**	N	GATS3s	geary coefficient of lag 3 weighted by intrinsic state
**622**	N	GATS4s	geary coefficient of lag 4 weighted by intrinsic state
**623**	N	GATS5s	geary coefficient of lag 5 weighted by intrinsic state
**624**	N	GATS6s	geary coefficient of lag 6 weighted by intrinsic state
**625**	N	GATS7s	geary coefficient of lag 7 weighted by intrinsic state
**626**	N	GATS8s	geary coefficient of lag 8 weighted by intrinsic state

**627**	N	GATS1Z	geary coefficient of lag 1 weighted by atomic number
**628**	N	GATS2Z	geary coefficient of lag 2 weighted by atomic number
**629**	N	GATS3Z	geary coefficient of lag 3 weighted by atomic number
**630**	N	GATS4Z	geary coefficient of lag 4 weighted by atomic number
**631**	N	GATS5Z	geary coefficient of lag 5 weighted by atomic number
**632**	N	GATS6Z	geary coefficient of lag 6 weighted by atomic number
**633**	N	GATS7Z	geary coefficient of lag 7 weighted by atomic number
**634**	N	GATS8Z	geary coefficient of lag 8 weighted by atomic number
**635**	N	GATS1m	geary coefficient of lag 1 weighted by mass
**636**	N	GATS2m	geary coefficient of lag 2 weighted by mass
**637**	N	GATS3m	geary coefficient of lag 3 weighted by mass
**638**	N	GATS4m	geary coefficient of lag 4 weighted by mass
**639**	N	GATS5m	geary coefficient of lag 5 weighted by mass
**640**	N	GATS6m	geary coefficient of lag 6 weighted by mass
**641**	N	GATS7m	geary coefficient of lag 7 weighted by mass
**642**	N	GATS8m	geary coefficient of lag 8 weighted by mass
**643**	N	GATS1v	geary coefficient of lag 1 weighted by vdw volume
**644**	N	GATS2v	geary coefficient of lag 2 weighted by vdw volume
**645**	N	GATS3v	geary coefficient of lag 3 weighted by vdw volume
**646**	N	GATS4v	geary coefficient of lag 4 weighted by vdw volume
**647**	N	GATS5v	geary coefficient of lag 5 weighted by vdw volume
**648**	N	GATS6v	geary coefficient of lag 6 weighted by vdw volume
**649**	N	GATS7v	geary coefficient of lag 7 weighted by vdw volume
**650**	N	GATS8v	geary coefficient of lag 8 weighted by vdw volume
**651**	N	GATS1se	geary coefficient of lag 1 weighted by sanderson EN
**652**	N	GATS2se	geary coefficient of lag 2 weighted by sanderson EN
**653**	N	GATS3se	geary coefficient of lag 3 weighted by sanderson EN
**654**	N	GATS4se	geary coefficient of lag 4 weighted by sanderson EN
**655**	N	GATS5se	geary coefficient of lag 5 weighted by sanderson EN
**656**	N	GATS6se	geary coefficient of lag 6 weighted by sanderson EN
**657**	N	GATS7se	geary coefficient of lag 7 weighted by sanderson EN
**658**	N	GATS8se	geary coefficient of lag 8 weighted by sanderson EN
**659**	N	GATS1pe	geary coefficient of lag 1 weighted by pauling EN
**660**	N	GATS2pe	geary coefficient of lag 2 weighted by pauling EN
**661**	N	GATS3pe	geary coefficient of lag 3 weighted by pauling EN
**662**	N	GATS4pe	geary coefficient of lag 4 weighted by pauling EN
**663**	N	GATS5pe	geary coefficient of lag 5 weighted by pauling EN
**664**	N	GATS6pe	geary coefficient of lag 6 weighted by pauling EN
**665**	N	GATS7pe	geary coefficient of lag 7 weighted by pauling EN
**666**	N	GATS8pe	geary coefficient of lag 8 weighted by pauling EN
**667**	N	GATS1are	geary coefficient of lag 1 weighted by allred-rocow EN
**668**	N	GATS2are	geary coefficient of lag 2 weighted by allred-rocow EN
**669**	N	GATS3are	geary coefficient of lag 3 weighted by allred-rocow EN
**670**	N	GATS4are	geary coefficient of lag 4 weighted by allred-rocow EN
**671**	N	GATS5are	geary coefficient of lag 5 weighted by allred-rocow EN

**672**	N	GATS6are	geary coefficient of lag 6 weighted by allred-rocow EN
**673**	N	GATS7are	geary coefficient of lag 7 weighted by allred-rocow EN
**674**	N	GATS8are	geary coefficient of lag 8 weighted by allred-rocow EN
**675**	N	GATS1p	geary coefficient of lag 1 weighted by polarizability
**676**	N	GATS2p	geary coefficient of lag 2 weighted by polarizability
**677**	N	GATS3p	geary coefficient of lag 3 weighted by polarizability
**678**	N	GATS4p	geary coefficient of lag 4 weighted by polarizability
**679**	N	GATS5p	geary coefficient of lag 5 weighted by polarizability
**680**	N	GATS6p	geary coefficient of lag 6 weighted by polarizability
**681**	N	GATS7p	geary coefficient of lag 7 weighted by polarizability
**682**	N	GATS8p	geary coefficient of lag 8 weighted by polarizability
**683**	N	GATS1i	geary coefficient of lag 1 weighted by ionization potential
**684**	N	GATS2i	geary coefficient of lag 2 weighted by ionization potential
**685**	N	GATS3i	geary coefficient of lag 3 weighted by ionization potential
**686**	N	GATS4i	geary coefficient of lag 4 weighted by ionization potential
**687**	N	GATS5i	geary coefficient of lag 5 weighted by ionization potential
**688**	N	GATS6i	geary coefficient of lag 6 weighted by ionization potential
**689**	N	GATS7i	geary coefficient of lag 7 weighted by ionization potential
**690**	N	GATS8i	geary coefficient of lag 8 weighted by ionization potential
**691**	N	BalabanJ	Balaban's J index
**692**	N	SpAbs_DzZ	graph energy from Barysz matrix weighted by atomic number
**693**	N	SpMax_DzZ	leading eigenvalue from Barysz matrix weighted by atomic number
**694**	N	SpDiam_DzZ	spectral diamiter from Barysz matrix weighted by atomic number
**695**	N	SpAD_DzZ	spectral absolute diviation from Barysz matrix weighted by atomic number
**696**	N	SpMAD_DzZ	spectral mean absolute diviation from Barysz matrix weighted by atomic number
**697**	N	LogEE_DzZ	Estrada-like index from Barysz matrix weighted by atomic number
**698**	N	SM1_DzZ	spectral moment from Barysz matrix weighted by atomic number
**699**	N	VE1_DzZ	coefficient sum of the last eigenvector from Barysz matrix weighted by atomic number
**700**	N	VE2_DzZ	average coefficient of the last eigenvector from Barysz matrix weighted by atomic number
**701**	N	VE3_DzZ	logarithmic coefficient sum of the last eigenvector from Barysz matrix weighted by atomic number
**702**	N	VR1_DzZ	Randic-like eigenvector-based index from Barysz matrix weighted by atomic number
**703**	N	VR2_DzZ	normalized Randic-like eigenvector-based index from Barysz matrix weighted by atomic number
**704**	N	VR3_DzZ	logarithmic Randic-like eigenvector-based index from Barysz matrix weighted by atomic number
**705**	N	SpAbs_Dzm	graph energy from Barysz matrix weighted by mass
**706**	N	SpMax_Dzm	leading eigenvalue from Barysz matrix weighted by mass
**707**	N	SpDiam_Dzm	spectral diamiter from Barysz matrix weighted by mass
**708**	N	SpAD_Dzm	spectral absolute diviation from Barysz matrix weighted by mass
**709**	N	SpMAD_Dzm	spectral mean absolute diviation from Barysz matrix weighted by mass
**710**	N	LogEE_Dzm	Estrada-like index from Barysz matrix weighted by mass
**711**	N	SM1_Dzm	spectral moment from Barysz matrix weighted by mass
**712**	N	VE1_Dzm	coefficient sum of the last eigenvector from Barysz matrix weighted by mass
**713**	N	VE2_Dzm	average coefficient of the last eigenvector from Barysz matrix weighted by mass
**714**	N	VE3_Dzm	logarithmic coefficient sum of the last eigenvector from Barysz matrix weighted by mass

**715**	N	VR1_Dzm	Randic-like eigenvector-based index from Barysz matrix weighted by mass
**716**	N	VR2_Dzm	normalized Randic-like eigenvector-based index from Barysz matrix weighted by mass
**717**	N	VR3_Dzm	logarithmic Randic-like eigenvector-based index from Barysz matrix weighted by mass
**718**	N	SpAbs_Dzv	graph energy from Barysz matrix weighted by vdw volume
**719**	N	SpMax_Dzv	leading eigenvalue from Barysz matrix weighted by vdw volume
**720**	N	SpDiam_Dzv	spectral diamiter from Barysz matrix weighted by vdw volume
**721**	N	SpAD_Dzv	spectral absolute diviation from Barysz matrix weighted by vdw volume
**722**	N	SpMAD_Dzv	spectral mean absolute diviation from Barysz matrix weighted by vdw volume
**723**	N	LogEE_Dzv	Estrada-like index from Barysz matrix weighted by vdw volume
**724**	N	SM1_Dzv	spectral moment from Barysz matrix weighted by vdw volume
**725**	N	VE1_Dzv	coefficient sum of the last eigenvector from Barysz matrix weighted by vdw volume
**726**	N	VE2_Dzv	average coefficient of the last eigenvector from Barysz matrix weighted by vdw volume
**727**	N	VE3_Dzv	logarithmic coefficient sum of the last eigenvector from Barysz matrix weighted by vdw volume
**728**	N	VR1_Dzv	Randic-like eigenvector-based index from Barysz matrix weighted by vdw volume
**729**	N	VR2_Dzv	normalized Randic-like eigenvector-based index from Barysz matrix weighted by vdw volume
**730**	N	VR3_Dzv	logarithmic Randic-like eigenvector-based index from Barysz matrix weighted by vdw volume
**731**	N	SpAbs_Dzse	graph energy from Barysz matrix weighted by sanderson EN
**732**	N	SpMax_Dzse	leading eigenvalue from Barysz matrix weighted by sanderson EN
**733**	N	SpDiam_Dzse	spectral diamiter from Barysz matrix weighted by sanderson EN
**734**	N	SpAD_Dzse	spectral absolute diviation from Barysz matrix weighted by sanderson EN
**735**	N	SpMAD_Dzse	spectral mean absolute diviation from Barysz matrix weighted by sanderson EN
**736**	N	LogEE_Dzse	Estrada-like index from Barysz matrix weighted by sanderson EN
**737**	N	SM1_Dzse	spectral moment from Barysz matrix weighted by sanderson EN
**738**	N	VE1_Dzse	coefficient sum of the last eigenvector from Barysz matrix weighted by sanderson EN
**739**	N	VE2_Dzse	average coefficient of the last eigenvector from Barysz matrix weighted by sanderson EN
**740**	N	VE3_Dzse	logarithmic coefficient sum of the last eigenvector from Barysz matrix weighted by sanderson EN
**741**	N	VR1_Dzse	Randic-like eigenvector-based index from Barysz matrix weighted by sanderson EN
**742**	N	VR2_Dzse	normalized Randic-like eigenvector-based index from Barysz matrix weighted by sanderson EN
**743**	N	VR3_Dzse	logarithmic Randic-like eigenvector-based index from Barysz matrix weighted by sanderson EN
**744**	N	SpAbs_Dzpe	graph energy from Barysz matrix weighted by pauling EN
**745**	N	SpMax_Dzpe	leading eigenvalue from Barysz matrix weighted by pauling EN
**746**	N	SpDiam_Dzpe	spectral diamiter from Barysz matrix weighted by pauling EN
**747**	N	SpAD_Dzpe	spectral absolute diviation from Barysz matrix weighted by pauling EN
**748**	N	SpMAD_Dzpe	spectral mean absolute diviation from Barysz matrix weighted by pauling EN
**749**	N	LogEE_Dzpe	Estrada-like index from Barysz matrix weighted by pauling EN
**750**	N	SM1_Dzpe	spectral moment from Barysz matrix weighted by pauling EN
**751**	N	VE1_Dzpe	coefficient sum of the last eigenvector from Barysz matrix weighted by pauling EN
**752**	N	VE2_Dzpe	average coefficient of the last eigenvector from Barysz matrix weighted by pauling EN
**753**	N	VE3_Dzpe	logarithmic coefficient sum of the last eigenvector from Barysz matrix weighted by pauling EN
**754**	N	VR1_Dzpe	Randic-like eigenvector-based index from Barysz matrix weighted by pauling EN
**755**	N	VR2_Dzpe	normalized Randic-like eigenvector-based index from Barysz matrix weighted by pauling EN

**756**	N	VR3_Dzpe	logarithmic Randic-like eigenvector-based index from Barysz matrix weighted by pauling EN
**757**	N	SpAbs_Dzare	graph energy from Barysz matrix weighted by allred-rocow EN
**758**	N	SpMax_Dzare	leading eigenvalue from Barysz matrix weighted by allred-rocow EN
**759**	N	SpDiam_Dzare	spectral diamiter from Barysz matrix weighted by allred-rocow EN
**760**	N	SpAD_Dzare	spectral absolute diviation from Barysz matrix weighted by allred-rocow EN
**761**	N	SpMAD_Dzare	spectral mean absolute diviation from Barysz matrix weighted by allred-rocow EN
**762**	N	LogEE_Dzare	Estrada-like index from Barysz matrix weighted by allred-rocow EN
**763**	N	SM1_Dzare	spectral moment from Barysz matrix weighted by allred-rocow EN
**764**	N	VE1_Dzare	coefficient sum of the last eigenvector from Barysz matrix weighted by allred-rocow EN
**765**	N	VE2_Dzare	average coefficient of the last eigenvector from Barysz matrix weighted by allred-rocow EN
**766**	N	VE3_Dzare	logarithmic coefficient sum of the last eigenvector from Barysz matrix weighted by allred-rocow EN
**767**	N	VR1_Dzare	Randic-like eigenvector-based index from Barysz matrix weighted by allred-rocow EN
**768**	N	VR2_Dzare	normalized Randic-like eigenvector-based index from Barysz matrix weighted by allred-rocow EN
**769**	N	VR3_Dzare	logarithmic Randic-like eigenvector-based index from Barysz matrix weighted by allred-rocow EN
**770**	N	SpAbs_Dzp	graph energy from Barysz matrix weighted by polarizability
**771**	N	SpMax_Dzp	leading eigenvalue from Barysz matrix weighted by polarizability
**772**	N	SpDiam_Dzp	spectral diamiter from Barysz matrix weighted by polarizability
**773**	N	SpAD_Dzp	spectral absolute diviation from Barysz matrix weighted by polarizability
**774**	N	SpMAD_Dzp	spectral mean absolute diviation from Barysz matrix weighted by polarizability
**775**	N	LogEE_Dzp	Estrada-like index from Barysz matrix weighted by polarizability
**776**	N	SM1_Dzp	spectral moment from Barysz matrix weighted by polarizability
**777**	N	VE1_Dzp	coefficient sum of the last eigenvector from Barysz matrix weighted by polarizability
**778**	N	VE2_Dzp	average coefficient of the last eigenvector from Barysz matrix weighted by polarizability
**779**	N	VE3_Dzp	logarithmic coefficient sum of the last eigenvector from Barysz matrix weighted by polarizability
**780**	N	VR1_Dzp	Randic-like eigenvector-based index from Barysz matrix weighted by polarizability
**781**	N	VR2_Dzp	normalized Randic-like eigenvector-based index from Barysz matrix weighted by polarizability
**782**	N	VR3_Dzp	logarithmic Randic-like eigenvector-based index from Barysz matrix weighted by polarizability
**783**	N	SpAbs_Dzi	graph energy from Barysz matrix weighted by ionization potential
**784**	N	SpMax_Dzi	leading eigenvalue from Barysz matrix weighted by ionization potential
**785**	N	SpDiam_Dzi	spectral diamiter from Barysz matrix weighted by ionization potential
**786**	N	SpAD_Dzi	spectral absolute diviation from Barysz matrix weighted by ionization potential
**787**	N	SpMAD_Dzi	spectral mean absolute diviation from Barysz matrix weighted by ionization potential
**788**	N	LogEE_Dzi	Estrada-like index from Barysz matrix weighted by ionization potential
**789**	N	SM1_Dzi	spectral moment from Barysz matrix weighted by ionization potential
**790**	N	VE1_Dzi	coefficient sum of the last eigenvector from Barysz matrix weighted by ionization potential
**791**	N	VE2_Dzi	average coefficient of the last eigenvector from Barysz matrix weighted by ionization potential
**792**	N	VE3_Dzi	logarithmic coefficient sum of the last eigenvector from Barysz matrix weighted by ionization potential
**793**	N	VR1_Dzi	Randic-like eigenvector-based index from Barysz matrix weighted by ionization potential

**794**	N	VR2_Dzi	normalized Randic-like eigenvector-based index from Barysz matrix weighted by ionization potential
**795**	N	VR3_Dzi	logarithmic Randic-like eigenvector-based index from Barysz matrix weighted by ionization potential
**796**	N	BCUTc-1h	first heighest eigenvalue of Burden matrix weighted by gasteiger charge
**797**	N	BCUTc-1l	first lowest eigenvalue of Burden matrix weighted by gasteiger charge
**798**	N	BCUTdv-1h	first heighest eigenvalue of Burden matrix weighted by valence electrons
**799**	N	BCUTdv-1l	first lowest eigenvalue of Burden matrix weighted by valence electrons
**800**	N	BCUTd-1h	first heighest eigenvalue of Burden matrix weighted by sigma electrons
**801**	N	BCUTd-1l	first lowest eigenvalue of Burden matrix weighted by sigma electrons
**802**	N	BCUTs-1h	first heighest eigenvalue of Burden matrix weighted by intrinsic state
**803**	N	BCUTs-1l	first lowest eigenvalue of Burden matrix weighted by intrinsic state
**804**	N	BCUTZ-1h	first heighest eigenvalue of Burden matrix weighted by atomic number
**805**	N	BCUTZ-1l	first lowest eigenvalue of Burden matrix weighted by atomic number
**806**	N	BCUTm-1h	first heighest eigenvalue of Burden matrix weighted by mass
**807**	N	BCUTm-1l	first lowest eigenvalue of Burden matrix weighted by mass
**808**	N	BCUTv-1h	first heighest eigenvalue of Burden matrix weighted by vdw volume
**809**	N	BCUTv-1l	first lowest eigenvalue of Burden matrix weighted by vdw volume
**810**	N	BCUTse-1h	first heighest eigenvalue of Burden matrix weighted by sanderson EN
**811**	N	BCUTse-1l	first lowest eigenvalue of Burden matrix weighted by sanderson EN
**812**	N	BCUTpe-1h	first heighest eigenvalue of Burden matrix weighted by pauling EN
**813**	N	BCUTpe-1l	first lowest eigenvalue of Burden matrix weighted by pauling EN
**814**	N	BCUTare-1h	first heighest eigenvalue of Burden matrix weighted by allred-rocow EN
**815**	N	BCUTare-1l	first lowest eigenvalue of Burden matrix weighted by allred-rocow EN
**816**	N	BCUTp-1h	first heighest eigenvalue of Burden matrix weighted by polarizability
**817**	N	BCUTp-1l	first lowest eigenvalue of Burden matrix weighted by polarizability
**818**	N	BCUTi-1h	first heighest eigenvalue of Burden matrix weighted by ionization potential
**819**	N	BCUTi-1l	first lowest eigenvalue of Burden matrix weighted by ionization potential
**820**	N	BertzCT	Bertz CT
**821**	N	nBonds	number of all bonds in non-kekulized structure
**822**	N	nBondsO	number of bonds connecting to heavy atom in non-kekulized structure
**823**	N	nBondsS	number of single bonds in non-kekulized structure
**824**	N	nBondsD	number of double bonds in non-kekulized structure
**825**	N	nBondsT	number of triple bonds in non-kekulized structure
**826**	N	nBondsA	number of aromatic bonds in non-kekulized structure
**827**	N	nBondsM	number of multiple bonds in non-kekulized structure
**828**	N	nBondsKS	number of single bonds in kekulized structure
**829**	N	nBondsKD	number of double bonds in kekulized structure
**830**	N	C1SP1	SP carbon bound to 1 other carbon
**831**	N	C2SP1	SP carbon bound to 2 other carbons
**832**	N	C1SP2	SP2 carbon bound to 1 other carbon
**833**	N	C2SP2	SP2 carbon bound to 2 other carbons
**834**	N	C3SP2	SP2 carbon bound to 3 other carbons
**835**	N	C1SP3	SP3 carbon bound to 1 other carbon
**836**	N	C2SP3	SP3 carbon bound to 2 other carbons

**837**	N	C3SP3	SP3 carbon bound to 3 other carbons
**838**	N	C4SP3	SP3 carbon bound to 4 other carbons
**839**	N	HybRatio	hybridization ratio
**840**	N	Xch-3d	3-ordered Chi chain weighted by sigma electrons
**841**	N	Xch-4d	4-ordered Chi chain weighted by sigma electrons
**842**	N	Xch-5d	5-ordered Chi chain weighted by sigma electrons
**843**	N	Xch-6d	6-ordered Chi chain weighted by sigma electrons
**844**	N	Xch-7d	7-ordered Chi chain weighted by sigma electrons
**845**	N	Xch-3dv	3-ordered Chi chain weighted by valence electrons
**846**	N	Xch-4dv	4-ordered Chi chain weighted by valence electrons
**847**	N	Xch-5dv	5-ordered Chi chain weighted by valence electrons
**848**	N	Xch-6dv	6-ordered Chi chain weighted by valence electrons
**849**	N	Xch-7dv	7-ordered Chi chain weighted by valence electrons
**850**	N	Xc-3d	3-ordered Chi cluster weighted by sigma electrons
**851**	N	Xc-4d	4-ordered Chi cluster weighted by sigma electrons
**852**	N	Xc-5d	5-ordered Chi cluster weighted by sigma electrons
**853**	N	Xc-6d	6-ordered Chi cluster weighted by sigma electrons
**854**	N	Xc-3dv	3-ordered Chi cluster weighted by valence electrons
**855**	N	Xc-4dv	4-ordered Chi cluster weighted by valence electrons
**856**	N	Xc-5dv	5-ordered Chi cluster weighted by valence electrons
**857**	N	Xc-6dv	6-ordered Chi cluster weighted by valence electrons
**858**	N	Xpc-4d	4-ordered Chi path-cluster weighted by sigma electrons
**859**	N	Xpc-5d	5-ordered Chi path-cluster weighted by sigma electrons
**860**	N	Xpc-6d	6-ordered Chi path-cluster weighted by sigma electrons
**861**	N	Xpc-4dv	4-ordered Chi path-cluster weighted by valence electrons
**862**	N	Xpc-5dv	5-ordered Chi path-cluster weighted by valence electrons
**863**	N	Xpc-6dv	6-ordered Chi path-cluster weighted by valence electrons
**864**	N	Xp-0d	0-ordered Chi path weighted by sigma electrons
**865**	N	Xp-1d	1-ordered Chi path weighted by sigma electrons
**866**	N	Xp-2d	2-ordered Chi path weighted by sigma electrons
**867**	N	Xp-3d	3-ordered Chi path weighted by sigma electrons
**868**	N	Xp-4d	4-ordered Chi path weighted by sigma electrons
**869**	N	Xp-5d	5-ordered Chi path weighted by sigma electrons
**870**	N	Xp-6d	6-ordered Chi path weighted by sigma electrons
**871**	N	Xp-7d	7-ordered Chi path weighted by sigma electrons
**872**	N	AXp-0d	0-ordered averaged Chi path weighted by sigma electrons
**873**	N	AXp-1d	1-ordered averaged Chi path weighted by sigma electrons
**874**	N	AXp-2d	2-ordered averaged Chi path weighted by sigma electrons
**875**	N	AXp-3d	3-ordered averaged Chi path weighted by sigma electrons
**876**	N	AXp-4d	4-ordered averaged Chi path weighted by sigma electrons
**877**	N	AXp-5d	5-ordered averaged Chi path weighted by sigma electrons
**878**	N	AXp-6d	6-ordered averaged Chi path weighted by sigma electrons
**879**	N	AXp-7d	7-ordered averaged Chi path weighted by sigma electrons
**880**	N	Xp-0dv	0-ordered Chi path weighted by valence electrons
**881**	N	Xp-1dv	1-ordered Chi path weighted by valence electrons

**882**	N	Xp-2dv	2-ordered Chi path weighted by valence electrons
**883**	N	Xp-3dv	3-ordered Chi path weighted by valence electrons
**884**	N	Xp-4dv	4-ordered Chi path weighted by valence electrons
**885**	N	Xp-5dv	5-ordered Chi path weighted by valence electrons
**886**	N	Xp-6dv	6-ordered Chi path weighted by valence electrons
**887**	N	Xp-7dv	7-ordered Chi path weighted by valence electrons
**888**	N	AXp-0dv	0-ordered averaged Chi path weighted by valence electrons
**889**	N	AXp-1dv	1-ordered averaged Chi path weighted by valence electrons
**890**	N	AXp-2dv	2-ordered averaged Chi path weighted by valence electrons
**891**	N	AXp-3dv	3-ordered averaged Chi path weighted by valence electrons
**892**	N	AXp-4dv	4-ordered averaged Chi path weighted by valence electrons
**893**	N	AXp-5dv	5-ordered averaged Chi path weighted by valence electrons
**894**	N	AXp-6dv	6-ordered averaged Chi path weighted by valence electrons
**895**	N	AXp-7dv	7-ordered averaged Chi path weighted by valence electrons
**896**	N	SZ	sum of constitutional weighted by atomic number
**897**	N	Sm	sum of constitutional weighted by mass
**898**	N	Sv	sum of constitutional weighted by vdw volume
**899**	N	Sse	sum of constitutional weighted by sanderson EN
**900**	N	Spe	sum of constitutional weighted by pauling EN
**901**	N	Sare	sum of constitutional weighted by allred-rocow EN
**902**	N	Sp	sum of constitutional weighted by polarizability
**903**	N	Si	sum of constitutional weighted by ionization potential
**904**	N	MZ	mean of constitutional weighted by atomic number
**905**	N	Mm	mean of constitutional weighted by mass
**906**	N	Mv	mean of constitutional weighted by vdw volume
**907**	N	Mse	mean of constitutional weighted by sanderson EN
**908**	N	Mpe	mean of constitutional weighted by pauling EN
**909**	N	Mare	mean of constitutional weighted by allred-rocow EN
**910**	N	Mp	mean of constitutional weighted by polarizability
**911**	N	Mi	mean of constitutional weighted by ionization potential
**912**	N	RNCG	relative negative charge
**913**	N	RPCG	relative positive charge
**914**	N	SpAbs_Dt	graph energy from detourn matrix
**915**	N	SpMax_Dt	leading eigenvalue from detourn matrix
**916**	N	SpDiam_Dt	spectral diamiter from detourn matrix
**917**	N	SpAD_Dt	spectral absolute diviation from detourn matrix
**918**	N	SpMAD_Dt	spectral mean absolute diviation from detourn matrix
**919**	N	LogEE_Dt	Estrada-like index from detourn matrix
**920**	N	SM1_Dt	spectral moment from detourn matrix
**921**	N	VE1_Dt	coefficient sum of the last eigenvector from detourn matrix
**922**	N	VE2_Dt	average coefficient of the last eigenvector from detourn matrix
**923**	N	VE3_Dt	logarithmic coefficient sum of the last eigenvector from detourn matrix
**924**	N	VR1_Dt	Randic-like eigenvector-based index from detourn matrix
**925**	N	VR2_Dt	normalized Randic-like eigenvector-based index from detourn matrix
**926**	N	VR3_Dt	logarithmic Randic-like eigenvector-based index from detourn matrix

**927**	N	DetourIndex	detour index
**928**	N	SpAbs_D	graph energy from distance matrix
**929**	N	SpMax_D	leading eigenvalue from distance matrix
**930**	N	SpDiam_D	spectral diamiter from distance matrix
**931**	N	SpAD_D	spectral absolute diviation from distance matrix
**932**	N	SpMAD_D	spectral mean absolute diviation from distance matrix
**933**	N	LogEE_D	Estrada-like index from distance matrix
**934**	N	SM1_D	spectral moment from distance matrix
**935**	N	VE1_D	coefficient sum of the last eigenvector from distance matrix
**936**	N	VE2_D	average coefficient of the last eigenvector from distance matrix
**937**	N	VE3_D	logarithmic coefficient sum of the last eigenvector from distance matrix
**938**	N	VR1_D	Randic-like eigenvector-based index from distance matrix
**939**	N	VR2_D	normalized Randic-like eigenvector-based index from distance matrix
**940**	N	VR3_D	logarithmic Randic-like eigenvector-based index from distance matrix
**941**	N	ECIndex	eccentric connectivity index
**942**	N	NsLi	number of sLi
**943**	N	NssBe	number of ssBe
**944**	N	NssssBe	number of ssssBe
**945**	N	NssBH	number of ssBH
**946**	N	NsssB	number of sssB
**947**	N	NssssB	number of ssssB
**948**	N	NsCH3	number of sCH3
**949**	N	NdCH2	number of dCH2
**950**	N	NssCH2	number of ssCH2
**951**	N	NtCH	number of tCH
**952**	N	NdsCH	number of dsCH
**953**	N	NaaCH	number of aaCH
**954**	N	NsssCH	number of sssCH
**955**	N	NddC	number of ddC
**956**	N	NtsC	number of tsC
**957**	N	NdssC	number of dssC
**958**	N	NaasC	number of aasC
**959**	N	NaaaC	number of aaaC
**960**	N	NssssC	number of ssssC
**961**	N	NsNH3	number of sNH3
**962**	N	NsNH2	number of sNH2
**963**	N	NssNH2	number of ssNH2
**964**	N	NdNH	number of dNH
**965**	N	NssNH	number of ssNH
**966**	N	NaaNH	number of aaNH
**967**	N	NtN	number of tN
**968**	N	NsssNH	number of sssNH
**969**	N	NdsN	number of dsN
**970**	N	NaaN	number of aaN
**971**	N	NsssN	number of sssN

**972**	N	NddsN	number of ddsN
**973**	N	NaasN	number of aasN
**974**	N	NssssN	number of ssssN
**975**	N	NsOH	number of sOH
**976**	N	NdO	number of dO
**977**	N	NssO	number of ssO
**978**	N	NaaO	number of aaO
**979**	N	NsF	number of sF
**980**	N	NsSiH3	number of sSiH3
**981**	N	NssSiH2	number of ssSiH2
**982**	N	NsssSiH	number of sssSiH
**983**	N	NssssSi	number of ssssSi
**984**	N	NsPH2	number of sPH2
**985**	N	NssPH	number of ssPH
**986**	N	NsssP	number of sssP
**987**	N	NdsssP	number of dsssP
**988**	N	NsssssP	number of sssssP
**989**	N	NsSH	number of sSH
**990**	N	NdS	number of dS
**991**	N	NssS	number of ssS
**992**	N	NaaS	number of aaS
**993**	N	NdssS	number of dssS
**994**	N	NddssS	number of ddssS
**995**	N	NsCl	number of sCl
**996**	N	NsGeH3	number of sGeH3
**997**	N	NssGeH2	number of ssGeH2
**998**	N	NsssGeH	number of sssGeH
**999**	N	NssssGe	number of ssssGe
**1000**	N	NsAsH2	number of sAsH2
**1001**	N	NssAsH	number of ssAsH
**1002**	N	NsssAs	number of sssAs
**1003**	N	NsssdAs	number of sssdAs
**1004**	N	NsssssAs	number of sssssAs
**1005**	N	NsSeH	number of sSeH
**1006**	N	NdSe	number of dSe
**1007**	N	NssSe	number of ssSe
**1008**	N	NaaSe	number of aaSe
**1009**	N	NdssSe	number of dssSe
**1010**	N	NddssSe	number of ddssSe
**1011**	N	NsBr	number of sBr
**1012**	N	NsSnH3	number of sSnH3
**1013**	N	NssSnH2	number of ssSnH2
**1014**	N	NsssSnH	number of sssSnH
**1015**	N	NssssSn	number of ssssSn
**1016**	N	NsI	number of sI

**1017**	N	NsPbH3	number of sPbH3
**1018**	N	NssPbH2	number of ssPbH2
**1019**	N	NsssPbH	number of sssPbH
**1020**	N	NssssPb	number of ssssPb
**1021**	N	SsLi	sum of sLi
**1022**	N	SssBe	sum of ssBe
**1023**	N	SssssBe	sum of ssssBe
**1024**	N	SssBH	sum of ssBH
**1025**	N	SsssB	sum of sssB
**1026**	N	SssssB	sum of ssssB
**1027**	N	SsCH3	sum of sCH3
**1028**	N	SdCH2	sum of dCH2
**1029**	N	SssCH2	sum of ssCH2
**1030**	N	StCH	sum of tCH
**1031**	N	SdsCH	sum of dsCH
**1032**	N	SaaCH	sum of aaCH
**1033**	N	SsssCH	sum of sssCH
**1034**	N	SddC	sum of ddC
**1035**	N	StsC	sum of tsC
**1036**	N	SdssC	sum of dssC
**1037**	N	SaasC	sum of aasC
**1038**	N	SaaaC	sum of aaaC
**1039**	N	SssssC	sum of ssssC
**1040**	N	SsNH3	sum of sNH3
**1041**	N	SsNH2	sum of sNH2
**1042**	N	SssNH2	sum of ssNH2
**1043**	N	SdNH	sum of dNH
**1044**	N	SssNH	sum of ssNH
**1045**	N	SaaNH	sum of aaNH
**1046**	N	StN	sum of tN
**1047**	N	SsssNH	sum of sssNH
**1048**	N	SdsN	sum of dsN
**1049**	N	SaaN	sum of aaN
**1050**	N	SsssN	sum of sssN
**1051**	N	SddsN	sum of ddsN
**1052**	N	SaasN	sum of aasN
**1053**	N	SssssN	sum of ssssN
**1054**	N	SsOH	sum of sOH
**1055**	N	SdO	sum of dO
**1056**	N	SssO	sum of ssO
**1057**	N	SaaO	sum of aaO
**1058**	N	SsF	sum of sF
**1059**	N	SsSiH3	sum of sSiH3
**1060**	N	SssSiH2	sum of ssSiH2
**1061**	N	SsssSiH	sum of sssSiH

**1062**	N	SssssSi	sum of ssssSi
**1063**	N	SsPH2	sum of sPH2
**1064**	N	SssPH	sum of ssPH
**1065**	N	SsssP	sum of sssP
**1066**	N	SdsssP	sum of dsssP
**1067**	N	SsssssP	sum of sssssP
**1068**	N	SsSH	sum of sSH
**1069**	N	SdS	sum of dS
**1070**	N	SssS	sum of ssS
**1071**	N	SaaS	sum of aaS
**1072**	N	SdssS	sum of dssS
**1073**	N	SddssS	sum of ddssS
**1074**	N	SsCl	sum of sCl
**1075**	N	SsGeH3	sum of sGeH3
**1076**	N	SssGeH2	sum of ssGeH2
**1077**	N	SsssGeH	sum of sssGeH
**1078**	N	SssssGe	sum of ssssGe
**1079**	N	SsAsH2	sum of sAsH2
**1080**	N	SssAsH	sum of ssAsH
**1081**	N	SsssAs	sum of sssAs
**1082**	N	SsssdAs	sum of sssdAs
**1083**	N	SsssssAs	sum of sssssAs
**1084**	N	SsSeH	sum of sSeH
**1085**	N	SdSe	sum of dSe
**1086**	N	SssSe	sum of ssSe
**1087**	N	SaaSe	sum of aaSe
**1088**	N	SdssSe	sum of dssSe
**1089**	N	SddssSe	sum of ddssSe
**1090**	N	SsBr	sum of sBr
**1091**	N	SsSnH3	sum of sSnH3
**1092**	N	SssSnH2	sum of ssSnH2
**1093**	N	SsssSnH	sum of sssSnH
**1094**	N	SssssSn	sum of ssssSn
**1095**	N	SsI	sum of sI
**1096**	N	SsPbH3	sum of sPbH3
**1097**	N	SssPbH2	sum of ssPbH2
**1098**	N	SsssPbH	sum of sssPbH
**1099**	N	SssssPb	sum of ssssPb
**1100**	N	MAXsLi	max of sLi
**1101**	N	MAXssBe	max of ssBe
**1102**	N	MAXssssBe	max of ssssBe
**1103**	N	MAXssBH	max of ssBH
**1104**	N	MAXsssB	max of sssB
**1105**	N	MAXssssB	max of ssssB
**1106**	N	MAXsCH3	max of sCH3

**1107**	N	MAXdCH2	max of dCH2
**1108**	N	MAXssCH2	max of ssCH2
**1109**	N	MAXtCH	max of tCH
**1110**	N	MAXdsCH	max of dsCH
**1111**	N	MAXaaCH	max of aaCH
**1112**	N	MAXsssCH	max of sssCH
**1113**	N	MAXddC	max of ddC
**1114**	N	MAXtsC	max of tsC
**1115**	N	MAXdssC	max of dssC
**1116**	N	MAXaasC	max of aasC
**1117**	N	MAXaaaC	max of aaaC
**1118**	N	MAXssssC	max of ssssC
**1119**	N	MAXsNH3	max of sNH3
**1120**	N	MAXsNH2	max of sNH2
**1121**	N	MAXssNH2	max of ssNH2
**1122**	N	MAXdNH	max of dNH
**1123**	N	MAXssNH	max of ssNH
**1124**	N	MAXaaNH	max of aaNH
**1125**	N	MAXtN	max of tN
**1126**	N	MAXsssNH	max of sssNH
**1127**	N	MAXdsN	max of dsN
**1128**	N	MAXaaN	max of aaN
**1129**	N	MAXsssN	max of sssN
**1130**	N	MAXddsN	max of ddsN
**1131**	N	MAXaasN	max of aasN
**1132**	N	MAXssssN	max of ssssN
**1133**	N	MAXsOH	max of sOH
**1134**	N	MAXdO	max of dO
**1135**	N	MAXssO	max of ssO
**1136**	N	MAXaaO	max of aaO
**1137**	N	MAXsF	max of sF
**1138**	N	MAXsSiH3	max of sSiH3
**1139**	N	MAXssSiH2	max of ssSiH2
**1140**	N	MAXsssSiH	max of sssSiH
**1141**	N	MAXssssSi	max of ssssSi
**1142**	N	MAXsPH2	max of sPH2
**1143**	N	MAXssPH	max of ssPH
**1144**	N	MAXsssP	max of sssP
**1145**	N	MAXdsssP	max of dsssP
**1146**	N	MAXsssssP	max of sssssP
**1147**	N	MAXsSH	max of sSH
**1148**	N	MAXdS	max of dS
**1149**	N	MAXssS	max of ssS
**1150**	N	MAXaaS	max of aaS
**1151**	N	MAXdssS	max of dssS

**1152**	N	MAXddssS	max of ddssS
**1153**	N	MAXsCl	max of sCl
**1154**	N	MAXsGeH3	max of sGeH3
**1155**	N	MAXssGeH2	max of ssGeH2
**1156**	N	MAXsssGeH	max of sssGeH
**1157**	N	MAXssssGe	max of ssssGe
**1158**	N	MAXsAsH2	max of sAsH2
**1159**	N	MAXssAsH	max of ssAsH
**1160**	N	MAXsssAs	max of sssAs
**1161**	N	MAXsssdAs	max of sssdAs
**1162**	N	MAXsssssAs	max of sssssAs
**1163**	N	MAXsSeH	max of sSeH
**1164**	N	MAXdSe	max of dSe
**1165**	N	MAXssSe	max of ssSe
**1166**	N	MAXaaSe	max of aaSe
**1167**	N	MAXdssSe	max of dssSe
**1168**	N	MAXddssSe	max of ddssSe
**1169**	N	MAXsBr	max of sBr
**1170**	N	MAXsSnH3	max of sSnH3
**1171**	N	MAXssSnH2	max of ssSnH2
**1172**	N	MAXsssSnH	max of sssSnH
**1173**	N	MAXssssSn	max of ssssSn
**1174**	N	MAXsI	max of sI
**1175**	N	MAXsPbH3	max of sPbH3
**1176**	N	MAXssPbH2	max of ssPbH2
**1177**	N	MAXsssPbH	max of sssPbH
**1178**	N	MAXssssPb	max of ssssPb
**1179**	N	MINsLi	min of sLi
**1180**	N	MINssBe	min of ssBe
**1181**	N	MINssssBe	min of ssssBe
**1182**	N	MINssBH	min of ssBH
**1183**	N	MINsssB	min of sssB
**1184**	N	MINssssB	min of ssssB
**1185**	N	MINsCH3	min of sCH3
**1186**	N	MINdCH2	min of dCH2
**1187**	N	MINssCH2	min of ssCH2
**1188**	N	MINtCH	min of tCH
**1189**	N	MINdsCH	min of dsCH
**1190**	N	MINaaCH	min of aaCH
**1191**	N	MINsssCH	min of sssCH
**1192**	N	MINddC	min of ddC
**1193**	N	MINtsC	min of tsC
**1194**	N	MINdssC	min of dssC
**1195**	N	MINaasC	min of aasC
**1196**	N	MINaaaC	min of aaaC

**1197**	N	MINssssC	min of ssssC
**1198**	N	MINsNH3	min of sNH3
**1199**	N	MINsNH2	min of sNH2
**1200**	N	MINssNH2	min of ssNH2
**1201**	N	MINdNH	min of dNH
**1202**	N	MINssNH	min of ssNH
**1203**	N	MINaaNH	min of aaNH
**1204**	N	MINtN	min of tN
**1205**	N	MINsssNH	min of sssNH
**1206**	N	MINdsN	min of dsN
**1207**	N	MINaaN	min of aaN
**1208**	N	MINsssN	min of sssN
**1209**	N	MINddsN	min of ddsN
**1210**	N	MINaasN	min of aasN
**1211**	N	MINssssN	min of ssssN
**1212**	N	MINsOH	min of sOH
**1213**	N	MINdO	min of dO
**1214**	N	MINssO	min of ssO
**1215**	N	MINaaO	min of aaO
**1216**	N	MINsF	min of sF
**1217**	N	MINsSiH3	min of sSiH3
**1218**	N	MINssSiH2	min of ssSiH2
**1219**	N	MINsssSiH	min of sssSiH
**1220**	N	MINssssSi	min of ssssSi
**1221**	N	MINsPH2	min of sPH2
**1222**	N	MINssPH	min of ssPH
**1223**	N	MINsssP	min of sssP
**1224**	N	MINdsssP	min of dsssP
**1225**	N	MINsssssP	min of sssssP
**1226**	N	MINsSH	min of sSH
**1227**	N	MINdS	min of dS
**1228**	N	MINssS	min of ssS
**1229**	N	MINaaS	min of aaS
**1230**	N	MINdssS	min of dssS
**1231**	N	MINddssS	min of ddssS
**1232**	N	MINsCl	min of sCl
**1233**	N	MINsGeH3	min of sGeH3
**1234**	N	MINssGeH2	min of ssGeH2
**1235**	N	MINsssGeH	min of sssGeH
**1236**	N	MINssssGe	min of ssssGe
**1237**	N	MINsAsH2	min of sAsH2
**1238**	N	MINssAsH	min of ssAsH
**1239**	N	MINsssAs	min of sssAs
**1240**	N	MINsssdAs	min of sssdAs
**1241**	N	MINsssssAs	min of sssssAs

**1242**	N	MINsSeH	min of sSeH
**1243**	N	MINdSe	min of dSe
**1244**	N	MINssSe	min of ssSe
**1245**	N	MINaaSe	min of aaSe
**1246**	N	MINdssSe	min of dssSe
**1247**	N	MINddssSe	min of ddssSe
**1248**	N	MINsBr	min of sBr
**1249**	N	MINsSnH3	min of sSnH3
**1250**	N	MINssSnH2	min of ssSnH2
**1251**	N	MINsssSnH	min of sssSnH
**1252**	N	MINssssSn	min of ssssSn
**1253**	N	MINsI	min of sI
**1254**	N	MINsPbH3	min of sPbH3
**1255**	N	MINssPbH2	min of ssPbH2
**1256**	N	MINsssPbH	min of sssPbH
**1257**	N	MINssssPb	min of ssssPb
**1258**	N	ETA_alpha	ETA core count
**1259**	N	AETA_alpha	averaged ETA core count
**1260**	N	ETA_shape_p	ETA shape index (type: p)
**1261**	N	ETA_shape_y	ETA shape index (type: y)
**1262**	N	ETA_shape_x	ETA shape index (type: x)
**1263**	N	ETA_beta	valence electron mobile count
**1264**	N	AETA_beta	averaged valence electron mobile count
**1265**	N	ETA_beta_s	sigma contribution to valence electron mobile count
**1266**	N	AETA_beta_s	averaged sigma contribution to valence electron mobile count
**1267**	N	ETA_beta_ns	nonsigma contribution to valence electron mobile count
**1268**	N	AETA_beta_ns	averaged nonsigma contribution to valence electron mobile count
**1269**	N	ETA_beta_ns_d	delta contribution to valence electron mobile count
**1270**	N	AETA_beta_ns_d	averaged delta contribution to valence electron mobile count
**1271**	N	ETA_eta	ETA composite index for reference graph
**1272**	N	AETA_eta	averaged ETA composite index for reference graph
**1273**	N	ETA_eta_L	local ETA composite index for reference graph
**1274**	N	AETA_eta_L	averaged local ETA composite index for reference graph
**1275**	N	ETA_eta_R	ETA composite index for reference graph
**1276**	N	AETA_eta_R	averaged ETA composite index for reference graph
**1277**	N	ETA_eta_RL	local ETA composite index for reference graph
**1278**	N	AETA_eta_RL	averaged local ETA composite index for reference graph
**1279**	N	ETA_eta_F	ETA functionality index
**1280**	N	AETA_eta_F	averaged ETA functionality index
**1281**	N	ETA_eta_FL	local ETA functionality index
**1282**	N	AETA_eta_FL	averaged local ETA functionality index
**1283**	N	ETA_eta_B	ETA branching index
**1284**	N	AETA_eta_B	averaged ETA branching index
**1285**	N	ETA_eta_BR	ETA branching index (use ring count)

**1286**	N	AETA_eta_BR	averaged ETA branching index (use ring count)
**1287**	N	ETA_dAlpha_A	ETA delta alpha (type: A)
**1288**	N	ETA_dAlpha_B	ETA delta alpha (type: B)
**1289**	N	ETA_epsilon_1	ETA epsilon (type: 1)
**1290**	N	ETA_epsilon_2	ETA epsilon (type: 2)
**1291**	N	ETA_epsilon_3	ETA epsilon (type: 3)
**1292**	N	ETA_epsilon_4	ETA epsilon (type: 4)
**1293**	N	ETA_epsilon_5	ETA epsilon (type: 5)
**1294**	N	ETA_dEpsilon_A	ETA delta epsilon (type: A)
**1295**	N	ETA_dEpsilon_B	ETA delta epsilon (type: B)
**1296**	N	ETA_dEpsilon_C	ETA delta epsilon (type: C)
**1297**	N	ETA_dEpsilon_D	ETA delta epsilon (type: D)
**1298**	N	ETA_dBeta	ETA delta beta
**1299**	N	AETA_dBeta	averaged ETA delta beta
**1300**	N	ETA_psi_1	ETA psi
**1301**	N	ETA_dPsi_A	ETA delta psi (type: A)
**1302**	N	ETA_dPsi_B	ETA delta psi (type: B)
**1303**	N	fragCpx	fragment complexity
**1304**	N	fMF	molecular framework ratio
**1305**	N	nHBAcc	number of hydrogen bond acceptor
**1306**	N	nHBDon	number of hydrogen bond donor
**1307**	N	IC0	0-ordered neighborhood information content
**1308**	N	IC1	1-ordered neighborhood information content
**1309**	N	IC2	2-ordered neighborhood information content
**1310**	N	IC3	3-ordered neighborhood information content
**1311**	N	IC4	4-ordered neighborhood information content
**1312**	N	IC5	5-ordered neighborhood information content
**1313**	N	TIC0	0-ordered neighborhood total information content
**1314**	N	TIC1	1-ordered neighborhood total information content
**1315**	N	TIC2	2-ordered neighborhood total information content
**1316**	N	TIC3	3-ordered neighborhood total information content
**1317**	N	TIC4	4-ordered neighborhood total information content
**1318**	N	TIC5	5-ordered neighborhood total information content
**1319**	N	SIC0	0-ordered structural information content
**1320**	N	SIC1	1-ordered structural information content
**1321**	N	SIC2	2-ordered structural information content
**1322**	N	SIC3	3-ordered structural information content
**1323**	N	SIC4	4-ordered structural information content
**1324**	N	SIC5	5-ordered structural information content
**1325**	N	BIC0	0-ordered bonding information content
**1326**	N	BIC1	1-ordered bonding information content
**1327**	N	BIC2	2-ordered bonding information content
**1328**	N	BIC3	3-ordered bonding information content
**1329**	N	BIC4	4-ordered bonding information content
**1330**	N	BIC5	5-ordered bonding information content

**1331**	N	CIC0	0-ordered complementary information content
**1332**	N	CIC1	1-ordered complementary information content
**1333**	N	CIC2	2-ordered complementary information content
**1334**	N	CIC3	3-ordered complementary information content
**1335**	N	CIC4	4-ordered complementary information content
**1336**	N	CIC5	5-ordered complementary information content
**1337**	N	MIC0	0-ordered modified information content
**1338**	N	MIC1	1-ordered modified information content
**1339**	N	MIC2	2-ordered modified information content
**1340**	N	MIC3	3-ordered modified information content
**1341**	N	MIC4	4-ordered modified information content
**1342**	N	MIC5	5-ordered modified information content
**1343**	N	ZMIC0	0-ordered Z-modified information content
**1344**	N	ZMIC1	1-ordered Z-modified information content
**1345**	N	ZMIC2	2-ordered Z-modified information content
**1346**	N	ZMIC3	3-ordered Z-modified information content
**1347**	N	ZMIC4	4-ordered Z-modified information content
**1348**	N	ZMIC5	5-ordered Z-modified information content
**1349**	N	Kier1	kappa shape index 1
**1350**	N	Kier2	kappa shape index 2
**1351**	N	Kier3	kappa shape index 3
**1352**	N	Lipinski	Lipinski rule of five
**1353**	N	GhoseFilter	Ghose filter
**1354**	N	VMcGowan	McGowan volume
**1355**	N	LabuteASA	Labute's Approximate Surface Area
**1356**	N	PEOE_VSA1	MOE Charge VSA Descriptor 1 (-inf < x < -0.30)
**1357**	N	PEOE_VSA2	MOE Charge VSA Descriptor 2 (-0.30 <= x < -0.25)
**1358**	N	PEOE_VSA3	MOE Charge VSA Descriptor 3 (-0.25 <= x < -0.20)
**1359**	N	PEOE_VSA4	MOE Charge VSA Descriptor 4 (-0.20 <= x < -0.15)
**1360**	N	PEOE_VSA5	MOE Charge VSA Descriptor 5 (-0.15 <= x < -0.10)
**1361**	N	PEOE_VSA6	MOE Charge VSA Descriptor 6 (-0.10 <= x < -0.05)
**1362**	N	PEOE_VSA7	MOE Charge VSA Descriptor 7 (-0.05 <= x < 0.00)
**1363**	N	PEOE_VSA8	MOE Charge VSA Descriptor 8 (0.00 <= x < 0.05)
**1364**	N	PEOE_VSA9	MOE Charge VSA Descriptor 9 (0.05 <= x < 0.10)
**1365**	N	PEOE_VSA10	MOE Charge VSA Descriptor 10 (0.10 <= x < 0.15)
**1366**	N	PEOE_VSA11	MOE Charge VSA Descriptor 11 (0.15 <= x < 0.20)
**1367**	N	PEOE_VSA12	MOE Charge VSA Descriptor 12 (0.20 <= x < 0.25)
**1368**	N	PEOE_VSA13	MOE Charge VSA Descriptor 13 (0.25 <= x < 0.30)
**1369**	N	SMR_VSA1	MOE MR VSA Descriptor 1 (-inf < x < 1.29)
**1370**	N	SMR_VSA2	MOE MR VSA Descriptor 2 (1.29 <= x < 1.82)
**1371**	N	SMR_VSA3	MOE MR VSA Descriptor 3 (1.82 <= x < 2.24)
**1372**	N	SMR_VSA4	MOE MR VSA Descriptor 4 (2.24 <= x < 2.45)
**1373**	N	SMR_VSA5	MOE MR VSA Descriptor 5 (2.45 <= x < 2.75)
**1374**	N	SMR_VSA6	MOE MR VSA Descriptor 6 (2.75 <= x < 3.05)
**1375**	N	SMR_VSA7	MOE MR VSA Descriptor 7 (3.05 <= x < 3.63)

**1376**	N	SMR_VSA8	MOE MR VSA Descriptor 8 (3.63 <= x < 3.80)
**1377**	N	SMR_VSA9	MOE MR VSA Descriptor 9 (3.80 <= x < 4.00)
**1378**	N	SlogP_VSA1	MOE logP VSA Descriptor 1 (-inf < x < -0.40)
**1379**	N	SlogP_VSA2	MOE logP VSA Descriptor 2 (-0.40 <= x < -0.20)
**1380**	N	SlogP_VSA3	MOE logP VSA Descriptor 3 (-0.20 <= x < 0.00)
**1381**	N	SlogP_VSA4	MOE logP VSA Descriptor 4 (0.00 <= x < 0.10)
**1382**	N	SlogP_VSA5	MOE logP VSA Descriptor 5 (0.10 <= x < 0.15)
**1383**	N	SlogP_VSA6	MOE logP VSA Descriptor 6 (0.15 <= x < 0.20)
**1384**	N	SlogP_VSA7	MOE logP VSA Descriptor 7 (0.20 <= x < 0.25)
**1385**	N	SlogP_VSA8	MOE logP VSA Descriptor 8 (0.25 <= x < 0.30)
**1386**	N	SlogP_VSA9	MOE logP VSA Descriptor 9 (0.30 <= x < 0.40)
**1387**	N	SlogP_VSA10	MOE logP VSA Descriptor 10 (0.40 <= x < 0.50)
**1388**	N	SlogP_VSA11	MOE logP VSA Descriptor 11 (0.50 <= x < 0.60)
**1389**	N	EState_VSA1	EState VSA Descriptor 1 (-inf < x < -0.39)
**1390**	N	EState_VSA2	EState VSA Descriptor 2 (-0.39 <= x < 0.29)
**1391**	N	EState_VSA3	EState VSA Descriptor 3 (0.29 <= x < 0.72)
**1392**	N	EState_VSA4	EState VSA Descriptor 4 (0.72 <= x < 1.17)
**1393**	N	EState_VSA5	EState VSA Descriptor 5 (1.17 <= x < 1.54)
**1394**	N	EState_VSA6	EState VSA Descriptor 6 (1.54 <= x < 1.81)
**1395**	N	EState_VSA7	EState VSA Descriptor 7 (1.81 <= x < 2.05)
**1396**	N	EState_VSA8	EState VSA Descriptor 8 (2.05 <= x < 4.69)
**1397**	N	EState_VSA9	EState VSA Descriptor 9 (4.69 <= x < 9.17)
**1398**	N	EState_VSA10	EState VSA Descriptor 10 (9.17 <= x < 15.00)
**1399**	N	VSA_EState1	VSA EState Descriptor 1 (-inf < x < 4.78)
**1400**	N	VSA_EState2	VSA EState Descriptor 2 (4.78 <= x < 5.00)
**1401**	N	VSA_EState3	VSA EState Descriptor 3 (5.00 <= x < 5.41)
**1402**	N	VSA_EState4	VSA EState Descriptor 4 (5.41 <= x < 5.74)
**1403**	N	VSA_EState5	VSA EState Descriptor 5 (5.74 <= x < 6.00)
**1404**	N	VSA_EState6	VSA EState Descriptor 6 (6.00 <= x < 6.07)
**1405**	N	VSA_EState7	VSA EState Descriptor 7 (6.07 <= x < 6.45)
**1406**	N	VSA_EState8	VSA EState Descriptor 8 (6.45 <= x < 7.00)
**1407**	N	VSA_EState9	VSA EState Descriptor 9 (7.00 <= x < 11.00)
**1408**	N	MDEC-11	molecular distance edge between primary C and primary C
**1409**	N	MDEC-12	molecular distance edge between primary C and secondary C
**1410**	N	MDEC-13	molecular distance edge between primary C and tertiary C
**1411**	N	MDEC-14	molecular distance edge between primary C and quaternary C
**1412**	N	MDEC-22	molecular distance edge between secondary C and secondary C
**1413**	N	MDEC-23	molecular distance edge between secondary C and tertiary C
**1414**	N	MDEC-24	molecular distance edge between secondary C and quaternary C
**1415**	N	MDEC-33	molecular distance edge between tertiary C and tertiary C
**1416**	N	MDEC-34	molecular distance edge between tertiary C and quaternary C
**1417**	N	MDEC-44	molecular distance edge between quaternary C and quaternary C
**1418**	N	MDEO-11	molecular distance edge between primary O and primary O
**1419**	N	MDEO-12	molecular distance edge between primary O and secondary O
**1420**	N	MDEO-22	molecular distance edge between secondary O and secondary O

**1421**	N	MDEN-11	molecular distance edge between primary N and primary N
**1422**	N	MDEN-12	molecular distance edge between primary N and secondary N
**1423**	N	MDEN-13	molecular distance edge between primary N and tertiary N
**1424**	N	MDEN-22	molecular distance edge between secondary N and secondary N
**1425**	N	MDEN-23	molecular distance edge between secondary N and tertiary N
**1426**	N	MDEN-33	molecular distance edge between tertiary N and tertiary N
**1427**	N	MID	molecular ID
**1428**	N	AMID	averaged molecular ID
**1429**	N	MID_h	molecular ID on h atoms
**1430**	N	AMID_h	averaged molecular ID on h atoms
**1431**	N	MID_C	molecular ID on C atoms
**1432**	N	AMID_C	averaged molecular ID on C atoms
**1433**	N	MID_N	molecular ID on N atoms
**1434**	N	AMID_N	averaged molecular ID on N atoms
**1435**	N	MID_O	molecular ID on O atoms
**1436**	N	AMID_O	averaged molecular ID on O atoms
**1437**	N	MID_X	molecular ID on halogen atoms
**1438**	N	AMID_X	averaged molecular ID on halogen atoms
**1439**	N	MPC2	2-ordered path count
**1440**	N	MPC3	3-ordered path count
**1441**	N	MPC4	4-ordered path count
**1442**	N	MPC5	5-ordered path count
**1443**	N	MPC6	6-ordered path count
**1444**	N	MPC7	7-ordered path count
**1445**	N	MPC8	8-ordered path count
**1446**	N	MPC9	9-ordered path count
**1447**	N	MPC10	10-ordered path count
**1448**	N	TMPC10	10-ordered total path count
**1449**	N	piPC1	1-ordered pi-path count (log scale)
**1450**	N	piPC2	2-ordered pi-path count (log scale)
**1451**	N	piPC3	3-ordered pi-path count (log scale)
**1452**	N	piPC4	4-ordered pi-path count (log scale)
**1453**	N	piPC5	5-ordered pi-path count (log scale)
**1454**	N	piPC6	6-ordered pi-path count (log scale)
**1455**	N	piPC7	7-ordered pi-path count (log scale)
**1456**	N	piPC8	8-ordered pi-path count (log scale)
**1457**	N	piPC9	9-ordered pi-path count (log scale)
**1458**	N	piPC10	10-ordered pi-path count (log scale)
**1459**	N	TpiPC10	10-ordered total pi-path count (log scale)
**1460**	N	apol	atomic polarizability
**1461**	N	bpol	bond polarizability
**1462**	N	nRing	ring count
**1463**	N	n3Ring	3-membered ring count
**1464**	N	n4Ring	4-membered ring count
**1465**	N	n5Ring	5-membered ring count

**1466**	N	n6Ring	6-membered ring count
**1467**	N	n7Ring	7-membered ring count
**1468**	N	n8Ring	8-membered ring count
**1469**	N	n9Ring	9-membered ring count
**1470**	N	n10Ring	10-membered ring count
**1471**	N	n11Ring	11-membered ring count
**1472**	N	n12Ring	12-membered ring count
**1473**	N	nG12Ring	12-or-greater-membered ring count
**1474**	N	nHRing	hetero ring count
**1475**	N	n3HRing	3-membered hetero ring count
**1476**	N	n4HRing	4-membered hetero ring count
**1477**	N	n5HRing	5-membered hetero ring count
**1478**	N	n6HRing	6-membered hetero ring count
**1479**	N	n7HRing	7-membered hetero ring count
**1480**	N	n8HRing	8-membered hetero ring count
**1481**	N	n9HRing	9-membered hetero ring count
**1482**	N	n10HRing	10-membered hetero ring count
**1483**	N	n11HRing	11-membered hetero ring count
**1484**	N	n12HRing	12-membered hetero ring count
**1485**	N	nG12HRing	12-or-greater-membered hetero ring count
**1486**	N	naRing	aromatic ring count
**1487**	N	n3aRing	3-membered aromatic ring count
**1488**	N	n4aRing	4-membered aromatic ring count
**1489**	N	n5aRing	5-membered aromatic ring count
**1490**	N	n6aRing	6-membered aromatic ring count
**1491**	N	n7aRing	7-membered aromatic ring count
**1492**	N	n8aRing	8-membered aromatic ring count
**1493**	N	n9aRing	9-membered aromatic ring count
**1494**	N	n10aRing	10-membered aromatic ring count
**1495**	N	n11aRing	11-membered aromatic ring count
**1496**	N	n12aRing	12-membered aromatic ring count
**1497**	N	nG12aRing	12-or-greater-membered aromatic ring count
**1498**	N	naHRing	aromatic hetero ring count
**1499**	N	n3aHRing	3-membered aromatic hetero ring count
**1500**	N	n4aHRing	4-membered aromatic hetero ring count
**1501**	N	n5aHRing	5-membered aromatic hetero ring count
**1502**	N	n6aHRing	6-membered aromatic hetero ring count
**1503**	N	n7aHRing	7-membered aromatic hetero ring count
**1504**	N	n8aHRing	8-membered aromatic hetero ring count
**1505**	N	n9aHRing	9-membered aromatic hetero ring count
**1506**	N	n10aHRing	10-membered aromatic hetero ring count
**1507**	N	n11aHRing	11-membered aromatic hetero ring count
**1508**	N	n12aHRing	12-membered aromatic hetero ring count
**1509**	N	nG12aHRing	12-or-greater-membered aromatic hetero ring count
**1510**	N	nARing	aliphatic ring count

**1511**	N	n3ARing	3-membered aliphatic ring count
**1512**	N	n4ARing	4-membered aliphatic ring count
**1513**	N	n5ARing	5-membered aliphatic ring count
**1514**	N	n6ARing	6-membered aliphatic ring count
**1515**	N	n7ARing	7-membered aliphatic ring count
**1516**	N	n8ARing	8-membered aliphatic ring count
**1517**	N	n9ARing	9-membered aliphatic ring count
**1518**	N	n10ARing	10-membered aliphatic ring count
**1519**	N	n11ARing	11-membered aliphatic ring count
**1520**	N	n12ARing	12-membered aliphatic ring count
**1521**	N	nG12ARing	12-or-greater-membered aliphatic ring count
**1522**	N	nAHRing	aliphatic hetero ring count
**1523**	N	n3AHRing	3-membered aliphatic hetero ring count
**1524**	N	n4AHRing	4-membered aliphatic hetero ring count
**1525**	N	n5AHRing	5-membered aliphatic hetero ring count
**1526**	N	n6AHRing	6-membered aliphatic hetero ring count
**1527**	N	n7AHRing	7-membered aliphatic hetero ring count
**1528**	N	n8AHRing	8-membered aliphatic hetero ring count
**1529**	N	n9AHRing	9-membered aliphatic hetero ring count
**1530**	N	n10AHRing	10-membered aliphatic hetero ring count
**1531**	N	n11AHRing	11-membered aliphatic hetero ring count
**1532**	N	n12AHRing	12-membered aliphatic hetero ring count
**1533**	N	nG12AHRing	12-or-greater-membered aliphatic hetero ring count
**1534**	N	nFRing	fused ring count
**1535**	N	n4FRing	4-membered fused ring count
**1536**	N	n5FRing	5-membered fused ring count
**1537**	N	n6FRing	6-membered fused ring count
**1538**	N	n7FRing	7-membered fused ring count
**1539**	N	n8FRing	8-membered fused ring count
**1540**	N	n9FRing	9-membered fused ring count
**1541**	N	n10FRing	10-membered fused ring count
**1542**	N	n11FRing	11-membered fused ring count
**1543**	N	n12FRing	12-membered fused ring count
**1544**	N	nG12FRing	12-or-greater-membered fused ring count
**1545**	N	nFHRing	fused hetero ring count
**1546**	N	n4FHRing	4-membered fused hetero ring count
**1547**	N	n5FHRing	5-membered fused hetero ring count
**1548**	N	n6FHRing	6-membered fused hetero ring count
**1549**	N	n7FHRing	7-membered fused hetero ring count
**1550**	N	n8FHRing	8-membered fused hetero ring count
**1551**	N	n9FHRing	9-membered fused hetero ring count
**1552**	N	n10FHRing	10-membered fused hetero ring count
**1553**	N	n11FHRing	11-membered fused hetero ring count
**1554**	N	n12FHRing	12-membered fused hetero ring count
**1555**	N	nG12FHRing	12-or-greater-membered fused hetero ring count

**1556**	N	nFaRing	aromatic fused ring count
**1557**	N	n4FaRing	4-membered aromatic fused ring count
**1558**	N	n5FaRing	5-membered aromatic fused ring count
**1559**	N	n6FaRing	6-membered aromatic fused ring count
**1560**	N	n7FaRing	7-membered aromatic fused ring count
**1561**	N	n8FaRing	8-membered aromatic fused ring count
**1562**	N	n9FaRing	9-membered aromatic fused ring count
**1563**	N	n10FaRing	10-membered aromatic fused ring count
**1564**	N	n11FaRing	11-membered aromatic fused ring count
**1565**	N	n12FaRing	12-membered aromatic fused ring count
**1566**	N	nG12FaRing	12-or-greater-membered aromatic fused ring count
**1567**	N	nFaHRing	aromatic fused hetero ring count
**1568**	N	n4FaHRing	4-membered aromatic fused hetero ring count
**1569**	N	n5FaHRing	5-membered aromatic fused hetero ring count
**1570**	N	n6FaHRing	6-membered aromatic fused hetero ring count
**1571**	N	n7FaHRing	7-membered aromatic fused hetero ring count
**1572**	N	n8FaHRing	8-membered aromatic fused hetero ring count
**1573**	N	n9FaHRing	9-membered aromatic fused hetero ring count
**1574**	N	n10FaHRing	10-membered aromatic fused hetero ring count
**1575**	N	n11FaHRing	11-membered aromatic fused hetero ring count
**1576**	N	n12FaHRing	12-membered aromatic fused hetero ring count
**1577**	N	nG12FaHRing	12-or-greater-membered aromatic fused hetero ring count
**1578**	N	nFARing	aliphatic fused ring count
**1579**	N	n4FARing	4-membered aliphatic fused ring count
**1580**	N	n5FARing	5-membered aliphatic fused ring count
**1581**	N	n6FARing	6-membered aliphatic fused ring count
**1582**	N	n7FARing	7-membered aliphatic fused ring count
**1583**	N	n8FARing	8-membered aliphatic fused ring count
**1584**	N	n9FARing	9-membered aliphatic fused ring count
**1585**	N	n10FARing	10-membered aliphatic fused ring count
**1586**	N	n11FARing	11-membered aliphatic fused ring count
**1587**	N	n12FARing	12-membered aliphatic fused ring count
**1588**	N	nG12FARing	12-or-greater-membered aliphatic fused ring count
**1589**	N	nFAHRing	aliphatic fused hetero ring count
**1590**	N	n4FAHRing	4-membered aliphatic fused hetero ring count
**1591**	N	n5FAHRing	5-membered aliphatic fused hetero ring count
**1592**	N	n6FAHRing	6-membered aliphatic fused hetero ring count
**1593**	N	n7FAHRing	7-membered aliphatic fused hetero ring count
**1594**	N	n8FAHRing	8-membered aliphatic fused hetero ring count
**1595**	N	n9FAHRing	9-membered aliphatic fused hetero ring count
**1596**	N	n10FAHRing	10-membered aliphatic fused hetero ring count
**1597**	N	n11FAHRing	11-membered aliphatic fused hetero ring count
**1598**	N	n12FAHRing	12-membered aliphatic fused hetero ring count
**1599**	N	nG12FAHRing	12-or-greater-membered aliphatic fused hetero ring count
**1600**	N	nRot	rotatable bonds count

**1601**	N	RotRatio	rotatable bonds ratio
**1602**	N	SLogP	Wildman-Crippen LogP
**1603**	N	SMR	Wildman-Crippen MR
**1604**	N	GGI1	1-ordered raw topological charge
**1605**	N	GGI2	2-ordered raw topological charge
**1606**	N	GGI3	3-ordered raw topological charge
**1607**	N	GGI4	4-ordered raw topological charge
**1608**	N	GGI5	5-ordered raw topological charge
**1609**	N	GGI6	6-ordered raw topological charge
**1610**	N	GGI7	7-ordered raw topological charge
**1611**	N	GGI8	8-ordered raw topological charge
**1612**	N	GGI9	9-ordered raw topological charge
**1613**	N	GGI10	10-ordered raw topological charge
**1614**	N	JGI1	1-ordered mean topological charge
**1615**	N	JGI2	2-ordered mean topological charge
**1616**	N	JGI3	3-ordered mean topological charge
**1617**	N	JGI4	4-ordered mean topological charge
**1618**	N	JGI5	5-ordered mean topological charge
**1619**	N	JGI6	6-ordered mean topological charge
**1620**	N	JGI7	7-ordered mean topological charge
**1621**	N	JGI8	8-ordered mean topological charge
**1622**	N	JGI9	9-ordered mean topological charge
**1623**	N	JGI10	10-ordered mean topological charge
**1624**	N	JGT10	10-ordered global topological charge
**1625**	N	Diameter	topological diameter
**1626**	N	Radius	topological radius
**1627**	N	TopoShapeIndex	topological shape index
**1628**	N	PetitjeanIndex	Petitjean index
**1629**	N	TopoPSA(NO)	topological polar surface area (use only nitrogen and oxygen)
**1630**	N	TopoPSA	topological polar surface area
**1631**	N	Vabc	ABC van der waals volume
**1632**	N	VAdjMat	vertex adjacency information
**1633**	N	MWC01	walk count (leg-1)
**1634**	N	MWC02	walk count (leg-2)
**1635**	N	MWC03	walk count (leg-3)
**1636**	N	MWC04	walk count (leg-4)
**1637**	N	MWC05	walk count (leg-5)
**1638**	N	MWC06	walk count (leg-6)
**1639**	N	MWC07	walk count (leg-7)
**1640**	N	MWC08	walk count (leg-8)
**1641**	N	MWC09	walk count (leg-9)
**1642**	N	MWC10	walk count (leg-10)
**1643**	N	TMWC10	total walk count (leg-10)
**1644**	N	SRW02	walk count (leg-2, only self returning walk)
**1645**	N	SRW03	walk count (leg-3, only self returning walk)

**1646**	N	SRW04	walk count (leg-4, only self returning walk)
**1647**	N	SRW05	walk count (leg-5, only self returning walk)
**1648**	N	SRW06	walk count (leg-6, only self returning walk)
**1649**	N	SRW07	walk count (leg-7, only self returning walk)
**1650**	N	SRW08	walk count (leg-8, only self returning walk)
**1651**	N	SRW09	walk count (leg-9, only self returning walk)
**1652**	N	SRW10	walk count (leg-10, only self returning walk)
**1653**	N	TSRW10	total walk count (leg-10, only self returning walk)
**1654**	N	MW	molecular weight
**1655**	N	AMW	averaged molecular weight
**1656**	N	WPath	Wiener index
**1657**	N	WPol	Wiener polarity index
**1658**	N	Zagreb1	Zagreb index (version 1)
**1659**	N	Zagreb2	Zagreb index (version 2)
**1660**	N	mZagreb1	modified Zagreb index (version 1)
**1661**	N	mZagreb2	modified Zagreb index (version 2)

Table S8. Final descriptors after feature selection

Hirshfeld Heavy Atom Charge	Hirshfeld Carbon Charge	Hirshfeld Hydrogen Charge
ESP Heavy Atom Charge	ESP Hydrogen Charge	NPA Hydrogen Charge
Mulliken Heavy Charge	Mulliken Hydrogen Charge	Steric Effect Index
Atomic Polarizability	Distance Degree	Dreiding Energy
AATS2d	AATS1s	AATS2Z
AATS0v	AATS4p	AATS1i
ATSC1d	ATSC2d	AATSC2dv
AATSC4d	AATSC3Z	AATSC5v
AATSC1pe	MATS1c	MATS5s
MATS1p	GATS3c	GATS4c
GATS1v	GATS2pe	BCUTZ-1l
RPCG	FilterItLogS	

2. Random Forest Model Testing and Development

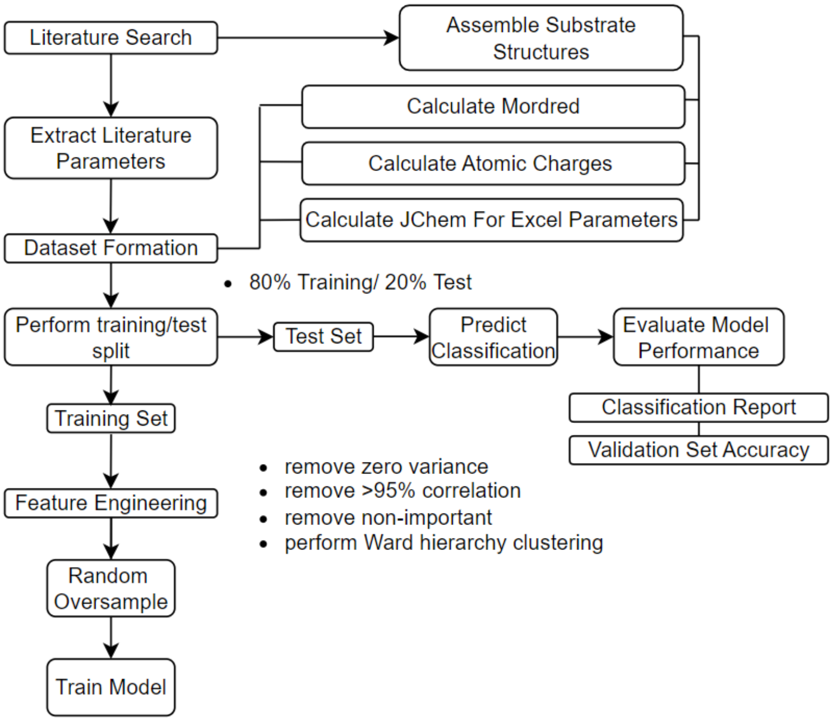

Figure S1. Flowchart of machine learning model operation

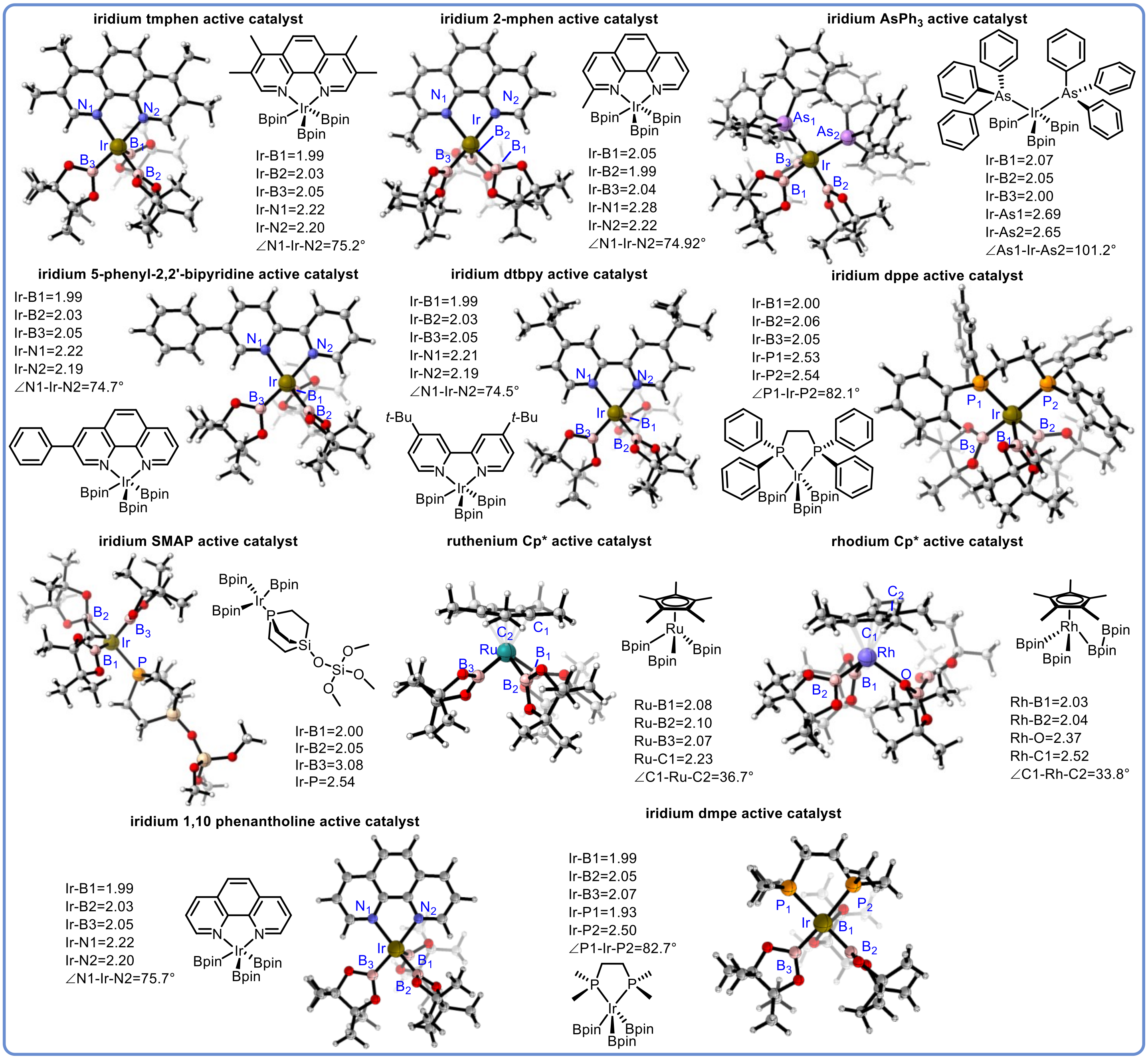

Figure S2. Computed geometries of the active catalyst-ligand combinations at the RB3LYP/LANL2DZ level.

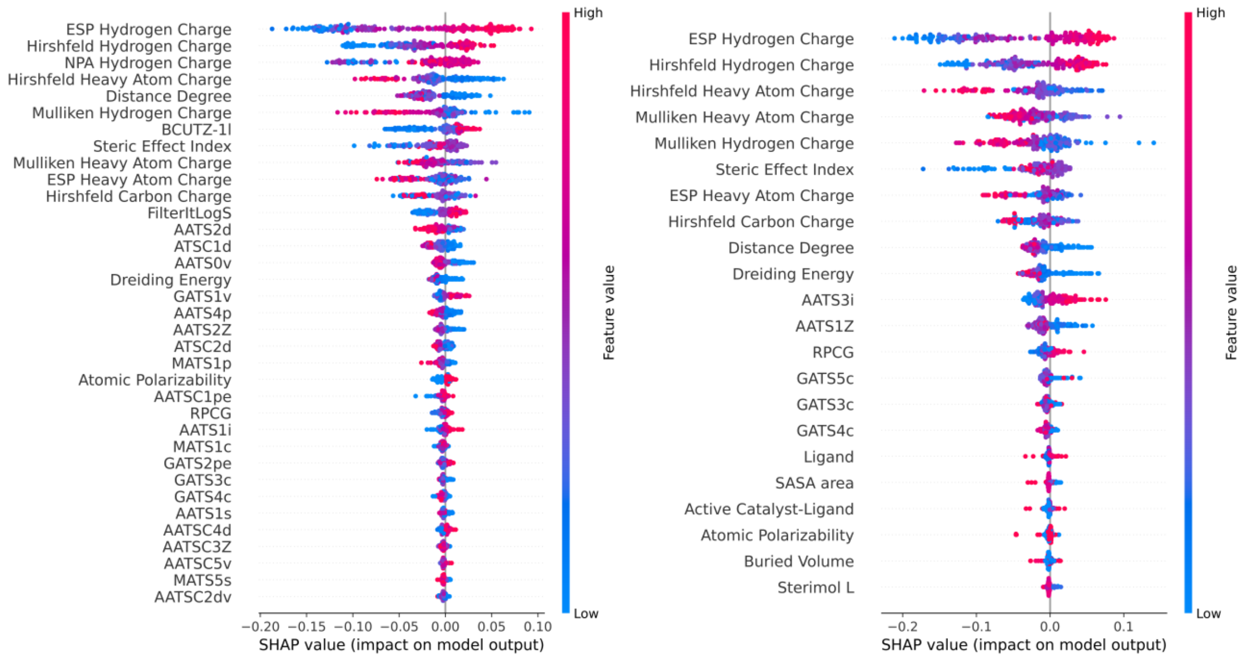

Figure S3. Key features utilized by the developed model, analysed via SHAP analysis. Original dataset (left), ligand dataset (right).

Table S9. Comparison of model accuracy across different model types, with hyperparameters included.

entry	classifier	average accuracy score[a]	hyperparameter
1	Decision Tree	77.2 ± 1.1%	max_depth = 9
2	SVM	81.9 ± 0.0%	kernel = “linear”, C = 1.0
3	Random Forest	88.9 ± 2.5%	n_estimators = 800, max_depth = 9
4	Multi-layer Perception	80.4 ± 1.4%	α = 1, max_iter =1000
5	Gaussian Process	79.8 ± 0.9%	ConstantKernel, RBF Kernel
6	Naive Bayes	59.1 ± 2.8%	α = 1, max_iter = 1000
7	Logistic Regression	81.9 ± 0.0%	solver = “liblinear”, C = 0.5
8	Graph Neural Network	80.6 ± 1.3%	batch normalize = false

[a] average accuracy of 10 model runs reported with standard deviation.

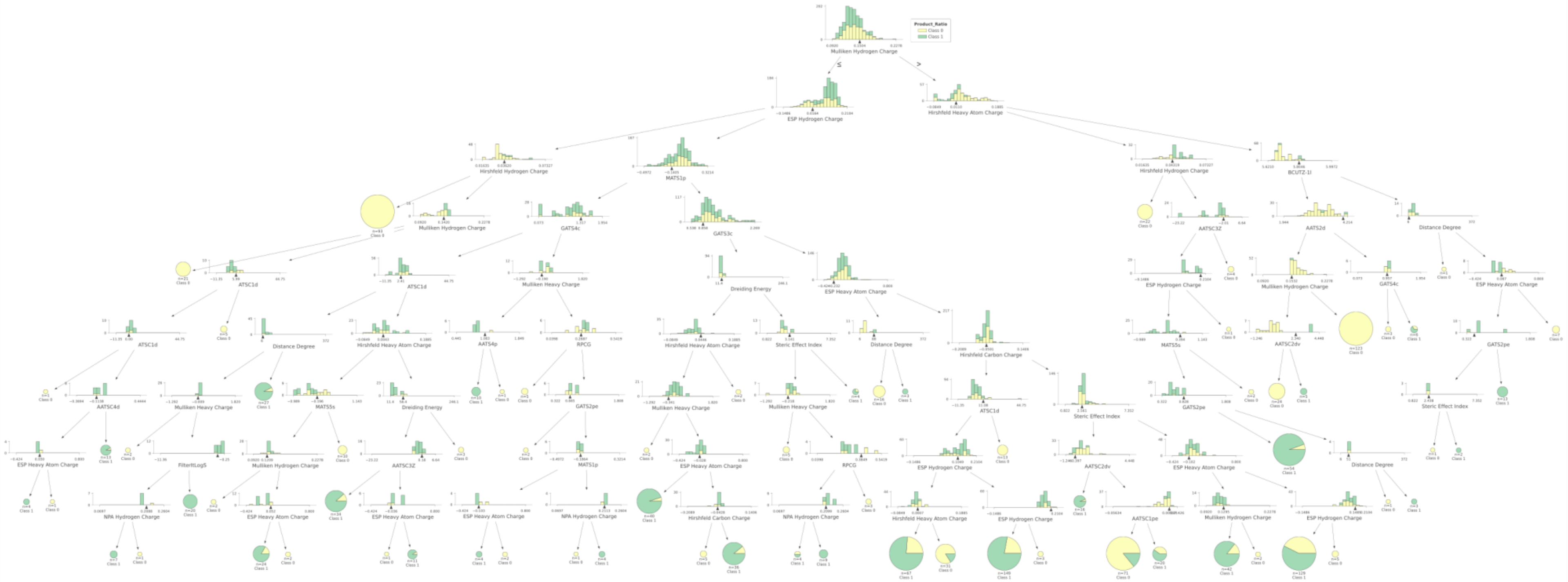

Figure S4. Example random forest tree, created using dtreeviz library.[63]

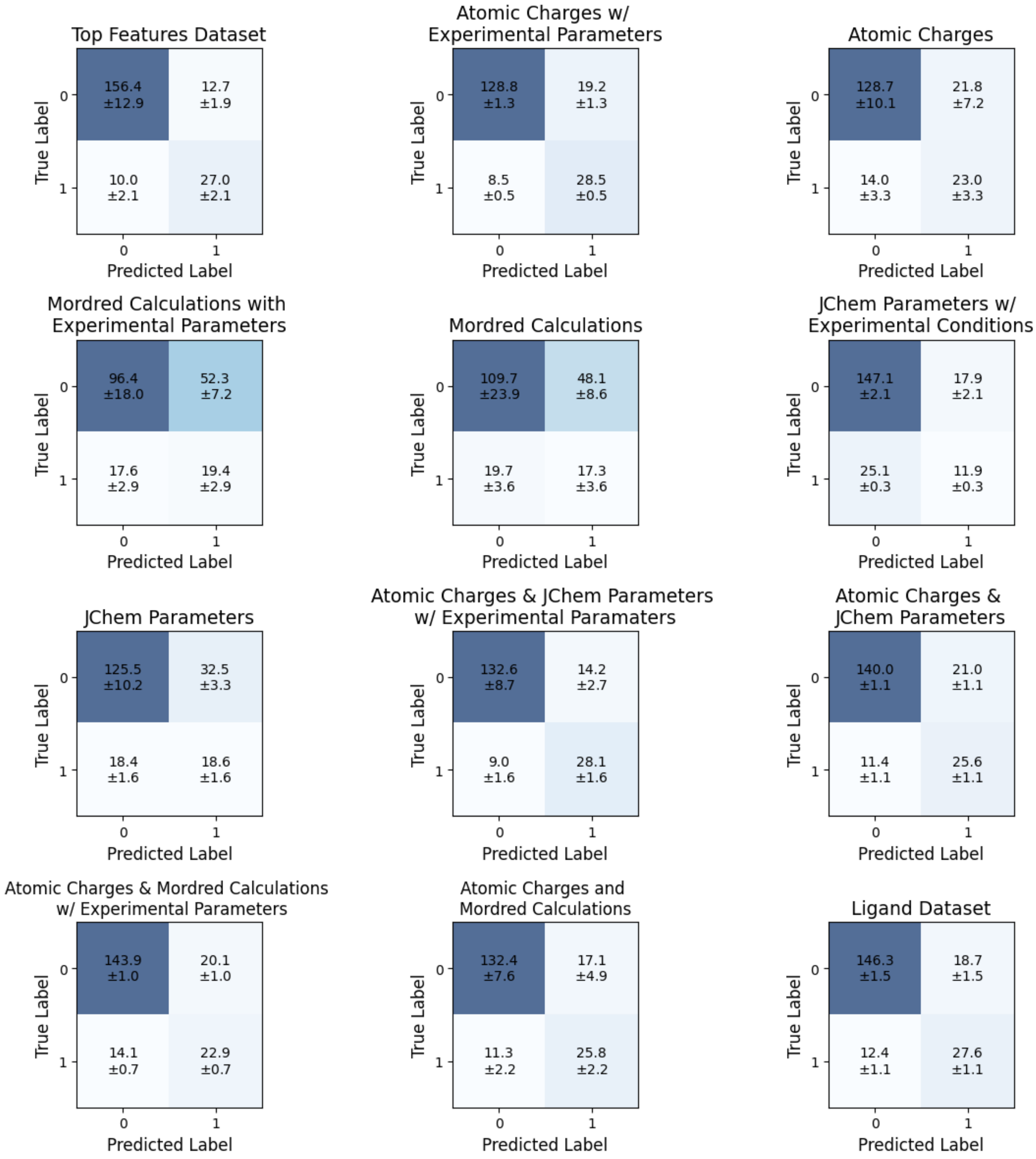

Figure S5. Confusion matrices for reported models.

3. **Table S10.** Table of Contents for GitHub Repository

Name	Description
README.md	General instructions for model usage and repository navigation
Catalyst Data	Folder containing active catalyst parameterization data
Gaussian Files	Model substrates Gaussian output files
BorylationModel_performanceReport_rfc.ipynb	Machine learning model used for dataset
BorylationModel_Validation.ipynb	Machine learning model used for extra validation dataset
BorylationTrainingTest 1-10-25.csv	Model substrates dataset
Validation1-9-25.csv	Input file for Table 2 and Figure 9 of main article
NonselectiveValidation9-1-24.csv	Input file for Figure 6 of main article

4. Model Operation Guide

1. **Table S11.** Install required python libraries.

Library	Version
python	3.7.7
pandas	1.1.4
matplotlib	3.1.2
numpy	1.19.4
seaborn	0.11.1
scikit-learn	1.0.2
imblearn	0.0
rdkit-pypi	2021.3.4
mordred	1.2.

2. Run Jupyter Notebook for Model

Our machine learning model is uploaded as **BorylationModel_performanceReport_rfc.ipynb**. The training / test set is saved under **BorylationTrainingTest 1-10-25.csv**. Additional datasets are included in support of the results section of the main article. The python code to run these datasets are included within the model Jupyter notebook. An additional file is included that uses the final set of features to test a compound for borylation. This is included to allow readers to test their own compounds for potential borylation sites. Figure S6 may serve as a reference for using the model. The referenced python code is available in the GitHub: https://github.com/LambertGroupChemistry/CH-Borylation-Model as **BorylationModel_Test_Compound_Example.ipyb**.

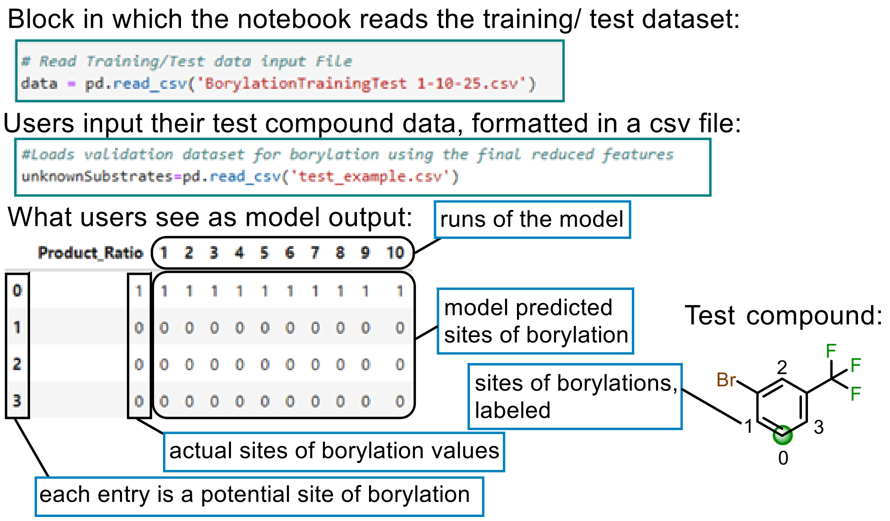

Figure S6. Additional information for model usage.

5. Ablation study on the extra dataset

As outlined in the manuscript, each possible C-H combination is an entry in the random forest model. This allows for multiple sites to have predicted borylation at 100%. The predictive model was run 10 times against the extra dataset, with different groups of features removed to test model performance. These groupings correspond to the groupings of Table 2. Figures S7 – S18 show the accuracy of the ablation study models against the extra validation set.

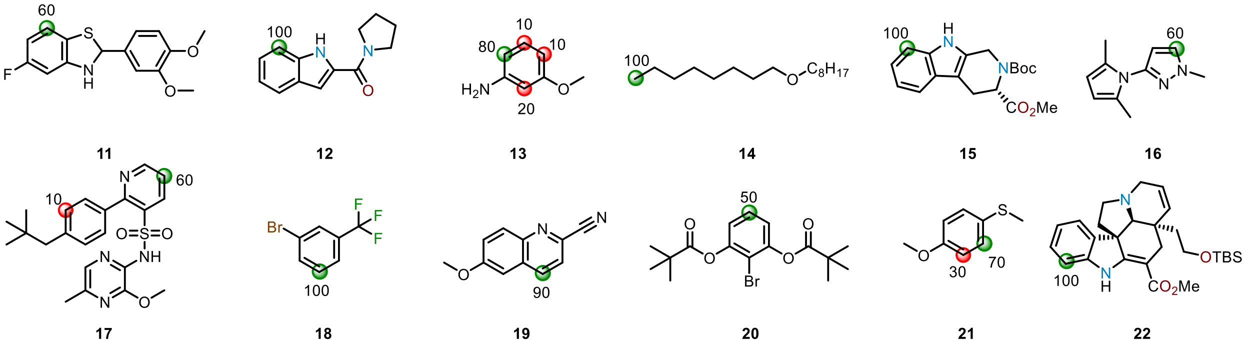

Figure S7. Extra dataset results using computational charges, experimental conditions, Mordred calculations and JChem for Excel calculations, with 10% results shown.

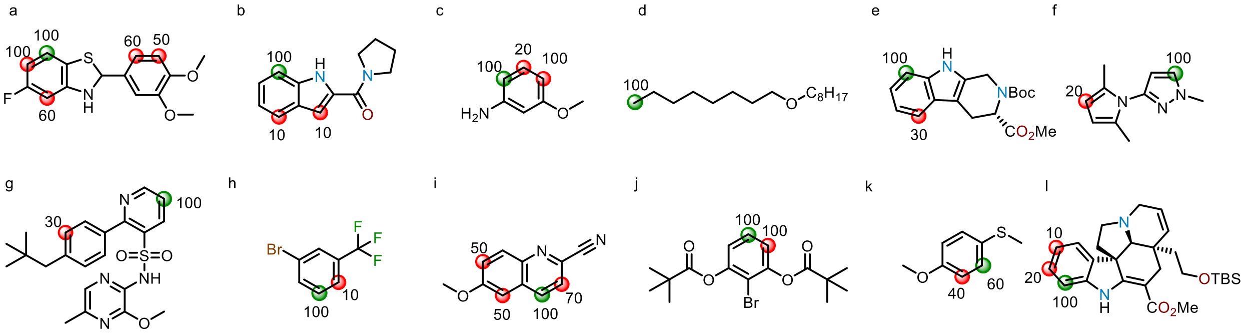

Figure S8. Extra dataset results using computational charges and experimental conditions.

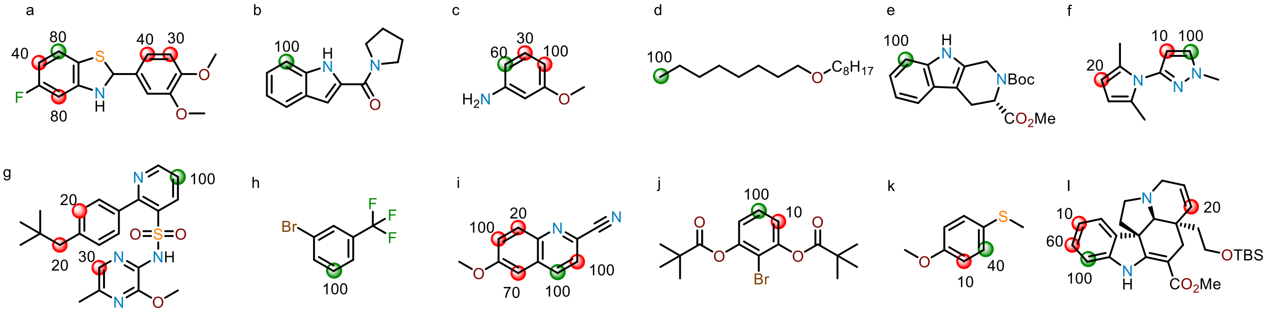

Figure S9. Extra dataset results using computational charges only.

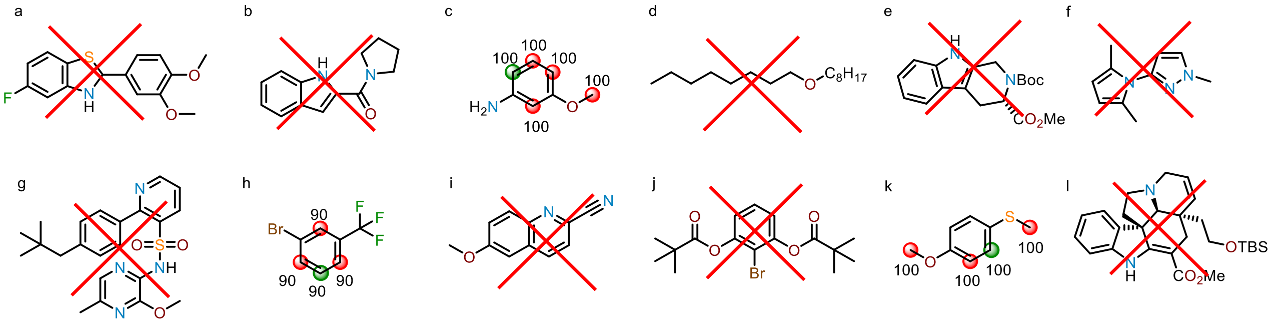

Figure S10. Extra dataset results using Mordred calculations and experimental conditions.

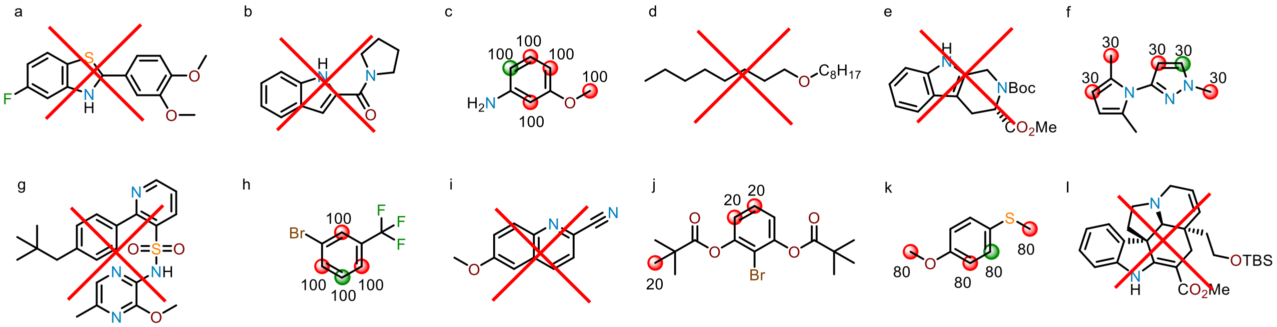

Figure S11. Extra dataset results using Mordred calculations only.

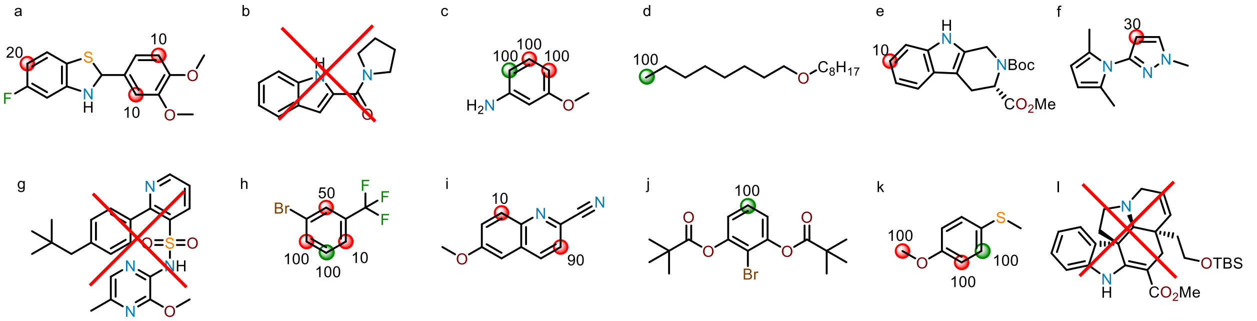

Figure S12. Extra dataset results using JChem for Excel calculations and experimental conditions.

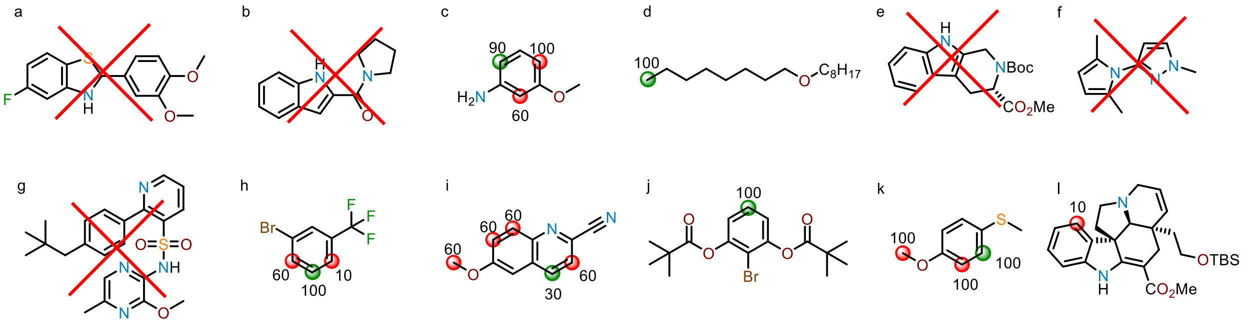

Figure S13. Extra dataset results using JChem for Excel calculations only.

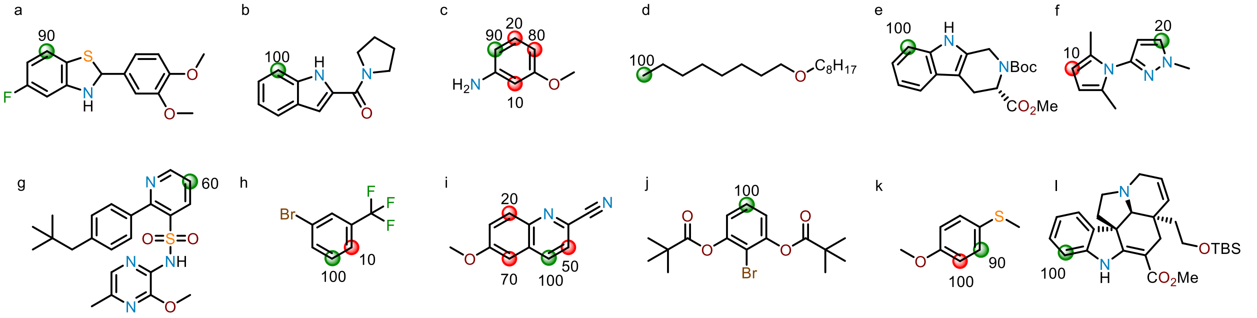

Figure S14. Extra dataset results using computational charges, JChem for Excel calculations, and experimental conditions.

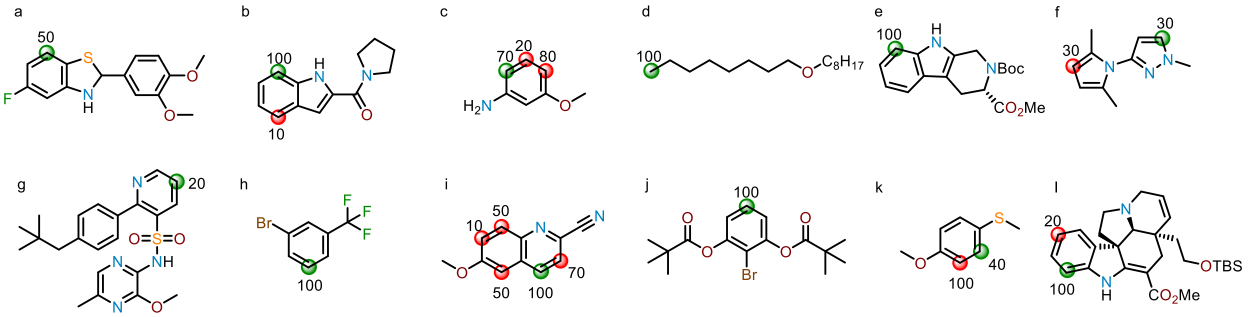

Figure S15. Extra dataset results using computational charges and JChem for Excel calculations.

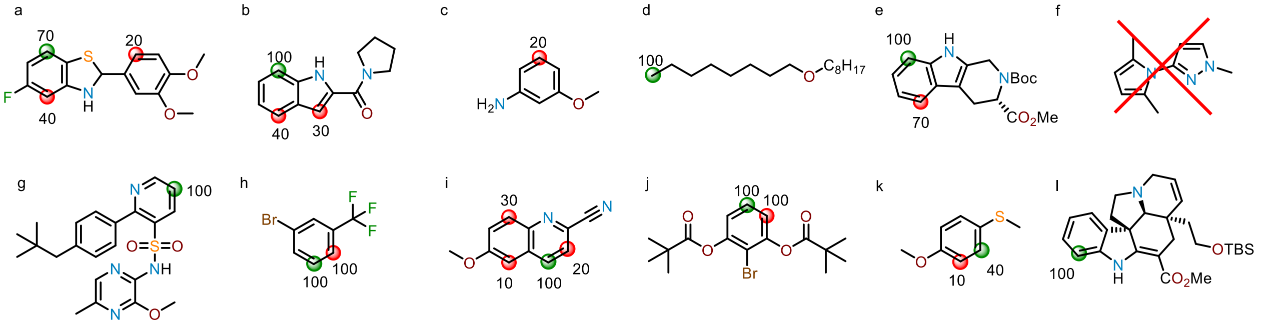

Figure S16. Extra dataset results using computational charges, Mordred calculations and experimental conditions.

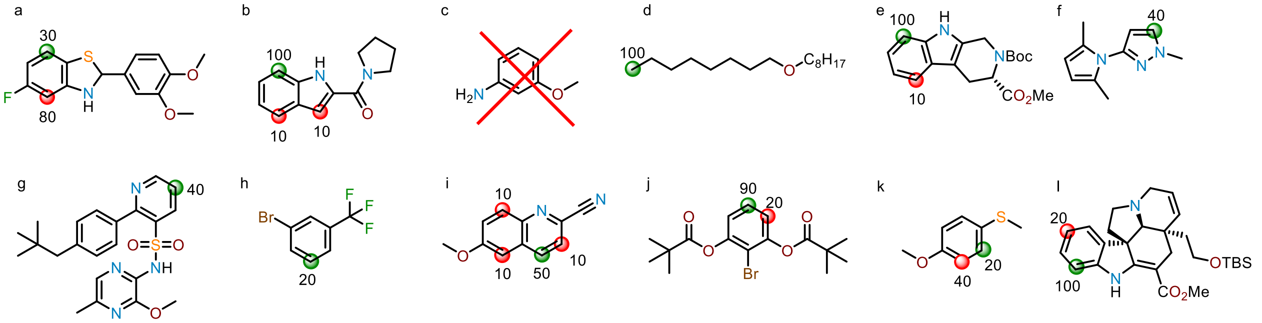

Figure S17. Extra dataset results using computational charges and Mordred calculations.

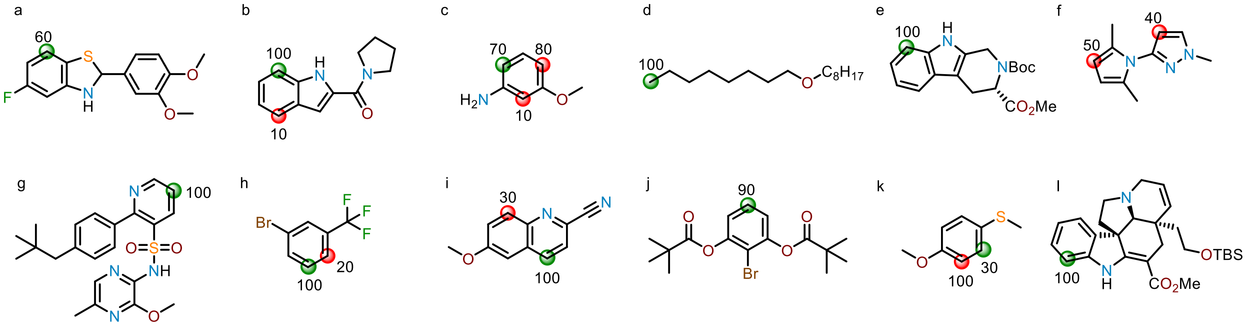

Figure S18. Extra dataset results using the ligand dataset.

6. Compound library Cartesian Coordinates, total energies, and thermal energies

The B3LYP DFT functional was utilized with the 6 311+g(d,p) basis set. All structures were computed in the gas phase and had no imaginary frequencies. All calculations were performed using Gaussian 09 software.[48]

6.1 **Table S12.** Calculated energies for dataset compounds.

File Names	Total Energy (Ha)	Thermal Energy (Ha)	Thermal Enthalpy (Ha)	Thermal Free Energy (Ha)	Thermal Energy (Kcal/mol)	Thermal Enthalpy (Kcal/mol)	Thermal Free Energy (Kcal/mol)
1	-631.1957	-630.9834	-630.9825	-631.0348	-395948.3908	-395947.7978	-395980.6323
2	-1023.4830	-1023.4830	-1023.4821	-1023.5519	-642245.8261	-642245.2337	-642289.0383
3	-1588.7817	-1588.6734	-1588.6725	-1588.7234	-996908.4477	-996907.8554	-996939.7944
4	-895.7403	-895.4637	-895.4627	-895.5248	-561912.4113	-561911.8190	-561950.7767
5	-406.8253	-406.7231	-406.7222	-406.7599	-255222.8119	-255222.2195	-255245.9343
6	-422.1153	-421.9722	-421.9712	-422.0164	-264791.7551	-264791.1628	-264819.4836
7	-745.0967	-744.9562	-744.9553	-745.0013	-467467.4726	-467466.8802	-467495.7858
8	-315.8021	-315.5467	-315.5458	-315.5947	-198008.7367	-198008.1443	-198038.8597
9	-312.3848	-312.1827	-312.1817	-312.2270	-195897.7529	-195897.1605	-195925.5673
10	-2219.5842	-2218.7919	-2218.7909	-2218.9151	-1392314.0989	-1392313.5065	-1392391.4320
11	-1282.2966	-1282.0411	-1282.0402	-1282.1065	-804493.6245	-804493.0321	-804534.6674
12	-688.7509	-688.4886	-688.4877	-688.5420	-432033.4833	-432032.8909	-432066.9848
13	-846.5748	-846.4679	-846.4670	-846.5085	-531167.0826	-531166.4902	-531192.5432
14	-704.9056	-704.4776	-704.4766	-704.5601	-442066.7130	-442066.1207	-442118.4989
15	-1109.9126	-1109.5105	-1109.5095	-1109.5872	-696228.9050	-696228.3126	-696277.0413
16	-553.2661	-553.0374	-553.0365	-553.0905	-347036.5271	-347035.9347	-347069.8504
17	-1697.1751	-1696.6941	-1696.6932	-1696.7857	-1064692.5354	-1064691.9430	-1064750.0097
18	-3143.0015	-3142.8977	-3142.8967	-3142.9444	-1972199.7113	-1972199.1183	-1972229.0329
19	-608.8501	-608.6734	-608.6724	-608.7220	-381948.6189	-381948.0265	-381979.1259
20	-3497.6792	-3497.3154	-3497.3144	-3497.3952	-2194600.3760	-2194599.7830	-2194650.4306
21	-784.4043	-784.2331	-784.2322	-784.2803	-492114.1295	-492113.5365	-492143.7392
22	-1676.8814	-1676.2432	-1676.2423	-1676.3424	-1051859.3842	-1051858.7919	-1051921.5975
23	-1084.1746	-1083.6745	-1083.6735	-1083.7515	-680016.5585	-680015.9661	-680064.9345
24	-1084.1746	-1083.6745	-1083.6735	-1083.7515	-680016.5855	-680015.9580	-680064.9038
S1	-315.8028	-315.5469	-315.5459	-315.5945	-198008.8252	-198008.2328	-198038.6783
S2	-472.4341	-472.0742	-472.0733	-472.1394	-296231.2900	-296230.6977	-296272.1811
S3	-432.5663	-432.2430	-432.2421	-432.2949	-271236.8231	-271236.2308	-271269.3639
S4	-276.4766	-276.2516	-276.2507	-276.2948	-173350.6641	-173350.0717	-173377.7776
S5	-908.9372	-908.7862	-908.7852	-908.8310	-570272.4095	-570271.8172	-570300.5546
S6	-1329.2346	-1329.1208	-1329.1198	-1329.1656	-834036.5869	-834035.9946	-834064.7264
S7	-1485.3631	-1485.1503	-1485.1494	-1485.2017	-931946.6861	-931946.0937	-931978.9276
S8	-1144.8839	-1144.5547	-1144.5537	-1144.6205	-718219.5123	-718218.9199	-718260.8363
S9	-987.5865	-987.3761	-987.3752	-987.4280	-619588.3891	-619587.7967	-619620.9242
S10	-948.2588	-948.0784	-948.0775	-948.1263	-594928.6743	-594928.0819	-594958.7521
S11	-582.7058	-582.7058	-582.7048	-582.7622	-365653.7115	-365653.1192	-365689.1382
S12	-547.6674	-547.3032	-547.3023	-547.3699	-343438.2417	-343437.6487	-343480.0809

S13	-346.9286	-346.9286	-346.9276	-346.9683	-217701.1363	-217700.5439	-217726.0572
S14	-310.9975	-310.9975	-310.9966	-311.0365	-195154.0444	-195153.4514	-195178.5072
S15	-310.9846	-310.9846	-310.9836	-311.0220	-195145.9332	-195145.3408	-195169.4366
S16	-271.8496	-271.6983	-271.6974	-271.7324	-170493.4203	-170492.8279	-170514.7851
S17	-700.5915	-700.5915	-700.5905	-700.6592	-439628.1427	-439627.5497	-439670.6759
S18	-542.4994	-542.2187	-542.2178	-542.2674	-340247.6615	-340247.0691	-340278.1867
S19	-468.4824	-468.1841	-468.1832	-468.2340	-293790.2209	-293789.6285	-293821.5167
S20	-468.4850	-468.1861	-468.1852	-468.2364	-293791.4759	-293790.8829	-293823.0422
S21	-463.8607	-463.6418	-463.6409	-463.6899	-290939.8747	-290939.2823	-290970.0410
S22	-487.9188	-487.5777	-487.5768	-487.6336	-305959.8913	-305959.2989	-305994.9503
S23	-716.6891	-716.6891	-716.6882	-716.7611	-449729.5922	-449728.9998	-449774.7629
S24	-251.8035	-251.8035	-251.8025	-251.8396	-158009.1860	-158008.5937	-158031.8756
S25	-291.0943	-291.0943	-291.0933	-291.1310	-182664.5666	-182663.9743	-182687.5956
S26	-330.3995	-330.3995	-330.3986	-330.4406	-207328.9997	-207328.4073	-207354.7903
S27	-327.0188	-327.0188	-327.0178	-327.0552	-205207.5465	-205206.9535	-205230.3922
S28	-519.2345	-518.9968	-518.9959	-519.0478	-325675.7115	-325675.1191	-325707.6975
S29	-558.5903	-558.3220	-558.3211	-558.3748	-350352.6401	-350352.0477	-350385.7921
S30	-741.8145	-741.6005	-741.5995	-741.6514	-465361.7103	-465361.1179	-465393.6706
S31	-675.3485	-675.0136	-675.0126	-675.0719	-423577.7791	-423577.1867	-423614.3830
S32	-561.9543	-561.6339	-561.6330	-561.6940	-352430.9118	-352430.3194	-352468.6170
S33	-519.2377	-519.0006	-518.9997	-519.0522	-325678.0791	-325677.4867	-325710.4448
S34	-680.3570	-680.1154	-680.1145	-680.1684	-426779.2253	-426778.6330	-426812.4890
S35	-719.6821	-719.4104	-719.4094	-719.4664	-451437.1956	-451436.6033	-451472.3487
S36	-755.3845	-755.1521	-755.1512	-755.2043	-473865.5213	-473864.9289	-473898.2302
S37	-1418.3125	-1418.3125	-1418.3116	-1418.3792	-890005.2926	-890004.7002	-890047.1061
S38	-854.4282	-854.4282	-854.4272	-854.4820	-536162.2279	-536161.6355	-536196.0061
S39	-794.4495	-794.4495	-794.4485	-794.5047	-498524.9769	-498524.3845	-498559.6161
S40	-889.0734	-889.0734	-889.0724	-889.1342	-557902.4398	-557901.8475	-557940.6156
S41	-1078.5857	-1078.3406	-1078.3397	-1078.4002	-676669.5338	-676668.9414	-676706.9202
S42	-709.1757	-709.0104	-709.0094	-709.0564	-444911.0941	-444910.5018	-444939.9885
S43	-709.1752	-709.0098	-709.0089	-709.0569	-444910.7515	-444910.1585	-444940.3123
S44	-386.2126	-386.0447	-386.0437	-386.0866	-242246.8921	-242246.2998	-242273.1980
S45	-461.4436	-461.2696	-461.2687	-461.3159	-289451.2873	-289450.6950	-289480.3655
S46	-538.8694	-538.6574	-538.6564	-538.7036	-338012.8975	-338012.3052	-338041.8891
S47	-500.7381	-500.5339	-500.5329	-500.5847	-314090.0201	-314089.4277	-314121.8750
S48	-362.9316	-362.8040	-362.8030	-362.8421	-227663.1267	-227662.5344	-227687.0744
S49	-402.2407	-402.0834	-402.0825	-402.1250	-252311.3801	-252310.7877	-252337.4600
S50	-2861.2301	-2861.1164	-2861.1154	-2861.1563	-1795379.1390	-1795378.5466	-1795404.1641
S51	-624.8381	-624.7076	-624.7067	-624.7527	-392010.2623	-392009.6699	-392038.5498
S52	-822.5560	-822.4366	-822.4356	-822.4777	-516087.1708	-516086.5778	-516112.9941
S53	-624.8398	-624.7094	-624.7084	-624.7548	-392011.3749	-392010.7825	-392039.9147
S54	-846.5748	-846.4679	-846.4670	-846.5085	-531167.0826	-531166.4902	-531192.5432
S55	-379.9577	-379.8348	-379.8338	-379.8743	-238350.1266	-238349.5342	-238374.9120
S56	-422.1169	-421.9719	-421.9710	-422.0126	-264791.5838	-264790.9915	-264817.0953
S57	-422.1153	-421.9707	-421.9698	-422.0116	-264790.8634	-264790.2711	-264816.4935
S58	-464.8540	-464.6272	-464.6262	-464.6745	-291558.1848	-291557.5924	-291587.8647

S59	-767.1812	-767.0798	-767.0789	-767.1179	-481350.2742	-481349.6812	-481374.1472
S60	-468.2612	-467.9803	-467.9794	-468.0333	-293662.3381	-293661.7458	-293695.5654
S61	-425.5169	-425.3205	-425.3196	-425.3655	-266892.8808	-266892.2884	-266921.1187
S62	-310.9663	-310.8037	-310.8028	-310.8451	-195032.4260	-195031.8337	-195058.4307
S63	-582.5610	-582.2618	-582.2609	-582.3186	-365375.1228	-365374.5298	-365410.7447
S64	-740.3073	-740.1025	-740.1015	-740.1542	-464421.7110	-464421.1186	-464454.1351
S65	-906.6078	-906.4872	-906.4862	-906.5390	-568829.7528	-568829.1604	-568862.3124
S66	-797.2464	-797.2464	-797.2454	-797.3001	-500280.0696	-500279.4773	-500313.7788
S67	-3328.9252	-3328.7015	-3328.7005	-3328.7579	-2088793.4670	-2088792.8746	-2088828.8598
S68	-1029.0818	-1028.9777	-1028.9767	-1029.0231	-645693.7658	-645693.1734	-645722.2805
S69	-502.9654	-502.7320	-502.7310	-502.7818	-315469.3322	-315468.7392	-315500.5759
S70	-709.1736	-709.0076	-709.0067	-709.0527	-444909.3779	-444908.7855	-444937.6654
S71	-709.1734	-709.0072	-709.0063	-709.0522	-444909.1081	-444908.5157	-444937.3523
S72	-1006.9988	-1006.8545	-1006.8535	-1006.9052	-631811.2566	-631810.6643	-631843.0896
S73	-669.8484	-669.7121	-669.7112	-669.7529	-420251.0411	-420250.4488	-420276.6128
S74	-897.7918	-897.6097	-897.6087	-897.6647	-563259.0566	-563258.4642	-563293.5608
S75	-1129.4708	-1129.3429	-1129.3419	-1129.3869	-708673.9349	-708673.3426	-708701.5742
S76	-437.9327	-437.8166	-437.8157	-437.8581	-274734.3229	-274733.7299	-274760.3363
S77	-437.9326	-437.8166	-437.8157	-437.8581	-274734.2972	-274733.7048	-274760.3288
S78	-731.1369	-731.1369	-731.1360	-731.1771	-458795.7362	-458795.1438	-458820.9383
S79	-2845.1819	-2845.0570	-2845.0560	-2845.0981	-1785301.7030	-1785301.1106	-1785327.5269
S80	-444.9378	-444.8296	-444.8286	-444.8686	-279135.0173	-279134.4243	-279159.4977
S81	-460.2281	-460.0775	-460.0766	-460.1206	-288703.2452	-288702.6522	-288730.2501
S82	-805.2831	-805.1759	-805.1750	-805.2163	-505255.9409	-505255.3479	-505281.2992
S83	-384.9950	-384.8502	-384.8492	-384.8904	-241497.3245	-241496.7315	-241522.5718
S84	-460.2581	-460.1064	-460.1055	-460.1497	-288721.3664	-288720.7741	-288748.5382
S85	-805.2904	-805.1832	-805.1822	-805.2235	-505260.4872	-505259.8949	-505285.8104
S86	-574.7701	-574.5848	-574.5839	-574.6352	-360557.7053	-360557.1130	-360589.3124
S87	-644.7092	-644.5914	-644.5905	-644.6363	-404487.5613	-404486.9690	-404515.7309
S88	-1226.8032	-1226.7100	-1226.7091	-1226.7512	-769772.8228	-769772.2305	-769798.6712
S89	-574.8368	-574.6503	-574.6494	-574.6997	-360598.8367	-360598.2437	-360629.8313
S90	-1132.5521	-1132.2741	-1132.2732	-1132.3358	-710513.3500	-710512.7576	-710552.0360
S91	-1439.1495	-1438.7674	-1438.7665	-1438.8471	-902840.9287	-902840.3357	-902890.9563
S92	-970.7726	-970.5073	-970.5064	-970.5726	-609003.0597	-609002.4673	-609044.0430
S93	-1415.8791	-1415.5362	-1415.5352	-1415.6154	-888263.1165	-888262.5241	-888312.8473
S94	-1057.3099	-1057.0368	-1057.0359	-1057.0965	-663301.1818	-663300.5895	-663338.6166
S95	-1093.2360	-1092.9876	-1092.9866	-1093.0472	-685860.6175	-685860.0251	-685898.0435
S96	-772.2178	-771.9388	-771.9379	-772.0006	-484399.3126	-484398.7203	-484438.0865
S97	-1192.5081	-1192.2665	-1192.2656	-1192.3273	-748159.1589	-748158.5666	-748197.3172
S98	-1093.2443	-1092.9954	-1092.9945	-1093.0542	-685865.5617	-685864.9687	-685902.4410
S99	-661.7240	-661.6110	-661.6101	-661.6579	-415167.5330	-415166.9407	-415196.9331
S100	-1263.1675	-1262.9739	-1262.9730	-1263.0311	-792528.7551	-792528.1628	-792564.6587
S101	-1221.4228	-1221.3057	-1221.3048	-1221.3567	-766381.5524	-766380.9600	-766413.5654
S102	-1243.7183	-1243.5668	-1243.5658	-1243.6197	-780350.5832	-780349.9902	-780383.7948
S103	-1357.1073	-1356.9434	-1356.9425	-1357.0016	-851495.5718	-851494.9794	-851532.0426
S104	-574.6299	-574.6299	-574.6290	-574.6794	-360586.0286	-360585.4363	-360617.0941

S105	-825.9382	-825.7651	-825.7642	-825.8109	-518175.8717	-518175.2793	-518204.5841
S106	-461.2490	-461.2490	-461.2481	-461.2934	-289438.3907	-289437.7984	-289466.2371
S107	-499.5859	-499.4051	-499.4042	-499.4538	-313381.7250	-313381.1327	-313412.2848
S108	-424.3217	-424.1471	-424.1461	-424.1919	-266156.5354	-266155.9431	-266184.6397
S109	-615.9160	-615.6997	-615.6988	-615.7452	-386357.7489	-386357.1559	-386386.2736
S110	-773.2116	-772.8782	-772.8773	-772.9363	-484988.8056	-484988.2132	-485025.2595
S111	-553.0732	-553.0027	-553.0018	-553.0340	-347014.7356	-347014.1432	-347034.3936
S112	-645.3388	-645.2677	-645.2667	-645.3036	-404911.9275	-404911.3352	-404934.4344
S113	-592.4018	-592.3020	-592.3010	-592.3376	-371675.4142	-371674.8218	-371697.7542
S114	-3126.6103	-3126.5483	-3126.5473	-3126.5845	-1961940.3080	-1961939.7157	-1961963.0371
S115	-823.5129	-823.3282	-823.3272	-823.3766	-516646.6687	-516646.0764	-516677.0471
S116	-1683.1795	-1682.8706	-1682.8697	-1682.9396	-1056018.1290	-1056017.5360	-1056061.4033
S117	-784.4211	-784.2508	-784.2498	-784.2993	-492125.1888	-492124.5964	-492155.6726
S118	-839.7890	-839.6001	-839.5992	-839.6514	-526857.4870	-526856.8946	-526889.6337
S119	-997.0720	-996.7656	-996.7647	-996.8280	-625480.4024	-625479.8094	-625519.5201
S120	-383.6510	-383.6510	-383.6500	-383.6871	-240744.8346	-240744.2422	-240767.5091
S121	-725.8815	-725.6235	-725.6226	-725.6798	-455336.0276	-455335.4352	-455371.3018
S122	-458.4473	-458.4473	-458.4463	-458.4924	-287680.2464	-287679.6540	-287708.5527
S123	-878.7800	-878.7800	-878.7791	-878.8241	-551443.2660	-551442.6737	-551470.9104
S124	-439.0242	-439.0242	-439.0233	-439.0642	-275492.0732	-275491.4809	-275517.1479
S125	-478.3241	-478.3241	-478.3232	-478.3686	-300153.1660	-300152.5737	-300181.0701
S126	-3012.5741	-3012.5741	-3012.5732	-3012.6189	-1890420.3773	-1890419.7843	-1890448.4608
S127	-553.5441	-553.5441	-553.5432	-553.5904	-347354.4864	-347353.8941	-347383.4975
S128	-269.4207	-269.3181	-269.3172	-269.3521	-168999.8116	-168999.2192	-169021.1200
S129	-344.6439	-344.5355	-344.5345	-344.5720	-216199.4647	-216198.8723	-216222.3469
S130	-458.0346	-457.9139	-457.9130	-457.9555	-287345.5683	-287344.9760	-287371.6822
S131	-461.4397	-461.2667	-461.2657	-461.3139	-289449.4537	-289448.8607	-289479.1092
S132	-500.7637	-500.5608	-500.5598	-500.6113	-314106.9032	-314106.3108	-314138.6251
S133	-656.8514	-656.5524	-656.5515	-656.6078	-411993.2066	-411992.6142	-412027.9549
S134	-579.4120	-579.1510	-579.1500	-579.2070	-363423.0233	-363422.4309	-363458.2009
S135	-536.6644	-536.4854	-536.4844	-536.5352	-336649.9421	-336649.3497	-336681.2046
S136	-516.8079	-516.6164	-516.6154	-516.6659	-324181.9283	-324181.3359	-324212.9938
S137	-674.0913	-673.7823	-673.7814	-673.8435	-422805.1317	-422804.5393	-422843.5334
S138	-497.3687	-497.2190	-497.2180	-497.2647	-312009.8784	-312009.2860	-312038.5411
S139	-2624.2046	-2623.4431	-2623.4421	-2623.5645	-1646236.7496	-1646236.1572	-1646312.9600
S140	-706.7577	-706.6375	-706.6366	-706.6751	-443422.1171	-443421.5247	-443445.6876
S141	-1964.0838	-1963.4252	-1963.4243	-1963.5294	-1232068.9692	-1232068.3768	-1232134.3263
S142	-363.9139	-363.7784	-363.7775	-363.8151	-228274.5982	-228274.0058	-228297.6203
S143	-403.2448	-403.0803	-403.0793	-403.1210	-252936.8933	-252936.3003	-252962.4405
S144	-595.0295	-594.8088	-594.8079	-594.8574	-373248.4764	-373247.8840	-373278.9771
S145	-1090.8178	-1090.6139	-1090.6130	-1090.6689	-684371.1271	-684370.5348	-684405.6439
S146	-517.7976	-517.5984	-517.5975	-517.6458	-324798.1845	-324797.5922	-324827.9197
S147	-689.9589	-689.6748	-689.6739	-689.7322	-432777.8381	-432777.2458	-432813.8346
S148	-862.8389	-862.6850	-862.6841	-862.7282	-541343.4926	-541342.9002	-541370.5803
S149	-480.6702	-480.4683	-480.4673	-480.5116	-301498.6498	-301498.0568	-301525.8398
S150	-772.6565	-772.4121	-772.4112	-772.4681	-484696.3439	-484695.7515	-484731.4631

S151	-745.7482	-745.5025	-745.5016	-745.5573	-467810.2976	-467809.7053	-467844.6613
S152	-725.8870	-725.6261	-725.6252	-725.6861	-455337.6579	-455337.0655	-455375.2652
S153	-1434.9656	-1434.5368	-1434.5359	-1434.6193	-900186.1987	-900185.6063	-900237.9864
S154	-403.2420	-403.0773	-403.0764	-403.1179	-252935.0434	-252934.4511	-252960.5410
S155	-517.8026	-517.6020	-517.6010	-517.6499	-324800.4091	-324799.8167	-324830.4844
S156	-709.8424	-709.5726	-709.5716	-709.6289	-445263.8922	-445263.2998	-445299.2442
S157	-670.5244	-670.2828	-670.2818	-670.3383	-420609.1554	-420608.5631	-420643.9910
S158	-1184.9385	-1184.5455	-1184.5445	-1184.6281	-743314.1178	-743313.5255	-743365.9621
S159	-706.4218	-706.2040	-706.2031	-706.2582	-443150.1015	-443149.5092	-443184.1126
S160	-327.0134	-326.8629	-326.8619	-326.9039	-205109.7152	-205109.1222	-205135.4456
S161	-264.3130	-264.3130	-264.3121	-264.3446	-165859.0513	-165858.4583	-165878.8555
S162	-303.6175	-303.6175	-303.6166	-303.6545	-190523.0444	-190522.4520	-190546.2610
S163	-356.5707	-356.5707	-356.5697	-356.6067	-223751.6517	-223751.0593	-223774.2785
S164	-1571.4662	-1571.0564	-1571.0555	-1571.1348	-985853.5984	-985853.0061	-985902.8184
S165	-1078.5830	-1078.0787	-1078.0777	-1078.1530	-676505.1550	-676504.5620	-676551.7972
S166	-1468.8958	-1468.6944	-1468.6935	-1468.7542	-921620.4223	-921619.8299	-921657.9600
S167	-554.9451	-554.7470	-554.7461	-554.7983	-348109.3107	-348108.7183	-348141.4724
S168	-1167.5932	-1167.5174	-1167.5165	-1167.5559	-732628.8719	-732628.2795	-732652.9752
S169	-402.6056	-402.4535	-402.4525	-402.4933	-252543.5851	-252542.9927	-252568.5406
S170	-480.6905	-480.4908	-480.4898	-480.5373	-301512.7574	-301512.1644	-301541.9718
S171	-555.9175	-555.7120	-555.7111	-555.7606	-348714.8421	-348714.2491	-348745.3354
S172	-900.9850	-900.8227	-900.8217	-900.8684	-565275.2462	-565274.6538	-565303.9498
S173	-555.9178	-555.7124	-555.7115	-555.7613	-348715.1013	-348714.5089	-348745.7476
S174	-480.6907	-480.4910	-480.4900	-480.5376	-301512.8999	-301512.3075	-301542.1726
S175	-1321.2748	-1321.1497	-1321.1488	-1321.1943	-829034.6759	-829034.0835	-829062.6622
S176	-533.6282	-533.4572	-533.4562	-533.5040	-334749.7100	-334749.1170	-334779.1208
S177	-1198.8007	-1198.6593	-1198.6583	-1198.7108	-752170.6741	-752170.0818	-752203.0185
S178	-521.2082	-520.9523	-520.9513	-520.9979	-326902.7608	-326902.1685	-326931.3822
S179	-210.2305	-210.1443	-210.1434	-210.1747	-131867.6748	-131867.0824	-131886.7316
S180	-249.5599	-249.4446	-249.4436	-249.4792	-156528.9665	-156528.3735	-156550.6620
S181	-438.1853	-438.0517	-438.0507	-438.0934	-274881.7965	-274881.2042	-274907.9794
S182	-556.1580	-555.9377	-555.9368	-555.9881	-348856.4956	-348855.9033	-348888.0694
S183	-595.4849	-595.2354	-595.2344	-595.2890	-373516.1508	-373515.5584	-373549.8224
S184	-784.0968	-783.8295	-783.8286	-783.8909	-491860.8546	-491860.2622	-491899.3492

6.2 Cartesian coordinates of computed compounds

(1)

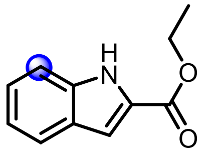

C	1.85106500	-0.63125500	0.00000500
C	1.63140700	0.77727000	-0.00000600
C	2.74221800	1.64176100	0.00000100
C	4.01387000	1.09737200	0.00001000
C	4.20854600	-0.30168900	0.00001000
C	3.13855600	-1.18143600	0.00001400
C	-0.36614800	-0.26924900	-0.00001000
C	0.21737200	0.97832000	-0.00004700
H	2.60026600	2.71676800	-0.00000700
H	4.87918400	1.75008300	0.00001000
H	5.21882800	-0.69477400	0.00001900
H	3.29379700	-2.25437000	0.00003400
H	0.42041100	-2.22609900	0.00000000
H	-0.31446500	1.91596200	-0.00008200
N	0.62133800	-1.23795800	0.00004600
C	-1.76498400	-0.70286300	-0.00000400
O	-2.09999700	-1.87156000	0.00007400
O	-2.62626500	0.33031300	-0.00014700
C	-4.03645300	-0.00653600	-0.00015300
C	-4.81552900	1.29228400	0.00019000
H	-4.24896900	-0.61375100	0.88263900
H	-4.24905400	-0.61330400	-0.88323200
H	-5.88731800	1.07654700	0.00013400
H	-4.58573500	1.88714800	0.88695300
H	-4.58573900	1.88759700	-0.88627400

(2)

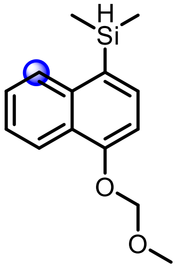

C	0.35925100	-1.37631600	0.03424800
C	0.90707500	-0.11742900	0.07625100
C	0.04686100	1.02332500	-0.01417000
C	-1.36985000	0.82909000	-0.14060700
C	-1.91960600	-0.49903200	-0.17117600
C	-1.03665900	-1.55854600	-0.08875000
H	1.62104300	2.51442100	0.10807900
H	1.01004100	-2.23844000	0.09547500
C	0.55422800	2.35080300	0.01613300
C	-2.19423400	1.98267800	-0.23790400
H	-1.40745400	-2.57777700	-0.11744600
C	-1.67110900	3.25388600	-0.20528200
C	-0.27988500	3.44041700	-0.07410600
H	-3.26400100	1.85706100	-0.35417300
H	-2.32724100	4.11338000	-0.28507800
H	0.13204800	4.44309700	-0.05005700
Si	-3.77911900	-0.85654500	-0.27408600
H	-4.37761500	-0.04534000	-1.37386700
C	-4.64895100	-0.40329100	1.34153400
H	-4.51594700	0.65111100	1.59665600
H	-5.72353700	-0.59827000	1.27051300
H	-4.25368300	-0.99661600	2.17152900
C	-4.07539500	-2.67882700	-0.66313300
H	-5.14806500	-2.85911200	-0.78235800
H	-3.58401100	-2.98035700	-1.59203900
H	-3.72077200	-3.33365300	0.13807000
O	3.06131300	-1.17726000	0.27530500
C	4.41129600	-1.09343200	0.57521900
H	4.76430200	-2.12305900	0.71339300
H	4.57646900	-0.51553100	1.50384500
O	5.10348600	-0.46940800	-0.48912100
C	6.48993300	-0.33285200	-0.23567200
H	6.97290200	-1.31038000	-0.09914600
H	6.92666300	0.15833100	-1.10455300
H	6.67948500	0.28128100	0.65591400
C	2.39726400	0.08228700	0.21343300
H	2.78702400	0.65775700	-0.63302900
H	2.62030100	0.65051000	1.12989600

(3)

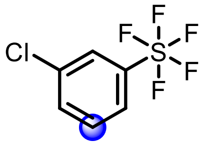

C	1.09060900	-0.51531100	0.00000500
C	0.06589700	0.42239500	0.00000200
C	0.29929200	1.79136600	0.00000300
C	1.62121800	2.23065500	0.00000000
C	2.67614700	1.32282600	0.00000100
C	2.39888900	-0.04107000	0.00000400
H	0.89109600	-1.57632500	0.00000300
H	-0.51662500	2.49899900	0.00000100
H	1.82809300	3.29403300	-0.00000500
H	3.70265600	1.66567000	-0.00000700
S	-1.66869700	-0.18376200	-0.00000300
F	-1.35302600	-1.30359400	1.16069700
F	-1.35300400	-1.30364700	-1.16063800
F	-3.21056900	-0.72024700	-0.00000400
F	-2.11606700	0.88999200	-1.16074600
F	-2.11609300	0.89004200	1.16069000
Cl	3.71885000	-1.19295800	-0.00000200

(4)

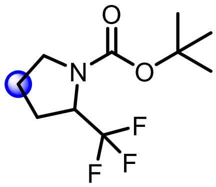

C	2.52604600	-0.69943500	-1.26299900
C	2.80880500	-1.79934400	-0.22373600
C	1.42666400	-2.14273500	0.35360000
C	1.33356600	0.07712300	-0.67025600
H	3.38660600	-0.06142500	-1.46587400
H	2.20669200	-1.13883000	-2.21123800
H	3.46439600	-1.41775200	0.55999800
H	3.28989100	-2.67250600	-0.66675100
H	0.96256700	-2.99416800	-0.15230900
H	1.45877300	-2.37139600	1.42001400
H	0.68793100	0.50638200	-1.43664200
N	0.61982500	-0.93114300	0.10422500
C	-0.74587200	-1.00628500	0.24083100
O	-1.30112500	-1.95459800	0.75986000
O	-1.34917300	0.08186300	-0.28092700
C	-2.80981800	0.28833800	-0.16058100
C	-3.00524300	1.64788500	-0.83220100
H	-4.06157000	1.92615200	-0.80849500
H	-2.43052900	2.41913300	-0.31516900
H	-2.68123300	1.61373000	-1.87505800
C	-3.20727400	0.35066700	1.31586100
H	-4.26036900	0.63431300	1.39435600
H	-3.06912400	-0.61201000	1.80596300
H	-2.61280100	1.10461000	1.83781900
C	-3.55833000	-0.80824100	-0.92170700
H	-3.41226200	-1.78211900	-0.45727800
H	-4.62744300	-0.57848900	-0.92906400
H	-3.21555100	-0.85360900	-1.95890400
C	1.78563700	1.26222600	0.19416400
F	2.67225800	0.90693300	1.15406700
F	0.76053600	1.87198600	0.81596400
F	2.39526700	2.19404300	-0.58117100

(5)

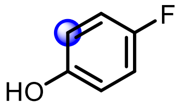

C	-0.67226800	1.22031900	0.00004100
C	0.71867800	1.20989600	-0.00003000
C	1.38324300	-0.00752800	-0.00000200
C	0.70241800	-1.21227500	0.00004500
C	-0.69235200	-1.19706100	0.00000000
C	-1.38021000	0.01702900	0.00003500
H	-1.22102200	2.15387300	0.00010400
H	1.25325000	-2.14445300	0.00003600
H	1.28627400	2.13210100	-0.00004700
H	-1.23934800	-2.13483300	-0.00006400
O	-2.74865200	0.09442300	-0.00003300
H	-3.12726600	-0.79055400	-0.00009400
F	2.74225300	-0.01708800	-0.00002200

(7)

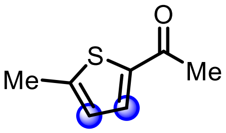

C	1.90594200	0.15294200	0.00000600
C	1.37112900	1.41883000	-0.00006100
C	-0.04616100	1.42781000	-0.00007300
C	-0.59768000	0.16675100	-0.00002800
H	1.98114300	2.31350000	-0.00009500
H	-0.63858300	2.33351800	-0.00011700
C	3.35438000	-0.23042600	0.00003000
H	3.61460400	-0.82273600	0.88212300
H	3.61463600	-0.82271800	-0.88206600
H	3.97658700	0.66652900	0.00005000
C	-2.00897300	-0.24568300	-0.00001900
O	-2.32123100	-1.42510800	-0.00013200
C	-3.06120800	0.84947300	0.00012300
H	-2.95904100	1.48643000	0.88324100
H	-2.95907800	1.48662500	-0.88285700
H	-4.04669200	0.38639800	0.00009600
S	0.65510600	-1.05405400	0.00005100

(6)

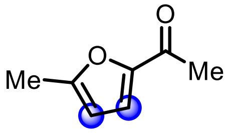

C	1.79241900	-0.00227100	0.00003400
C	1.50418000	1.33632900	-0.00008400
C	0.08537300	1.44068600	-0.00011600
C	-0.40499900	0.15959700	-0.00004000
O	0.64458300	-0.72186200	-0.00001500
H	2.22191800	2.14137000	-0.00011900
H	-0.49987400	2.34671100	-0.00017700
C	3.06519800	-0.76854000	0.00011700
H	3.13472000	-1.41108200	0.88270900
H	3.13479000	-1.41115800	-0.88241600
H	3.91410100	-0.08412300	0.00012000
C	-1.76468000	-0.39224700	-0.00001400
O	-1.97085200	-1.59210100	-0.00015500
C	-2.90155300	0.61595300	0.00019000
H	-2.85240400	1.25919100	0.88347600
H	-2.85254100	1.25939100	-0.88295900
H	-3.84617700	0.07436500	0.00020400

(8)

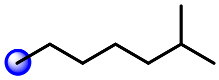

C	-1.65219200	-0.36712400	0.15897200
C	-2.88821100	0.43954600	-0.25505200
H	-1.71875600	-1.37469100	-0.27204000
H	-1.65915800	-0.50329700	1.24841200
C	-4.20763600	-0.21374800	0.16671100
H	-2.82424800	1.44527800	0.17812400
H	-2.88085200	0.57712300	-1.34326400
H	-5.06820900	0.38600400	-0.14276600
H	-4.31788400	-1.20713600	-0.27984800
H	-4.26016400	-0.33349400	1.25354700
C	-0.32588600	0.27737500	-0.26159900
C	0.90243000	-0.53819000	0.16085700
H	-0.27241700	1.28610100	0.16345800
H	-0.31696400	0.40435600	-1.35229300
C	2.25960200	0.00670900	-0.32301500
H	0.92481100	-0.62146100	1.25646100
H	0.78677500	-1.56219100	-0.21674800
H	2.20084700	0.12002400	-1.41465700
C	3.38135500	-0.99624100	-0.01782000
H	4.34595700	-0.64064600	-0.39216100
H	3.48209000	-1.15252100	1.06203100
H	3.18181100	-1.96927500	-0.47672400
C	2.58960900	1.38192000	0.27557300
H	1.84529600	2.13634600	0.00972400
H	2.63556200	1.32811200	1.36940000
H	3.56107100	1.73988800	-0.07841700

(9)

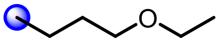

C	2.44493700	0.47045700	0.00025600
C	3.60877100	-0.50392100	0.00009200
H	2.48601600	1.12277200	-0.88604900
H	2.48537900	1.12168900	0.88738800
H	4.55815600	0.03889500	0.00076800
H	3.57539100	-1.14341600	0.88524900
H	3.57603700	-1.14234500	-0.88586100
C	0.07330700	0.55402700	-0.00003700
C	-1.16326800	-0.33234000	0.00017500
H	0.07645000	1.20790700	-0.88702900
H	0.07710000	1.20745900	0.88728000
C	-2.47051000	0.46824500	-0.00014700
H	-1.12515300	-0.98606700	-0.87823000
H	-1.12533200	-0.98554200	0.87898100
C	-3.71568800	-0.42345400	0.00013300
H	-2.49543700	1.12714200	0.87636600
H	-2.49536400	1.12652100	-0.87712600
H	-4.63275800	0.17207300	-0.00005800
H	-3.73827400	-1.07003700	-0.88255900
H	-3.73828100	-1.06947400	0.88323700
O	1.23134800	-0.26570700	-0.00064800

(10)

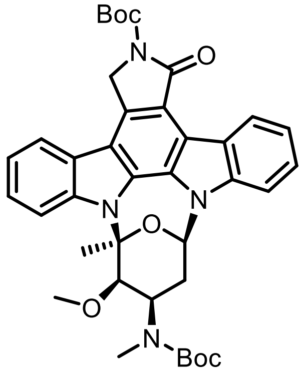

C	0.91542600	2.33789500	-1.35248300
C	1.25673400	0.98009900	-0.98582700
C	2.40664200	0.33298800	-0.47709200
C	3.74265500	0.88491800	-0.16851000
C	2.37846500	-1.01803700	-0.16765200
C	1.21514000	-1.77634900	-0.35254400
C	0.85175700	-3.13673500	-0.04566600
C	-0.51748400	-3.28982200	-0.38537100
N	-0.99322300	-2.07390600	-0.92243300
C	0.05993200	-1.15979900	-0.88537500
C	0.08870600	0.20746700	-1.18864100
N	-0.94460700	1.02804300	-1.61766700
C	-0.44715000	2.32156400	-1.75096800
O	4.14157200	2.01944000	-0.30391000
O	-2.02824000	-0.79765600	-2.63579600
C	-2.23261500	-1.87476600	-1.72516800
C	-3.52891100	-1.63054100	-0.88491500
C	-3.81590100	-0.19605200	-0.35501200
O	-3.60722600	-2.63869200	0.09650600
C	-2.45101200	-3.06915900	-2.66508300
H	-3.21356800	-2.79046200	-3.39289400
H	-1.52527400	-3.28999900	-3.19673100
H	-2.77892100	-3.95828400	-2.13454900
C	-4.90362400	-2.84600500	0.64478300
H	-4.82985600	-3.71865800	1.29328100
H	-5.23346000	-1.98915400	1.23992300
H	-5.63771600	-3.04581800	-0.14742400
C	-2.04376700	-0.26930200	1.52298200
H	-1.24536700	0.35978300	1.12232000
H	-2.06357300	-0.14957800	2.60286100
H	-1.85188800	-1.31018100	1.28123100
C	-1.09762700	3.47111500	-2.20423400
H	-2.13714800	3.45872800	-2.50954900
C	-0.36483600	4.65309600	-2.24572700
H	-0.84477700	5.56109600	-2.59334000
C	0.97818700	4.69098900	-1.84040100
H	1.51865600	5.62969100	-1.87811300
C	1.62605400	3.54550900	-1.39404500
H	2.66288500	3.56423800	-1.08641900
C	1.56386700	-4.20441100	0.51462700
H	2.60987100	-4.09288900	0.77391500
C	0.91204600	-5.40787800	0.74265600
H	1.45091400	-6.24230000	1.17584600
C	-0.44898400	-5.54070500	0.43503300
H	-0.95531600	-6.47591500	0.64607300
C	-1.17751900	-4.49284100	-0.11775400
H	-2.23618600	-4.60274900	-0.28815700
C	3.70698400	-1.45235000	0.37646500
H	3.64387100	-1.83064800	1.40139100
H	4.18377400	-2.22798000	-0.23031300
N	4.49425700	-0.21630000	0.33588900
C	-3.45547300	0.82555900	-1.46037800
H	-3.42060900	1.82887900	-1.03375300
H	-4.27227300	0.82154700	-2.19003800
C	-2.17310000	0.55753200	-2.25093400
H	-2.24845800	1.08713700	-3.20560500
H	-4.32973100	-1.78857900	-1.62492500
H	-4.89930000	-0.14859300	-0.27870000
C	5.81791900	-0.27224900	0.77418200
O	6.29482700	-1.31264200	1.18227400
O	6.42805900	0.90399800	0.68008100
C	7.83998200	1.08289900	1.09342900
C	8.07126600	2.56549800	0.80264600
H	9.09621900	2.84065100	1.06388200
H	7.90895600	2.77808400	-0.25578200
H	7.38251000	3.18213800	1.38321200
C	8.75164900	0.20767400	0.23071800
H	9.79528500	0.44783200	0.45186300
H	8.58903200	-0.85136600	0.42574100
H	8.57629300	0.40647900	-0.82961200
C	7.98729100	0.78837500	2.58756000
H	7.81478800	-0.26476500	2.80490800
H	8.99937000	1.05068900	2.90804000
H	7.28247200	1.39199100	3.16497300
C	-4.08974800	0.97881300	1.79718700
O	-3.75078200	1.33100700	2.91058800
O	-5.24046700	1.37190000	1.18762400
C	-6.20652800	2.25646000	1.87545200
C	-5.56187400	3.61321300	2.16916300
H	-4.76504900	3.52228000	2.90495100
H	-5.15123200	4.04602400	1.25306700
H	-6.32174000	4.29835500	2.55517700
C	-6.73983700	1.57810300	3.13984500
H	-7.54592200	2.18432000	3.56223800
H	-7.14968600	0.59296100	2.90036400
H	-5.95797000	1.46446500	3.88857300
C	-7.31727700	2.40274800	0.83380700
H	-8.11037100	3.04534100	1.22314000
H	-6.93021200	2.85152800	-0.08400000
H	-7.75124000	1.43015900	0.58970600
N	-3.35565800	0.12524900	1.00189100

(11)

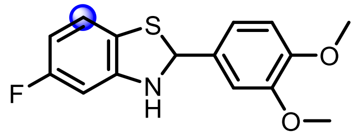

C	-3.01472100	-0.91707100	-0.06117000
C	-2.63151400	0.43913300	0.06121000
C	-3.61371200	1.43418900	0.16505500
C	-4.93223000	1.03052500	0.14253600
C	-5.32652600	-0.30372600	0.02329700
C	-4.35685900	-1.29442200	-0.08042400
C	-0.59154400	-0.42739600	-0.04399500
H	-3.34538600	2.47801800	0.25933300
H	-6.38152800	-0.54583600	0.01304100
H	-4.64838300	-2.33314000	-0.17383900
S	-1.57912300	-1.91105100	-0.16970500
N	-1.27331600	0.67027000	0.06706200
F	-5.90312500	1.97317200	0.24056800
C	0.87428400	-0.48529000	-0.06683900
C	1.57133000	-1.68853400	-0.23535500
C	1.60385900	0.70515600	0.07532700
C	2.96077800	-1.70274500	-0.23787700
H	1.03542200	-2.62247500	-0.35941500
C	2.99122000	0.69661400	0.05732800
H	1.08289800	1.64612600	0.19608900
C	3.68422800	-0.52219700	-0.07794000
H	3.50922800	-2.62917800	-0.35950700
O	5.05374200	-0.56968000	-0.11441700
O	3.67315000	1.88005800	0.21977000
C	4.27079700	2.40127000	-0.97741400
H	4.77713800	3.32127300	-0.68760600
H	4.99312300	1.69569800	-1.39627500
H	3.49976000	2.62787600	-1.72091700
C	5.72071800	-0.26545400	1.12264100
H	5.45722900	-1.00566800	1.88499100
H	6.78742400	-0.32604500	0.91087100
H	5.46459300	0.73640600	1.47286100

(12)

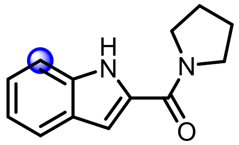

C	-2.34160200	0.63391900	0.06706900
C	-2.07098800	-0.75453300	-0.08980100
C	-3.14757000	-1.65736500	-0.16148900
C	-4.43920900	-1.16664900	-0.07771300
C	-4.68546100	0.21498800	0.07815700
C	-3.64783600	1.13036200	0.15320700
C	-0.10416900	0.36772300	-0.01886300
C	-0.64881300	-0.89734300	-0.14200900
H	-2.96707200	-2.72015800	-0.28094100
H	-5.27926600	-1.84955200	-0.13193200
H	-5.70924400	0.56618900	0.14005600
H	-3.84135300	2.19026700	0.27327100
H	-0.11577800	-1.82245200	-0.27422600
N	-1.13492400	1.27958300	0.10833100
H	-0.94950100	2.27079200	0.16175700
C	1.25405000	0.96798200	-0.03678400
O	1.35136200	2.20033000	-0.05685200
C	2.44416100	-1.28159200	0.12031000
C	3.68934100	0.80979100	-0.10717600
C	3.92526800	-1.51966300	0.44807700
H	2.16246900	-1.78981300	-0.80992400
H	1.77993900	-1.62668900	0.91431000
C	4.64036000	-0.37747700	-0.28906400
H	3.88899700	1.35920000	0.81954200
H	3.72050800	1.53098600	-0.92476300
H	4.08579000	-1.43034600	1.52673300
H	4.26005700	-2.51207900	0.14101100
H	5.63781000	-0.17264100	0.10315400
H	4.74156200	-0.61953600	-1.35153100
N	2.35906600	0.17789400	-0.03907900

(13)

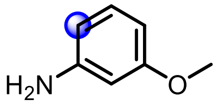

C	-0.24800600	0.90811100	-0.00241100
C	-0.86305600	-0.33983400	-0.00076100
C	-0.05217700	-1.47181000	-0.00218300
C	1.33171200	-1.35569700	-0.00484100
C	1.94461000	-0.09246300	-0.00512200
C	1.12912800	1.04722700	-0.00536900
H	-0.51518000	-2.45057500	-0.00049100
H	1.94250000	-2.25154800	-0.01179800
H	1.55000800	2.04575300	-0.01309000
N	3.33028600	0.02966800	-0.06431200
H	3.71781000	0.90418600	0.25614700
H	3.86046300	-0.76925700	0.24914000
Cl	-2.60606200	-0.47542500	0.00552700
F	-1.00197900	2.02475500	0.00005000

(14)

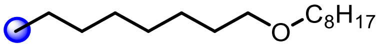

C	3.73979900	0.31056700	0.42684000
C	2.40749900	-0.35766900	0.50088600
H	2.36110700	-1.19903500	-0.20207100
H	2.24641300	-0.80242600	1.49840600
H	3.80933800	1.36353400	0.69113000
C	5.00340000	-0.47588600	0.31739400
H	5.19728100	-1.00818100	1.26737400
H	4.87558200	-1.27505300	-0.42727800
C	6.23992000	0.36322900	-0.03446500
H	6.06616000	0.87317300	-0.98971800
H	6.36193200	1.15380800	0.71678700
C	7.53075700	-0.45749700	-0.12079900
H	7.40677400	-1.25089100	-0.86955500
C	8.76742700	0.37706200	-0.47277800
H	8.89282400	1.16890700	0.27584100
H	8.59767400	0.88746700	-1.42866500
C	10.05279000	-0.45116300	-0.55883400
H	10.91475600	0.17312400	-0.81038000
H	10.26861500	-0.94703400	0.39294200
H	9.97156200	-1.22888900	-1.32485000
H	7.70101500	-0.96826000	0.83613400
C	1.24382700	0.58619700	0.21992400
H	1.26598400	1.43559500	0.92121300
H	1.32339800	0.99727700	-0.79795500
C	-1.12522200	0.65600800	0.10917100
H	-1.16842700	1.49754600	0.81914000
H	-1.06947700	1.08569600	-0.90379300
C	-2.36237700	-0.21941600	0.24443300
H	-2.37530200	-0.65404000	1.24990200
H	-2.27435000	-1.05561500	-0.45780300
C	-3.66459100	0.54680000	-0.01376400
H	-3.75136400	1.37500900	0.70128600
H	-3.62747200	1.00683500	-1.00976700
O	0.02878800	-0.13052400	0.36255800
C	-4.91454400	-0.33578400	0.08346600
H	-4.95143700	-0.80265800	1.07601600
H	-4.82953700	-1.15983600	-0.63636000
C	-6.22244600	0.42279400	-0.16832600
H	-6.31594800	1.23669400	0.56235700
H	-6.17728800	0.90392800	-1.15408500
C	-7.46989800	-0.46524200	-0.09829500
H	-7.37764300	-1.27655400	-0.83215800
H	-7.51515700	-0.95069800	0.88540600
C	-8.77969300	0.29160000	-0.34668200
H	-8.87681300	1.09653300	0.39209500
H	-8.73177100	0.78309300	-1.32615200
C	-10.01876600	-0.60672000	-0.28795300
H	-10.11236200	-1.08835600	0.69061600
H	-10.93512600	-0.03697300	-0.46603700
H	-9.96853400	-1.39880000	-1.04179200

(15)

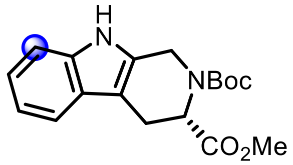

C	3.60510500	-1.35866400	0.19670600
C	3.43676800	0.03230700	-0.05816100
C	4.54391900	0.77972100	-0.48926600
C	5.76397900	0.14073500	-0.66143100
C	5.90577200	-1.23665600	-0.40951600
C	4.83023300	-2.00296700	0.02270900
C	1.46169100	-0.82789000	0.61230100
C	2.05756300	0.33218100	0.21726500
H	4.44929200	1.84209700	-0.68595300
H	6.62472900	0.70911000	-0.99492100
H	6.87109600	-1.70816000	-0.55359700
H	4.94357700	-3.06382800	0.21828100
H	2.18054200	-2.82582700	0.80357700
N	2.38173300	-1.85825200	0.61300000
C	1.26085700	1.59683200	0.21033900
H	1.25804000	2.04064200	1.21387800
H	1.68337600	2.34599100	-0.46191900
C	0.02285300	-0.91490700	0.99341500
H	-0.38066200	-1.91078300	0.81946100
H	-0.11481700	-0.69003300	2.06039800
N	-0.74491500	0.03805400	0.17427800
C	-0.20742700	1.34151100	-0.24057000
H	-0.25125800	1.39425700	-1.32949600
C	-1.07561200	2.47575800	0.31380500
O	-1.64530600	2.46206900	1.37532100
O	-1.04744000	3.53329800	-0.51680100
C	-1.79944800	4.68670500	-0.09469500
H	-1.66368900	5.42572400	-0.88115700
H	-2.85344900	4.42803800	0.01184800
H	-1.42176000	5.06289700	0.85720500
C	-2.00824200	-0.23938300	-0.27907500
O	-2.63307400	0.52266700	-0.99564600
O	-2.45061500	-1.43952900	0.16043800
C	-3.79808600	-1.93712300	-0.19414300
C	-3.90899700	-2.12322500	-1.70917400
H	-4.85880000	-2.61092300	-1.94565000
H	-3.86735800	-1.16729700	-2.22836200
H	-3.10041900	-2.76173200	-2.07502500
C	-4.86797200	-0.99316700	0.35956200
H	-5.85539300	-1.43819500	0.20810400
H	-4.72363600	-0.84198300	1.43226500
H	-4.83905900	-0.02589100	-0.13886700
C	-3.84628700	-3.28749400	0.52218500
H	-4.81143400	-3.76873000	0.34676700
H	-3.05955100	-3.94998800	0.15329700
H	-3.71563400	-3.15767900	1.59888500

(16)

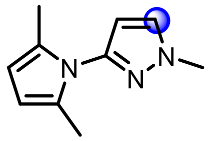

C	-1.81792600	-1.02991900	-0.20881900
C	-3.06532800	-0.46294500	-0.29977800
C	-2.92743500	0.94415300	-0.10738000
C	-1.59725800	1.21904400	0.09427500
N	-0.91390300	0.00455900	0.03413500
H	-3.97957500	-1.00312100	-0.49499300
H	-3.71826200	1.67909700	-0.11524800
C	-1.39198300	-2.45320300	-0.36766000
H	-0.63039700	-2.56849500	-1.14611600
H	-0.98133600	-2.87892000	0.55411400
H	-2.25679900	-3.05436100	-0.65344300
C	-0.90720400	2.51737200	0.35673000
H	-0.35369100	2.50429800	1.30231400
H	-0.19595800	2.77198700	-0.43426100
H	-1.65197400	3.31286200	0.41661400
C	0.47484000	-0.15604600	0.21078200
C	1.13884400	-0.80507500	1.27471400
C	2.47833600	-0.66524800	0.97418200
N	1.33602500	0.34392100	-0.67501900
N	2.55164400	0.03066500	-0.18961100
C	3.74128900	0.43500800	-0.91790700
H	3.76861600	1.52158000	-1.01413000
H	4.61941700	0.09729900	-0.36828800
H	3.74145300	-0.01248300	-1.91317700
H	0.70063900	-1.28692300	2.13243000
H	3.36445200	-0.99567600	1.49280800

(17)

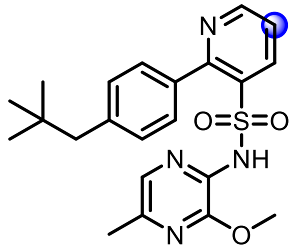

C	-0.44113900	1.90890800	0.11466700
C	0.78234100	1.74134900	-0.57366300
C	1.70394700	2.78342000	-0.61425200
C	1.42511600	3.95799400	0.06991400
C	0.24124000	4.01684000	0.79846800
H	2.63752100	2.65480300	-1.14407600
H	2.11821200	4.78999600	0.06069100
H	-0.00128100	4.89394800	1.39237100
N	-0.65854300	3.03663200	0.81549100
C	-1.56075800	0.93232200	0.14318000
C	-2.19956500	0.66287100	1.36007700
C	-2.04596400	0.31728500	-1.01561200
C	-3.27062800	-0.21935200	1.41699100
H	-1.84377700	1.14543300	2.26202900
C	-3.13032300	-0.55206900	-0.95065100
H	-1.58811400	0.51810200	-1.97602300
C	-3.76521900	-0.84046600	0.26200900
H	-3.72852800	-0.43432900	2.37680700
H	-3.48190400	-1.02034900	-1.86368400
C	-4.93283800	-1.79982800	0.32628800
H	-4.90412100	-2.32509100	1.28742100
H	-4.80212600	-2.56440800	-0.44734500
C	-6.36429600	-1.19681800	0.15887500
C	-6.51574400	-0.50956200	-1.20960300
H	-5.83601600	0.33972400	-1.31203200
H	-6.31200500	-1.20623800	-2.02923200
H	-7.53672400	-0.13660900	-1.33751400
C	-6.66860400	-0.18324600	1.27591700
H	-5.99049100	0.67274500	1.24004900
H	-7.69023300	0.19674400	1.17755300
H	-6.58019200	-0.64436300	2.26509600
C	-7.36748600	-2.36240900	0.25091100
H	-7.29428600	-2.87305100	1.21641900
H	-8.39453900	-2.00094100	0.14116200
H	-7.18839600	-3.10357800	-0.53449600
S	1.21353800	0.18949500	-1.40825700
O	0.88612300	-0.93532000	-0.54577900
O	0.72825700	0.26214300	-2.78708800
N	2.92802500	0.28943800	-1.57750700
H	3.12515700	0.16839500	-2.56897900
C	3.78047100	-0.54121400	-0.81204800
C	3.84448500	-0.46766100	0.60421200
C	5.43398600	-2.11181000	-0.76584500
C	5.47056900	-2.07824800	0.62064400
H	6.08785300	-2.76414800	-1.33348000
N	4.57865000	-1.35303700	-1.47105200
N	4.66002600	-1.23354400	1.29291200
C	6.38940200	-2.94607600	1.43156200
H	7.02054400	-3.56425800	0.79112400
H	7.03150800	-2.33198700	2.06886700
H	5.81202700	-3.60257400	2.08862800
O	3.04897500	0.42545100	1.21326600
C	3.02849100	0.42871200	2.64973100
H	2.69431200	-0.53906600	3.02679500
H	4.01921200	0.64988000	3.04980700
H	2.32094500	1.20903100	2.92141700

(18)

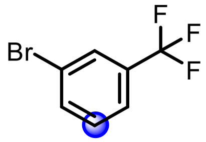

C	1.13763200	0.35408400	-0.00333800
C	1.11824400	1.74597500	0.01123200
C	-0.10862700	2.40630600	0.00282400
C	-1.29987600	1.68691400	-0.01887300
C	-1.25830900	0.29319100	-0.03465500
C	-0.03927400	-0.38579700	-0.02514900
H	2.04500900	2.30457100	0.02603600
H	-0.13165800	3.48955800	0.01073900
H	-2.25181800	2.20156500	-0.03175200
H	-0.01081700	-1.46671900	-0.04216300
C	-2.54253900	-0.49411600	-0.00360200
F	-3.55722200	0.16515100	-0.60505500
F	-2.94355900	-0.74635500	1.26790100
F	-2.42393800	-1.69221300	-0.61620000
Br	2.81795000	-0.56307300	0.00133400

(19)

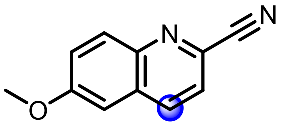

C	1.94696800	-1.20141500	0.00004300
C	0.61827100	-1.55009700	0.00004500
C	-0.39467000	-0.56001300	0.00008900
C	-0.00479800	0.81909100	0.00000300
C	1.36267400	1.15676900	0.00001500
C	2.32820100	0.16741200	0.00006200
H	2.69952900	-1.97833200	-0.00014100
H	0.31195600	-2.58900600	-0.00001300
C	-1.04558800	1.78406600	-0.00002600
H	1.67319200	2.19512500	-0.00000100
C	-2.35145500	1.37260100	0.00003900
C	-2.62372600	-0.02405600	0.00030200
H	-0.79840000	2.84010600	-0.00012200
H	-3.17176900	2.07897400	-0.00004100
N	-1.69195900	-0.95797600	0.00016400
C	-3.99622000	-0.46446600	-0.00005000
N	-5.10868500	-0.77400400	-0.00033100
O	3.62153000	0.58530100	-0.00007300
C	4.66956300	-0.38010600	-0.00012500
H	4.63359600	-1.00902900	-0.89525800
H	5.59467000	0.19314000	-0.00068700
H	4.63416900	-1.00824400	0.89562900

(20)

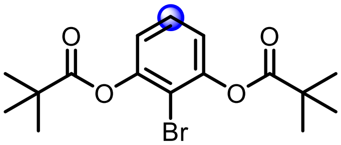

C	1.18436300	-2.46989600	0.22826900
C	-0.00003600	-3.16134100	-0.00003600
C	-1.18441000	-2.46984700	-0.22830600
C	-1.18381500	-1.08022400	-0.22464000
C	0.00001400	-0.37324600	0.00003300
C	1.18381800	-1.08027700	0.22467200
H	2.11460000	-2.99432100	0.40517600
H	-0.00005100	-4.24439900	-0.00006200
H	-2.11466600	-2.99422700	-0.40523900
Br	0.00004800	1.52899800	0.00007900
O	2.34604000	-0.38301600	0.51963600
O	-2.34599500	-0.38288700	-0.51957200
C	3.35505600	-0.33926000	-0.42357900
O	3.25512900	-0.86075100	-1.49845300
C	-3.35515800	-0.33938600	0.42350000
O	-3.25541500	-0.86119000	1.49823900
C	4.55717700	0.44446500	0.10516300
C	-4.55717100	0.44452800	-0.10520600
C	-5.05048400	-0.20422600	-1.41729400
H	-5.34399000	-1.24609500	-1.25942100
H	-5.92748500	0.33817700	-1.78089400
H	-4.28228700	-0.17489700	-2.19119400
C	-4.12477200	1.90350800	-0.37254500
H	-4.98443800	2.47298500	-0.73664200
H	-3.76490000	2.38318500	0.54151400
H	-3.33256500	1.95390800	-1.12039200
C	-5.67011700	0.40895400	0.95302800
H	-5.99004000	-0.61434700	1.16064400
H	-5.33784500	0.84951900	1.89476500
H	-6.53389000	0.97380800	0.59233300
C	4.12483300	1.90333700	0.37316700
H	3.33279500	1.95344100	1.12121200
H	4.98457600	2.47268400	0.73728800
H	3.76475100	2.38336500	-0.54062600
C	5.05078200	-0.20480100	1.41689000
H	5.92786000	0.33746600	1.78050600
H	4.28275600	-0.17577700	2.19097100
H	5.34425900	-1.24660500	1.25854200
C	5.66989800	0.40930800	-0.95332100
H	5.98977200	-0.61391000	-1.16141800
H	5.33742700	0.85025300	-1.89481000
H	6.53375000	0.97401400	-0.59258100

(21)

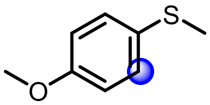

C	0.92880000	-1.41060500	0.06479900
C	-0.44082500	-1.27064000	0.22535600
C	-1.02721600	0.00072600	0.30987800
C	-0.20238200	1.12294500	0.24717900
C	1.17950500	0.99553100	0.09115400
C	1.74923300	-0.27675600	-0.00448200
H	1.38907700	-2.38921300	-0.00100600
H	-1.06699700	-2.15261000	0.29368100
H	-0.64046200	2.11109200	0.32538100
H	1.79022100	1.88737200	0.04904200
S	-2.79680500	0.18258700	0.55078000
C	-3.39565600	0.08311800	-1.18157000
H	-3.14429300	-0.88014500	-1.62615900
H	-4.48123000	0.18534300	-1.13816600
H	-2.98157100	0.89301900	-1.78260200
O	3.08157600	-0.51814200	-0.15995000
C	3.97164300	0.58813600	-0.23366900
H	4.96535300	0.16145300	-0.35880500
H	3.74165700	1.22922200	-1.09144600
H	3.94590200	1.18347500	0.68532900

(22)

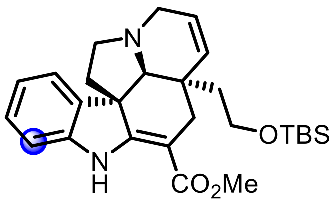

N	-2.90499600	1.91554600	-0.14873100
C	-2.84233100	-0.41729200	-0.22129300
C	-2.17296800	0.87308800	-0.67568600
H	-2.58596400	2.86621000	-0.26672700
C	-3.86609300	-0.92265800	-1.31702400
H	-3.53589500	-0.60015000	-2.30610700
H	-4.86842500	-0.53493500	-1.13527700
C	-3.75786800	-2.44316300	-1.21710200
H	-4.35285800	-2.83226400	-0.36812800
H	-4.09919700	-2.95243300	-2.12320500
N	-2.33090600	-2.60548300	-1.03973200
C	-1.86930500	-1.62838600	-0.05403200
H	-1.97363400	-2.02207800	0.97302200
C	-0.37444000	-1.31876200	-0.31256100
C	-1.01303800	0.89430200	-1.38707300
C	-0.28781700	-0.42497600	-1.58413100
H	0.74978500	-0.22086200	-1.84712600
H	-0.71245400	-0.99785000	-2.41391800
C	0.34128600	-2.64333900	-0.53343500
H	1.42166600	-2.60700500	-0.45979800
C	-0.27545000	-3.80837600	-0.73641200
H	0.30163100	-4.72422200	-0.82484600
C	-1.76983900	-3.92571600	-0.86820200
H	-2.19026000	-4.45656100	0.01279300
H	-2.02045100	-4.53627200	-1.74386100
C	-0.34505700	2.11061800	-1.83760600
O	0.74548500	2.16104500	-2.37259400
O	-1.06549100	3.25977300	-1.59606700
C	-0.45085100	4.48120400	-2.04163500
H	0.50023400	4.64319200	-1.53239900
H	-0.27720400	4.45127500	-3.11786200
H	-1.15655000	5.27208400	-1.79266200
C	0.22033000	-0.62584600	0.94947500
H	0.29943600	-1.37389300	1.74561100
H	-0.48494900	0.13046400	1.31094100
C	1.56691500	0.08422800	0.81604000
H	1.75859900	0.60981200	1.76105800
H	1.52457000	0.84261300	0.02626900
O	2.62664000	-0.83427900	0.56416200
Si	4.18381400	-0.50017800	0.01789600
C	4.10187300	0.35431600	-1.65966000
H	3.70026900	-0.33167100	-2.41158200
H	3.45750200	1.23737300	-1.65419900
H	5.09478100	0.66744000	-1.99747200
C	4.95807900	-2.20499800	-0.15180000
H	5.96329400	-2.15248700	-0.57968300
H	5.02570600	-2.71698100	0.81174500
H	4.34842400	-2.82624900	-0.81433600
C	5.14690900	0.57536500	1.29073900
C	4.98000000	-0.01166700	2.70795300
H	5.53647900	0.59140400	3.43709200
H	3.93296900	-0.02938600	3.02307500
H	5.36030900	-1.03552600	2.77444000
C	4.63714900	2.03285000	1.28407800
H	5.20240500	2.63336600	2.00874400
H	4.75814800	2.50435700	0.30457300
H	3.58109800	2.10481800	1.55902700
C	6.64805500	0.58236200	0.92591100
H	7.20624100	1.20391900	1.63779800
H	7.08360600	-0.42042200	0.95853900
H	6.82872600	0.99414000	-0.07230700
C	-5.04863400	0.13185800	2.92578200
C	-5.10572200	1.52188400	2.84194300
C	-4.42476800	2.21384200	1.83426900
C	-3.62596900	0.06620600	0.98954600
C	-4.30994100	-0.60549400	1.98892800
H	-5.57603200	-0.38276900	3.72023200
H	-5.68100500	2.08054800	3.57154300
H	-4.46591000	3.29562300	1.77575000
H	-4.27260800	-1.68689600	2.05937800
C	-3.68677600	1.46722200	0.92541600

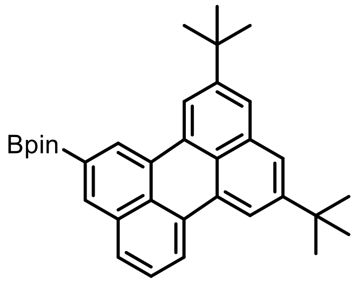

(23)

C	2.41971600	4.14480700	-0.00000600
C	1.23143200	4.83299000	0.00012700
C	-0.00000300	4.13224800	0.00011300
C	-0.00000200	2.69826500	0.00005900
C	1.25026800	1.99874200	-0.00001800
C	2.42512200	2.73977700	-0.00010200
H	-1.21728400	5.91753000	0.00016500
H	3.36273400	4.67994900	-0.00005000
H	1.21727500	5.91753200	0.00020600
C	-1.23143900	4.83298800	0.00008600
C	-1.25027200	1.99874000	-0.00004600
H	3.38398800	2.23900700	-0.00027000
C	-2.42512700	2.73977300	-0.00017600
C	-2.41972300	4.14480300	-0.00008700
H	-3.38399300	2.23900200	-0.00037700
H	-3.36274200	4.67994400	-0.00016600
C	2.41948900	-0.22284000	0.00003800
C	1.24950200	0.52185500	-0.00001500
C	0.00000000	-0.16881200	-0.00007000
C	0.00000100	-1.59291900	-0.00008200
C	1.23118900	-2.29661800	-0.00009100
C	2.44231300	-1.63802800	-0.00001500
H	3.36831600	0.29563200	0.00015000
C	-1.24950400	0.52185300	-0.00002100
C	-1.23118700	-2.29662000	-0.00008400
H	1.18596600	-3.37820600	-0.00012200
C	-2.44231100	-1.63803100	0.00000100
C	-2.41949000	-0.22284400	0.00004500
H	-1.18596100	-3.37820800	-0.00011500
H	-3.36831800	0.29562600	0.00016100
C	-3.79638600	-2.36995200	0.00004100
C	3.79639000	-2.36994600	0.00001800
C	3.63132500	-3.90017400	-0.00002000
H	4.61601300	-4.37548200	0.00006200
H	3.09594200	-4.25133300	0.88630600
H	3.09609700	-4.25129700	-0.88645000
C	4.59738800	-1.97318100	1.26233600
H	5.56521900	-2.48405100	1.27253700
H	4.78887800	-0.89845900	1.30413100
H	4.05620200	-2.25136000	2.17085400
C	4.59747100	-1.97312000	-1.26224000
H	4.05629700	-2.25118500	-2.17080100
H	4.78903400	-0.89840900	-1.30393000
H	5.56525300	-2.48407200	-1.27243200
C	-4.59737400	-1.97320700	1.26237100
H	-4.78887100	-0.89848700	1.30418100
H	-5.56520100	-2.48408300	1.27257600
H	-4.05617700	-2.25139300	2.17088000
C	-4.59747900	-1.97311100	-1.26220500
H	-5.56526400	-2.48406000	-1.27239200
H	-4.78903900	-0.89839900	-1.30388100
H	-4.05631600	-2.25116800	-2.17077500
C	-3.63131500	-3.90017900	-0.00001900
H	-3.09610100	-4.25129000	-0.88646200
H	-3.09591600	-4.25134700	0.88629300
H	-4.61600100	-4.37549100	0.00007300

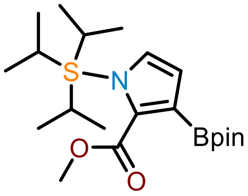

(24)

C	-1.72252100	0.99732800	-0.04372200
C	-2.28070200	2.26781200	-0.05127200
C	-1.22659100	3.19285100	0.04402900
C	-0.06068200	2.45630700	0.10320200
N	-0.32690300	1.10594400	0.05002800
H	-3.33703500	2.47323600	-0.11444100
H	-1.29590200	4.26938000	0.06695200
H	0.94790100	2.82126900	0.17542200
Si	1.01875800	-0.16065400	0.11023300
C	2.63364400	0.88029600	-0.07317100
H	2.52646200	1.71794300	0.62632900
C	1.09068600	-0.95997300	1.84611000
H	2.08328800	-1.43262200	1.84493900
C	0.80487400	-1.43680400	-1.29369300
H	-0.00008800	-2.08103100	-0.93429700
C	1.09470800	0.10628400	2.96099500
H	0.13774800	0.63472900	3.00485300
H	1.25559500	-0.36197600	3.93838400
H	1.87924000	0.85673600	2.82693900
C	0.07759900	-2.07130600	2.17006500
H	0.34175300	-2.54594400	3.12238700
H	-0.93659200	-1.68045000	2.26017000
H	0.05205900	-2.84868400	1.40381200
C	3.90727700	0.13105500	0.37864200
H	4.10496700	-0.75603000	-0.22749800
H	4.77836100	0.78855100	0.27951900
H	3.85815500	-0.18349400	1.42330600
C	2.85246800	1.47484100	-1.48010300
H	1.98453000	2.02665400	-1.84933600
H	3.70533100	2.16319700	-1.47636900
H	3.07654600	0.69107000	-2.20831600
C	2.05383300	-2.32250200	-1.48177700
H	2.39496800	-2.77029300	-0.54344800
H	1.82839800	-3.14590000	-2.16877200
H	2.89283800	-1.76799300	-1.91162000
C	0.34105800	-0.85067900	-2.64097000
H	0.11953600	-1.65998500	-3.34604700
H	-0.56513400	-0.24961600	-2.53864900
H	1.10584000	-0.22031700	-3.10248400
C	-2.44234700	-0.26500900	-0.09391700
O	-1.95020000	-1.37474600	-0.01285100
O	-3.77756700	-0.07404200	-0.23839300
C	-4.57802100	-1.26546100	-0.27449400
H	-5.60375400	-0.92027200	-0.38742800
H	-4.29285900	-1.89675000	-1.11764800
H	-4.46602200	-1.83380100	0.65031100

(S1)

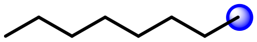

C	-0.16333600	4.50331300	0.00000000
H	-0.90278400	5.30946600	0.00000000
H	0.47041800	4.63445400	0.88310800
H	0.7041800	4.63445400	-0.88310800
C	-0.83478700	3.12680000	0.00000000
H	-1.48919600	3.04074700	-0.87630900
H	-1.48919600	3.04074700	0.87630900
C	0.16333600	1.96342900	0.00000000
H	0.81853100	2.05092400	0.87688400
H	0.81853100	2.05092400	-0.87688400
C	-0.49954700	0.58133500	0.00000000
H	-1.15468500	0.49499400	-0.87687000
H	-1.15468500	0.49499400	0.87687000
C	0.49954700	-0.58133500	0.00000000
H	1.15468500	-0.49499400	0.87687000
H	1.15468500	-0.49499400	-0.87687000
C	-0.16333600	-1.96342900	0.00000000
H	-0.81853100	-2.05092400	-0.87688400
H	-0.81853100	-2.05092400	0.87688400
C	0.83478700	-3.12680000	0.00000000
C	0.16333600	-4.50331300	0.00000000
H	1.48919600	-3.04074700	0.87630900
H	1.48919600	-3.04074700	-0.87630900
H	0.90278400	-5.30946600	0.00000000
H	-0.47041800	-4.63445400	0.88310800
H	-0.47041800	-4.63445400	-0.88310800

(S2)

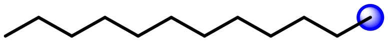

C	-0.64117800	-0.11495100	0.33275300
C	0.64504500	0.62319300	0.16324600
H	0.60901800	1.21995800	-0.76056600
H	0.75926500	1.36952400	0.97168400
H	-0.62083900	-1.08887000	0.81621100
C	-1.96072400	0.55161400	0.12684000
H	-2.12395600	1.31261000	0.91285900
H	-1.94117900	1.12536400	-0.81215400
C	-3.15828800	-0.40851000	0.11691100
H	-3.01636800	-1.14913900	-0.67945800
H	-3.17320000	-0.97138800	1.05880400
C	-4.50571800	0.29502000	-0.07435300
H	-4.48984400	0.85989200	-1.01575200
C	-5.70301600	-0.66219000	-0.08255000
H	-5.71978900	-1.22616100	0.85809600
H	-5.56569600	-1.40441200	-0.87842900
C	-7.04601400	0.04838800	-0.27443500
H	-7.87763200	-0.66185100	-0.27665500
H	-7.22925600	0.77131300	0.52690500
H	-7.07314100	0.59393700	-1.22310100
H	-4.64382000	1.03818000	0.72210900
C	1.88980100	-0.27511700	0.14609400
H	1.91820700	-0.86458400	1.07124100
H	1.79779500	-0.99765100	-0.67379400
C	3.20297800	0.50060800	-0.00127500
H	3.17121700	1.09435900	-0.92416500
H	3.29124900	1.22291600	0.82083800
C	4.44597000	-0.39585200	-0.02122100
H	4.47906700	-0.99020300	0.90131300
H	4.35865100	-1.11809300	-0.84349400
C	5.76120300	0.37778700	-0.16884400
H	5.84968700	1.09862900	0.65324800
H	5.72839300	0.97171300	-1.09045400
C	6.99714400	-0.52637000	-0.18882200
H	7.91707000	0.05541500	-0.29520700
H	7.07698900	-1.10751400	0.73540500
H	6.95488800	-1.23566000	-1.02155100

(S3)

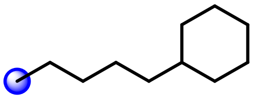

C	-1.05399200	-0.28139200	0.23214000
C	-2.10611200	-1.35393100	-0.10742400
C	-3.51794600	-0.96177400	0.35012900
C	-3.93640700	0.40096900	-0.21793400
C	-2.90143300	1.48232600	0.11888800
C	-1.49036700	1.08214700	-0.33731500
H	-4.23676800	-1.73362500	0.05519500
H	-2.11029800	-1.51439000	-1.19465900
H	-1.81720600	-2.30951400	0.34452600
H	-1.01970300	-0.18888300	1.32870600
H	-4.03503700	0.32282100	-1.30875100
H	-4.92249300	0.68591600	0.16388100
H	-3.18571000	2.43583800	-0.33875000
H	-2.89473000	1.64913500	1.20411100
H	-1.46670200	1.02782100	-1.43507400
H	-0.77825100	1.86171300	-0.05017200
H	-3.54251700	-0.91739900	1.44695800
C	0.34490000	-0.71056700	-0.24362500
H	0.52319700	-1.74046900	0.09172100
H	0.35297400	-0.74536200	-1.34170000
C	1.50307300	0.16846900	0.24589200
H	1.38064100	1.19242300	-0.12522000
H	1.46658700	0.23446700	1.34140700
C	2.88057200	-0.35245500	-0.18068200
H	2.91724700	-0.42580400	-1.27557000
C	4.04558100	0.51771200	0.30468800
H	4.00681200	0.59401800	1.39829900
H	3.91598300	1.53850100	-0.07523500
C	5.41775200	-0.01359300	-0.12005500
H	6.22539300	0.62874000	0.24220800
H	5.59173500	-1.01976800	0.27461400
H	5.50096100	-0.06837900	-1.21020400
H	3.01415100	-1.37527700	0.19550300

(S4)

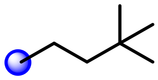

C	0.50529900	0.58447500	-0.00063700
C	1.65606500	-0.43044800	-0.00010100
H	0.61734200	1.23645100	-0.87728400
H	0.61760200	1.23776000	0.87500200
C	3.03044500	0.24816800	0.00002400
H	1.58101500	-1.08266700	0.87648500
H	1.58135800	-1.08308100	-0.87641800
H	3.83889100	-0.48842300	0.00029500
H	3.15944000	0.88230300	-0.88291200
H	3.15911400	0.88260300	0.88279300
C	-1.20408300	-0.82340800	-1.25777900
C	-1.91104500	1.22514700	-0.00069400
H	-0.57709200	-1.71851700	-1.28390600
H	-2.24737200	-1.15318400	-1.29024600
H	-1.00855600	-0.24916500	-2.16936500
H	-1.76188000	1.85318100	0.88334400
H	-1.76192900	1.85204600	-0.88554800
H	-2.95256800	0.88869500	-0.00045600
C	-0.94210700	0.02699800	0.00001000
C	-1.20360600	-0.82160200	1.25911900
H	-2.24706000	-1.15071200	1.29284800
H	-0.57709200	-1.71702000	1.28593000
H	-1.00701700	-0.24625100	2.16978100

(S5)

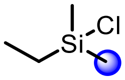

Si	-0.09284900	0.23504400	0.00000000
Cl	-1.93676100	-0.79687200	0.00000000
C	-0.05359700	1.29039200	1.55137900
H	-0.11625700	0.67248200	2.45095400
H	0.87342500	1.87055700	1.60291300
H	-0.89002300	1.99398700	1.56740300
C	1.25657600	-1.08176400	0.00000000
H	1.10049100	-1.72424500	-0.87389800
H	1.10049100	-1.72424500	0.87389800
C	-0.05359700	1.29039200	-1.55137900
H	-0.89002300	1.99398700	-1.56740400
H	0.87342500	1.87055700	-1.60291300
H	-0.11625700	0.67248200	-2.45095400
C	2.69567000	-0.53113200	0.00000000
H	2.89590500	0.08356000	0.88244900
H	3.42742400	-1.34380600	0.00000000
H	2.89590500	0.08356000	-0.88244900

(S6)

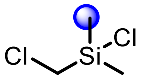

Si	0.03738800	0.51696800	0.00000000
Cl	-1.78435800	1.57505600	0.00000000
C	0.96545500	0.95148300	1.56253600
H	0.36797500	0.73218200	2.45089800
H	1.89451300	0.37773800	1.62569000
H	1.21827200	2.01483900	1.57881600
C	-0.49798000	-1.30201200	0.00000000
H	-1.08058200	-1.55932800	-0.88418700
H	-1.08058200	-1.55932800	0.88418700
C	0.96545500	0.95148300	-1.56253600
H	1.21827200	2.01483900	-1.57881600
H	1.89451300	0.37773800	-1.62569000
H	0.36797500	0.73218200	-2.45089800
Cl	0.96545500	-2.39706400	0.00000000

(S7)

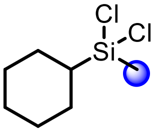

Si	-1.17407800	0.00001600	0.24011600
Cl	-2.11559800	1.67844000	-0.57641100
C	-1.48179600	-0.00005000	2.08016500
H	-2.55401700	-0.00038200	2.28760700
H	-1.04339600	0.88786700	2.54350600
H	-1.04280000	-0.88753500	2.54375700
Cl	-2.11452200	-1.67886200	-0.57679600
C	0.63232800	0.00023400	-0.29439100
C	1.38336900	1.27304500	0.16502200
C	1.38323700	-1.27251700	0.16575300
H	0.60693400	-0.00018300	-1.39359600
C	2.84573100	1.26704000	-0.30873600
H	1.36840900	1.33223100	1.26163800
H	0.87807100	2.16987900	-0.20426500
C	2.84558000	-1.26694800	-0.30810600
H	1.36834100	-1.33096400	1.26239300
H	0.87780300	-2.16948600	-0.20302200
C	3.58516600	0.00010800	0.13990800
H	3.35803600	2.15933700	0.06569300
H	2.86643400	1.33139800	-1.40410400
H	3.35778600	-2.15912000	0.06676300
H	2.86623500	-1.33187300	-1.40344100
H	4.60722900	-0.00005800	-0.25221500
H	3.67237400	0.00037500	1.23449700

(S8)

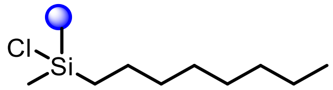

Si	3.26505700	0.28934100	0.00000000
Cl	4.80387800	-1.15987500	0.00000000
C	3.48822400	1.32385400	-1.55019700
H	3.39933200	0.71105300	-2.45105000
H	2.73129100	2.11320600	-1.60134400
H	4.47181400	1.80070400	-1.56324200
C	1.63657000	-0.65900400	0.00000000
H	1.63131100	-1.32069700	0.87478500
H	1.63131100	-1.32069700	-0.87478500
C	3.48822400	1.32385400	1.55019700
H	4.47181400	1.80070400	1.56324200
H	2.73129100	2.11320600	1.60134400
H	3.39933200	0.71105300	2.45105000
C	0.36841800	0.21693200	0.00000000
H	0.36993800	0.87667000	-0.87653400
H	0.36993800	0.87667100	0.87653400
C	-0.93095300	-0.59993600	0.00000000
H	-0.93917500	-1.25974600	0.87681800
H	-0.93917500	-1.25974600	-0.87681800
C	-2.19731500	0.26349900	0.00000000
H	-2.18556500	0.92374200	-0.87699200
H	-2.18556500	0.92374200	0.87699200
C	-3.49577100	-0.55164700	0.00000000
H	-3.50799300	-1.21192400	0.87679600
H	-3.50799300	-1.21192400	-0.87679600
C	-4.76234500	0.31150300	0.00000000
H	-4.75079400	0.97213200	-0.87684900
H	-4.75079400	0.97213200	0.87684900
C	-6.06200500	-0.50156500	0.00000000
H	-6.07435100	-1.16118600	0.87625200
H	-6.07435100	-1.16118600	-0.87625200
C	-7.32123700	0.37009000	0.00000000
H	-7.35481900	1.01628300	-0.88293700
H	-8.22956700	-0.23886300	0.00000000
H	-7.35481900	1.01628300	0.88293700

(S9)

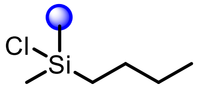

Si	1.08371800	0.28405000	0.00000000
Cl	2.66057900	-1.12333200	0.00000000
C	1.27811800	1.32410000	-1.55026300
H	1.20623900	0.70892200	-2.45101400
H	0.49948100	2.09205600	-1.60122300
H	2.24803700	1.82816800	-1.56340300
C	-0.51844700	-0.70830500	0.00000000
H	-0.50560100	-1.36989200	0.87482200
H	-0.50560100	-1.36989200	-0.87482200
C	1.27811800	1.32410000	1.55026300
H	2.24803700	1.82816800	1.56340300
H	0.49948100	2.09205600	1.60122300
H	1.20623900	0.70892200	2.45101400
C	-1.81043300	0.13174700	0.00000000
H	-1.82847500	0.79140200	-0.87663900
H	-1.82847500	0.79140200	0.87663900
C	-3.08682400	-0.72091100	0.00000000
H	-3.07577500	-1.37989900	0.87629400
H	-3.07577500	-1.37989900	-0.87629400
C	-4.37068600	0.11353300	0.00000000
H	-4.42454900	0.75794600	-0.88323400
H	-5.25967900	-0.52305900	0.00000000
H	-4.42454900	0.75794600	0.88323400

(S10)

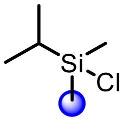

Si	-0.34619100	0.37375300	0.09754800
Cl	-1.84138000	-1.04131700	-0.39168100
C	-0.79899300	1.95935800	-0.80073000
H	-1.80625300	2.28365100	-0.52632000
H	-0.10569500	2.76701500	-0.54610100
H	-0.77537200	1.82064500	-1.88491400
C	-0.40304800	0.60238300	1.96024100
H	-0.20754100	-0.33564700	2.48540300
H	-1.38601800	0.96333000	2.27415300
C	1.30682300	-0.33443800	-0.50856300
H	1.16560100	-0.50966400	-1.58359300
H	0.34273300	1.33549000	2.28430400
C	1.66627900	-1.67966900	0.14781100
H	0.88165500	-2.42687400	0.00736100
H	2.58993900	-2.08238700	-0.28188600
H	1.83342500	-1.56725200	1.22408500
C	2.45271200	0.68516700	-0.34236800
H	3.38661900	0.28068200	-0.74704300
H	2.25329100	1.62595500	-0.86304600
H	2.63511800	0.91811200	0.71215600

(S11)

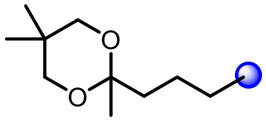

C	-2.30407100	0.65837200	-0.04619200
C	-3.37223100	-0.24147100	-0.68827000
H	-4.22709300	-0.36517800	-0.01614700
H	-3.73930400	0.19826600	-1.62094900
H	-2.97046700	-1.22993800	-0.91356400
C	-2.88632200	2.04418200	0.25923100
H	-3.73379800	1.96783400	0.94749100
H	-2.14244700	2.70497500	0.71562900
H	-3.24746800	2.52622100	-0.65460300
C	-1.77178300	0.02158700	1.25089100
H	-1.15805800	0.74921900	1.79994700
H	-2.59246400	-0.27944300	1.90672600
C	-1.08864200	0.79980400	-0.98090700
H	-1.39812800	1.09292500	-1.98706000
H	-0.41036000	1.57663500	-0.59989600
O	-0.39553300	-0.43671200	-1.14061100
O	-1.03364800	-1.17215700	0.99647600
C	0.05174100	-1.04491400	0.07164800
C	1.21571200	-0.26201000	0.71834100
C	2.44141900	-0.04833400	-0.17810400
H	1.50001000	-0.80768900	1.62402600
H	0.85735300	0.71455000	1.05697100
C	3.52090400	0.81085200	0.49211700
H	2.87828900	-1.01488900	-0.45232800
H	2.12837600	0.42208400	-1.11654900
H	3.09380300	1.78589200	0.75773400
H	3.82080100	0.34394300	1.43832100
C	4.75730100	1.01970100	-0.38702800
H	4.49448300	1.51214900	-1.32859000
H	5.23179200	0.06516000	-0.63566600
C	0.43380100	-2.47012500	-0.29794200
H	1.20225700	-2.47255100	-1.07116300
H	0.80347300	-3.00000700	0.58186300
H	-0.44918400	-2.98537600	-0.67707600
H	5.50461400	1.64030800	0.11525200

(S12)

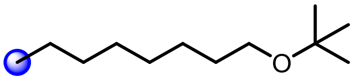

C	-0.72070200	0.07189200	-0.40389200
C	0.56564700	-0.66542400	-0.23624900
H	0.52889100	-1.30510300	0.65446800
H	0.73536500	-1.35438400	-1.08199400
H	-0.71190800	1.01949800	-0.93812800
C	-2.03591600	-0.58650300	-0.15049400
H	-2.20857800	-1.37952300	-0.90186900
H	-2.00020300	-1.12039800	0.81083800
C	-3.23528300	0.37110100	-0.16316200
H	-3.08470400	1.14396400	0.60031000
H	-3.26534900	0.89472000	-1.12710300
C	-4.57739000	-0.32817300	0.07571100
H	-4.54568800	-0.85482900	1.03858500
C	-5.77803400	0.62476500	0.06314200
H	-5.81030100	1.15108400	-0.89869600
H	-5.63253100	1.39815800	0.82722300
C	-7.11528800	-0.08259100	0.30159900
H	-7.94981700	0.62405200	0.28682900
H	-7.30603400	-0.83756200	-0.46775800
H	-7.12725800	-0.58995300	1.27151100
H	-4.72414600	-1.10297300	-0.68829700
C	1.78061000	0.25368400	-0.13725700
H	1.82069200	0.90576700	-1.02059800
H	1.68651900	0.89557100	0.74806700
C	4.22737600	0.06408600	0.10951300
C	5.20361400	-1.11495200	0.10212000
H	5.13234900	-1.65800900	-0.84303300
H	4.96353800	-1.80737300	0.91213800
O	2.93729100	-0.56683000	-0.05805900
H	6.23243500	-0.76878300	0.23069500
C	4.53376700	1.01295500	-1.05926900
H	4.41802200	0.49083400	-2.01264000
H	3.87846700	1.88750000	-1.06146400
H	5.56301400	1.37522100	-0.99003300
C	4.30397500	0.80513000	1.45350900
H	4.03341200	0.13290500	2.27172400
H	5.32125100	1.16601900	1.62805200
H	3.63997800	1.67241700	1.48402500

(S13)

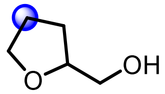

C	0.30389100	-0.23772900	0.44294800
O	-0.69287700	-1.19098400	0.04103900
C	-1.96973600	-0.55595200	0.06109900
C	-1.69623700	0.87454800	-0.39927900
C	-0.33510700	1.16656900	0.26480400
H	0.56631300	-0.40828000	1.49463900
H	-2.63222200	-1.12109500	-0.59621500
H	-2.38771400	-0.57114200	1.07875800
H	-1.61165600	0.90243000	-1.48871800
H	-2.47553800	1.57846400	-0.10014800
H	0.28325400	1.83002400	-0.34709200
H	-0.47087300	1.65264400	1.23337500
C	1.54716200	-0.49445200	-0.40643800
H	1.33246600	-0.26168000	-1.45797100
H	1.80898900	-1.55142900	-0.34322500
H	2.55043400	1.16380500	-0.09511400
O	2.68496600	0.22452900	0.06432400

(S14)

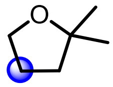

C	0.63798000	0.01913200	-0.02060700
O	-0.20688700	-0.80324500	0.83292000
C	-1.56615100	-0.73812000	0.40209400
C	-1.70320100	0.61578900	-0.29190200
C	-0.34133700	0.73206600	-0.99095400
H	-1.78637700	-1.56024700	-0.29533200
H	-2.20351400	-0.85504400	1.28120700
H	-2.54714400	0.65790000	-0.98405800
H	-1.83169300	1.40927700	0.44992900
H	-0.36722700	0.20805100	-1.95130400
H	-0.04311800	1.76416900	-1.18558600
C	1.62461100	-0.89506000	-0.74933500
H	2.26116300	-0.31962700	-1.42903200
H	2.26640800	-1.41283500	-0.03216600
H	1.08950800	-1.64737300	-1.33448300
C	1.37243200	1.00526600	0.89142300
H	1.91106500	0.46221300	1.67167000
H	2.09316900	1.60146400	0.32351300
H	0.66685000	1.68358100	1.37796900

(S15)

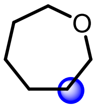

C	0.30646600	1.50474800	0.41574100
C	1.51394300	0.72600000	-0.13264200
C	1.46824100	-0.78872500	0.06877700
C	-0.99539700	1.23739200	-0.36074700
C	-1.74078800	-0.06945700	-0.01822400
C	-0.85702600	-1.26666200	0.35756800
H	0.16987100	1.29504200	1.48476900
H	1.60838000	0.93719100	-1.20432700
H	1.55733800	-1.02844300	1.13992800
H	-0.75461600	1.24109300	-1.42902800
H	-2.37407700	-0.33971800	-0.86913200
H	0.53571800	2.57343900	0.34768800
H	2.43498900	1.09008800	0.34016500
H	2.31297100	-1.25525700	-0.44467100
H	-1.68738700	2.07185800	-0.20686200
H	-2.42017300	0.09978200	0.82671300
H	-1.43311400	-2.19065600	0.25246700
H	-0.54961500	-1.19508900	1.40996900
O	0.30338500	-1.41988800	-0.45381500

(S16)

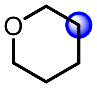

C	-1.25921800	0.69693700	-0.22145800
C	-1.18373800	-0.77118800	0.19928700
C	1.18377600	-0.77113600	0.19928300
C	1.25918500	0.69699900	-0.22145300
C	-0.00003600	1.44942800	0.23308900
H	-1.21811700	-0.85046500	1.29851900
H	-2.01704500	-1.34785800	-0.20652400
H	-1.34842400	0.74736400	-1.31249500
H	-2.16163100	1.15280400	0.20017200
H	1.21816700	-0.85042000	1.29851300
H	2.01710700	-1.34776200	-0.20654000
H	2.16157300	1.15290600	0.20018500
H	1.34839400	0.74743700	-1.31249000
H	-0.00004000	1.52787700	1.32809400
H	-0.00005900	2.47252600	-0.15456400
O	0.00003200	-1.40133100	-0.28317000

(S17)

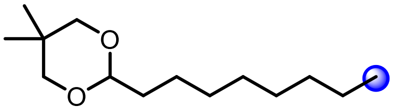

C	-4.69651600	0.38791000	0.09128100
C	-4.90288100	0.23316000	1.60672900
H	-5.15238100	1.19631200	2.06282700
H	-5.72358800	-0.45979700	1.81658800
H	-4.00247300	-0.14924400	2.08892400
C	-5.96954700	0.93009100	-0.57063300
H	-6.24013400	1.90547800	-0.15454600
H	-5.84565000	1.04892200	-1.65168800
H	-6.81451400	0.25535900	-0.40182400
C	-3.51675900	1.33787400	-0.19496800
H	-3.47697100	1.56993200	-1.27090800
H	-3.62023700	2.27698200	0.35325800
C	-4.31968900	-0.96755900	-0.54024800
H	-5.02505800	-1.75002900	-0.25180200
H	-4.32974100	-0.88291000	-1.63858100
O	-3.03290900	-1.41070400	-0.11332200
O	-2.27230600	0.77098100	0.21224000
C	-2.02350600	-0.46865300	-0.42187800
H	-2.02370000	-0.30466400	-1.51646900
C	-0.69117100	-1.01276500	0.05521900
C	0.50442400	-0.14068800	-0.34008500
H	-0.58347000	-2.01946800	-0.36087700
H	-0.74698200	-1.11792600	1.14349100
C	1.84225200	-0.70928500	0.14610500
H	0.53316600	-0.03521200	-1.43262500
H	0.36375300	0.86676100	0.06393000
H	1.81555700	-0.80868200	1.23885800
H	1.97155800	-1.72587500	-0.24781600
C	3.05055000	0.14469800	-0.25396900
H	2.91739300	1.16274500	0.13420300
H	3.08076100	0.23872500	-1.34737400
C	4.38900700	-0.41417300	0.24186800
H	4.52045300	-1.43384100	-0.14320000
H	4.35941700	-0.50492500	1.33548700
C	5.59834600	0.43689700	-0.16109300
H	5.46667600	1.45759400	0.22141400
H	5.63079000	0.52562800	-1.25500800
C	6.93700100	-0.11853000	0.33829600
C	8.13909500	0.73840700	-0.06934900
H	7.06950200	-1.13811600	-0.04403100
H	6.90545900	-0.20635100	1.43121900
H	9.07668700	0.31559100	0.30250000
H	8.21878700	0.81549100	-1.15843400
H	8.05302300	1.75501100	0.32749500

(S18)

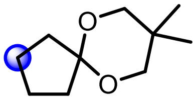

C	1.17008500	0.51623400	1.16025600
C	2.05116800	0.05748000	-0.01574700
C	1.15127400	0.11504100	-1.26261800
H	0.96619800	1.59325600	1.07088800
H	1.66920400	0.34898700	2.11772900
H	0.91927500	1.16183100	-1.50906500
H	1.64378100	-0.33321800	-2.12876900
O	-0.05232200	-0.62627100	-1.07163500
O	-0.05319900	-0.21450400	1.23175800
C	3.23244100	1.02217400	-0.18190000
H	3.86431900	1.02367100	0.71187100
H	3.86135700	0.72733000	-1.02773800
H	2.89648600	2.04944600	-0.35608800
C	2.55729400	-1.37527500	0.21589800
H	3.16101100	-1.71396900	-0.63193300
H	3.18180300	-1.42424000	1.11334700
H	1.72594600	-2.06985900	0.34073400
C	-0.83266300	-0.19007600	0.03755300
C	-1.50674200	1.19507600	-0.21731200
C	-1.98850900	-1.19297600	0.22014200
C	-2.98588700	0.99335000	0.15718000
H	-1.42503000	1.43139400	-1.28199100
H	-1.03598800	2.00437800	0.34274100
C	-3.25849300	-0.46442700	-0.24342600
H	-2.05031500	-1.43080100	1.28471400
H	-1.77563500	-2.11290600	-0.32483700
H	-3.11896300	1.11144000	1.23803400
H	-3.64569600	1.71226800	-0.33479800
H	-4.16706700	-0.87309900	0.20502100
H	-3.36631300	-0.53931700	-1.33098300

(S19)

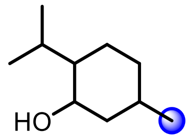

C	0.25850900	-1.57203200	-0.05229800
C	-0.62972600	-0.35687500	-0.39750500
C	-0.07037300	0.88622400	0.30976000
C	1.40764600	1.11535700	-0.02556300
C	2.29252900	-0.09041700	0.32570000
C	1.74125200	-1.34421300	-0.36878600
H	-0.53099000	-0.17257100	-1.47629400
H	0.15052300	-1.81400200	1.01295000
H	-0.10785400	-2.44450800	-0.60267300
H	1.49034700	1.33455200	-1.09788100
H	1.76348000	2.00659600	0.50869900
H	2.22531600	-0.25325700	1.41122800
H	1.86968500	-1.23356000	-1.45418400
H	2.32662200	-2.22254000	-0.07451400
C	3.76239900	0.16425400	-0.02254000
H	4.15019500	1.04342300	0.50105600
H	4.38775300	-0.69061100	0.25197000
H	3.88590000	0.33522800	-1.09731700
C	-2.13207600	-0.66435700	-0.12028900
H	-2.26366900	-1.72169000	-0.38726100
C	-2.53252000	-0.52126300	1.35655600
H	-3.54960200	-0.89394300	1.50973100
H	-1.87467700	-1.08797800	2.02232200
H	-2.51560600	0.52636800	1.66932800
C	-3.08513000	0.13844900	-1.02007600
H	-2.84638900	-0.01122500	-2.07774000
H	-4.11973000	-0.18480500	-0.86489800
H	-3.01970800	1.20677400	-0.81025800
H	-0.16137500	0.74036900	1.39645900
O	-0.85809400	2.02461800	-0.07237900
H	-0.47053100	2.80968500	0.32855200

(S20)

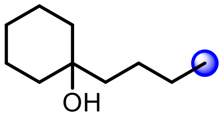

C	-1.63483500	-1.49721800	0.78279200
C	-1.01042300	-0.20299100	1.32305200
C	-0.59884400	0.77524900	0.21052600
C	-1.81422900	1.04450500	-0.70274700
C	-2.47205600	-0.23253800	-1.25100400
C	-2.84129200	-1.20163000	-0.11926300
H	-1.74049600	0.31919700	1.95112300
H	-0.88807800	-2.06959900	0.21891900
H	-1.93758100	-2.13300500	1.62076200
H	-2.54653100	1.60005200	-0.10594400
H	-1.51266000	1.70188600	-1.52826900
H	-1.79618600	-0.73235100	-1.95460500
H	-3.64040100	-0.75714200	0.48785100
H	-3.24441400	-2.13103900	-0.53463100
O	-0.24977000	1.99248600	0.90448600
H	-0.03155300	2.66405500	0.24833500
H	-3.36447100	0.03587600	-1.82568300
H	-0.15164400	-0.41781400	1.96334900
C	0.61735700	0.30004200	-0.61640500
H	0.85216900	1.10168100	-1.33143700
H	0.33342700	-0.56260900	-1.22840500
C	1.88160100	-0.03998500	0.18170700
H	2.08871300	0.76623800	0.89314400
H	1.71219200	-0.94321400	0.77925400
C	3.10734600	-0.26353900	-0.71267500
H	2.88579900	-1.04884900	-1.44627000
H	3.30060600	0.64697200	-1.29341400
C	4.36630900	-0.64195300	0.07304900
H	5.22472600	-0.78219700	-0.58993000
H	4.62838000	0.13598900	0.79695500
H	4.22055600	-1.57366600	0.62881200

(S21)

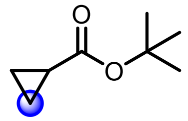

C	3.14767200	-0.12628000	0.74630600
C	1.94053000	-0.67197100	0.00004800
C	3.14764000	-0.12637500	-0.74633500
H	3.01096700	0.82474300	1.24535200
H	3.76236100	-0.84717600	1.27100400
H	1.78734900	-1.74254000	0.00011900
H	3.76230600	-0.84733700	-1.27097000
H	3.01091200	0.82458500	-1.24549500
C	0.70006100	0.15040200	0.00001400
O	0.68388600	1.36224900	-0.00003000
O	-0.39046600	-0.64139100	0.00003700
C	-1.76454000	-0.08946900	0.00000200
C	-2.62780100	-1.35125300	0.00001200
H	-2.42444000	-1.95653600	0.88615500
H	-3.68637200	-1.08003300	0.00001300
H	-2.42445000	-1.95655500	-0.88612100
C	-1.99918500	0.72880900	1.27237600
H	-1.76849600	0.13010500	2.15745300
H	-1.38809000	1.62996200	1.28550300
H	-3.05215000	1.01917000	1.32615700
C	-1.99915000	0.72875600	-1.27241400
H	-1.76829200	0.13006600	-2.15745600
H	-3.05214700	1.01898400	-1.32631500
H	-1.38817000	1.62998900	-1.28550700

(S22)

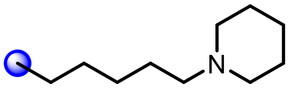

C	0.30488300	0.73402100	0.36086500
H	0.18270000	1.76173200	0.00290500
H	0.28171200	0.78504000	1.46776800
C	-0.88447600	-0.09226800	-0.13836800
H	-0.83155100	-1.11373100	0.25355600
H	-0.81749400	-0.17169400	-1.22910500
C	-2.23178300	0.52169200	0.26086300
H	-2.27505700	0.63034100	1.35235500
H	-2.30330000	1.53787300	-0.14841700
C	-3.43924200	-0.29619700	-0.21081800
H	-3.37476300	-1.31016900	0.20526800
H	-3.39418100	-0.41290300	-1.30141700
C	-4.78853800	0.32104500	0.17438900
H	-4.83229700	0.44120800	1.26381900
H	-4.85523400	1.33232600	-0.24515800
C	-5.98919100	-0.50538200	-0.29567000
H	-5.97078000	-1.51099900	0.13645800
H	-5.99203100	-0.61468400	-1.38485800
C	2.64191900	1.28557600	0.04709500
C	2.01458800	-1.00158800	0.50304500
C	3.96723700	0.84894300	-0.58143300
H	2.80554400	1.51640000	1.12011600
H	2.28969800	2.20651400	-0.42638800
C	3.31608900	-1.52995800	-0.10493500
H	2.14562700	-0.87406000	1.59793200
H	1.22049800	-1.73711400	0.36196900
C	4.43591900	-0.48936000	-0.00032200
H	4.71973900	1.62735200	-0.41763300
H	3.82855400	0.75295700	-1.66409400
H	3.60030700	-2.45783300	0.40218200
H	3.13804200	-1.77594300	-1.15769700
H	5.33779900	-0.83867100	-0.51280200
H	4.70590600	-0.35197200	1.05478600
N	1.60582700	0.26236500	-0.11611800
H	-6.93466600	-0.03766600	-0.00698200

(S23)

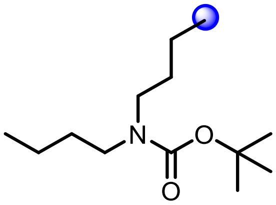

N	0.46647200	-0.15707300	-0.85852000
C	1.77121200	-0.79148400	-1.07464300
C	2.67565000	-0.84045000	0.16393600
H	2.26899500	-0.24485100	-1.88148900
H	1.59251700	-1.80957500	-1.42607400
C	3.99549700	-1.56997600	-0.11120700
H	2.88584000	0.17760900	0.51135500
H	2.13520600	-1.34789700	0.96933100
C	4.91642100	-1.62737000	1.11099400
H	3.77790900	-2.58928600	-0.45157000
H	4.52010400	-1.07684700	-0.93947800
H	5.84641500	-2.15661100	0.88542100
H	5.18105200	-0.62247400	1.45522900
H	4.43250800	-2.14559700	1.94452800
C	0.28656000	1.26759400	-1.15668900
C	0.48914800	2.21106700	0.03695400
H	-0.71427900	1.41195500	-1.56624800
H	0.99849300	1.51969600	-1.94890600
C	0.26916000	3.68028000	-0.34034600
H	-0.20615400	1.92586000	0.83305200
H	1.50009900	2.08335700	0.43937300
C	0.47079200	4.64029700	0.83574900
H	0.95248900	3.95417200	-1.15403300
H	-0.74454400	3.80241000	-0.74090600
H	0.30521400	5.67878400	0.53598600
H	-0.22208800	4.41551000	1.65252700
H	1.48699200	4.56805600	1.23584300
C	-0.53710100	-0.94452100	-0.36558800
O	-0.40887700	-2.13055500	-0.11203100
O	-1.68520700	-0.23648500	-0.20307300
C	-2.92063500	-0.88166800	0.28008300
C	-3.36997800	-1.96637200	-0.70273100
C	-2.71483900	-1.42978900	1.69501600
C	-3.91645300	0.27958800	0.29179000
H	-3.46187400	-1.55161900	-1.71014700
H	-2.66464100	-2.79539200	-0.72412900
H	-4.35070500	-2.34558200	-0.40190600
H	-2.34700900	-0.64230400	2.35825100
H	-3.67108500	-1.78499200	2.08946300
H	-2.00455900	-2.25464300	1.69790500
H	-4.89310500	-0.06837400	0.63689800
H	-3.57578800	1.07314400	0.96084100
H	-4.03322700	0.69814800	-0.71055800

(S24)

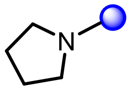

C	2.12881400	0.00000200	-0.02312100
H	2.60821500	-0.88389200	-0.45179300
H	2.60829300	0.88392100	-0.45166400
H	2.31891800	-0.00008900	1.06757800
C	-0.02210400	-1.15839200	0.15908400
H	0.17874700	-1.32089300	1.23673000
H	0.27555300	-2.06598600	-0.37289600
C	-1.49738400	0.77840300	-0.05712300
H	-1.85484600	1.16125300	-1.01491100
H	-2.13716100	1.19656200	0.72260200
C	-0.02212900	1.15831700	0.15937700
H	0.27574300	2.06615500	-0.37208100
H	0.17837100	1.32031600	1.23718300
N	0.71462900	0.00005900	-0.34275700
C	-1.49748700	-0.77834800	-0.05673100
H	-2.13674700	-1.19599600	0.72367700
H	-1.85574600	-1.16165500	-1.01404400

(S25)

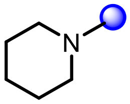

C	0.48387100	2.31509200	0.00000000
H	1.03239200	2.64772600	0.88519000
H	1.03239200	2.64772600	-0.88519000
H	-0.50235400	2.81837800	0.00000000
C	-0.27806900	0.36831600	1.20818700
H	-1.31665700	0.74999500	1.27009000
H	0.25623700	0.77934000	2.06889000
C	-0.27806900	-1.18344100	1.25968800
H	-1.30682700	-1.54620600	1.35559800
H	0.25616900	-1.53064400	2.14833700
C	-0.27806900	-1.18344100	-1.25968800
H	0.25616900	-1.53064400	-2.14833700
H	-1.30682700	-1.54620600	-1.35559800
C	-0.27806900	0.36831600	-1.20818700
H	0.25623700	0.77934000	-2.06889000
H	-1.31665700	0.74999500	-1.27009000
N	0.37853100	0.86660800	0.00000000
C	0.35429600	-1.78786600	0.00000000
H	0.22503500	-2.87462800	0.00000000
H	1.42962900	-1.59228300	0.00000000

(S26)

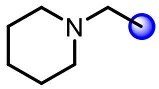

C	-1.77616800	-1.06160800	-0.37874000
C	-0.38104500	-1.31444900	0.19672900
C	0.06077900	1.05147600	0.38167500
C	-1.31841700	1.40194000	-0.18356300
C	-2.33055200	0.28586400	0.09767100
H	-0.45696900	-1.44416200	1.29613900
H	0.02605300	-2.24659700	-0.20579000
H	-1.71119400	-1.06779500	-1.47243600
H	-2.44269100	-1.87998900	-0.08733100
H	0.00848600	1.02584100	1.49013800
H	0.77346800	1.83397100	0.11464900
H	-1.65643700	2.34934100	0.24900700
H	-1.22660600	1.55270800	-1.26495700
H	-2.52615700	0.23659400	1.17676300
H	-3.28849000	0.50212600	-0.38574500
N	0.54345900	-0.22928200	-0.13938700
C	1.91046900	-0.53668100	0.28509800
C	2.97301800	0.35427200	-0.35859600
H	2.10915900	-1.57476700	0.00191000
H	2.00413300	-0.49046700	1.38797200
H	3.97076400	0.00254300	-0.08090500
H	2.88738500	0.32516600	-1.44775400
H	2.89638300	1.39556900	-0.03758400

(S27)

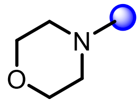

C	0.47367600	2.30918400	0.00000000
H	1.01807600	2.64828400	0.88516600
H	1.01807600	2.64828400	-0.88516600
H	-0.51984500	2.79740800	0.00000000
C	-0.25110500	0.33916100	1.19783400
H	-1.29089400	0.71303000	1.28657200
H	0.29602100	0.70578800	2.07025500
C	-0.25110500	-1.21570600	1.17415400
H	-1.28017900	-1.59537400	1.26249500
H	0.32976800	-1.61939000	2.00382300
C	-0.25110500	-1.21570600	-1.17415400
H	0.32976800	-1.61939000	-2.00382300
H	-1.28017900	-1.59537400	-1.26249500
C	-0.25110500	0.33916100	-1.19783400
H	0.29602100	0.70578800	-2.07025500
H	-1.29089400	0.71303000	-1.28657200
O	0.35052900	-1.73211600	0.00000000
N	0.39350100	0.85975400	0.00000000

(S28)

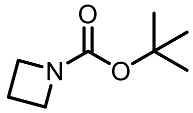

C	-3.56704200	-0.60499900	0.10859900
C	-2.16346400	-1.26273700	-0.02522400
C	-2.86840100	0.78389000	0.02849900
H	-4.23189500	-0.81252600	-0.72917600
H	-4.08979400	-0.80984400	1.04173800
H	-2.02314700	-1.94071800	-0.86998100
H	-1.79146800	-1.74460600	0.88378500
H	-3.18939300	1.44317100	-0.78086600
H	-2.85298700	1.35152200	0.96373100
N	-1.60658500	0.08255500	-0.23838300
C	-0.34948000	0.57268000	-0.07365500
O	-0.09491900	1.75777900	0.03801300
O	0.54658300	-0.44562800	-0.06998800
C	1.99710800	-0.19173900	0.01475800
C	2.45692700	0.63551600	-1.18871600
H	2.03612000	1.63956900	-1.16175600
H	3.54783400	0.71154100	-1.18233800
H	2.15643400	0.14955800	-2.12073900
C	2.58513200	-1.60246400	-0.04239800
H	3.67539400	-1.55564000	0.01492100
H	2.21989000	-2.20580300	0.79185100
H	2.30737900	-2.09818400	-0.97528600
C	2.33809500	0.48197200	1.34696600
H	1.95747600	-0.11283900	2.18166600
H	3.42441400	0.55406000	1.45071000
H	1.91328500	1.48293800	1.40324400

(S29)

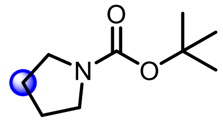

C	-0.02723500	0.61004400	-0.04434800
O	0.28004800	1.78851600	-0.10511700
O	0.84419000	-0.43136300	0.02663500
C	2.30142800	-0.21112800	0.02126700
C	2.73207200	0.45260700	-1.28999500
H	2.33499700	1.46329000	-1.36994300
H	3.82392800	0.49891300	-1.33262100
H	2.38472300	-0.13420600	-2.14466400
C	2.85172700	-1.63573400	0.10970600
H	3.94433100	-1.61555200	0.11218000
H	2.51168800	-2.12356900	1.02607500
H	2.51790400	-2.23105000	-0.74328300
C	2.71817500	0.60481200	1.24839200
H	2.35452200	0.12778000	2.16247900
H	3.80962500	0.65048700	1.30129600
H	2.32612600	1.61933800	1.19875500
C	-1.72990900	-1.24693300	0.03438100
C	-2.43780800	1.09794800	-0.06139300
C	-3.23935100	-1.16954100	-0.23758400
H	-1.52018700	-1.66170700	1.02733500
H	-1.19320900	-1.85213000	-0.69760800
C	-3.63334500	0.21174200	0.31209800
H	-2.55255300	1.53265400	-1.06148700
H	-2.26737100	1.91920100	0.63628100
H	-3.42626900	-1.21136300	-1.31514200
H	-3.78665300	-1.99134900	0.22788200
H	-4.57265900	0.58604200	-0.09880700
H	-3.73855700	0.16613300	1.40056100
N	-1.30840500	0.15956700	-0.03665300

(S30)

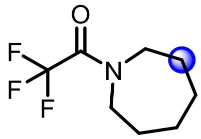

C	3.13969300	-0.05865200	0.35805400
C	2.46875100	1.31991100	0.25625200
C	1.34498000	1.43037600	-0.77747300
C	2.21324400	-1.16828300	0.88972500
C	1.29631700	-1.84726700	-0.14215200
C	0.54141800	-0.91018300	-1.11323400
H	3.56079100	-0.34881200	-0.61390100
H	2.07242700	1.60013200	1.23824800
H	1.74433400	1.28349700	-1.78633000
H	1.60148600	-0.75208100	1.69796400
H	0.57297200	-2.46267000	0.39818600
H	3.99314800	0.03619400	1.03681500
H	3.22012400	2.07497000	-0.00175200
H	0.89391200	2.42049000	-0.73085100
H	2.81905900	-1.95447500	1.35114200
H	1.88673300	-2.53466000	-0.75850300
H	-0.38487900	-1.37948300	-1.43824000
H	1.13696900	-0.75940600	-2.01663300
C	-0.86397400	0.86051700	0.02445700
O	-1.03802600	1.97199800	0.48754200
C	-2.04459400	-0.15670700	0.12714700
F	-1.67803600	-1.30778900	0.74389000
F	-2.51994300	-0.49213200	-1.09811000
F	-3.05111800	0.36527600	0.81983500
N	0.26485600	0.44197900	-0.60052400

(S31)

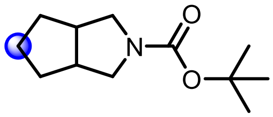

C	2.66844100	0.72025900	0.68218200
C	2.34592000	-0.81437500	0.77812500
C	0.93170700	-0.98195300	0.17858400
C	1.42834000	1.36637800	0.02765200
H	2.81243000	1.14692200	1.67644100
H	2.33455100	-1.14217200	1.81916400
H	0.98655100	-1.37588000	-0.84661300
H	0.29982200	-1.65514200	0.75582600
H	1.60891300	1.57541200	-1.03667200
H	1.12610600	2.30245500	0.49594000
C	3.95981600	0.82497800	-0.15971900
H	3.99454800	1.73669400	-0.76256000
C	3.46393100	-1.52750300	-0.01634900
H	3.11355900	-2.43975700	-0.50745900
H	4.27406700	-1.81747400	0.66081100
C	3.97541200	-0.46260200	-1.00121400
H	4.96036400	-0.69562700	-1.41316300
H	4.83413200	0.84113700	0.49933800
H	3.28542100	-0.36825900	-1.84750800
N	0.37315400	0.36876000	0.18114200
C	-0.93697500	0.69968800	0.01060100
O	-1.33720100	1.84197000	-0.13233700
O	-1.71967900	-0.41061200	0.03837700
C	-3.18737500	-0.31519900	-0.06562700
C	-3.58312400	0.27351000	-1.42278800
H	-4.66945000	0.22189000	-1.53881500
H	-3.26952000	1.31260100	-1.50850300
H	-3.12866300	-0.30268700	-2.23327900
C	-3.62068800	-1.77889500	0.03380700
H	-3.30483900	-2.20995100	0.98660600
H	-4.70869900	-1.85340000	-0.03638900
H	-3.18005000	-2.36632700	-0.77494400
C	-3.75054400	0.49494200	1.10539700
H	-3.40747400	0.07726300	2.05578300
H	-3.44493800	1.53835400	1.04681200
H	-4.84302600	0.44639400	1.08896500

(S32)

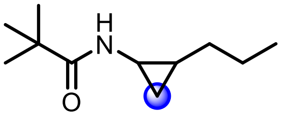

C	-0.69493600	0.39275200	-0.40779700
H	-1.89915400	2.08997000	0.37162600
H	-0.83774400	1.28467500	1.62020800
H	-1.72626700	-0.94844300	0.99479100
H	-0.81531000	0.75825700	-1.42356100
N	0.57316400	-0.23320800	-0.14632400
H	0.60021600	-1.23796300	-0.08947300
C	1.75120900	0.46090200	-0.20504800
O	1.77752800	1.66330400	-0.41840600
C	-3.23845400	-0.26934000	-0.45440000
H	-3.24739600	-1.19625800	-1.04286300
H	-3.32465400	0.55117600	-1.17746300
C	-4.45529600	-0.24992200	0.48048800
H	-4.36563500	-1.06913800	1.20423000
H	-4.44157200	0.67598800	1.06648600
C	-5.78766900	-0.36992700	-0.26440500
H	-5.84136600	-1.30284200	-0.83460200
H	-6.63387300	-0.35547600	0.42786300
H	-5.92265000	0.45611400	-0.96988900
C	3.06174100	-0.32946100	0.03658800
C	2.86606500	-1.84481700	0.22365900
H	3.83705800	-2.32103000	0.38490700
H	2.24819400	-2.07738000	1.09672000
H	2.42364800	-2.31678900	-0.65988800
C	3.71036300	0.26271000	1.30621000
H	4.68459900	-0.20299000	1.48295000
H	3.85114600	1.33877800	1.19611200
H	3.08686000	0.08762800	2.18834800
C	3.97850000	-0.07621400	-1.17786900
H	4.95498200	-0.54216600	-1.01515500
H	3.55136700	-0.49783600	-2.09300900
H	4.12073600	0.99401600	-1.33169200
C	-1.36184300	1.19935000	0.67685000
C	-1.91893800	-0.14974500	0.28153600

(S33)

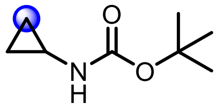

C	3.41881300	0.65773700	-0.54555200
C	3.86506700	-0.73109000	-0.15922900
C	2.66870400	-0.10628100	0.51122100
H	4.01076500	1.50546400	-0.22424200
H	2.92514600	0.78101800	-1.50057900
H	4.76788900	-0.84772800	0.42750400
H	3.67812600	-1.53109800	-0.86589500
H	2.79407100	0.23095500	1.53657300
N	1.37706200	-0.64446800	0.19639400
H	1.23028800	-1.63949500	0.27341600
C	0.25226100	0.13821400	0.17511400
O	0.24918700	1.34802100	0.25612900
O	-0.83895000	-0.65801800	0.03673700
C	-2.19716000	-0.09222000	-0.06579800
C	-2.30530100	0.79179000	-1.31121000
H	-3.34510400	1.10120300	-1.44857900
H	-1.68524300	1.68218100	-1.21821800
H	-1.99696200	0.23529300	-2.20020800
C	-3.06589500	-1.34247900	-0.21286200
H	-2.95359800	-1.99395600	0.65684800
H	-4.11765900	-1.05914700	-0.30004300
H	-2.78350900	-1.90572500	-1.10526800
C	-2.55203600	0.66562500	1.21646200
H	-3.60170800	0.97016200	1.17941800
H	-2.41728600	0.02058900	2.08877400
H	-1.93326800	1.55375300	1.33393700

(S34)

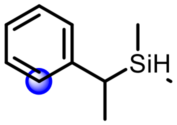

C	2.84957000	0.77285500	0.91007600
C	1.53035900	0.42040000	1.18932300
C	0.77608500	-0.34943100	0.29343600
C	2.71338000	-0.40653800	-1.18094800
C	3.44886300	0.35980600	-0.27806200
H	3.41004600	1.36607000	1.62459300
H	1.07799900	0.74327900	2.12202500
H	3.16721200	-0.73429700	-2.11001700
H	4.47573100	0.62967000	-0.49713800
Si	-1.88144600	0.33977000	-0.44034400
H	-1.69566200	-0.04318700	-1.87155200
C	-3.66663600	-0.03508000	0.05505100
H	-4.36232700	0.57865200	-0.52532800
H	-3.92582700	-1.08218200	-0.12253200
H	-3.84180000	0.17952600	1.11410000
C	-1.52113400	2.17928400	-0.23917700
H	-1.66403700	2.50165000	0.79705400
H	-0.49256900	2.41456200	-0.52369800
H	-2.18951600	2.77589300	-0.86720600
C	-0.66304100	-0.71021900	0.60601200
C	-0.95071000	-2.22289900	0.48215100
H	-0.85047200	-0.42049000	1.64806200
H	-1.95759100	-2.46265800	0.83425600
H	-0.87444900	-2.57179500	-0.55120100
H	-0.24110600	-2.80426900	1.07892400
C	1.39407500	-0.75475700	-0.89908700
H	0.83975100	-1.34773600	-1.61818200

(S35)

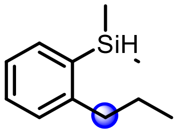

C	1.08154600	-2.84496500	-0.18417200
C	1.49204400	-1.51710700	-0.27615600
C	0.61564400	-0.44610200	-0.02754300
C	-0.72182300	-0.74682600	0.31965700
C	-1.12132300	-2.08559200	0.41278000
C	-0.23511900	-3.12988400	0.16563800
H	1.78410000	-3.64734900	-0.38097900
H	2.52259200	-1.31521100	-0.54947400
H	-2.14717900	-2.31161300	0.68898700
H	-0.57082300	-4.15793000	0.24850200
C	-1.76287300	0.32908300	0.55855400
H	-1.29331400	1.24827100	0.92070000
H	-2.44013100	0.00183700	1.35582600
C	-2.59710100	0.65811700	-0.69514100
H	-1.92429000	0.97652700	-1.49804900
H	-3.08425400	-0.25728100	-1.04861900
C	-3.64846100	1.74087500	-0.43895600
H	-3.18183400	2.67837900	-0.12061200
H	-4.23008400	1.94978000	-1.34103100
H	-4.34920800	1.43662300	0.34524000
Si	1.29743400	1.32282600	-0.12199000
H	0.31142900	2.19944300	-0.81695300
C	2.90367400	1.35895900	-1.11324200
H	3.24667500	2.39212600	-1.22409400
H	3.70497500	0.80003300	-0.62124500
H	2.76787300	0.94474900	-2.11598500
C	1.60801900	2.02293100	1.60650600
H	1.98718100	3.04788800	1.54743600
H	0.69757100	2.03734600	2.21124700
H	2.34928400	1.41987200	2.13941200

(S36)

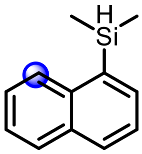

C	-0.60867600	0.43709800	-0.10706300
C	0.70831900	-0.14026800	-0.10850000
C	1.85487300	0.70280500	0.06843300
C	1.66948000	2.09949200	0.23827300
C	0.40971500	2.64117300	0.23136800
H	0.09086400	-2.19410000	-0.43754600
H	-1.69842600	2.27487400	0.05674600
C	0.93545200	-1.53307500	-0.28393000
C	3.15279900	0.12747400	0.06935300
H	2.54207100	2.73072900	0.37226700
H	0.27175900	3.70940000	0.35793000
C	3.32858400	-1.22443100	-0.09845700
C	2.20518900	-2.06165200	-0.27954500
H	4.00962300	0.77963100	0.20510000
H	4.32521100	-1.65121100	-0.09573800
H	2.34680900	-3.12763400	-0.41882100
Si	-2.19013500	-0.59769000	-0.28027500
H	-2.03264900	-1.54078500	-1.42486000
C	-3.67054400	0.51544600	-0.63877800
H	-3.51855600	1.11418500	-1.54083600
H	-4.56153400	-0.09986300	-0.79641000
H	-3.88613100	1.19610200	0.18987600
C	-2.51198800	-1.60368000	1.28627600
H	-3.41080200	-2.21805400	1.17514500
H	-1.67782600	-2.26920500	1.52224500
H	-2.66047800	-0.94262600	2.14538200
C	-0.71954600	1.80731900	0.05813200

(S37)

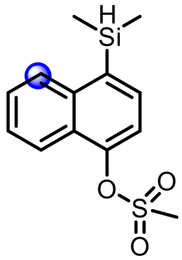

C	0.36623800	-1.41299000	-0.68147400
C	0.96063200	-0.18059700	-0.61346800
C	0.20320400	1.00836200	-0.41939700
C	-1.21717400	0.88500800	-0.25989100
C	-1.84134900	-0.40908400	-0.29971000
C	-1.03316500	-1.51110700	-0.51563400
H	1.87689000	2.36673200	-0.55483900
H	0.96649700	-2.29675800	-0.84823600
C	0.80858000	2.29145800	-0.39889300
C	-1.95959500	2.08299300	-0.07293600
H	-1.46899500	-2.50249100	-0.56950200
C	-1.34887200	3.31455300	-0.04528600
C	0.04880100	3.42105800	-0.21104100
H	-3.03593200	2.02573100	0.03331600
H	-1.94414200	4.20986700	0.09400200
H	0.52046300	4.39708900	-0.20215900
Si	-3.70512200	-0.67160000	-0.04300200
H	-4.44970200	0.28760700	-0.90801600
C	-4.18422800	-0.35606700	1.75670300
H	-3.92796300	0.65692700	2.07706800
H	-5.26114800	-0.48959200	1.89831700
H	-3.66947200	-1.05474100	2.42298800
C	-4.19239300	-2.42316000	-0.54076500
H	-5.27601900	-2.54134000	-0.44674500
H	-3.92511100	-2.63988600	-1.57842400
H	-3.72508600	-3.17884300	0.09717900
S	3.46401800	-0.73604900	0.18549400
C	3.17959500	0.11084300	1.74721800
H	3.87810300	-0.32566400	2.46050300
H	3.37939800	1.17051500	1.60441300
H	2.15185500	-0.06814600	2.05843800
O	3.13604200	-2.14277800	0.35067200
O	4.72917500	-0.31692400	-0.37857900
O	2.33680200	-0.01098000	-0.80668500

(S38)

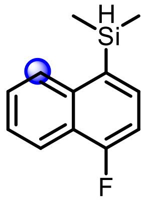

C	0.66746300	-2.35377400	0.12201200
C	1.77683300	-1.56046500	0.12975200
C	1.71854900	-0.15296300	0.00401300
C	0.42294600	0.44668200	-0.13320400
C	-0.75909800	-0.37210600	-0.13436400
C	-0.59987100	-1.74172800	-0.01206100
H	3.85082600	0.17120300	0.11615400
H	0.76941900	-3.42773500	0.21689700
C	2.88630600	0.65109300	0.00963100
C	0.37804200	1.86093800	-0.27003300
H	-1.46765100	-2.39201400	-0.01818100
C	1.52282100	2.62318200	-0.26320500
C	2.78939000	2.01544900	-0.11950500
H	-0.58051400	2.34949000	-0.39554100
H	1.45365400	3.69971600	-0.37225200
H	3.68419200	2.62721800	-0.11487300
Si	-2.51194800	0.34295600	-0.25678500
H	-2.55792900	1.32460100	-1.37852000
C	-2.99055200	1.23300100	1.33885200
H	-2.29577100	2.04196200	1.57882600
H	-3.99262700	1.66491200	1.25581800
H	-2.99297600	0.53787500	2.18373100
C	-3.75239800	-1.02985600	-0.62422700
H	-4.74848700	-0.59770600	-0.75922400
H	-3.49926700	-1.56933200	-1.54084700
H	-3.81949100	-1.75534700	0.19170100
F	3.00014500	-2.13254800	0.26059300

(S39)

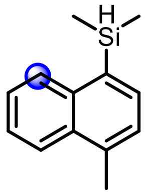

C	0.73644700	-2.29941300	0.10147900
C	1.86233200	-1.50879300	0.11937200
C	1.70310600	-0.09036000	-0.00261100
C	0.38737200	0.46968300	-0.13656000
C	-0.76860200	-0.38526600	-0.14100600
C	-0.55693700	-1.74607800	-0.02655100
H	3.81738500	0.37378400	0.10464500
H	0.84019200	-3.37649900	0.18806500
C	2.82132400	0.78623500	0.00270600
C	0.27438900	1.88043300	-0.26781500
H	-1.39867100	-2.43075600	-0.03381000
C	1.37822800	2.70041600	-0.26021800
C	2.66758500	2.14728200	-0.12025500
H	-0.70749600	2.32170400	-0.39043200
H	1.25806200	3.77294500	-0.36492900
H	3.53676100	2.79550100	-0.11351500
Si	-2.54621000	0.26445600	-0.25207600
H	-2.64462100	1.24218100	-1.37442900
C	-3.04967600	1.13878500	1.34586300
H	-2.38525600	1.97471600	1.57905900
H	-4.06872900	1.53044300	1.26956500
H	-3.01802800	0.44563300	2.19183900
C	-3.74118600	-1.15216700	-0.60725500
H	-4.75328100	-0.75572400	-0.73299000
H	-3.47741800	-1.68301400	-1.52591000
H	-3.77404700	-1.87921500	0.20934100
C	3.22870900	-2.13142000	0.26285800
H	3.86912900	-1.90422000	-0.59544600
H	3.74576000	-1.76899900	1.15711500
H	3.14864000	-3.21688300	0.34085400

(S40)

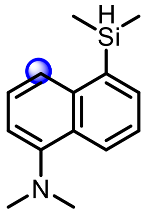

C	0.23334200	2.43439600	0.50210600
C	-0.94922300	1.73932600	0.44832000
C	-0.96773100	0.36167000	0.11110000
C	0.27949700	-0.31331900	-0.10638200
C	1.51128000	0.42775900	-0.05952600
C	1.45378900	1.78144300	0.22587800
H	0.23551600	3.48558100	0.76960700
C	-2.20187800	-0.37397900	0.02951200
C	0.25616400	-1.71639500	-0.33605500
H	2.36553900	2.36856300	0.25997200
C	-0.92805600	-2.40949800	-0.33677600
C	-2.15835500	-1.74173900	-0.16211500
H	1.18361300	-2.24648700	-0.51259200
H	-0.92821200	-3.48167300	-0.50054500
H	-3.07590300	-2.31450600	-0.20711400
Si	3.21180700	-0.37141200	-0.32356300
H	3.15669600	-1.26258400	-1.51815900
C	3.71942000	-1.40546000	1.17422400
H	2.99809900	-2.19836000	1.38724900
H	4.69482300	-1.87361700	1.00918300
H	3.79427900	-0.77795100	2.06740900
C	4.51506500	0.95534800	-0.64637500
H	5.47436100	0.47983900	-0.87224700
H	4.24833100	1.58526200	-1.49924100
H	4.66832900	1.60384800	0.22104900
H	-1.88426000	2.22640400	0.69172900
N	-3.42587600	0.33538100	0.17810900
C	-3.79859900	1.16854600	-0.96949200
H	-4.18629600	0.56323800	-1.80571700
H	-4.57616600	1.87629300	-0.66876300
H	-2.93991700	1.73264600	-1.32990200
C	-4.56387700	-0.42223900	0.67945600
H	-5.35184200	0.27941400	0.96547700
H	-4.99297000	-1.11831600	-0.06162900
H	-4.27320600	-0.99063200	1.56410000

(S41)

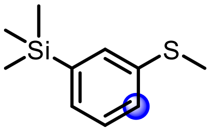

C	0.59254300	0.54350300	-0.00001200
C	-0.61516900	-0.17858300	-0.00011500
C	-1.85362100	0.46806000	-0.00008000
C	-1.89237700	1.87047200	0.00005800
C	-0.70972200	2.59634600	0.00014000
C	0.52437900	1.94229400	0.00010900
H	-0.58187000	-1.26175000	-0.00023400
H	-2.84725800	2.38516100	0.00011600
H	-0.74959500	3.68044100	0.00024300
H	1.43271700	2.53477800	0.00018000
S	-3.43249100	-0.36429100	-0.00025000
C	-2.99227100	-2.13031900	0.00036000
H	-2.43442500	-2.40459900	0.89662000
H	-3.94342300	-2.66349900	0.00057700
H	-2.43447300	-2.40527700	-0.89572100
Si	2.25028700	-0.38093100	-0.00001600
C	2.36043200	-1.47034800	-1.54120200
H	3.30673700	-2.02038500	-1.56460700
H	1.55100900	-2.20583800	-1.57427800
H	2.29835100	-0.87038800	-2.45376900
C	2.36056400	-1.46993700	1.54145000
H	3.30664800	-2.02035300	1.56476900
H	2.29895200	-0.86967900	2.45385400
H	1.55083300	-2.20507100	1.57495000
C	3.67675000	0.85602100	-0.00028500
H	4.63381100	0.32479900	-0.00035200
H	3.65928000	1.49907700	-0.88513900
H	3.65949400	1.49921700	0.88447200

(S42)

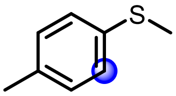

C	1.32160800	1.20447200	-0.00010700
C	-0.06347600	1.02334200	-0.00016500
C	-0.60319200	-0.26379300	-0.00006700
C	0.27303300	-1.35981700	0.00010100
C	1.64659100	-1.16323800	0.00013800
C	2.20264700	0.12400600	0.00003600
H	1.71600700	2.21587300	-0.00018400
H	-0.70455500	1.89525000	-0.00026500
H	-0.12595200	-2.36862500	0.00022900
H	2.30157700	-2.02925700	0.00027200
S	-2.34824900	-0.63443600	-0.00020100
C	-3.13283100	1.00788500	0.00033100
H	-2.87619600	1.57489800	-0.89547900
H	-4.20605500	0.81531900	0.00044200
H	-2.87591700	1.57442100	0.89636300
C	3.69915100	0.32102200	0.00002900
H	4.16109800	-0.13627500	0.88079200
H	4.16097300	-0.13535100	-0.88128600
H	3.95982900	1.38145500	0.00055300

(S43)

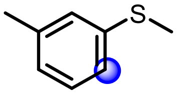

C	-1.86491300	-0.43855400	0.03699900
C	-0.53785800	-0.82702600	-0.18189100
C	0.48589300	0.11796600	-0.28076800
C	0.18520800	1.47909600	-0.16964600
C	-1.13001500	1.87583900	0.05525500
C	-2.14539500	0.92569500	0.15549800
H	-0.29340200	-1.87910200	-0.28180300
H	0.97290400	2.21600000	-0.27315000
H	-1.36756600	2.93076300	0.13849300
H	-3.16827400	1.24885900	0.32012100
C	-2.95679600	-1.47372600	0.16753300
H	-2.77423900	-2.33000300	-0.48608200
H	-3.01730300	-1.85276000	1.19358600
H	-3.93351600	-1.05548700	-0.08559100
S	2.17059700	-0.42243300	-0.61087600
C	2.90434800	-0.28181800	1.06512200
H	3.94842300	-0.58343600	0.96881700
H	2.86085500	0.74627300	1.42491400
H	2.39973500	-0.94700100	1.76610700

(S44)

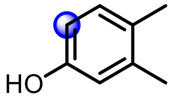

C	-1.07863800	-0.60692800	-0.00005000
C	-0.68654700	0.74896300	0.00013200
C	0.66927700	1.07436000	0.00002200
C	1.64787500	0.08079700	-0.00003800
C	1.27238400	-1.25892900	0.00015400
C	-0.08357500	-1.58447400	0.00000100
H	2.02409100	-2.04285900	0.00009600
H	-0.37023800	-2.63126100	0.00009100
O	2.95837400	0.48835400	-0.00004400
H	3.53560400	-0.28203700	-0.00049400
H	0.98635400	2.11122700	0.00009300
C	-2.53665100	-0.99896200	-0.00007500
H	-3.06246300	-0.61242300	0.87969600
H	-3.06277300	-0.61099900	-0.87900500
H	-2.64772200	-2.08500900	-0.00092200
C	-1.71862100	1.85042400	-0.00001100
H	-2.36907800	1.79057900	-0.87891600
H	-2.36873200	1.79093100	0.87918900
H	-1.24506700	2.83350300	-0.00029400

(S45)

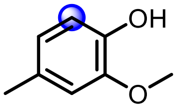

C	-1.73179300	-0.35974100	-0.00032400
C	-0.45491000	-0.94612500	-0.00015100
C	0.68674700	-0.15542700	0.00009700
C	0.57952800	1.24747800	0.00014000
C	-0.67638700	1.83086600	-0.00013500
C	-1.82214600	1.02996800	-0.00039700
H	-0.36807600	-2.02608000	-0.00026400
H	-0.74782000	2.91205000	-0.00027100
H	-2.79747000	1.50421900	-0.00070700
O	1.70170800	2.02464600	0.00025900
H	2.46746300	1.43504500	0.00032600
O	1.98324700	-0.61306000	0.00014400
C	2.21118700	-2.01634700	-0.00021600
H	1.78804900	-2.48457900	-0.89493400
H	3.29223000	-2.14547300	-0.00022200
H	1.78800300	-2.48505800	0.89422900
C	-2.96459800	-1.23273300	0.00041000
H	-2.99259800	-1.88834000	-0.87603000
H	-3.00160700	-1.87403100	0.88712900
H	-3.87360000	-0.62808100	-0.00902200

(S46)

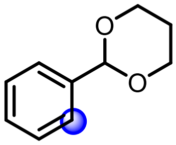

C	-3.60635200	0.28207400	0.00030600
C	-3.13104700	-1.02678400	-0.00091400
C	-1.75700400	-1.26792000	-0.00118000
C	-0.85412100	-0.20589400	-0.00023100
C	-1.33490200	1.10617200	0.00098300
C	-2.70501400	1.34813800	0.00125200
H	-4.67383400	0.47275400	0.00053900
H	-3.82599800	-1.85903400	-0.00165200
H	-1.38750700	-2.28836200	-0.00215000
H	-0.63124800	1.92939300	0.00169800
H	-3.07263900	2.36830500	0.00219800
C	0.62837400	-0.46734300	-0.00054400
H	0.83143000	-1.55288800	-0.00176900
C	2.60584100	-0.13402100	-1.24246000
C	2.60584600	-0.13686600	1.24217300
C	3.30379000	0.41595300	0.00048200
H	2.94632800	0.35432400	-2.15619500
H	2.79240700	-1.21493400	-1.34097700
H	2.94639300	0.34933500	2.15703200
H	2.79240900	-1.21800200	1.33816600
H	4.36212100	0.13423500	0.00014900
H	3.24255000	1.50796100	0.00174200
O	1.19657000	0.09732600	-1.16922600
O	1.19657100	0.09465600	1.16947600

(S47)

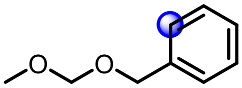

C	-0.88827800	-3.66746700	0.00000000
C	-0.88750400	-2.96820300	1.20608200
C	-0.88750400	-1.57510500	1.20360800
C	-0.88714400	-0.86518000	0.00000000
C	-0.88750400	-1.57510500	-1.20360800
C	-0.88750400	-2.96820300	-1.20608200
H	-0.89088500	-4.75176100	0.00000000
H	-0.88787200	-3.50718300	2.14691600
H	-0.88334400	-1.03267300	2.14344000
H	-0.88334400	-1.03267300	-2.14344000
H	-0.88787200	-3.50718300	-2.14691600
C	-0.85847100	0.63987400	0.00000000
H	-1.37519500	1.03103500	0.88842700
H	-1.37519500	1.03103500	-0.88842700
O	0.49977800	1.07887100	0.00000000
C	0.61219500	2.47193200	0.00000000
H	0.12752900	2.90178800	0.89888300
H	0.12752900	2.90178800	-0.89888300
O	1.97984100	2.75188400	0.00000000
C	2.25070100	4.14123700	0.00000000
H	1.84164900	4.63339900	0.89345500
H	3.33447700	4.25030900	0.00000000
H	1.84164900	4.63339900	-0.89345500

(S48)

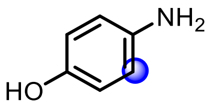

C	-0.72320500	1.19010800	0.00033300
C	-1.41775000	-0.01741900	-0.00251900
C	-0.70309900	-1.21419300	0.00019600
C	0.68659000	-1.19946900	0.00625800
C	1.39939600	0.00790200	0.00675000
C	0.67067600	1.20181200	0.00578000
H	-1.24605900	-2.15178000	-0.00186900
H	1.22764900	-2.14041100	0.01551000
H	1.19408400	2.15254000	0.01459100
N	2.80396700	0.01598200	0.07580600
H	3.24869700	-0.81360900	-0.29076100
H	3.23988900	0.85156300	-0.28767800
H	-1.26556700	2.13137600	-0.00207400
O	-2.79229300	-0.09351300	-0.00940700
H	-3.16377200	0.79410600	-0.00389500

(S49)

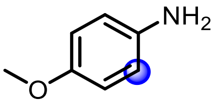

C	-0.41338300	1.00875500	0.00149300
C	-0.94563600	-0.28022400	-0.00182200
C	-0.07339300	-1.37351400	0.00036400
C	1.29966300	-1.18129400	0.00653600
C	1.84954100	0.11049200	0.00676700
C	0.97129900	1.19552000	0.00601000
H	-0.49236600	-2.37279700	-0.00182400
H	1.95828600	-2.04417000	0.01637900
H	1.36631500	2.20657000	0.01415700
N	3.24158600	0.29842700	0.07484000
H	3.78833300	-0.46256900	-0.30249900
H	3.56505800	1.18749100	-0.27910100
H	-1.05587500	1.87919900	-0.00019700
O	-2.28552100	-0.57791700	-0.00833900
C	-3.21042000	0.49634500	-0.00270200
H	-4.19967300	0.04067600	-0.00492800
H	-3.10126900	1.11708500	0.89437700
H	-3.10177300	1.12637800	-0.89341400

(S50)

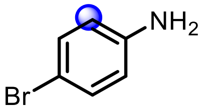

C	0.36880800	1.20728700	-0.00386500
C	-0.32230500	0.00002000	-0.00272500
C	0.36881400	-1.20725900	-0.00394700
C	1.75977400	-1.20455000	-0.00605700
C	2.47853700	-0.00000300	-0.00531300
C	1.75978100	1.20453300	-0.00608900
H	-0.16995400	2.14630900	-0.00275000
H	-0.16998000	-2.14626400	-0.00266800
H	2.29334100	-2.14950200	-0.01282800
H	2.29340500	2.14945700	-0.01269100
N	3.87267700	-0.00003200	-0.06405600
H	4.32862500	0.83851400	0.26332900
H	4.32856400	-0.83835700	0.26396900
Br	-2.24266300	-0.00000300	0.00342900

(S51)

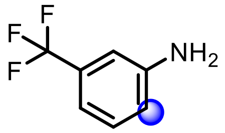

C	-0.28374900	0.19687600	-0.03498800
C	0.07807900	1.54349900	-0.02547700
C	1.43314100	1.86749900	-0.00157000
C	2.40506400	0.87513000	0.01515400
C	2.04138000	-0.48095700	0.00494800
C	0.67905300	-0.80902800	-0.01758700
H	1.73591600	2.90829100	0.00147100
H	3.45567600	1.14602600	0.03706700
H	0.37465000	-1.84924800	-0.02647600
N	3.00953000	-1.47915800	0.07139500
H	2.73131700	-2.39592500	-0.24462300
H	3.93639700	-1.22458000	-0.23467600
H	-0.67805500	2.31643400	-0.04542800
C	-1.73874300	-0.18569700	-0.00429000
F	-2.18609900	-0.37069500	1.26543700
F	-1.98251400	-1.34283800	-0.66504200
F	-2.53227100	0.75899800	-0.55642100

(S53)

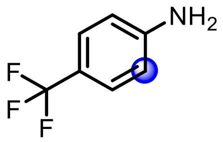

C	0.32298200	1.20483100	-0.02935200
C	-0.38324100	-0.00002500	-0.04091200
C	0.32299900	-1.20485700	-0.02936200
C	1.70961100	-1.20751700	-0.01268300
C	2.42781100	-0.00000600	-0.00128700
C	1.70957800	1.20750200	-0.01268100
H	-0.21360100	-2.14558500	-0.04106000
H	2.24605500	-2.15047800	-0.01481300
H	2.24603500	2.15045600	-0.01479100
N	3.81447500	0.00002600	-0.03679800
H	4.27996000	0.84319600	0.26160000
H	4.28001700	-0.84311000	0.26160900
H	-0.21364900	2.14554100	-0.04105800
C	-1.87911300	-0.00001400	0.00004100
F	-2.41257000	1.08926700	-0.60364900
F	-2.41258700	-1.08965700	-0.60292200
F	-2.36483300	0.00042400	1.27362900

(S52)

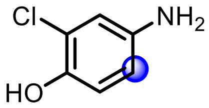

C	0.59219000	-0.34852800	0.00212100
C	0.66535300	1.04536600	-0.00170800
C	-0.53648000	1.75394800	-0.00047200
C	-1.75742500	1.09405800	0.00473400
C	-1.82221300	-0.30712500	0.00615000
C	-0.62217800	-1.02515900	0.00673600
H	-0.49249900	2.83645100	-0.00356300
H	-2.67453000	1.67345600	0.01237300
H	-0.62662800	-2.10851700	0.01698600
N	-3.05496500	-0.97266900	0.07394100
H	-3.84158600	-0.44774300	-0.28012800
H	-3.05325100	-1.91808300	-0.28073400
O	1.83734900	1.74383900	-0.00801300
H	2.57616600	1.12169100	-0.00494300
Cl	2.09898900	-1.26909700	-0.00110800

(S54)

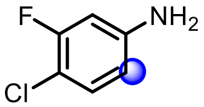

C	-0.24800600	0.90811100	-0.00241100
C	-0.86305600	-0.33983400	-0.00076100
C	-0.05217700	-1.47181000	-0.00218300
C	1.33171200	-1.35569700	-0.00484100
C	1.94461000	-0.09246300	-0.00512200
C	1.12912800	1.04722700	-0.00536900
H	-0.51518000	-2.45057500	-0.00049100
H	1.94250000	-2.25154800	-0.01179800
H	1.55000800	2.04575300	-0.01309000
N	3.33028600	0.02966800	-0.06431200
H	3.71781000	0.90418600	0.25614700
H	3.86046300	-0.76925700	0.24914000
Cl	-2.60606200	-0.47542500	0.00552700
F	-1.00197900	2.02475500	0.00005000

(S55)

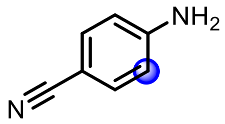

C	0.38858500	1.20858000	0.00205200
C	1.10305900	-0.00005600	0.00068500
C	0.38838400	-1.20868600	0.00210900
C	-0.99580400	-1.20901300	0.00438600
C	-1.71432200	0.00023500	0.00431200
C	-0.99573500	1.20921500	0.00440900
H	0.92817500	-2.14783500	0.00035700
H	-1.53320500	-2.15136800	0.00981000
H	-1.53263300	2.15185200	0.00938700
N	-3.09670700	-0.00012200	0.05507100
H	-3.57321800	0.84501800	-0.21818600
H	-3.57244300	-0.84548900	-0.21882500
H	0.92839600	2.14770100	0.00061100
C	2.52987700	-0.00003600	-0.00361900
N	3.68680100	-0.00006600	-0.00780700

(S56)

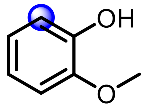

C	0.47759800	0.98691200	0.00004100
C	-0.43937600	-0.08039300	0.00035200
C	0.01664400	-1.39360500	0.00035900
C	1.39252100	-1.64836300	0.00001400
C	2.29812000	-0.59373400	-0.00020600
C	1.84044300	0.72562100	-0.00020900
H	3.36384700	-0.78967800	-0.00042500
H	2.52834000	1.56271400	-0.00042700
O	0.02971800	2.27462500	-0.00004900
H	-0.93665600	2.25057200	0.00041400
H	-0.68294600	-2.21926300	0.00068400
O	-1.75503000	0.31771200	0.00048800
C	-2.76785700	-0.68077600	-0.00057300
H	-3.71658400	-0.14680200	-0.00118500
H	-2.70211600	-1.30827000	-0.89529400
H	-2.70351600	-1.30860100	0.89405300
H	1.74357100	-2.67333800	0.00000700

(S57)

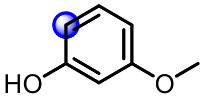

C	-1.64783800	-0.34708900	-0.00002100
C	-0.40778700	-0.97459400	-0.00009100
C	0.76235000	-0.20560800	-0.00007900
C	0.68808900	1.18887800	-0.00002700
C	-0.57210300	1.79494900	-0.00001700
C	-1.74100300	1.04863900	0.00001100
H	-0.32008600	-2.05602800	-0.00003300
H	-0.63391000	2.87749700	-0.00001500
H	-2.71704200	1.51647700	0.00002800
O	-2.82123400	-1.05272500	0.00008700
H	-2.63306700	-1.99701100	0.00006700
H	1.57889000	1.80091100	0.00008000
O	1.92590100	-0.91948900	-0.00006100
C	3.15726000	-0.20935300	0.00010500
H	3.93765700	-0.96859800	0.00013600
H	3.25832200	0.41476300	-0.89414000
H	3.25809100	0.41477100	0.89438500

(S58)

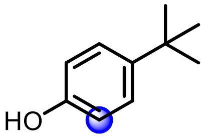

C	-2.48176600	-0.01299500	-0.00003300
C	-1.76132200	-1.20789700	0.00003100
C	-0.37335500	-1.17608600	0.00015400
C	0.34938200	0.02885000	0.00003400
C	-0.39823400	1.20906900	0.00001700
C	-1.79487100	1.19681600	0.00000000
H	-2.29984100	-2.14817200	0.00005000
H	0.15571300	-2.12241300	0.00019800
H	0.09794400	2.17069600	0.00003600
H	-2.34239600	2.13535700	0.00006900
C	1.88827200	0.00789300	-0.00004600
O	-3.85048900	-0.09609600	-0.00005800
H	-4.22817800	0.78944100	-0.00017100
C	2.49120300	1.42484900	-0.00010700
H	2.19586700	1.99348400	0.88611200
H	3.58250400	1.35768200	0.00037700
H	2.19670900	1.99311000	-0.88683000
C	2.39630500	-0.72865100	1.26142700
H	2.03828400	-1.76005900	1.30101700
H	3.49057200	-0.75529800	1.27324900
H	2.05829900	-0.22317400	2.17027700
C	2.39629300	-0.72890800	-1.26140000
H	2.03864000	-1.76046300	-1.30047000
H	2.05779800	-0.22393100	-2.17035000
H	3.49056400	-0.75512400	-1.27356600

(S59)

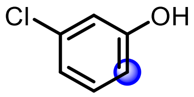

C	0.77441100	1.81763500	-0.00002000
C	1.80756100	0.88332500	0.00006300
C	1.50212900	-0.47883900	-0.00006200
C	0.17292000	-0.90583600	-0.00005700
C	-0.83384200	0.04965900	-0.00012500
C	-0.55835200	1.41503500	-0.00004500
H	1.01183500	2.87518300	0.00007600
H	2.84255000	1.21062200	0.00019800
H	-1.36355800	2.13710200	0.00009800
Cl	-2.50922700	-0.48703200	0.00004800
H	-0.05199000	-1.96387100	-0.00004100
O	2.46159600	-1.45199800	0.00005300
H	3.33628400	-1.04936900	-0.00008100

(S60)

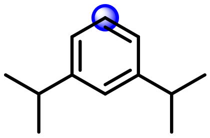

C	1.20949600	1.39973000	-0.00008000
C	1.22627800	0.00085900	-0.00004100
C	0.00000000	-0.67221600	0.00000000
C	-1.22627800	0.00085900	0.00004100
C	-1.20949600	1.39973000	0.00008000
C	0.00000000	2.09059700	0.00000000
H	2.14058000	1.95661300	-0.00018300
H	-2.14058000	1.95661300	0.00018300
H	0.00000000	3.17550700	0.00000000
C	-2.53379400	-0.77954600	-0.00006800
H	-2.26824100	-1.84301000	-0.00034300
C	2.53379400	-0.77954600	0.00006800
H	2.26824100	-1.84301000	0.00034300
H	0.00000000	-1.75922500	0.00000000
C	-3.36249900	-0.51208500	1.26944000
H	-3.67615600	0.53455800	1.32509800
H	-4.26511900	-1.13074800	1.27876200
H	-2.78691700	-0.73551200	2.17136500
C	-3.36263500	-0.51153900	-1.26938600
H	-2.78710700	-0.73452300	-2.17145000
H	-4.26519800	-1.13028100	-1.27887600
H	-3.67639600	0.53510500	-1.32454100
C	3.36263500	-0.51153900	1.26938600
H	2.78710700	-0.73452300	2.17145000
H	4.26519800	-1.13028100	1.27887600
H	3.67639600	0.53510500	1.32454100
C	3.36249900	-0.51208500	-1.26944000
H	4.26511900	-1.13074800	-1.27876200
H	2.78691700	-0.73551200	-2.17136500
H	3.67615600	0.53455800	-1.32509800

(S61)

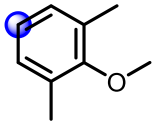

C	1.72568700	1.20280600	0.13655500
C	0.34728000	1.22708400	-0.09485400
C	-0.32682900	0.00047500	-0.18984300
C	0.34446300	-1.22766600	-0.09500300
C	1.72294700	-1.20653100	0.13639600
C	2.41052300	-0.00266400	0.25944600
H	2.26487900	2.14173000	0.20953200
H	2.25999100	-2.14669900	0.20922800
H	3.48044900	-0.00389100	0.43499600
C	-0.39917900	-2.53009500	-0.26111500
H	-0.99215200	-2.77626700	0.62613000
H	-1.09021800	-2.48274700	-1.10540100
H	0.29898500	-3.35285100	-0.42650200
C	-0.39350500	2.53113100	-0.26097100
H	-1.08223100	2.48637200	-1.10730300
H	-0.98865000	2.77693000	0.62490400
H	0.30660600	3.35287900	-0.42307600
O	-1.68531400	0.00218500	-0.46937700
C	-2.53960300	0.00219000	0.67459100
H	-3.56263900	0.00409600	0.29796400
H	-2.38291700	-0.88975400	1.29071000
H	-2.38028600	0.89234600	1.29262600

(S62)

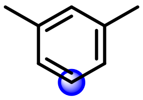

C	0.00000000	1.22354700	-0.27008800
C	0.00399200	1.20841100	1.12828800
C	0.00000000	0.00000000	1.82105300
C	-0.00399200	-1.20841100	1.12828800
C	0.00000000	-1.22354700	-0.27008800
C	0.00000000	0.00000000	-0.94736700
H	0.00813300	2.14536100	1.67610700
H	0.00000000	0.00000000	2.90576500
H	-0.00813300	-2.14536100	1.67610700
H	0.00000000	0.00000000	-2.03418000
C	0.03596800	-2.52744000	-1.03148100
H	-0.45281200	-2.43593700	-2.00440600
H	1.06803900	-2.84771200	-1.21256300
H	-0.46090500	-3.32661000	-0.47630200
C	-0.03596800	2.52744000	-1.03148100
H	0.45281200	2.43593700	-2.00440600
H	-1.06803900	2.84771200	-1.21256300
H	0.46090500	3.32661000	-0.47630200

(S63)

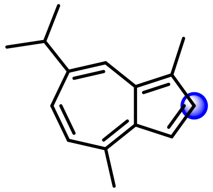

C	0.71096500	0.57604100	-0.00001500
C	-0.65998100	0.76134700	-0.00007100
C	-1.74697600	-0.27170500	-0.00003400
C	1.47384400	-0.60527100	-0.00003700
C	-1.59706400	-1.66875600	-0.00005200
C	0.94132600	-1.89296600	-0.00002900
C	-0.38079700	-2.35616700	0.00001200
H	1.28631400	1.49885300	0.00003600
H	1.68089100	-2.69077300	0.00004300
H	-0.46790000	-3.43883500	0.00001800
C	-1.29422400	2.03464000	0.00002900
C	-2.67218400	1.81482400	-0.00004700
H	-3.42007300	2.59891700	-0.00016900
C	-2.95453500	0.43228100	-0.00000300
H	-3.94348100	-0.00058400	-0.00007800
C	2.99940100	-0.46868100	-0.00002400
H	3.40500500	-1.48590000	-0.00006500
C	3.52380800	0.22613800	1.27012300
H	4.61794800	0.23904000	1.27586600
H	3.18223900	1.26368700	1.33204800
H	3.18466600	-0.29070100	2.17152700
C	3.52383900	0.22625100	-1.27009100
H	4.61797900	0.23909600	-1.27583300
H	3.18467200	-0.29046700	-2.17155500
H	3.18232800	1.26382600	-1.33189900
C	-0.61418400	3.37298000	0.00009300
H	0.02035300	3.51489100	0.88210800
H	0.02046700	3.51488400	-0.88183900
H	-1.35425000	4.17634400	0.00004200
C	-2.85793800	-2.50723300	0.00006000
H	-3.47078700	-2.29081900	0.88014000
H	-3.47091100	-2.29085500	-0.87994300
H	-2.62726800	-3.57295000	0.00006800

(S64)

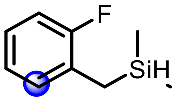

C	2.65957900	-1.45548300	0.40178500
C	1.35803400	-1.52774100	-0.08837300
C	0.66085500	-0.38410100	-0.50360300
C	1.34833500	0.82429900	-0.39379100
C	2.64335400	0.93256700	0.08618100
C	3.30635000	-0.22368700	0.49103300
H	3.16887700	-2.36089700	0.71077800
H	0.86343500	-2.49102100	-0.15777500
H	4.31979500	-0.15977800	0.86910000
C	-0.75220600	-0.44529800	-1.01630900
H	-0.89958300	-1.37297100	-1.57887500
H	-0.94335800	0.38042100	-1.70732200
Si	-2.08948700	-0.39100600	0.34864200
H	-1.91517100	-1.60914200	1.19320500
C	-3.79229700	-0.44535600	-0.46386000
H	-3.90940000	-1.33628200	-1.08770100
H	-4.58480000	-0.46293900	0.29016400
H	-3.95688100	0.43129100	-1.09792600
C	-1.90564000	1.13843100	1.43193600
H	-0.92357900	1.17089300	1.91097700
H	-2.02300800	2.05621600	0.84875500
H	-2.66204700	1.14227400	2.22250900
F	0.71654800	1.96622900	-0.78899200
H	3.11142100	1.90816900	0.13406400

(S65)

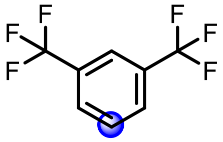

C	-1.20190100	0.30961700	-0.02986100
C	-1.20623600	1.70457800	-0.02843000
C	-0.00000100	2.39813200	0.00000000
C	1.20623700	1.70455800	0.02842100
C	1.20188800	0.30960800	0.02983500
C	-0.00001700	-0.39435300	-0.00001100
H	-2.14580100	2.24182400	-0.05492300
H	0.00001400	3.48127100	0.00000700
H	2.14579500	2.24181800	0.05491100
H	-0.00001000	-1.47635700	-0.00004300
C	2.50776800	-0.44203100	0.00427900
C	-2.50777300	-0.44203600	-0.00425900
F	3.49360400	0.23532000	0.63259200
F	2.41163500	-1.65226900	0.59452000
F	2.93304900	-0.65953700	-1.26516200
F	-2.41166000	-1.65220900	-0.59467700
F	-3.49368300	0.23539900	-0.63240700
F	-2.93292200	-0.65970400	1.26515700

(S66)

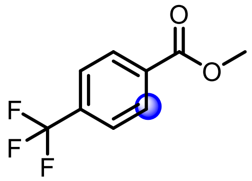

C	-1.47955500	0.00024800	-0.03719600
C	-0.70245800	-1.15797400	-0.03112900
C	0.68471200	-1.05934200	-0.01888500
C	1.29749600	0.19740100	-0.00900400
C	0.51144900	1.35413800	-0.01381800
C	-0.87384900	1.25846800	-0.02686100
H	-1.17894000	-2.12973100	-0.04387100
H	1.29398900	-1.95295400	-0.01860700
H	1.00164600	2.31941300	-0.00922600
H	-1.48185500	2.15435900	-0.03655100
C	2.78293000	0.36152000	0.00266100
O	3.34871500	1.42993800	0.01081700
O	3.43001900	-0.82078100	0.00328700
C	-2.98192200	-0.10208600	0.00201600
F	-3.57890300	0.89543900	-0.68774500
F	-3.45381600	-0.03059000	1.27244200
F	-3.43013400	-1.26796100	-0.51281300
C	4.86892600	-0.75135000	0.01483400
H	5.20790100	-1.78470000	0.01175400
H	5.23026600	-0.22393600	-0.86869800
H	5.21643300	-0.23383400	0.90970300

(S67)

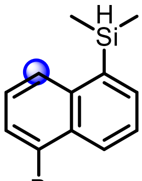

C	-0.40390100	-2.44561100	0.12763600
C	0.75526300	-1.71349400	0.10894600
C	0.71618900	-0.30303400	-0.03708800
C	-0.56151400	0.34899400	-0.16213900
C	-1.76777300	-0.43315200	-0.13034300
C	-1.65656000	-1.80505100	0.00807700
H	-0.36104100	-3.52377100	0.23519500
C	1.88301300	0.51311700	-0.06923700
C	-0.59024900	1.76036900	-0.31917500
H	-2.54821900	-2.42242900	0.02612600
C	0.56485000	2.50201100	-0.34574500
C	1.82203700	1.87596900	-0.21650800
H	-1.54271500	2.26209100	-0.43466100
H	0.52134800	3.57797200	-0.46970300
H	2.72956900	2.46491300	-0.23719900
Si	-3.50352400	0.33505500	-0.22660800
H	-3.54017100	1.31498700	-1.34981600
C	-3.92721400	1.23790600	1.37687300
H	-3.20735300	2.02820100	1.60437300
H	-4.91901400	1.69583200	1.31209000
H	-3.93266200	0.54251400	2.22147800
C	-4.78834700	-1.00205400	-0.57079700
H	-5.77261900	-0.54038700	-0.69449300
H	-4.56376000	-1.55270900	-1.48818700
H	-4.86620800	-1.72187000	0.24908200
H	1.71391300	-2.20556000	0.20372000
Br	3.63038600	-0.28075300	0.09632900

(S68)

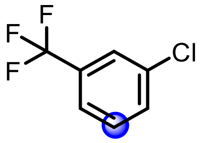

C	1.74234300	-0.00948100	-0.00233900
C	1.91242400	1.37176900	0.01104500
C	0.78711600	2.19278700	0.00263900
C	-0.49120800	1.64247100	-0.01808700
C	-0.63925200	0.25571700	-0.03285400
C	0.47557800	-0.58197800	-0.02302900
H	2.90889000	1.79437200	0.02455100
H	0.91164600	3.26903100	0.00952300
H	-1.36406700	2.28232800	-0.03108000
H	0.36060200	-1.65719300	-0.03954800
C	-2.01844100	-0.35016700	-0.00363900
F	-2.05046000	-1.57666300	-0.56845600
F	-2.92307900	0.41464500	-0.65389400
F	-2.47464000	-0.49485800	1.26615100
Cl	3.15324600	-1.05313800	0.00234900

(S69)

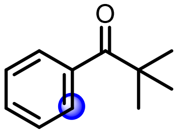

C	-0.71386000	0.25441900	-0.08007900
C	-1.67868700	1.25379600	0.12590700
C	-3.02974100	0.93950200	0.20632200
H	-4.50334300	-0.63094100	0.11811600
H	-2.82422900	-2.41346100	-0.27943000
H	-0.44563300	-1.87049500	-0.39399700
H	-1.34306400	2.27895800	0.21842900
H	-3.75693600	1.72538500	0.37689900
C	0.72393800	0.71770200	-0.16960600
O	0.92934600	1.88926500	-0.42797100
C	1.92904800	-0.22847600	0.07915700
C	2.14462200	-1.12895400	-1.16218300
H	3.04744800	-1.73005100	-1.01871700
H	1.31803200	-1.81494200	-1.35070900
H	2.28698500	-0.52121300	-2.05970800
C	1.73308100	-1.07691200	1.35462400
H	0.90041100	-1.77619500	1.28649900
H	2.64099800	-1.65627000	1.54642000
H	1.55950000	-0.43745400	2.22500700
C	3.18995600	0.63734500	0.26205100
H	3.09233100	1.30418000	1.12170900
H	4.05530900	-0.01125600	0.42594300
H	3.37884900	1.25760100	-0.61441400
C	-3.44921300	-0.38315000	0.06233300
C	-2.50620500	-1.38429000	-0.15655900
C	-1.14984300	-1.07064500	-0.21834800

(S70)

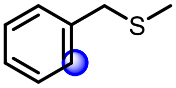

S	-2.08028200	-0.00025000	-0.68396600
C	-3.71950100	0.00004700	0.12042400
H	-4.46254500	-0.00028300	-0.67790600
H	-3.86004900	-0.89347100	0.73198800
H	-3.86007800	0.89405000	0.73127300
C	-1.00771200	0.00043500	0.82194700
H	-1.24986000	-0.88632600	1.41249300
H	-1.24980500	0.88776000	1.41166200
C	0.44610900	0.00021200	0.43180700
C	1.13346900	1.20351000	0.23950200
C	1.13319000	-1.20331200	0.23986700
C	2.47709600	1.20497600	-0.12809600
H	0.61075800	2.14424900	0.37861000
C	2.47681100	-1.20520300	-0.12772900
H	0.61025400	-2.14388400	0.37925600
C	3.15304000	-0.00021800	-0.31257300
H	2.99605600	2.14655200	-0.26919200
H	2.99555400	-2.14694000	-0.26854400
H	4.19921500	-0.00038400	-0.59709300

(S71)

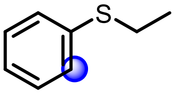

C	-2.10873600	1.43567800	-0.00028700
C	-0.75846700	1.08250400	-0.00022700
C	-0.38644200	-0.26598400	0.00003100
C	-1.38820700	-1.24890500	0.00022300
C	-2.72970400	-0.88660100	0.00015800
C	-3.10012300	0.45939600	-0.00009800
H	-2.37993300	2.48585300	-0.00048700
H	-0.01111400	1.86436900	-0.00038200
H	-1.11169200	-2.29775500	0.00042700
H	-3.48954900	-1.66030000	0.00030900
H	-4.14679900	0.74007700	-0.00014700
S	1.29767400	-0.85191600	0.00012500
C	2.30823400	0.67932000	-0.00008300
H	2.06347000	1.26639000	0.88768900
H	2.06353600	1.26611100	-0.88805600
C	3.78812900	0.29971700	0.00002900
H	4.04996400	-0.28495700	-0.88497800
H	4.40132800	1.20479500	-0.00009800
H	4.04990400	-0.28467100	0.88524300

(S72)

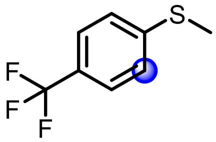

C	-0.34266000	-1.10803500	-0.03088900
C	1.04422700	-0.98091500	-0.01788900
C	1.63630400	0.28635900	-0.00575300
C	0.81122900	1.42341400	-0.00997500
C	-0.56802000	1.29283000	-0.02395200
C	-1.15400200	0.02302800	-0.03660700
H	-0.78885300	-2.09448500	-0.04407800
H	1.64952100	-1.87702700	-0.01908400
H	1.25599200	2.41210900	-0.00457000
H	-1.19150600	2.17852500	-0.03264400
S	3.38813100	0.57941900	0.01051800
C	4.10747800	-1.09321400	0.01210200
H	3.83885800	-1.64511500	-0.88923000
H	5.18679700	-0.93986600	0.02306600
H	3.82143900	-1.65101100	0.90440400
C	-2.64810600	-0.10915400	0.00054600
F	-3.26503900	0.84234800	-0.74015300
F	-3.14069600	0.01499700	1.26223500
F	-3.07215900	-1.30842500	-0.45893200

(S73)

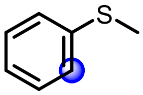

C	1.70161200	1.37544000	0.00001100
C	0.33414000	1.09588800	0.00000700
C	-0.10990300	-0.23003600	-0.00001900
C	0.83591900	-1.26660400	-0.00000900
C	2.19504700	-0.97743700	0.00000300
C	2.63810900	0.34650600	-0.00000800
H	2.02951500	2.40926700	0.00003500
H	-0.37025700	1.91705300	0.00001000
H	0.50252600	-2.29880900	0.00000600
H	2.91186700	-1.79113600	-0.00002200
H	3.69853800	0.56951700	-0.00000400
S	-1.82425400	-0.71999800	0.00001500
C	-2.72498800	0.86156800	-0.00001900
H	-2.51118700	1.44570900	-0.89603900
H	-3.78123000	0.59073200	-0.00008100
H	-2.51132600	1.44567800	0.89605800

(S74)

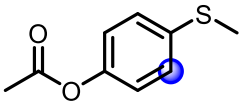

C	0.26887000	1.41619600	0.37069300
C	-1.11998400	1.31721500	0.34010200
C	-1.73831400	0.23764400	-0.29688600
C	-0.94592500	-0.73294000	-0.91888500
C	0.44274100	-0.64116100	-0.89436700
C	1.03714000	0.43419700	-0.24256700
H	0.76032600	2.24998200	0.85726700
H	-1.72756600	2.08238200	0.80798900
H	-1.42034800	-1.56166100	-1.43062700
H	1.05279800	-1.39574700	-1.37268900
S	-3.53143800	0.12709900	-0.36167400
C	-3.86000100	-0.89082800	1.13012200
H	-3.52155800	-0.37994500	2.03165400
H	-4.94182000	-1.02451200	1.17661800
H	-3.38055100	-1.86634000	1.04825700
O	2.42085600	0.62536400	-0.25201600
C	3.25800400	-0.34954100	0.23272900
O	2.87709900	-1.39075000	0.69100700
C	4.68897900	0.09801200	0.10365600
H	4.91507300	0.33189200	-0.93895400
H	4.84595800	1.00983300	0.68449400
H	5.34801000	-0.68913800	0.46326700

(S75)

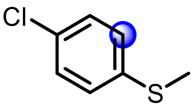

C	-0.96014200	-1.15596300	0.00001900
C	0.42607100	-0.99985400	0.00001200
C	0.99769100	0.27592500	-0.00000600
C	0.15230800	1.39551300	-0.00001400
C	-1.22819100	1.24514800	-0.00000600
C	-1.77831100	-0.03469500	0.00001000
H	-1.39537300	-2.14731800	0.00003300
H	1.04487800	-1.88713500	0.00002100
H	0.57681100	2.39338400	-0.00002800
H	-1.87310200	2.11468600	-0.00001300
S	2.74928600	0.59937000	-0.00001300
C	3.49160100	-1.06264100	-0.00002500
H	3.22182800	-1.62204200	-0.89662300
H	4.56899700	-0.89556200	-0.00000200
H	3.22179400	-1.62207100	0.89654500
Cl	-3.52709200	-0.22967400	0.00002100

(S76)

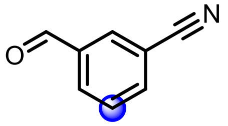

C	0.17148900	-0.81718300	0.00000100
C	-1.08910600	-0.22158100	0.00000100
C	-1.20502000	1.17495000	0.00000100
C	-0.06378600	1.96726400	0.00000000
C	1.19943200	1.37836600	-0.00000100
C	1.32202400	-0.01849100	0.00000000
H	-2.19557600	1.61426200	0.00000100
H	-0.15046000	3.04722300	-0.00000100
H	2.09237700	1.99136400	-0.00000100
C	-2.30102600	-1.08122500	0.00000300
O	-3.43499700	-0.66265500	-0.00000300
H	-2.09696600	-2.17201100	-0.00000400
H	0.26515400	-1.89769900	0.00000200
C	2.61857500	-0.62596600	0.00000000
N	3.66427900	-1.11695800	-0.00000100

(S77)

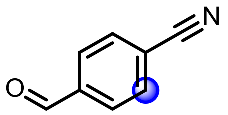

C	-0.52290100	1.35414000	0.00000000
C	0.86241500	1.25296100	0.00000000
C	1.46025400	-0.01512900	-0.00000100
C	0.66537200	-1.17490600	0.00000300
C	-0.71523300	-1.06198600	0.00000500
C	-1.31703300	0.20325600	0.00000000
H	-0.99281600	2.33223200	-0.00000200
H	1.48368200	2.13967400	-0.00000100
H	1.14083000	-2.14781500	0.00000400
H	-1.34731500	-1.94188200	0.00000500
C	2.88729300	-0.12936200	-0.00000200
N	4.03895200	-0.22119000	-0.00000100
C	-2.79677800	0.33393900	0.00000100
O	-3.56588700	-0.59879500	-0.00000700
H	-3.17028700	1.37899600	0.00002600

(S78)

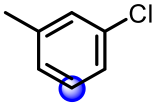

C	0.66109100	1.88049700	0.00236100
C	1.73281400	0.99112400	-0.00848600
C	1.51664700	-0.39097400	-0.01164800
C	0.19957900	-0.86208200	-0.00965200
C	-0.86092900	0.03750900	0.00122300
C	-0.65097100	1.41199100	0.00775700
H	0.84398500	2.94927200	0.00286700
H	2.74808500	1.37334600	-0.01690200
H	-0.00102700	-1.92693600	-0.01855200
H	-1.49106900	2.09441800	0.01268100
Cl	-2.50996600	-0.58242700	0.00056000
C	2.67437500	-1.35979800	0.00945400
H	3.55125000	-0.94064000	-0.48921400
H	2.41845400	-2.29954800	-0.48514100
H	2.96411100	-1.59825800	1.03867600

(S79)

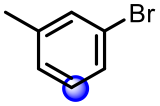

C	0.23884000	0.21252100	0.00107900
C	-0.11803900	1.55639800	0.00775400
C	-1.47386900	1.87926600	0.00232200
C	-2.44249500	0.87864900	-0.00841400
C	-2.07820600	-0.47176800	-0.01149000
C	-0.71697400	-0.79759300	-0.00988300
H	0.63924900	2.32913700	0.01283500
H	-1.77208900	2.92173300	0.00272500
H	-3.49312800	1.14878000	-0.01668300
H	-0.40606600	-1.83537700	-0.01895500
C	-3.12390000	-1.56066500	0.00952300
H	-2.77343600	-2.46109500	-0.49966000
H	-3.37373800	-1.84109900	1.03845500
H	-4.04658100	-1.23322100	-0.47453400
Br	2.10039000	-0.26313500	0.00029900

(S80)

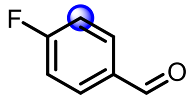

C	-0.16027200	1.33839400	0.00000000
C	-0.98982800	0.21148300	0.00000400
C	-0.42039000	-1.07024300	0.00000600
C	0.95779600	-1.22473900	0.00000300
C	1.75172300	-0.08226200	0.00000000
C	1.22385600	1.20059200	-0.00000200
H	-1.07658600	-1.93243100	0.00000900
H	1.42560900	-2.20127000	0.00000300
C	-2.45868000	0.38413900	0.00000700
O	-3.26286700	-0.52128500	-0.00001100
H	-2.79910000	1.44145200	0.00002000
H	-0.60161800	2.32988000	-0.00000200
H	1.88733600	2.05601400	-0.00000400
F	3.09356300	-0.22972800	-0.00000400

(S81)

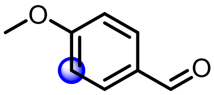

C	-0.54124000	1.27810800	-0.00033700
C	-1.45864200	0.22564500	-0.00008500
C	-0.97849800	-1.09651100	-0.00006600
C	0.37778200	-1.34860700	-0.00025200
C	1.29391400	-0.28067400	-0.00030600
C	0.83090100	1.04010000	-0.00039300
H	0.76660600	-2.35967900	-0.00025200
C	-2.90315000	0.51104500	0.00014500
O	-3.78216400	-0.32524700	0.00052300
H	-3.15806600	1.59286700	-0.00029200
H	-0.90333700	2.30184300	-0.00041600
H	1.51932200	1.87389200	-0.00054200
H	-1.69429900	-1.91017500	0.00010300
O	2.60289900	-0.63474700	-0.00039200
C	3.59658800	0.38610600	0.00081300
H	4.55264700	-0.13376500	0.00151700
H	3.52343200	1.01199900	-0.89424200
H	3.52187800	1.01169300	0.89595100

(S82)

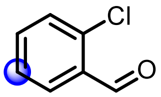

C	-1.28458300	1.40429500	-0.00001100
C	-0.01994300	0.79482800	-0.00001300
C	0.03435700	-0.61147900	-0.00000300
C	-1.13762200	-1.36365600	0.00000900
C	-2.37755700	-0.72956600	0.00001100
C	-2.45705100	0.66176500	0.00000200
H	-1.07122500	-2.44410800	0.00001600
C	1.14676600	1.71639900	-0.00002800
O	2.31630400	1.41890700	0.00003000
H	0.83893800	2.78458000	0.00004900
H	-1.32945800	2.48897600	-0.00001900
H	-3.41964300	1.15836900	0.00000400
H	-3.28035100	-1.32957000	0.00002100
Cl	1.54735900	-1.48500100	-0.00000700

(S83)

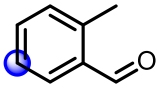

C	-0.74932100	-1.45665200	0.00000100
C	0.31380000	-0.54151600	0.00000300
C	0.05301700	0.85074900	0.00000000
C	-1.28403700	1.25674200	-0.00000200
C	-2.33356700	0.33861100	-0.00000300
C	-2.07054400	-1.02928700	-0.00000100
H	-1.50722700	2.31840900	-0.00000300
C	1.67482400	-1.12074200	0.00000600
O	2.72277800	-0.51067100	-0.00001000
H	1.68042100	-2.23242600	0.00001500
H	-0.52336200	-2.51862300	0.00000100
H	-2.88178500	-1.74757100	-0.00000100
H	-3.35765400	0.69549600	-0.00000400
C	1.14592700	1.88777300	0.00000400
H	1.79618100	1.78265600	0.87133600
H	1.79618900	1.78265500	-0.87132100
H	0.71442600	2.89071200	0.00000200

(S84)

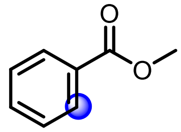

C	2.47922800	1.01492600	0.00002700
C	2.99319400	-0.28220900	0.00001400
C	2.12804200	-1.37612300	-0.00001500
C	0.75036400	-1.17729200	-0.00002700
C	0.23192400	0.12305800	-0.00001100
C	1.10349000	1.21813800	0.00001400
H	3.15097300	1.86570000	0.00004300
H	4.06600100	-0.44010800	0.00002400
H	2.52703600	-2.38405400	-0.00002900
H	0.07346800	-2.02130000	-0.00004500
H	0.68493600	2.21697500	0.00002900
C	-1.23351400	0.39693200	-0.00002700
O	-1.72532200	1.50269000	-0.00004100
O	-1.97210800	-0.73510000	0.00002000
C	-3.39888800	-0.55297300	0.00003200
H	-3.71341500	-0.00418200	-0.88871600
H	-3.81920300	-1.55636400	0.00006400
H	-3.71339400	-0.00413500	0.88876000

(S85)

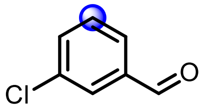

C	-0.05904000	-0.79756200	-0.00006900
C	1.20020000	-0.18970500	-0.00016500
C	1.31041800	1.20613600	-0.00014200
C	0.16135900	1.98557100	-0.00003100
C	-1.10107600	1.38724400	0.00002500
C	-1.19786400	-0.00109200	-0.00000100
H	-0.14728300	-1.87802100	-0.00007100
H	2.29698500	1.65318300	-0.00020900
H	0.23535700	3.06682400	-0.00000100
H	-1.99987400	1.99081200	0.00010300
C	2.41111000	-1.04754300	-0.00028600
O	3.54751200	-0.63340300	0.00048600
H	2.20565800	-2.13853200	-0.00113800
Cl	-2.78362200	-0.75796100	0.00008500

(S86)

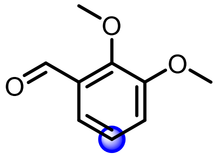

C	-0.04659200	-0.31512700	0.02670100
C	1.29943300	0.08426800	0.08834700
C	1.63137500	1.43629600	-0.08236600
C	0.64267100	2.37813300	-0.31888600
C	-0.69655400	1.98308600	-0.36443400
C	-1.04881200	0.64973100	-0.18040100
H	2.67731800	1.71370600	-0.02992300
H	0.90182100	3.42060300	-0.46128600
H	-1.48891300	2.70200900	-0.53925900
C	2.37401800	-0.90992000	0.33054800
O	3.55370500	-0.62818500	0.37501300
H	2.03556900	-1.94975200	0.47675600
O	-2.37261800	0.28276300	-0.25169300
C	-2.98877100	-0.01214300	1.01192100
H	-2.98496600	0.87420500	1.65477200
H	-4.01706500	-0.29443700	0.78912400
H	-2.47812700	-0.83680500	1.51561100
O	-0.37703100	-1.63202200	0.22446800
C	-0.85879300	-2.33368200	-0.93579900
H	-1.10227900	-3.33995500	-0.59762700
H	-1.74795000	-1.85043900	-1.34503800
H	-0.07571700	-2.38342900	-1.69922100

(S87)

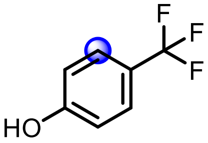

C	-0.36811900	0.00605600	-0.03511200
C	0.32678300	-1.20737900	-0.02367900
C	1.71316200	-1.22194700	-0.00597300
C	2.41915600	-0.01577800	0.00369100
C	1.73025800	1.19879300	-0.00669200
C	0.33948400	1.20664100	-0.02356300
H	-0.21871700	-2.14302600	-0.03614700
H	2.27728900	2.13644100	-0.00370800
H	-0.19222400	2.14948700	-0.03564400
O	3.78003600	-0.09056000	0.01925800
H	4.16115200	0.79406200	0.02502400
H	2.26424000	-2.15400500	-0.00186200
C	-1.86738800	0.00696000	-0.00182600
F	-2.39319000	1.16497900	-0.46344100
F	-2.35320800	-0.15883800	1.25802700
F	-2.39716200	-0.99487000	-0.74378700

(S88)

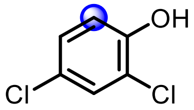

C	0.89925200	-0.36589800	-0.00007300
C	-0.39977000	-0.86147600	-0.00006900
C	-1.45718200	0.03937400	0.00022900
C	-1.22621900	1.41289200	0.00027100
C	0.07823000	1.88908700	-0.00025700
C	1.16198700	1.00844500	-0.00036100
H	-2.05949400	2.10331300	0.00043600
H	-0.57610200	-1.92829500	0.00005000
H	0.28100500	2.95295300	-0.00016100
O	2.41433800	1.52893400	0.00020200
H	3.06207400	0.81133500	-0.00014300
Cl	-3.10540600	-0.57020200	-0.00005400
Cl	2.26070100	-1.48305200	0.00004000

(S89)

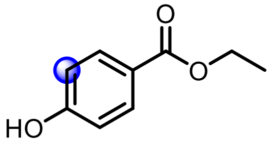

C	1.97008700	-1.35723700	0.00014200
C	0.62716800	-1.01077000	0.00016400
C	0.23552900	0.33543800	0.00008100
C	1.22028100	1.32905500	-0.00009200
C	2.56668300	0.99151000	-0.00012000
C	2.94382300	-0.35491400	0.00005700
H	2.28398300	-2.39381700	0.00019500
H	0.91299700	2.36755300	-0.00020700
H	-0.12872500	-1.78487400	0.00026300
C	-1.18843700	0.75818900	0.00009400
O	-1.56228400	1.91107800	0.00022000
O	-2.04080100	-0.29138100	-0.00009400
C	-3.45308900	0.02736700	-0.00012700
H	-3.67419900	0.63240600	-0.88240400
H	-3.67422200	0.63249500	0.88207900
C	-4.21846400	-1.28006700	-0.00008100
H	-5.29273700	-1.07662900	-0.00012000
H	-3.98279500	-1.87316500	-0.88669700
H	-3.98284400	-1.87308100	0.88660500
H	3.32489800	1.76897500	-0.00028000
O	4.24873100	-0.75103300	-0.00010400
H	4.82699400	0.01939800	-0.00032400

(S90)

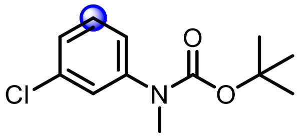

C	-0.25405200	-1.71865900	1.13793900
H	-0.25385600	-2.50184500	0.37269600
N	-0.17976900	-0.37195200	0.55623500
C	-1.31121500	0.23921400	0.03740400
O	-1.32159700	1.32223500	-0.50903700
O	-2.39341600	-0.54696500	0.22716900
C	-3.73951800	-0.12621400	-0.22373800
C	-3.75630300	0.03255100	-1.74580300
H	-4.78256000	0.21096300	-2.07835700
H	-3.13500500	0.86748500	-2.06521500
H	-3.39920700	-0.88130500	-2.22778800
C	-4.61066400	-1.30704000	0.20656300
H	-4.56728700	-1.44582900	1.28923300
H	-5.65006400	-1.12543000	-0.07695600
H	-4.27671600	-2.22860200	-0.27539100
C	-4.16355900	1.15258800	0.50250800
H	-5.20734300	1.37365300	0.26309800
H	-4.08680100	1.01973900	1.58493000
H	-3.55014800	2.00116200	0.20441000
H	-1.16515400	-1.81271600	1.72258200
H	0.60425600	-1.86011400	1.79354000
C	1.10512400	0.21120500	0.35571500
C	1.32557900	1.58370000	0.52615300
C	2.18146900	-0.61796900	0.01670900
C	2.60219400	2.10607000	0.35446200
H	0.50078400	2.23255600	0.77805800
C	3.44945900	-0.06902500	-0.13677300
H	2.03906300	-1.67806400	-0.14070200
C	3.68343600	1.28966500	0.02482900
H	2.76228800	3.16967900	0.48907100
Cl	4.78799900	-1.13481600	-0.55925700
H	4.67854200	1.69551800	-0.10035900

(S91)

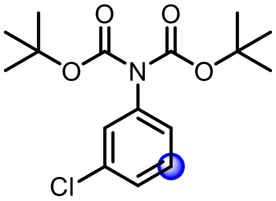

C	4.35177200	0.57654300	0.94769500
C	3.43388600	0.93669200	1.93171900
C	2.06718700	0.78537400	1.71529500
C	1.61894500	0.27371500	0.50227700
C	2.51908700	-0.08921600	-0.49493700
C	3.87949100	0.06530100	-0.25669500
H	5.41589600	0.68899300	1.11017000
H	3.79136900	1.33277700	2.87493600
H	1.35029700	1.05379400	2.48131400
H	2.16282200	-0.47704400	-1.43999200
Cl	5.02627900	-0.39038900	-1.51089000
N	0.20043900	0.11503000	0.26713600
C	-0.49145700	1.28056800	-0.14045000
O	0.09158800	2.21487200	-0.63723500
O	-1.78790100	1.22550900	0.16273600
C	-0.31956100	-1.18417200	0.47491700
O	0.27086700	-1.99675500	1.14659100
O	-1.44783500	-1.38615700	-0.20458100
C	-2.70924800	2.32756800	-0.21844100
C	-2.18976100	-2.66884400	-0.09253600
C	-2.31396000	3.61216100	0.51335500
H	-1.35287200	3.98916400	0.16814000
H	-3.07528100	4.37625000	0.33404600
H	-2.26075100	3.43526000	1.59073200
C	-2.72229900	2.49667000	-1.73865700
H	-3.49783400	3.21712700	-2.01221100
H	-1.76545800	2.86134700	-2.10908500
H	-2.95528400	1.54653500	-2.22565700
C	-4.05784100	1.81314400	0.28364200
H	-4.83573100	2.55207000	0.07742700
H	-4.32520700	0.88016600	-0.21602400
H	-4.02622200	1.63481000	1.36066800
C	-1.33930800	-3.81037200	-0.65339900
H	-0.46156700	-3.99392600	-0.03618500
H	-1.94068500	-4.72290600	-0.68813000
H	-1.01649700	-3.57988200	-1.67192300
C	-2.60965800	-2.90631400	1.35904000
H	-3.16191600	-2.04448500	1.74190500
H	-3.26770600	-3.77820300	1.40401900
H	-1.74796400	-3.08947000	1.99901900
C	-3.40909700	-2.41571800	-0.97831700
H	-3.10190700	-2.18790500	-2.00132300
H	-4.04510100	-3.30390700	-0.99741300
H	-3.99504100	-1.57752600	-0.59640200

(S92)

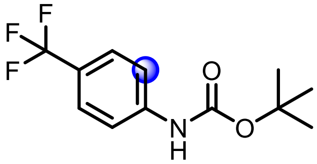

C	2.54201700	-1.36626800	-0.02663900
C	1.17145500	-1.57139200	-0.01754200
C	0.28300900	-0.48399400	-0.01760000
C	0.79847700	0.81970700	-0.03026600
C	2.17487100	1.01441700	-0.03914200
C	3.05407400	-0.06772800	-0.04028400
H	3.21279800	-2.21620100	-0.03128900
H	0.78262600	-2.58448000	-0.01252200
H	0.12442200	1.66202600	-0.03473000
H	2.56417900	2.02487800	-0.05317300
N	-1.08804800	-0.77586000	-0.00873800
H	-1.34555900	-1.75150500	-0.00134600
C	-2.15437300	0.10052900	-0.00662500
O	-2.07541500	1.30841900	-0.01402000
O	-3.29043400	-0.62929400	0.00501500
C	-4.62628800	0.01393800	0.01075800
C	-4.79584400	0.85067700	1.28061400
H	-4.60670600	0.24046700	2.16753000
H	-4.12243500	1.70638700	1.28889200
H	-5.82453800	1.21691100	1.33596000
C	-4.81321200	0.83784500	-1.26504200
H	-4.63538000	0.21882200	-2.14820100
H	-5.84288200	1.20272200	-1.31042400
H	-4.14064700	1.69387300	-1.29097500
C	-5.56769700	-1.19003200	0.02325100
H	-6.60571100	-0.84958300	0.02888500
H	-5.41185000	-1.81087000	-0.86166500
H	-5.39937500	-1.80214300	0.91195600
C	4.53550900	0.15873600	0.00755800
F	5.22748800	-0.83610600	-0.59742700
F	4.89971800	1.31695800	-0.58959300
F	5.00415000	0.22449300	1.28369300

(S93)

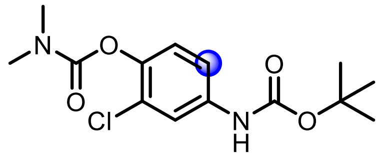

N	2.13879600	0.61664400	-0.01646000
H	2.37526400	1.56442100	-0.26812100
C	3.21498100	-0.23466500	0.09437300
O	3.16247000	-1.41050100	0.38090400
O	4.33588300	0.47486500	-0.17121900
C	5.67530100	-0.15561400	-0.14794700
C	5.98268000	-0.67922400	1.25691500
H	5.86209300	0.11786100	1.99507800
H	5.33190300	-1.51047200	1.52354700
H	7.02040400	-1.02159500	1.29508300
C	5.75363000	-1.25290700	-1.21186400
H	5.47667900	-0.85439600	-2.19125800
H	6.78109700	-1.62155400	-1.27479700
H	5.09826800	-2.08867200	-0.97142800
C	6.59296200	1.01384000	-0.50443600
H	7.63259700	0.67849900	-0.52443100
H	6.34014800	1.41678800	-1.48764500
H	6.50161700	1.81445700	0.23293100
C	0.77347800	0.32638900	0.15570100
C	0.28471600	-0.94438500	0.48411400
C	-0.12762600	1.38515200	-0.01583300
C	-1.08504300	-1.13253800	0.62792700
H	0.97038400	-1.76595100	0.61933400
C	-1.49159600	1.17577500	0.13349300
H	0.22846900	2.37716300	-0.26776700
C	-1.98321200	-0.08797600	0.45602100
H	-1.47470800	-2.11199600	0.87758900
Cl	-2.59231300	2.52060300	-0.08215600
O	-3.33549700	-0.28831700	0.68150000
C	-4.10911500	-0.65226000	-0.41081100
O	-3.63728100	-0.86318500	-1.50462200
N	-5.42115600	-0.74063400	-0.06982000
C	-5.95492300	-0.47921600	1.26095500
H	-6.75466700	0.26609900	1.19797900
H	-6.37230300	-1.39650300	1.69207700
H	-5.17478900	-0.10386800	1.91477700
C	-6.38001000	-1.15792100	-1.08347500
H	-6.87559400	-2.08676200	-0.78012600
H	-7.14427800	-0.38592500	-1.22220900
H	-5.85868100	-1.31950900	-2.02328700

(S94)

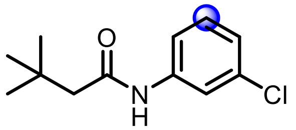

N	-0.24762800	-0.10054800	0.58054400
H	-0.32701200	-1.08490100	0.78424500
C	-1.42637300	0.61972200	0.62938200
O	-1.48792000	1.81649300	0.40635900
C	-3.64005800	-0.47031800	-0.19462900
C	-4.28126700	0.84506600	-0.67295800
H	-4.83269400	1.32940000	0.13851200
H	-3.53300300	1.55353200	-1.03015500
H	-4.98607800	0.64406000	-1.48562300
C	-2.90850700	-1.14955500	-1.36694500
H	-2.44472300	-2.09282200	-1.05830500
H	-3.61192500	-1.37794900	-2.17271700
H	-2.12776400	-0.50709300	-1.78327800
C	-4.74408200	-1.40903800	0.32832700
H	-5.48180400	-1.60784000	-0.45471300
H	-4.33320700	-2.37097200	0.65169200
H	-5.27163500	-0.96444300	1.17772700
C	1.05279300	0.33630800	0.25695600
C	1.36915300	1.66391000	-0.06034100
C	2.06513800	-0.63550700	0.26018400
C	2.68692500	1.99256200	-0.36870800
H	0.59268200	2.41203600	-0.06182000
C	3.36650100	-0.27232800	-0.05176000
H	1.83870100	-1.66730800	0.50304400
C	3.70186000	1.03910200	-0.37076900
H	2.92720000	3.02087100	-0.61375800
Cl	4.61831700	-1.51033000	-0.04231000
H	4.72312200	1.30209000	-0.61179500
C	-2.65369200	-0.21041100	0.98493600
H	-3.18201300	0.33712000	1.77066600
H	-2.35483700	-1.17535700	1.40881700

(S95)

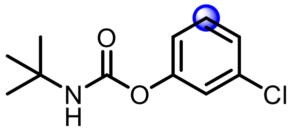

C	-2.11146600	0.68414300	-0.20359900
C	-3.40450600	0.21979100	0.00787800
C	-3.69000900	-1.13997800	0.06131600
C	-2.64533000	-2.04709100	-0.10790400
C	-1.34117500	-1.61348300	-0.33017200
C	-1.08895100	-0.24501500	-0.37217100
H	-1.89230900	1.74254800	-0.24328800
H	-4.70347500	-1.47925900	0.22977500
H	-2.85334600	-3.10997200	-0.06811200
H	-0.53399200	-2.32095100	-0.45455800
Cl	-4.70499600	1.38727600	0.21478600
O	0.16369700	0.27480400	-0.66774000
C	1.28242300	-0.16683200	0.02939700
O	1.24525400	-0.97296600	0.92811800
N	2.37041000	0.46044500	-0.47295300
H	2.19878500	1.11791100	-1.21782300
C	3.76027500	0.29560200	0.01855900
C	3.85535000	0.72858400	1.49212300
H	4.88565300	0.63155200	1.84472300
H	3.55070100	1.77229200	1.60732700
H	3.21568000	0.10815900	2.12049600
C	4.62736700	1.21276800	-0.85579500
H	5.67253200	1.14295200	-0.54838900
H	4.56690400	0.92553100	-1.90989400
H	4.31839100	2.25811200	-0.75903600
C	4.20898800	-1.16640900	-0.14859400
H	4.15201300	-1.46830500	-1.19788200
H	5.24390200	-1.27972600	0.18549000
H	3.58122200	-1.83483100	0.44122100

(S96)

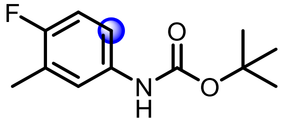

C	-2.09609800	-1.11352100	-0.00002800
C	-1.09890700	-0.12724800	-0.00007900
C	-1.46844000	1.22181700	-0.00011200
C	-2.82116800	1.55677200	-0.00008100
C	-3.77724900	0.55928900	-0.00001800
C	-3.45343400	-0.79548500	0.00000700
H	-1.80883300	-2.16119500	-0.00003500
H	-0.71076200	1.98967900	-0.00015100
H	-3.13389000	2.59378900	-0.00012200
C	-4.52721700	-1.85177600	0.00013500
H	-5.17130900	-1.76048300	0.87913700
H	-5.17248600	-1.75952200	-0.87788800
H	-4.08834200	-2.85085500	-0.00068100
F	-5.09375400	0.91058600	0.00000400
N	0.24002300	-0.56835600	-0.00016600
H	0.38915700	-1.56584000	-0.00039600
C	1.38925500	0.18465100	0.00001900
O	1.44612000	1.39546500	0.00032300
O	2.44562500	-0.66408600	-0.00022300
C	3.83830600	-0.16520700	-0.00000700
C	4.65011900	-1.46069500	-0.00011900
H	5.71831900	-1.23109100	-0.00004500
H	4.42428200	-2.05738000	0.88648500
H	4.42436500	-2.05719000	-0.88687200
C	4.10462100	0.64304700	-1.27225500
H	3.85361100	0.05300500	-2.15755300
H	3.52614500	1.56547100	-1.28577600
H	5.16724500	0.89551800	-1.32422400
C	4.10448100	0.64265700	1.27251800
H	3.85324100	0.05239300	2.15760500
H	5.16714400	0.89491800	1.32472100
H	3.52616300	1.56517100	1.28623600

(S97)

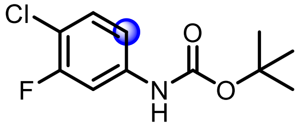

C	1.68320400	1.28331100	0.00010100
C	0.72963600	0.25626200	0.00019200
C	1.16139100	-1.07709700	0.00026800
C	2.52326800	-1.36093200	0.00015300
C	3.47440500	-0.34579200	0.00002500
C	3.03067700	0.97263200	0.00002400
H	1.39400700	2.32807000	0.00026900
H	0.43756600	-1.87663700	0.00041300
H	2.85593800	-2.39130000	0.00017800
N	-0.62122400	0.63822000	0.00017900
H	-0.81631400	1.62793800	0.00025900
C	-1.73895600	-0.16930100	0.00006600
O	-1.73566200	-1.38017500	-0.00023600
O	-2.82820100	0.62984400	0.00060700
C	-4.20067000	0.06974600	-0.00006000
C	-5.06700000	1.32889600	-0.00015100
H	-6.12387500	1.05235800	-0.00055000
H	-4.86794000	1.93434000	0.88680200
H	-4.86728800	1.93458500	-0.88678200
C	-4.42882700	-0.74834600	-1.27299900
H	-4.20597000	-0.14663800	-2.15791400
H	-3.81014900	-1.64430700	-1.28844900
H	-5.47882400	-1.04874900	-1.32467800
C	-4.42938300	-0.74875800	1.27251500
H	-4.20720300	-0.14718400	2.15767500
H	-5.47924800	-1.04973200	1.32362900
H	-3.81029500	-1.64446200	1.28799200
Cl	5.18353500	-0.70597600	-0.00015300
F	3.92138800	1.98274200	-0.00014100

(S98)

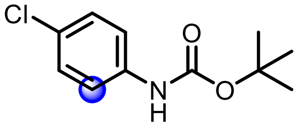

C	3.25082400	-1.28730800	-0.00008700
C	1.87967700	-1.50982500	0.00017900
C	0.97475400	-0.43868900	0.00044500
C	1.47151000	0.87085600	0.00056700
C	2.84664300	1.09211700	0.00029100
C	3.72883900	0.01878300	-0.00007200
H	3.93994500	-2.12195600	-0.00029700
H	1.50935300	-2.53012900	0.00026500
H	0.78613900	1.70442000	0.00094200
H	3.22697500	2.10571400	0.00036700
N	-0.39665500	-0.75154100	0.00073500
H	-0.63924400	-1.73072400	0.00053900
C	-1.47177200	0.10769800	0.00030400
O	-1.41176800	1.31780500	0.00009200
O	-2.60147200	-0.63695100	0.00019200
C	-3.94250000	-0.00941900	-0.00022200
C	-4.13204600	0.81925600	1.27244500
H	-3.94197900	0.20669600	2.15760000
H	-3.46822200	1.68227300	1.28981600
H	-5.16540100	1.17320400	1.32213500
C	-4.13139900	0.81916400	-1.27303200
H	-3.94068300	0.20660900	-2.15805200
H	-5.16481700	1.17284900	-1.32334900
H	-3.46776700	1.68232900	-1.29008700
C	-4.87146200	-1.22338900	-0.00045900
H	-5.91323000	-0.89438900	-0.00076800
H	-4.70214000	-1.83818600	-0.88715600
H	-4.70264400	-1.83812400	0.88637600
Cl	5.46516500	0.31046500	-0.00046500

(S99)

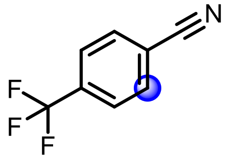

C	-2.14106600	0.00000100	-0.00251800
C	-1.44088700	-1.21436500	-0.01135700
C	-0.05223200	-1.21136500	-0.02640600
C	0.64015800	0.00001600	-0.03574300
C	-0.05224300	1.21138700	-0.02640500
C	-1.44090100	1.21437200	-0.01135800
H	-1.98633300	-2.14946000	-0.00820800
H	0.49089400	-2.14758800	-0.03902700
H	0.49087500	2.14761400	-0.03902400
H	-1.98635500	2.14946300	-0.00820900
C	2.14749000	0.00000400	0.00004200
C	-3.57292400	-0.00000600	0.01078600
N	-4.72797500	-0.00001100	0.02163800
F	2.61251100	-0.00035800	1.27351300
F	2.66944000	1.08805200	-0.60530100
F	2.66942400	-1.08772000	-0.60590600

(S100)

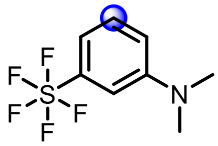

C	-0.84313600	-0.33681700	-0.03792600
C	0.23825300	0.52789800	-0.01126300
C	0.11998300	1.91021200	0.01085900
C	-1.17566500	2.42369500	0.01003600
C	-2.28998400	1.59692400	-0.01719200
C	-2.15386700	0.19059600	-0.05341900
H	-0.67728800	-1.40108400	-0.04701100
H	0.98174700	2.55932300	0.03350700
H	-1.31584200	3.49824300	0.03446800
H	-3.27138800	2.04909500	-0.01002900
S	1.92473600	-0.20716200	0.00296500
F	1.55786000	-1.28155500	-1.19245800
F	1.51559300	-1.33558500	1.13307500
F	3.42753200	-0.86044900	0.01554100
F	2.43860100	0.79889900	1.19921600
F	2.48132400	0.85267600	-1.12516600
N	-3.25180100	-0.64531500	-0.10885800
C	-4.58660400	-0.09114100	0.05862600
H	-5.31777500	-0.89042400	-0.05006200
H	-4.72925800	0.37392200	1.04414400
H	-4.80212300	0.65795900	-0.70911400
C	-3.07255500	-2.08312200	0.03320300
H	-2.64271200	-2.35889500	1.00593600
H	-4.04058000	-2.57185300	-0.06259800
H	-2.42471200	-2.47983500	-0.75407800

(S101)

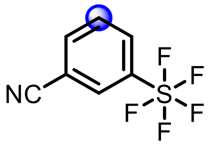

C	-1.18868600	-0.53971300	0.00000100
C	-0.16775100	0.39740100	0.00000600
C	-0.41133400	1.76587800	0.00000500
C	-1.73318400	2.20678800	-0.00000400
C	-2.78232900	1.29630100	-0.00000900
C	-2.51203800	-0.07910700	0.00001000
H	-0.98313700	-1.60025500	0.00000400
H	0.40321700	2.47603500	0.00000300
H	-1.93860200	3.27019100	-0.00000800
H	-3.80960100	1.63843400	-0.00000500
S	1.57015300	-0.19747000	0.00000100
F	1.25811400	-1.31651900	-1.15980800
F	1.25808900	-1.31658400	1.15973300
F	3.11304600	-0.72168700	0.00000200
F	2.00522900	0.88065100	1.15970100
F	2.00525600	0.88071400	-1.15963600
C	-3.58907200	-1.02225100	0.00000300
N	-4.46365100	-1.77654700	-0.00000500

(S102)

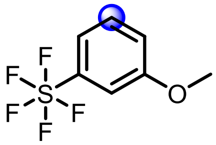

C	-1.00372500	-0.60103800	-0.00002100
C	-0.02055700	0.37004500	-0.00000600
C	-0.29926200	1.73432500	0.00000500
C	-1.63631600	2.11209700	-0.00000100
C	-2.66093400	1.16539300	-0.00001600
C	-2.34537300	-0.19572600	-0.00002300
H	-0.77536000	-1.65671100	-0.00002500
H	0.48852200	2.47220700	0.00001500
H	-1.88785100	3.16606900	0.00000400
H	-3.69000300	1.49744700	-0.00002400
S	1.73524700	-0.17260700	0.00000300
F	1.46825200	-1.30639500	-1.16171000
F	1.46818500	-1.30659700	1.16150100
F	3.30005700	-0.65010200	0.00001000
F	2.14679500	0.91665300	1.16210300
F	2.14687200	0.91685400	-1.16188300
O	-3.25413600	-1.20464900	-0.00003800
C	-4.64066100	-0.87843600	0.00004600
H	-5.16676300	-1.83087300	0.00009700
H	-4.91489000	-0.31144400	0.89552400
H	-4.91500900	-0.31147700	-0.89541700

(S103)

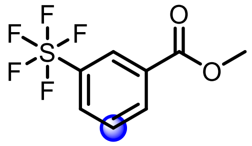

C	-0.66632700	-0.07130000	0.00000200
C	0.54298600	0.60720800	-0.00000100
C	0.62210000	1.99511500	-0.00000200
C	-0.56452400	2.72569300	-0.00000100
C	-1.79207100	2.07348400	0.00000100
C	-1.84685700	0.67723600	0.00000200
H	-0.71027500	-1.14954700	0.00000300
H	1.57834700	2.49825600	-0.00000300
H	-0.52161700	3.80802100	-0.00000200
H	-2.72115400	2.62957400	0.00000000
S	2.09765600	-0.36816700	0.00000000
F	1.54165000	-1.39034600	-1.16136100
F	1.54163500	-1.39037100	1.16133100
F	3.48196700	-1.23475500	0.00000000
F	2.77578600	0.57928800	1.16126900
F	2.77580300	0.57931200	-1.16124000
C	-3.19187000	0.02309200	0.00000500
O	-4.23680900	0.63015900	-0.00000100
O	-3.10648000	-1.31908600	0.00000300
C	-4.35856200	-2.03383500	-0.00000400
H	-4.08880000	-3.08709200	-0.00001300
H	-4.93675000	-1.78272700	0.88975000
H	-4.93674900	-1.78271200	-0.88975300

(S104)

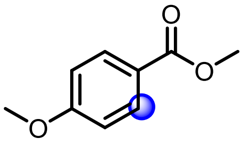

C	2.09922200	-0.31786400	0.00002800
C	1.21086200	-1.40334700	-0.00001500
C	-0.15508600	-1.18377700	0.00002300
C	-0.66759300	0.12352200	0.00003400
C	0.22431100	1.19796000	0.00008500
C	1.59987300	0.99005100	0.00009800
H	1.61947600	-2.40646600	-0.00003800
H	-0.83712000	-2.02369800	-0.00000400
H	-0.17439500	2.20507100	0.00011700
H	2.26542700	1.84233500	0.00015400
C	-2.12197900	0.41579400	-0.00001100
O	-2.60472700	1.52711900	-0.00011300
O	-2.87660600	-0.70893000	0.00008000
O	3.41988800	-0.63709700	-0.00003400
C	4.38243300	0.41189700	-0.00007000
H	5.35383900	-0.07909100	-0.00017900
H	4.29070000	1.03618200	-0.89477800
H	4.29084600	1.03610900	0.89468900
C	-4.29986500	-0.50803800	-0.00004000
H	-4.73381700	-1.50581000	0.00013400
H	-4.60827100	0.04491200	0.88841900
H	-4.60818200	0.04453200	-0.88877200

(S105)

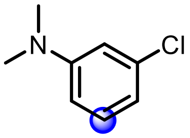

C	-1.66030100	1.41132400	0.01915600
C	-0.48908300	2.16658100	0.01234600
C	0.76512900	1.57062800	-0.02385400
C	0.89518700	0.16411400	-0.06502900
C	-0.29063400	-0.60342700	-0.04099000
C	-1.52508800	0.02900800	-0.00570800
H	-2.63671700	1.87479900	0.04953400
H	-0.55617400	3.24863400	0.03978300
H	1.64146800	2.20256200	-0.02130400
H	-0.26071600	-1.68220800	-0.05044900
N	2.13786200	-0.44678000	-0.13804700
C	3.33759400	0.34798300	0.07306200
H	3.38242600	0.79186200	1.07836000
H	4.21146600	-0.28878000	-0.05860600
H	3.40780200	1.15570000	-0.66061700
C	2.23943900	-1.88666100	0.04092800
H	1.64923800	-2.41805500	-0.71074100
H	3.27834900	-2.18729100	-0.08811600
H	1.90588600	-2.21616400	1.03584100
Cl	-2.97773600	-0.97358300	0.01724600

(S107)

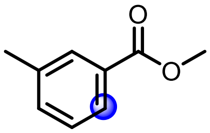

C	-2.48836300	0.99458700	0.00004000
C	-1.44084900	1.91173400	-0.00003400
C	-0.12011600	1.47037200	-0.00005900
C	0.14344600	0.09774300	-0.00000900
C	-0.91699300	-0.81669400	0.00006500
C	-2.24238300	-0.38538200	0.00009100
H	-3.51323500	1.35318100	0.00005900
H	-1.65428800	2.97473300	-0.00007200
H	0.69903300	2.17678600	-0.00011700
H	-0.68233100	-1.87478300	0.00010400
C	1.53033400	-0.44861500	-0.00003200
O	1.80537300	-1.62748300	0.00000900
O	2.47054800	0.52282800	-0.00010300
C	-3.38672500	-1.37055900	0.00017000
H	-3.02390900	-2.39979400	0.00023100
H	-4.02188000	-1.23811200	0.88136600
H	-4.02190300	-1.23822600	-0.88102700
C	3.83648500	0.07293700	-0.00012900
H	4.43984200	0.97820400	-0.00018800
H	4.04116800	-0.52570500	0.88857800
H	4.04110900	-0.52578200	-0.88879700

(S106)

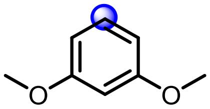

C	0.00000000	2.31952100	0.00000000
C	-1.21253500	1.63835800	0.00017400
C	-1.20749800	0.24221300	0.00010800
C	0.00000000	-0.46748100	0.00000000
C	1.20749800	0.24221300	-0.00010800
C	1.21253500	1.63835800	-0.00017400
H	0.00000000	3.40380900	0.00000000
H	-2.16069900	2.16059200	0.00020600
H	0.00000000	-1.54636300	0.00000000
H	2.16069900	2.16059200	-0.00020600
O	2.43563300	-0.35740500	-0.00044100
O	-2.43563300	-0.35740500	0.00044100
C	-2.51093700	-1.77479700	-0.00043800
H	-2.04458300	-2.20314800	0.89395500
H	-3.57285400	-2.01590700	-0.00040200
H	-2.04465000	-2.20178100	-0.89541400
C	2.51093700	-1.77479700	0.00043800
H	3.57285400	-2.01590700	0.00040200
H	2.04465100	-2.20178200	0.89541400
H	2.04458200	-2.20314800	-0.89395500

(S108)

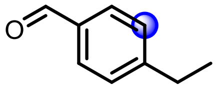

C	-0.71570800	1.36068300	-0.02021200
C	-1.49544900	0.20011700	0.00662300
C	-0.87427800	-1.04698000	-0.14713500
C	0.49934000	-1.12115300	-0.32127300
C	1.29156400	0.03834200	-0.34640000
C	0.66199800	1.27907800	-0.19537000
H	-1.48722500	-1.94070600	-0.13093300
H	0.97276200	-2.08999600	-0.44584100
C	-2.95780100	0.30419100	0.19190800
O	-3.72440700	-0.63364500	0.23032600
H	-3.33523700	1.34361700	0.30045600
H	-1.19340700	2.32932800	0.09310800
H	1.25652200	2.18660600	-0.21982200
C	2.79230100	-0.05722000	-0.49640800
H	3.16806100	0.85140500	-0.97654400
H	3.03735200	-0.88957300	-1.16365900
C	3.51291100	-0.25690700	0.84957300
H	4.59431700	-0.32345500	0.70284300
H	3.31267100	0.57585800	1.52887200
H	3.18016600	-1.17482300	1.34107800

(S109)

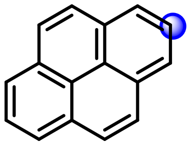

C	3.51955600	0.00000000	0.00036800
C	2.82977400	-1.20908200	0.00030600
C	1.42740800	-1.23447100	0.00015700
C	0.71271600	0.00000000	0.00007200
C	1.42740700	1.23447200	0.00013300
C	2.82977100	1.20908400	0.00028000
C	0.67958800	-2.46124400	0.00009200
C	-0.71271600	0.00000000	-0.00007400
C	-1.42740600	-1.23447200	-0.00013700
C	-0.67958500	-2.46124400	-0.00005000
C	-2.82977200	-1.20908400	-0.00028000
H	-3.37609000	-2.14640600	-0.00032700
C	-3.51955600	-0.00000300	-0.00036000
C	-2.82977300	1.20908200	-0.00030000
C	-1.42740900	1.23447100	-0.00015700
C	-0.67958800	2.46124500	-0.00009400
C	0.67958400	2.46124500	0.00004300
H	1.22702700	3.39809800	0.00008400
H	-1.22703200	3.39809700	-0.00015700
H	1.22702900	-3.39809800	0.00015500
H	4.60381700	0.00000200	0.00048300
H	3.37609300	-2.14640300	0.00037500
H	3.37609400	2.14640300	0.00032600
H	-1.22702800	-3.39809700	-0.00010100
H	-4.60381600	-0.00000100	-0.00047000
H	-3.37609500	2.14640100	-0.00036400

(S110)

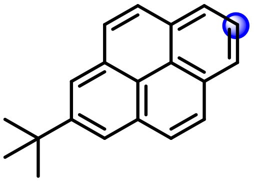

C	-2.25166900	0.03306600	0.00003800
C	-1.52282500	1.22466500	0.00005100
C	-0.11853300	1.24167800	0.00004300
C	0.59263900	0.01094500	0.00002800
C	-0.13688100	-1.21223100	0.00003800
C	-1.53551700	-1.17213600	0.00003800
C	0.63417600	2.46549500	0.00003500
C	2.01656000	0.00071200	0.00000300
C	2.73782300	1.23072200	-0.00000900
C	1.99433400	2.45995700	0.00000600
C	4.14060000	1.19538600	-0.00003000
H	4.69407200	2.12856300	-0.00004200
C	4.82092000	-0.01871500	-0.00003800
C	4.12260600	-1.22400200	-0.00002500
C	2.72073800	-1.23941600	-0.00000500
C	1.95953400	-2.45933100	0.00001100
C	0.60044300	-2.44716300	0.00003500
H	0.04537900	-3.37970900	0.00004900
H	2.49783300	-3.40155600	0.00000300
H	0.09175100	3.40544300	0.00004700
H	-2.03600200	2.17788000	0.00010200
H	-2.06773000	-2.11722300	0.00004300
H	2.54494900	3.39503100	-0.00000800
H	5.90518600	-0.02673100	-0.00005100
H	4.66313600	-2.16474600	-0.00003000
C	-3.79200000	0.00343900	-0.00002200
C	-4.29249900	-0.73701200	-1.26220700
H	-3.93125200	-1.76717300	-1.30021300
H	-5.38648900	-0.76673000	-1.27574800
H	-3.95491100	-0.23096100	-2.17085800
C	-4.29263700	-0.73708800	1.26208600
H	-3.95502500	-0.23116300	2.17079300
H	-5.38662900	-0.76666500	1.27554100
H	-3.93150700	-1.76729000	1.29999000
C	-4.40739500	1.41498900	-0.00000700
H	-4.11874100	1.98637000	0.88649400
H	-4.11864900	1.98641100	-0.88645700
H	-5.49788200	1.33648700	-0.00007400

(S111)

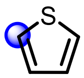

C	-0.00013500	-0.01141400	1.24122500
C	-0.00013500	-1.27146100	0.71386000
C	-0.00013500	-1.27146100	-0.71386000
C	-0.00013500	-0.01141400	-1.24122500
S	0.00004300	1.19791200	0.00000000
H	0.00079200	0.28293000	2.27928100
H	0.00048100	-2.16898100	1.31814400
H	0.00048100	-2.16898100	-1.31814400
H	0.00079200	0.28293000	-2.27928100

(S112)

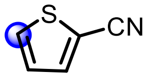

C	1.92712500	-0.22918900	0.00000500
C	1.64114500	1.10943800	0.00003200
C	0.24717500	1.37107000	-0.00004600
C	-0.50886800	0.22075800	0.00001500
S	0.50392600	-1.20388000	-0.00001200
H	2.89643700	-0.70440300	0.00001600
H	2.39943500	1.88038200	0.00005200
H	-0.19287300	2.35873800	-0.00007200
C	-1.91762300	0.11625300	0.00001700
N	-3.07136200	0.02819600	0.00000700

(S113)

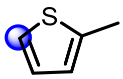

C	1.68052600	-0.22951300	0.00001300
C	1.38599300	1.10156500	0.00000000
C	-0.01993000	1.35639000	-0.00001100
C	-0.78543000	0.22314200	-0.00002400
S	0.23705400	-1.19457900	-0.00000700
H	2.64837900	-0.70663200	0.00005000
H	2.14020700	1.87762500	0.00001200
H	-0.45004700	2.35035900	0.00000200
C	-2.27960600	0.10560600	0.00001500
H	-2.64694100	-0.42659100	-0.88265900
H	-2.64689800	-0.42642400	0.88280700
H	-2.72688100	1.10178600	-0.00006700

(S114)

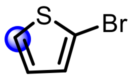

C	-2.58588200	-0.19899000	-0.00023400
C	-2.26443900	1.12723700	0.00043000
C	-0.85635400	1.36391600	-0.00036600
C	-0.14449100	0.19969600	0.00010500
S	-1.17048300	-1.20383100	0.00013500
H	-3.56396700	-0.65500600	-0.00065400
H	-3.00278900	1.91803500	0.00054200
H	-0.39861100	2.34303900	-0.00057000
Br	1.73714600	0.02011600	-0.00003100

(S115)

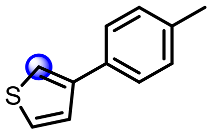

C	1.12716200	0.07839800	0.03115900
C	1.90046500	1.22120900	0.43101300
C	3.24625300	1.01760000	0.35657500
S	3.61786500	-0.57660900	-0.21040900
H	1.64198300	-1.93236400	-0.72069300
H	1.46156600	2.14435700	0.78571500
H	4.04239800	1.69865000	0.61516200
C	-0.34978000	0.03671800	0.02011300
C	-1.10244200	1.17450500	-0.30355000
C	-1.04834900	-1.14013400	0.32459500
C	-2.49319700	1.13309800	-0.32461000
H	-0.59458900	2.09588200	-0.56599900
C	-2.43866400	-1.17725300	0.29746600
H	-0.49675400	-2.03079700	0.60437300
C	-3.18991100	-0.04169500	-0.02366300
H	-3.04634800	2.02915600	-0.58859500
H	-2.94923400	-2.10404900	0.54019100
C	-4.69880900	-0.07466500	-0.01439100
H	-5.09187700	0.18299500	0.97548900
H	-5.11749600	0.63971400	-0.72727600
H	-5.07794900	-1.06766000	-0.26738900
C	1.93768400	-0.96613900	-0.34211300

(S116)

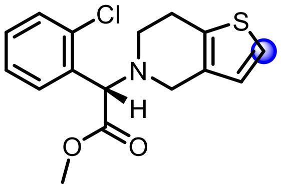

C	-3.26007400	-2.29486400	1.76322400
C	-3.47116200	-2.19417400	0.39327100
C	-2.71972500	-1.29042700	-0.35755800
C	-1.74866800	-0.47492900	0.23423800
C	-1.55879800	-0.59619100	1.61809600
C	-2.29868500	-1.49340700	2.37788800
H	-3.84483300	-2.99842600	2.34459300
H	-4.21156400	-2.80955400	-0.10142400
H	-0.80503700	0.02877900	2.08021200
H	-2.12671700	-1.56723600	3.44542200
Cl	-3.04127300	-1.22771300	-2.09089500
C	-1.53560700	1.93135000	-0.30804500
O	-2.23023200	2.49715100	-1.11495200
O	-1.25414500	2.42234600	0.91152700
C	-1.86224000	3.68831500	1.23545500
H	-2.94933500	3.60544100	1.20460800
H	-1.52271000	3.92311300	2.24171800
H	-1.54160100	4.45730600	0.53127200
C	1.06888000	-0.83855500	-0.49524300
C	1.29615600	1.54766900	-0.80600900
H	0.88433100	-1.10146800	-1.55522800
H	0.56097800	-1.59399000	0.10792000
C	2.67759600	1.64489100	-0.14595900
H	1.41339100	1.37104900	-1.89015800
H	0.77910800	2.50037200	-0.68687600
C	3.27234700	0.27230300	-0.06572500
H	3.31003300	2.32189400	-0.72952500
H	2.57342500	2.07937900	0.85468500
N	0.50070900	0.48247200	-0.17841600
C	2.54799500	-0.87470100	-0.21937700
C	3.34436400	-2.05809400	-0.10087400
H	2.95223400	-3.06349900	-0.19239300
C	4.65789500	-1.78791500	0.14536900
H	5.47273500	-2.48203800	0.28173500
S	4.95251000	-0.07745300	0.23674300
C	-0.91893400	0.54218200	-0.54885600
H	-1.06094400	0.36523300	-1.62230200

(S117)

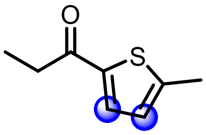

C	2.35853100	0.18198100	0.00002400
C	1.79614500	1.43560400	-0.00010300
C	0.37872300	1.41411600	-0.00015400
C	-0.14597800	0.14184900	-0.00007600
H	2.38631300	2.34356100	-0.00015100
H	-0.23160300	2.30770400	-0.00024700
C	3.81507500	-0.16952100	0.00010100
H	4.08850500	-0.75576800	0.88225900
H	4.08858100	-0.75583500	-0.88198900
H	4.41713100	0.74111500	0.00009200
C	-1.54740900	-0.30843700	-0.00008600
O	-1.82325000	-1.49671000	-0.00027800
C	-2.63077900	0.76615900	0.00014500
H	-2.47110000	1.40854800	0.87409400
H	-2.47120100	1.40881800	-0.87362200
C	-4.04725400	0.19625300	0.00014500
H	-4.78131900	1.00586800	0.00032900
H	-4.22046500	-0.42676400	-0.87915100
H	-4.22034800	-0.42705700	0.87925700
S	1.13369900	-1.05153300	0.00008600

(S118)

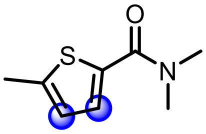

C	2.55033500	0.19811600	-0.01830700
C	1.94493500	1.32816900	-0.50458600
C	0.52861500	1.22580900	-0.57291400
C	0.05015500	0.01495400	-0.13817600
H	2.49959500	2.20723500	-0.80886000
H	-0.10577500	2.01213100	-0.95959500
C	4.01250700	-0.04145500	0.20807300
H	4.23357500	-0.23014600	1.26291000
H	4.37593200	-0.90057200	-0.36323600
H	4.58367900	0.83550900	-0.10315000
C	-1.31654900	-0.57307700	-0.15673300
O	-1.46154500	-1.77126000	-0.38265000
N	-2.39092000	0.26275300	0.03931700
C	-3.72674800	-0.28984500	-0.15438900
H	-4.16591300	-0.61537400	0.79680600
H	-4.37224400	0.47616900	-0.59299200
H	-3.66918300	-1.14825700	-0.81877200
C	-2.34588700	1.55170900	0.71820400
H	-2.64397800	2.36734500	0.04920000
H	-3.03952100	1.53984900	1.56646600
H	-1.34838200	1.75280300	1.09848500
S	1.36855200	-1.02826000	0.33573100

(S119)

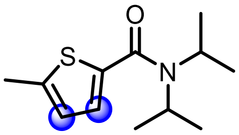

C	-3.32424100	0.04834000	0.39414300
C	-2.56842000	0.64670400	1.37025900
C	-1.17036200	0.46227600	1.19471800
C	-0.85662800	-0.27390800	0.07998500
H	-3.00515900	1.19549700	2.19559500
H	-0.41916000	0.82557200	1.88103600
C	-4.81658400	0.04121800	0.25742300
H	-5.13912100	0.49227400	-0.68571800
H	-5.22085800	-0.97475200	0.29133000
H	-5.26629900	0.60999800	1.07372400
C	0.45383500	-0.77992400	-0.38474100
O	0.52185400	-1.85080800	-0.97455100
N	1.57750000	-0.01730200	-0.04103100
C	2.86583900	-0.77664400	0.02443400
H	3.15319900	-1.07625100	-0.99341700
C	1.67343700	1.38062900	-0.59250600
H	2.66981000	1.42274700	-1.03605900
C	2.72492500	-2.03987500	0.88728400
H	2.38789000	-1.77214700	1.89365600
H	2.03071500	-2.75968900	0.46229200
H	3.70208000	-2.52135100	0.97774700
C	3.99668200	0.07166500	0.62734700
H	4.90617500	-0.53323500	0.65117900
H	4.23063800	0.97514700	0.06366000
H	3.76061000	0.35934200	1.65582700
C	1.60199100	2.46781700	0.48640300
H	2.26345400	2.24515500	1.32495500
H	1.90120900	3.43210500	0.06333800
H	0.58502200	2.57571100	0.86851200
C	0.71022300	1.68471300	-1.75020900
H	0.74831100	0.90711000	-2.51698500
H	-0.32262700	1.79459600	-1.41848300
H	1.01220200	2.62755200	-2.21456800
S	-2.30872600	-0.78099100	-0.75132800

(S120)

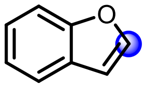

C	0.24937500	-0.65144200	-0.00015500
C	0.25730300	0.75375500	-0.00005400
C	-0.96660300	1.43683700	0.00010100
C	-2.13970000	0.69177900	0.00013500
C	-2.11281500	-0.71412500	0.00005000
C	-0.90938400	-1.41413200	-0.00008500
C	2.35117200	-0.02971400	0.00056200
C	1.65151500	1.12849700	-0.00039100
H	-0.99869400	2.52035100	0.00008900
H	-3.09644900	1.20124400	0.00018300
H	-3.04675500	-1.26392600	0.00006700
H	-0.87251500	-2.49630000	-0.00013100
H	3.40704500	-0.24420300	0.00090600
H	2.06910400	2.12295600	-0.00072900
O	1.53163600	-1.13110700	-0.00017000

(S121)

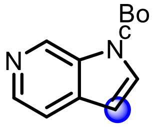

C	2.62191800	0.95764100	0.00023400
C	1.71929600	-0.12929900	0.00006800
C	4.36419800	-0.66419400	-0.00047200
C	3.99108600	0.67271500	0.00002500
C	1.83986600	2.16757600	0.00048300
C	0.53082800	1.80498800	-0.00011600
H	5.41579500	-0.93502600	-0.00060800
H	4.73761000	1.45833100	0.00023500
H	2.21194300	3.18022500	0.00070100
H	-0.36165200	2.40581000	-0.00039400
N	0.42065000	0.40886800	-0.00007400
C	-0.76765900	-0.33463600	0.00065300
O	-0.78345600	-1.54146300	0.00148000
O	-1.82229500	0.48783600	0.00019300
C	-3.21534000	-0.03891600	-0.00024800
N	3.49960800	-1.69544000	-0.00048700
C	2.19055100	-1.44349300	-0.00030800
H	1.51423900	-2.28765900	-0.00060400
C	-3.45901700	-0.85043600	-1.27340900
H	-2.86656200	-1.76396900	-1.28599400
H	-4.51680800	-1.12115300	-1.32805600
H	-3.21671100	-0.25606800	-2.15799800
C	-3.45991300	-0.85010400	1.27300000
H	-3.21778000	-0.25554100	2.15751700
H	-4.51782300	-1.12036600	1.32723500
H	-2.86771400	-1.76379600	1.28613200
C	-4.04555700	1.24355000	-0.00074100
H	-3.83102800	1.84312800	-0.88807000
H	-5.10932500	0.99517700	-0.00099400
H	-3.83152400	1.84357800	0.88641000

(S122)

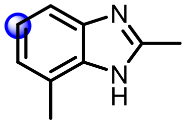

C	-0.86259000	-1.97328700	0.00001600
C	0.16519600	-1.02530700	0.00001300
C	-0.14854900	0.35056100	-0.00007700
C	-1.45723800	0.84423100	-0.00006900
C	-2.45761700	-0.12729200	-0.00005500
C	-2.16974400	-1.50515400	-0.00000800
H	-3.49438800	0.19317600	-0.00006300
C	2.05521900	0.00075000	0.00000300
N	1.54298300	-1.20320400	0.00006400
C	3.51184900	0.32355700	-0.00000700
H	3.79371800	0.90256700	-0.88524000
H	3.79341000	0.90384300	0.88447800
H	4.07658900	-0.60733900	0.00071400
H	-0.63412900	-3.03212300	0.00004200
N	1.08496000	0.98537900	0.00006400
H	1.25102500	1.97862700	-0.00069600
H	-2.99204800	-2.21162500	-0.00001500
C	-1.75236700	2.32168100	0.00009300
H	-1.33431600	2.81694700	0.88408600
H	-1.33216700	2.81762200	-0.88249600
H	-2.82824400	2.50464200	-0.00116200

(S124)

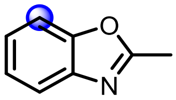

C	1.39901200	1.42985000	0.00021900
C	0.18966400	0.73601700	0.00044800
C	0.18726200	-0.66211300	0.00025700
C	1.33522500	-1.43499300	-0.00010100
C	2.53989900	-0.72976000	-0.00017700
C	2.56979300	0.67463600	-0.00012900
H	1.41848000	2.51267300	0.00004200
H	1.30068000	-2.51697100	0.00000100
H	3.47363100	-1.27975700	-0.00033400
H	3.52866400	1.17976300	-0.00036800
C	-1.84561800	0.10577900	-0.00009200
O	-1.12418900	-1.07024300	0.00016900
N	-1.13472200	1.18275600	-0.00033100
C	-3.32373200	-0.02485600	-0.00012900
H	-3.66208700	-0.57443200	-0.88272400
H	-3.66210200	-0.57392300	0.88281200
H	-3.76973800	0.96793900	-0.00023300

(S123)

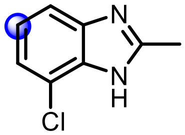

C	0.17699700	2.31373900	0.00011400
C	-0.61341500	1.16139200	-0.00001000
C	-0.00419500	-0.11446800	-0.00009600
C	1.37800500	-0.25756100	-0.00006100
C	2.16149400	0.88696400	0.00004400
C	1.55788900	2.15761000	0.00012700
H	3.23992000	0.79418900	0.00009400
C	-2.21985400	-0.27005600	-0.00015500
N	-1.99370800	1.01919300	-0.00033600
C	-3.56364200	-0.91633900	0.00023400
H	-3.70253500	-1.54631700	-0.88378100
H	-3.70327900	-1.54400100	0.88577400
H	-4.32702600	-0.14017300	-0.00110100
H	-0.28363100	3.29374000	0.00006900
N	-1.05150100	-1.01056100	-0.00003500
H	-0.97207100	-2.01530700	0.00039100
H	2.19719300	3.03255100	0.00018900
Cl	2.09577700	-1.86487000	-0.00001300

(S125)

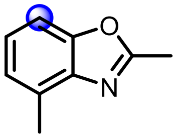

C	1.45714100	0.84465800	0.00002000
C	0.13155500	0.39026700	0.00004000
C	-0.15870500	-0.97536300	0.00003000
C	0.80306100	-1.97152300	-0.00001900
C	2.12250100	-1.52142800	-0.00003100
C	2.43611200	-0.15183500	-0.00000900
H	0.54802900	-3.02328200	-0.00002300
H	2.92875700	-2.24582700	-0.00004500
H	3.48013700	0.14300500	-0.00001800
C	-1.99168200	0.19319100	0.00002300
O	-1.52658900	-1.10435800	0.00004000
N	-1.07384700	1.10036800	0.00000300
C	-3.46545300	0.36827900	-0.00005600
H	-3.90976900	-0.10033500	-0.88248400
H	-3.90974000	-0.09951400	0.88283900
H	-3.69901600	1.43144600	-0.00049000
C	1.78364400	2.31283200	-0.00000900
H	1.35974700	2.80908600	-0.87777700
H	1.35928200	2.80920700	0.87746200
H	2.86315900	2.47402500	0.00026100

(S126)

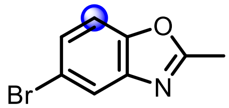

C	-0.22496500	-0.89167900	-0.00003500
C	1.11626200	-0.50963300	0.00001400
C	1.46519900	0.84359800	-0.00000600
C	0.54075000	1.87232800	-0.00000800
C	-0.80213600	1.49308000	0.00005200
C	-1.16024000	0.13716200	0.00000200
H	0.83397600	2.91421200	0.00008000
H	-1.57501300	2.24986900	0.00015000
C	3.24280400	-0.40379900	-0.00005700
O	2.83504200	0.91402700	-0.00007600
N	2.28642700	-1.27111200	0.00006600
C	4.70656300	-0.64418400	0.00002700
H	5.17023400	-0.19512700	-0.88246400
H	5.16989200	-0.19599300	0.88315600
H	4.89217900	-1.71662100	-0.00049700
H	-0.51567200	-1.93329200	-0.00001900
Br	-3.02761000	-0.31196300	-0.00000500

(S127)

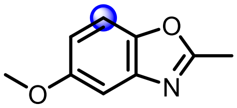

C	0.93059100	-0.81932700	0.00000700
C	-0.42386000	-0.47348100	0.00005900
C	-0.81839500	0.86209800	0.00003000
C	0.07955300	1.91776000	-0.00006700
C	1.42673600	1.57746900	0.00004000
C	1.84973600	0.23054700	0.00003600
H	-0.24342500	2.95095300	-0.00010800
H	2.18985700	2.34559400	0.00010500
C	-2.55444300	-0.44014900	-0.00001100
O	-2.19466300	0.88705900	-0.00001500
N	-1.56753900	-1.27520500	0.00003600
C	-4.00898900	-0.73549500	-0.00002200
H	-4.49032600	-0.30591500	0.88284400
H	-4.49039200	-0.30559800	-0.88271300
H	-4.15383800	-1.81431100	-0.00023300
H	1.22621500	-1.85865600	-0.00003100
O	3.20680000	0.05964600	0.00000500
C	3.72145100	-1.26404600	-0.00004500
H	4.80546500	-1.16204300	-0.00003700
H	3.40890000	-1.81478700	0.89409200
H	3.40894500	-1.81470300	-0.89425400

(S128)

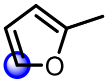

C	0.63994000	0.10648200	-0.00033300
C	-0.21916000	1.16271400	-0.00011400
C	-1.54719000	0.61637200	0.00000900
C	-1.39936400	-0.73203500	0.00011900
O	-0.07363900	-1.06202000	0.00003700
H	0.05745300	2.20535000	-0.00025800
H	-2.47959400	1.15836900	0.00102800
H	-2.08999100	-1.55798800	-0.00078100
C	2.12143400	-0.01579300	0.00012400
H	2.47562300	-0.55510500	0.88416700
H	2.47621100	-0.55706300	-0.88246400
H	2.57544700	0.97616300	-0.00081600

(S129)

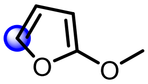

C	-0.19449900	-0.16604000	-0.00012600
C	0.33850900	1.09026500	-0.00004400
C	1.76483700	0.89083600	0.00005600
C	1.97456600	-0.44634000	0.00003600
O	0.76691200	-1.11178200	0.00000400
H	-0.19347600	2.02615700	-0.00043200
H	2.52518800	1.65580300	0.00010900
H	2.84867200	-1.07286200	0.00023500
O	-1.43378300	-0.66746800	-0.00010500
C	-2.48033900	0.30210600	0.00012800
H	-3.41110900	-0.26081700	0.00009700
H	-2.42631100	0.93021200	0.89505600
H	-2.42644900	0.93053900	-0.89455300

(S130)

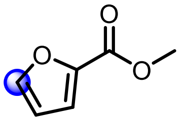

C	-2.62131900	0.46786000	-0.00035600
C	-2.53473100	-0.89328000	0.00031900
C	-1.14228100	-1.19607700	-0.00001200
C	-0.48741100	0.00512900	0.00017300
O	-1.39109700	1.02807700	-0.00027200
H	-3.45053400	1.15553300	-0.00061600
H	-3.35925500	-1.58805600	0.00065400
H	-0.67516900	-2.16708700	-0.00002600
C	0.93141700	0.37550100	0.00018300
O	1.36079500	1.50402100	0.00039300
O	1.70792000	-0.73327800	-0.00000900
C	3.12720400	-0.49747700	-0.00028400
H	3.42182100	0.06084100	0.88942700
H	3.58383200	-1.48470000	-0.00159900
H	3.42108200	0.06296900	-0.88886900

(S131)

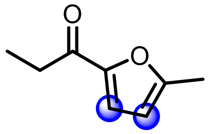

C	2.27425700	-0.01079800	0.00005700
C	1.99948500	1.33035100	-0.00008000
C	0.58148800	1.44911400	-0.00016500
C	0.07777600	0.17342700	-0.00008600
O	1.11892100	-0.71851300	-0.00000300
H	2.72514400	2.12828900	-0.00010000
H	0.00637800	2.36151200	-0.00025700
C	3.53909400	-0.79029700	0.00019000
H	3.60220200	-1.43351400	0.88280200
H	3.60232100	-1.43362400	-0.88233500
H	4.39504400	-0.11466100	0.00020500
C	-1.28677400	-0.37088700	-0.00009100
O	-1.49499900	-1.57015800	-0.00027100
C	-2.42100900	0.65124900	0.00014200
H	-2.29435200	1.30095300	0.87429400
H	-2.29450100	1.30118700	-0.87385800
C	-3.80693800	0.01084800	0.00017900
H	-4.58121200	0.78203700	0.00035700
H	-3.94841400	-0.62030500	-0.87902300
H	-3.94825600	-0.62055000	0.87923100

(S132)

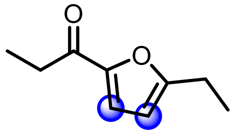

C	1.85926700	0.42072000	-0.22157900
C	1.43287100	1.69683400	0.03450400
C	0.01528200	1.63411400	0.13833500
C	-0.33792900	0.32352200	-0.05974600
O	0.79430700	-0.41657600	-0.27942700
H	2.06046200	2.56847000	0.13343200
H	-0.65847000	2.45324900	0.33420500
C	3.20577600	-0.18245100	-0.43016400
H	3.22683300	-0.66715900	-1.41294900
H	3.93472900	0.63154600	-0.46316300
C	-1.62692500	-0.38069800	-0.07853900
O	-1.69485200	-1.58102800	-0.26977800
C	-2.86877400	0.47759900	0.15055300
H	-2.74215600	1.02059800	1.09446400
H	-2.89821300	1.24523000	-0.63210500
C	-4.16412800	-0.33046600	0.16164000
H	-5.02112900	0.32860000	0.32101500
H	-4.30538200	-0.85928900	-0.78254700
H	-4.15308200	-1.07997100	0.95518800
C	3.59631700	-1.20477100	0.65161200
H	2.88039800	-2.02824500	0.68524100
H	4.58490200	-1.62052200	0.44169300
H	3.62492100	-0.73809400	1.63947300

(S133)

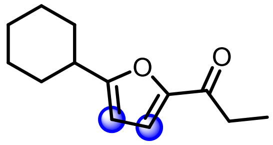

C	-0.38795000	0.38065300	-0.00001400
C	0.06651400	1.67499500	-0.00012200
C	1.48576600	1.60370200	-0.00012300
C	1.81681500	0.27179800	-0.00001100
O	0.66622300	-0.47232600	0.00008100
H	-0.54089000	2.56612800	-0.00019400
H	2.17630700	2.43231400	-0.00019700
C	3.09576100	-0.44852500	0.00004300
O	3.14442000	-1.66527800	0.00014800
C	4.35598300	0.41434100	-0.00005000
H	4.31690600	1.07522000	-0.87409700
H	4.31692800	1.07537700	0.87387900
C	5.64470300	-0.40427300	0.00000800
H	6.51454900	0.25732900	-0.00006600
H	5.70088700	-1.04872700	0.87915700
H	5.70086200	-1.04888900	-0.87902400
C	-1.75280900	-0.23203300	0.00003900
C	-2.55274200	0.13879900	1.27055300
C	-2.55269900	0.13849800	-1.27059100
H	-1.59999400	-1.31758100	0.00017000
C	-3.94654200	-0.50470700	1.26699900
H	-2.65348900	1.23013300	1.32242000
H	-1.99253600	-0.16705200	2.15925800
C	-3.94649900	-0.50500700	-1.26693600
H	-2.65344500	1.22982000	-1.32271800
H	-1.99246200	-0.16756100	-2.15920500
C	-4.73443800	-0.14638200	-0.00002400
H	-4.49922600	-0.19460300	2.15965000
H	-3.83960400	-1.59519300	1.33135100
H	-4.49915200	-0.19511400	-2.15967900
H	-3.83955900	-1.59550800	-1.33102600
H	-5.70288400	-0.65669500	0.00002100
H	-4.94951300	0.93028300	-0.00015500

(S134)

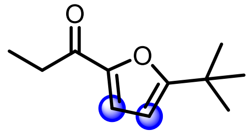

C	-1.24904100	0.41242100	0.00001200
C	-0.81966300	1.71443300	0.00008700
C	0.60260000	1.67117900	0.00014800
C	0.95853100	0.34710900	0.00012400
O	-0.17658300	-0.41935000	0.00006200
H	-1.44202000	2.59405700	0.00009000
H	1.27631900	2.51342300	0.00019700
C	-2.60647400	-0.24053800	-0.00007300
C	2.25091400	-0.35050200	0.00016200
O	2.31911800	-1.56622500	0.00037300
C	3.49604700	0.53322300	-0.00001500
H	3.44542400	1.19359500	-0.87385300
H	3.44578700	1.19333700	0.87404600
C	4.79846500	-0.26344700	-0.00039600
H	5.65685900	0.41288000	-0.00044000
H	4.86584700	-0.90701500	0.87860500
H	4.86552900	-0.90670900	-0.87964800
C	-2.74698900	-1.12235200	-1.26278100
H	-1.97568900	-1.89432100	-1.28962500
H	-3.72490100	-1.61232100	-1.26823500
H	-2.66130800	-0.52180300	-2.17250200
C	-2.74702400	-1.12260000	1.26245500
H	-2.66138200	-0.52223600	2.17230100
H	-3.72492700	-1.61258700	1.26777300
H	-1.97570800	-1.89456000	1.28917600
C	-3.69983800	0.84165400	0.00002400
H	-3.63486900	1.47815100	-0.88669800
H	-4.68546500	0.36928300	-0.00007300
H	-3.63493700	1.47793000	0.88691100

(S135)

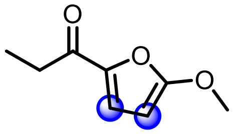

C	-1.81008400	-0.06321800	0.00124200
C	-1.57978000	1.29220400	0.00722600
C	-0.16228900	1.41896400	0.00275800
C	0.36694900	0.15488900	-0.00668900
O	-0.67065800	-0.76152600	-0.00710900
H	-2.31354800	2.07989700	0.01516300
H	0.39847700	2.34041600	0.00540600
C	1.73255300	-0.36952900	-0.01983300
O	1.96116200	-1.56616100	-0.01369800
C	2.85247900	0.67023600	-0.05241600
H	2.75779300	1.23609100	-0.98729100
H	2.68279500	1.39352600	0.75320800
C	4.24459400	0.05311800	0.06114700
H	5.00974700	0.83233800	0.01925900
H	4.35796300	-0.49094600	1.00092000
H	4.42694100	-0.65566200	-0.74817700
O	-2.90362500	-0.81712500	0.00267400
C	-4.14746900	-0.11065700	0.01314100
H	-4.24248200	0.51526600	-0.87885400
H	-4.92157900	-0.87414600	0.01320400
H	-4.23286100	0.50566400	0.91276100

(S136)

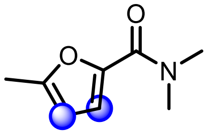

C	2.47369000	0.01849600	0.04409500
C	2.15380400	1.24507400	-0.46367400
C	0.73035500	1.28036300	-0.56766500
C	0.27315500	0.07226900	-0.11850500
O	1.34067700	-0.70226700	0.24848400
H	2.84884500	2.02427100	-0.73431900
H	0.13065200	2.08856400	-0.95427300
C	3.75814300	-0.63705400	0.40335400
H	3.79130600	-0.88299200	1.46908200
H	3.89249300	-1.56802200	-0.15501900
H	4.59218800	0.02801800	0.17617700
C	-1.04059100	-0.62183400	-0.11100700
O	-1.09500900	-1.82901700	-0.30925500
N	-2.16916200	0.14660000	0.06073200
C	-3.46609100	-0.49171700	-0.12729300
H	-3.92133600	-0.75684900	0.83487800
H	-4.13663000	0.19490900	-0.65239800
H	-3.33708900	-1.39991900	-0.71058500
C	-2.20849800	1.47390900	0.65929300
H	-2.53130900	2.23210800	-0.06420100
H	-2.92158500	1.47629500	1.49108900
H	-1.23255800	1.75064200	1.04903000

(S137)

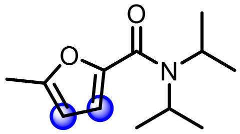

C	3.26471200	-0.13790800	-0.21215900
C	2.81513500	0.42048300	-1.37536100
C	1.39033500	0.34530800	-1.33665400
C	1.06248000	-0.25319000	-0.15236500
O	2.20890900	-0.55944600	0.53290800
H	3.42891700	0.82541000	-2.16464100
H	0.69619400	0.65630700	-2.09936200
C	4.61873100	-0.35796600	0.35957600
H	4.73428200	0.16468400	1.31390700
H	4.79932400	-1.42150000	0.54152200
H	5.37898400	0.01027900	-0.33032800
C	-0.20567300	-0.76220600	0.41022200
O	-0.21992300	-1.80551700	1.04625400
N	-1.35342600	-0.03586700	0.07958100
C	-2.63319800	-0.81059100	0.13470100
H	-2.84104300	-1.07590900	1.18060500
C	-1.42442600	1.40427500	0.51001500
H	-2.40024800	1.49335900	0.98905900
C	-2.52848200	-2.10397000	-0.68808200
H	-2.29447400	-1.86773700	-1.73088600
H	-1.77360400	-2.78311100	-0.30105600
H	-3.49192900	-2.61995000	-0.66799500
C	-3.82075300	-0.00593300	-0.41559900
H	-4.71265500	-0.63506700	-0.36860600
H	-4.04550800	0.90373400	0.14192300
H	-3.65786300	0.26268100	-1.46309900
C	-1.39472300	2.39022100	-0.66342400
H	-2.10754800	2.10850200	-1.43993500
H	-1.65171000	3.39510500	-0.31348300
H	-0.39951100	2.43812700	-1.10962400
C	-0.41093100	1.79981300	1.59600500
H	-0.40698800	1.08263200	2.42037800
H	0.60487300	1.88845800	1.20930400
H	-0.69663900	2.77475700	2.00070700

(S138)

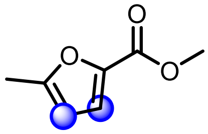

C	-2.22794700	-0.00262000	0.00000000
C	-1.94217600	1.33579600	0.00000000
C	-0.52258300	1.44752500	0.00000000
C	-0.03629200	0.16972500	0.00000000
O	-1.07579900	-0.71994800	0.00000000
H	-2.66264100	2.13845200	0.00000100
H	0.07244500	2.34607700	0.00000100
C	-3.49738200	-0.77451700	0.00000000
H	-3.56595700	-1.41699200	-0.88278600
H	-3.56596000	-1.41698800	0.88278800
H	-4.34836300	-0.09261500	-0.00000300
C	1.31513800	-0.39099100	0.00000000
O	1.58785200	-1.56836400	0.00000100
O	2.23999400	0.60017100	-0.00000100
C	3.61143500	0.16830200	0.00000000
H	4.20210500	1.08183700	-0.00000500
H	3.82541100	-0.42697200	0.88892800
H	3.82540900	-0.42698100	-0.88892200

(S139)

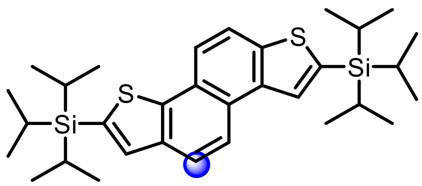

C	-2.18678000	1.24188500	0.00341900
C	-1.81024000	-0.10602800	-0.05503000
C	0.14154300	1.88057800	0.04503800
C	-1.17909400	2.24107100	0.05420200
C	-3.61027900	1.40974700	0.00369900
C	-4.33231500	0.24766800	-0.05698400
H	0.90815100	2.64605200	0.08509800
H	-1.46255000	3.28687100	0.10058700
H	-4.07010900	2.38864600	0.05179900
C	2.27983600	-1.24580700	-0.09724000
C	1.89909600	0.10085300	-0.03239000
C	-0.04939800	-1.88612500	-0.13650100
C	1.27244000	-2.24504100	-0.14982600
C	3.70577700	-1.40962100	-0.09686000
C	4.41911300	-0.24469800	-0.03084400
H	-0.81442300	-2.65316500	-0.17716500
H	1.55616400	-3.29058300	-0.20017600
H	4.17307400	-2.38499500	-0.14477800
C	-0.44938300	-0.51933500	-0.07123600
C	0.53879600	0.51259600	-0.01776100
S	-3.22647200	-1.13030700	-0.11064600
S	3.31113400	1.13164000	0.02982200
Si	-6.22517600	0.07386800	0.01809200
Si	6.29174100	0.04544300	-0.03150200
C	-6.89650100	1.87756800	0.03478300
H	-6.23995800	2.40527800	0.73910400
C	-6.90096500	-0.85925900	-1.52127100
H	-6.85030800	-0.11114600	-2.32367500
C	-6.70249900	-0.78388900	1.66451300
H	-7.79874900	-0.71779200	1.70402200
C	6.78073500	0.90578200	-1.68626500
H	7.83958500	0.64709900	-1.83302700
C	6.70080900	1.22450100	1.42880600
H	6.27306900	2.18572100	1.11251700
C	7.26510900	-1.60343300	0.11903900
H	8.30198800	-1.25059800	0.22348500
C	-6.77502700	2.58449900	-1.33011700
H	-7.01730400	3.64938000	-1.23713300
H	-5.77121200	2.51018500	-1.75644500
H	-7.47286200	2.16033400	-2.05859100
C	-8.33198900	2.02541000	0.57772300
H	-8.45032700	1.59425000	1.57460600
H	-8.60550000	3.08460300	0.64553500
H	-9.06634300	1.54886200	-0.07816800
C	-8.38446500	-1.25403200	-1.35898800
H	-9.02376300	-0.40355400	-1.10947600
H	-8.76782800	-1.68650700	-2.29017800
H	-8.51823800	-2.00685000	-0.57592400
C	-6.07978100	-2.07085000	-2.00232500
H	-6.52651100	-2.49037000	-2.91144100
H	-5.04845800	-1.80289900	-2.23824700
H	-6.05292400	-2.87360100	-1.25971700
C	-6.14437300	-0.02695800	2.88578800
H	-6.49083800	-0.48896100	3.81730000
H	-5.05056700	-0.04700200	2.89652600
H	-6.45369000	1.02162400	2.90698500
C	-6.33383400	-2.27747400	1.75087300
H	-6.68576300	-2.70223500	2.69822300
H	-6.78122000	-2.86433900	0.94516400
H	-5.25105400	-2.42971700	1.71439600
C	5.99581000	0.37112500	-2.90202100
H	4.93785400	0.63912900	-2.83265000
H	6.38530900	0.80647500	-3.82953700
H	6.04906400	-0.71472300	-3.00068400
C	6.68564900	2.44437900	-1.65515700
H	7.01177100	2.86380800	-2.61386100
H	5.65604300	2.78086700	-1.49484100
H	7.30711900	2.89121800	-0.87590600
C	8.21774300	1.43716300	1.60549200
H	8.41543900	2.22496400	2.34121400
H	8.70773800	0.52875100	1.97008300
H	8.71495700	1.72825400	0.67535000
C	6.04503600	0.87330800	2.77789000
H	4.96607600	0.72984100	2.68833100
H	6.46546100	-0.03816700	3.21109300
H	6.21308900	1.67846700	3.50256500
C	7.23535800	-2.50097400	-1.13441400
H	7.60816900	-1.98527700	-2.02192200
H	7.86730300	-3.38342200	-0.98168900
H	6.22739400	-2.86123800	-1.36074200
C	6.94818900	-2.43329400	1.37913300
H	7.60377900	-3.31031800	1.43090900
H	7.09320900	-1.86471600	2.29949900
H	5.91751100	-2.79809300	1.38149500

(S140)

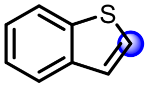

C	0.06162200	-0.55364800	0.00003700
C	-0.11102500	0.85138900	-0.00005600
C	-1.41701600	1.37197000	0.00002600
C	-2.49983300	0.50738700	0.00019600
C	-2.30748700	-0.88492700	0.00028700
C	-1.02927600	-1.42642000	0.00020800
C	2.21562600	0.71166800	-0.00026300
C	1.14880900	1.54819700	-0.00023200
H	-1.56985800	2.44576500	-0.00004400
H	-3.50764500	0.90648600	0.00026000
H	-3.16686100	-1.54560100	0.00042000
H	-0.88396800	-2.50041100	0.00027900
H	3.26295200	0.97448800	-0.00038000
H	1.23709800	2.62695000	-0.00032600
S	1.76623500	-0.97883500	-0.00008900

(S141)

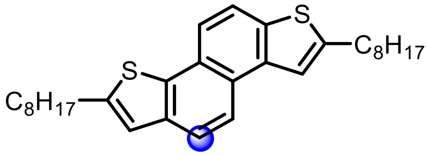

C	1.93554200	-1.64828600	-0.29968200
C	1.77707300	-0.41346900	0.34199500
C	-0.37510700	-1.55983200	-1.00045300
C	0.82710800	-2.21678600	-0.97880800
C	3.26532100	-2.17300900	-0.15854600
C	4.10087400	-1.37757200	0.56116300
H	-1.21782500	-2.00080800	-1.52089700
H	0.93793700	-3.17262300	-1.47912400
H	3.57937700	-3.12002700	-0.58104700
C	-1.93570000	1.64498500	0.28936700
C	-1.77729900	0.40999700	-0.35201500
C	0.37496100	1.55653800	0.99014400
C	-0.82720200	2.21358400	0.96831800
C	-3.26548500	2.16969400	0.14822200
C	-4.10116100	1.37397000	-0.57103800
H	1.21768800	1.99755200	1.51054100
H	-0.93796400	3.16953900	1.46842200
H	-3.57947200	3.11684300	0.57048700
C	0.54834200	0.29956400	0.34207500
C	-0.54852100	-0.30295000	-0.35222100
S	3.27404600	0.08098900	1.11021600
S	-3.27437900	-0.08468900	-1.11983800
C	5.54618600	-1.60374800	0.89517500
H	5.79385900	-2.63360900	0.61670400
H	5.69198600	-1.53497200	1.98015800
C	6.52040900	-0.63674500	0.19436800
H	6.26671200	0.39424800	0.46592200
H	6.37693100	-0.71190200	-0.88948200
C	7.98604900	-0.91167700	0.54630700
H	8.11750000	-0.83642800	1.63355200
H	8.23610100	-1.94723400	0.28098800
C	8.96750000	0.03888500	-0.14881500
H	8.70881200	1.07464100	0.10624000
H	8.84395800	-0.04552400	-1.23606900
C	10.43386700	-0.21945500	0.21566300
H	10.55642700	-0.13162300	1.30299600
H	10.69263700	-1.25628800	-0.03589800
C	11.41586100	0.72903600	-0.48101300
H	11.15301000	1.76647400	-0.23580100
H	11.29951800	0.63643600	-1.56875200
C	12.88192100	0.48033500	-0.10866800
H	12.99878900	0.57526900	0.97781500
H	13.14532800	-0.55620700	-0.35252400
C	13.85527000	1.43166300	-0.81084600
H	13.63958000	2.47500400	-0.55930600
H	14.89067700	1.22853000	-0.52334600
H	13.78795200	1.33427200	-1.89907700
C	-5.54688000	1.59922400	-0.90393100
H	-5.79335300	2.63108200	-0.63185200
H	-5.69485400	1.52329400	-1.98812100
C	-6.52034900	0.63760000	-0.19463000
H	-6.26669800	-0.39545200	-0.45825100
H	-6.37582100	0.72138500	0.88845000
C	-7.98637100	0.90952300	-0.54730700
H	-8.11920700	0.82401200	-1.63362600
H	-8.23590200	1.94759900	-0.29149200
C	-8.96710300	-0.03418800	0.15808900
H	-8.70845200	-1.07243100	-0.08668900
H	-8.84271300	0.06122100	1.24433700
C	-10.43380100	0.22021100	-0.20782200
H	-10.69229100	1.25974700	0.03263500
H	-10.55739200	0.12050600	-1.29401400
C	-11.41516000	-0.72054600	0.50014100
H	-11.15249500	-1.76064300	0.26624800
H	-11.29790100	-0.61586400	1.58668700
C	-12.88153500	-0.47601100	0.12627700
H	-13.14466900	0.56324200	0.35860800
H	-12.99934200	-0.58323000	-0.95895900
C	-13.85431100	-1.41928100	0.84002300
H	-14.88999800	-1.21906300	0.55148800
H	-13.63917900	-2.46539400	0.59977300
H	-13.78570700	-1.30989100	1.92703500

(142)

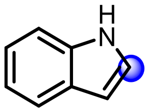

C	0.24740900	-0.67178500	0.00001900
C	0.24858800	0.74964400	-0.00005100
C	-0.98181500	1.42718100	0.00003600
C	-2.15695000	0.69110600	0.00018100
C	-2.13361200	-0.71758200	0.00024200
C	-0.93447300	-1.41692800	0.00016300
C	2.38854800	0.03134900	-0.00034100
C	1.62396100	1.16591100	-0.00019700
H	-1.01125400	2.51137300	-0.00000600
H	-3.11185500	1.20436900	0.00025500
H	-3.06892400	-1.26565000	0.00036300
H	-0.92010400	-2.50146200	0.00022300
H	1.88187600	-2.03524600	0.00002600
H	3.46121100	-0.08261100	-0.00051200
H	1.99726300	2.17802300	-0.00027100
N	1.56597900	-1.08031000	-0.00005600

(S143)

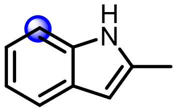

C	0.20970300	-0.65888500	0.00013000
C	0.23401600	0.76142200	0.00009100
C	1.47561500	1.41577900	0.00010600
C	2.63862100	0.65788000	-0.00009500
C	2.58982400	-0.74876800	-0.00012000
C	1.37582300	-1.42540800	0.00007800
C	-1.93187400	0.08361100	-0.00022200
C	-1.13486100	1.19921200	-0.00004600
H	1.52506100	2.49933500	0.00017000
H	3.60266600	1.15390300	-0.00023900
H	3.51462400	-1.31441600	-0.00022600
H	1.34061900	-2.50961100	0.00018000
H	-1.45053900	-1.99093200	-0.00099300
H	-1.48982700	2.21858500	-0.00008800
N	-1.11871600	-1.04066300	0.00022200
C	-3.41920300	-0.04380800	-0.00001700
H	-3.77921100	-0.58298300	-0.88328900
H	-3.77896400	-0.58026000	0.88500500
H	-3.87940300	0.94480200	-0.00150300

(S144)

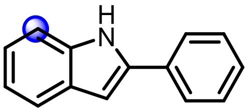

C	1.83675800	-0.63913500	-0.11737300
C	1.89258600	0.75656900	0.14381900
C	3.14907000	1.37356300	0.26620900
C	4.29351700	0.60582400	0.11193500
C	4.21359100	-0.77420000	-0.16068300
C	2.98679600	-1.41461300	-0.27761200
C	-0.28840800	0.13487700	0.01980300
C	0.53915500	1.21761100	0.22302700
H	3.22152700	2.43547200	0.47435300
H	5.26851000	1.07138400	0.20073700
H	5.12628900	-1.34702400	-0.27863900
H	2.92841400	-2.47807900	-0.48255200
H	0.15669200	-1.88350900	-0.47969500
H	0.21316500	2.22122100	0.44704600
C	-1.75036500	0.05320600	0.00924200
C	-2.52236900	1.17509000	-0.33550100
C	-2.41966800	-1.13533100	0.34439200
C	-3.91103800	1.11053500	-0.33821200
H	-2.02508000	2.09484300	-0.62010800
C	-3.81035300	-1.20034800	0.33080400
H	-1.85335900	-2.00779700	0.65157500
C	-4.56333700	-0.07829500	-0.00910800
H	-4.48731900	1.98795500	-0.60988600
H	-4.30610400	-2.12691500	0.59808300
H	-5.64605100	-0.12835900	-0.01737500
N	0.50395800	-0.99304600	-0.16400000

(S145)

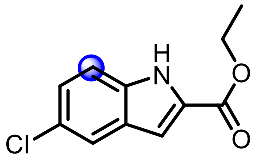

C	-0.95296000	1.08019700	0.00025700
C	-0.94122600	-0.34434000	-0.00033000
C	-2.16136100	-1.04566900	-0.00041800
C	-3.32889100	-0.31062400	0.00009500
C	-3.33696200	1.09906400	0.00067300
C	-2.14793000	1.80801000	0.00077200
C	1.18570300	0.39932100	-0.00038100
C	0.42771600	-0.75074500	-0.00081400
H	-2.18754400	-2.12781600	-0.00088300
H	-4.28427800	1.62214000	0.00107000
H	-2.15268200	2.89173000	0.00127400
H	0.69582000	2.44921600	0.00091100
H	0.81702700	-1.75579900	-0.00142800
N	0.35154300	1.50121700	0.00027800
C	2.63439800	0.62659800	-0.00056500
O	3.13077000	1.73585900	-0.00006900
O	3.33606700	-0.51871300	-0.00126800
C	4.78151100	-0.38958000	-0.00114400
C	5.36477400	-1.78697100	0.00241000
H	5.07806900	0.18291400	0.88051900
H	5.07847300	0.17842200	-0.88558300
H	6.45635900	-1.72709600	0.00235300
H	5.05215500	-2.34061200	0.89048900
H	5.05232700	-2.34513900	-0.88289300
Cl	-4.87940200	-1.15207100	-0.00002400

(S146)

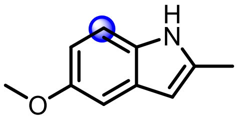

C	0.73497400	0.76851500	-0.00006500
C	0.54755600	-0.64010500	-0.00028500
C	-0.75233200	-1.15717700	-0.00019900
C	-1.83112200	-0.27757600	-0.00036200
C	-1.62946300	1.11672100	-0.00040000
C	-0.34064600	1.64825600	-0.00013500
C	2.77722900	-0.21165800	-0.00007500
C	1.85822400	-1.23034100	-0.00033300
H	-0.94263800	-2.22382900	-0.00033100
H	-2.47207000	1.79410400	-0.00063800
H	-0.19632800	2.72322500	-0.00016000
H	2.54137100	1.90315600	-0.00501800
H	2.09474100	-2.28335200	-0.00003800
N	2.10212600	0.99801000	0.00115000
C	4.26950800	-0.25944800	0.00039500
H	4.69009200	0.23483800	-0.88235200
H	4.68923000	0.23067500	0.88585300
H	4.61070700	-1.29509600	-0.00196300
O	-3.07505500	-0.86469200	-0.00031800
C	-4.22161800	-0.03222200	0.00078600
H	-5.07869300	-0.70470800	0.00167200
H	-4.26158400	0.60139300	0.89467500
H	-4.26313300	0.60126500	-0.89317000

(S147)

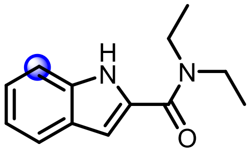

C	-2.24063100	-0.72170100	-0.08760000
C	-2.05838400	0.67698400	0.09945300
C	-3.18777800	1.49634800	0.27910100
C	-4.44416500	0.91531100	0.26864400
C	-4.60283900	-0.47510600	0.08114900
C	-3.51122600	-1.30929900	-0.10048400
C	-0.02580800	-0.29570200	-0.14862600
C	-0.64973400	0.92045000	0.05816700
H	-3.07467100	2.56532900	0.42350000
H	-5.32412700	1.53309700	0.40636200
H	-5.60091300	-0.89827900	0.07970100
H	-3.63781200	-2.37629700	-0.24467600
H	-0.74787200	-2.25348200	-0.28149100
H	-0.17519000	1.87598300	0.20220900
N	-0.99719200	-1.27610700	-0.23739200
C	1.37237700	-0.80849900	-0.18900400
O	1.53760700	-2.02159600	-0.02365100
N	2.43413800	0.02916400	-0.38670500
C	2.34538700	1.41457200	-0.85450400
C	2.67491200	2.46069900	0.21420000
H	1.34985000	1.57939500	-1.26137700
H	3.04081700	1.52086200	-1.69440900
H	2.57210400	3.46724900	-0.20208800
H	2.00713900	2.37315100	1.07444600
H	3.70117400	2.35297600	0.57385400
C	3.77476200	-0.57713300	-0.30293800
C	4.25060400	-0.82131000	1.13059900
H	4.46068900	0.09753500	-0.82014000
H	3.75669700	-1.52308200	-0.84831300
H	5.25291900	-1.25959400	1.11864100
H	4.29214000	0.10928700	1.70199000
H	3.58271200	-1.51644300	1.64074300

(S148)

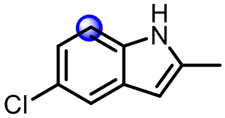

C	-0.72021800	0.84034700	-0.10689600
C	-0.42965500	-0.49970600	-0.09915700
C	0.87361300	-0.98360200	-0.08185700
C	1.91641900	-0.04067400	0.01821000
C	1.61990700	1.34537500	0.10220200
C	0.28302100	1.79512100	0.04045900
C	-2.75287700	-0.28541900	-0.03787000
C	-1.74496300	-1.25687100	-0.08278200
H	1.07707400	-2.03271300	-0.13543400
H	2.41472600	2.05359700	0.20983600
H	0.04844800	2.83749900	0.09809700
H	-2.54202900	1.76913700	0.32116200
H	-1.87515200	-2.31892000	-0.08453000
N	-2.17589100	1.06576800	-0.28811300
C	-4.24087900	-0.57148300	0.23711600
H	-4.42526300	-0.52048200	1.28987500
H	-4.84286700	0.15605500	-0.26607200
H	-4.48838000	-1.54878300	-0.12139300
Cl	3.59049900	-0.58319400	0.04521700

(S149)

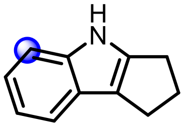

C	-0.95278700	-0.84958500	-0.00130100
C	-0.56271100	0.52544600	0.00885100
C	-1.56430500	1.50879200	0.00883400
C	-2.89744300	1.12021600	-0.00729100
C	-3.25781800	-0.23938600	-0.02413500
C	-2.29135400	-1.23936200	-0.02239300
C	1.28583900	-0.77009700	0.00954200
C	0.87265500	0.52822800	0.01236700
H	-1.29937600	2.56047500	0.02005200
H	-3.67624500	1.87451600	-0.00749900
H	-4.30660400	-0.51335700	-0.03767300
H	-2.57370400	-2.28683900	-0.03361900
C	2.04246500	1.46888600	0.09453900
C	3.25226800	0.51460400	-0.18040000
H	2.11734900	1.93333900	1.08601800
H	2.00716400	2.28542300	-0.63306300
H	4.12801900	0.77110100	0.41903100
H	3.54130500	0.60081400	-1.23058700
C	2.76779000	-0.94809800	0.08884700
H	3.07581000	-1.30283700	1.08114000
H	3.17422700	-1.65413000	-0.64254200
N	0.20209300	-1.62270700	0.01206700
H	0.22981600	-2.62741700	-0.03048900

(S150)

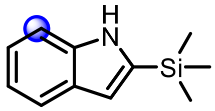

C	-1.60658100	-0.65539600	0.00010600
C	-1.63614900	0.76439800	0.00005800
C	-2.88024300	1.41818400	-0.00002600
C	-4.04001200	0.65887200	-0.00007100
C	-3.98693000	-0.74992400	-0.00001900
C	-2.77421500	-1.42473500	0.00008100
C	0.54637800	0.09013900	0.00010200
C	-0.27052300	1.20202700	0.00007300
H	-2.93095700	2.50169900	-0.00007100
H	-5.00578600	1.15140600	-0.00015500
H	-4.91124200	-1.31653300	-0.00006500
H	-2.73794800	-2.50886500	0.00011400
H	0.03837300	-1.98725000	0.00012300
H	0.07100600	2.22620700	0.00001600
N	-0.28157600	-1.03321400	0.00011500
Si	2.42719400	0.00429700	-0.00003800
C	3.01720400	-0.91983300	-1.53971000
H	4.11021500	-0.97742900	-1.56525300
H	2.63610600	-1.94553900	-1.57118800
H	2.68535700	-0.41656200	-2.45222200
C	3.01766400	-0.91924500	1.53981500
H	4.11069100	-0.97682600	1.56493900
H	2.68620600	-0.41552400	2.45222300
H	2.63656300	-1.94492300	1.57196300
C	3.09359600	1.76634300	-0.00033300
H	2.76623000	2.31942300	-0.88510000
H	2.76643400	2.31955500	0.88441900
H	4.18793200	1.75851500	-0.00047300

(S151)

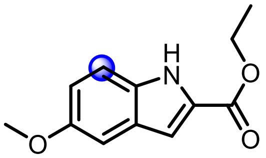

C	0.93204200	0.98586300	-0.00015600
C	0.87903800	-0.43584900	-0.00028800
C	2.07113900	-1.17100900	-0.00027400
C	3.27782200	-0.49100400	-0.00022700
C	3.31431000	0.92057600	0.00003300
C	2.14630400	1.66649300	0.00006300
C	-1.22288900	0.37374600	-0.00023300
C	-0.50119000	-0.79767400	-0.00018300
H	2.07161400	-2.25152900	-0.00027000
H	4.25994400	1.43890600	0.00033100
H	2.18905800	2.74677900	0.00023100
H	-0.67474800	2.40304400	-0.00044900
H	-0.91993000	-1.78774200	0.00023500
N	-0.36031900	1.44864600	-0.00027500
C	-2.66219100	0.63910700	0.00007000
O	-3.13035900	1.75968500	0.00000300
O	-3.39650600	-0.48693300	0.00047700
C	-4.83374200	-0.30836700	0.00044500
C	-5.46566700	-1.68264100	-0.00029200
H	-5.11189800	0.27033400	-0.87972500
H	-5.11191200	0.26932600	0.88128800
H	-6.55153100	-1.58445600	-0.00018000
H	-5.17342000	-2.24746400	-0.88487500
H	-5.17333300	-2.24848700	0.88360800
O	4.40471300	-1.27297100	0.00015600
C	5.67401900	-0.64125700	0.00030900
H	6.40618800	-1.44395900	0.00038700
H	5.81781700	-0.02571100	-0.89131700
H	5.81765100	-0.02571700	0.89196400

(S152)

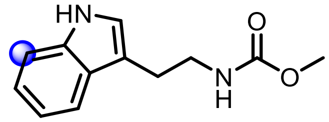

C	-3.07372200	-0.71679700	0.20024500
C	-2.07796900	0.25072700	-0.09586500
C	-2.44925800	1.60236800	-0.18068600
C	-3.77626700	1.95118100	0.02510200
C	-4.74738300	0.97365500	0.31700200
C	-4.41094700	-0.37019700	0.40855400
C	-1.10220800	-1.77787200	-0.05004100
C	-0.82554300	-0.44972700	-0.25176900
H	-1.71069300	2.36494400	-0.40290900
H	-4.07472800	2.99139900	-0.03797500
H	-5.77654100	1.27628900	0.47327100
H	-5.16123700	-1.12038300	0.63367900
H	-2.89921700	-2.82774000	0.40083800
H	-0.44879100	-2.63568300	-0.07432600
N	-2.45014700	-1.94563100	0.22322500
C	0.49660900	0.19047600	-0.56589400
H	0.37760000	0.86578000	-1.42251600
H	0.81694200	0.81990500	0.27285800
C	1.62029800	-0.80621300	-0.88010800
H	1.33258700	-1.43994100	-1.72380400
H	1.79809800	-1.45959800	-0.02556300
N	2.88387400	-0.16483800	-1.22689400
H	3.02761500	0.15017300	-2.17352400
C	3.84329600	0.26554300	-0.36003400
O	4.83705500	0.88465400	-0.68186300
O	3.55880800	-0.09961800	0.91768100
C	4.53766500	0.28500200	1.89634700
H	4.15233800	-0.07840000	2.84703700
H	5.50310600	-0.17204200	1.67511200
H	4.65250100	1.36942300	1.91983200

(S153)

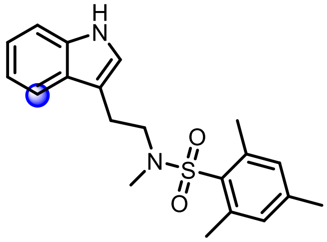

C	-5.54337400	-0.21268100	0.38102500
C	-4.45372200	0.46974000	-0.22165000
C	-4.70279000	1.66566200	-0.91427100
C	-6.00343800	2.14264500	-0.99176200
C	-7.06880700	1.44802000	-0.38682600
C	-6.85423100	0.26406600	0.30608000
C	-3.66025200	-1.38051600	0.78721200
C	-3.26058900	-0.29469500	0.05106800
H	-3.89110700	2.21215500	-1.38235100
H	-6.20706500	3.06467400	-1.52430700
H	-8.07464100	1.84503900	-0.46314000
H	-7.67680300	-0.26805500	0.77152800
H	-5.56076000	-2.02536800	1.49536800
H	-3.08167000	-2.19468300	1.19436500
N	-5.02958800	-1.33741400	0.98936700
C	-1.87128600	0.07669400	-0.38581100
H	-1.89844000	0.34524600	-1.44788300
H	-1.54047500	0.97689700	0.14587300
C	-0.84048100	-1.03141900	-0.14965400
H	-1.12278400	-1.93854100	-0.70131800
H	-0.81225300	-1.27894800	0.91112900
N	0.51954700	-0.59687200	-0.53724900
C	0.76409700	-0.58716400	-1.98539600
H	0.64915500	-1.58102700	-2.43478700
H	0.05948400	0.10261400	-2.45095100
H	1.76908400	-0.21586600	-2.18962500
S	1.77057900	-1.36397400	0.34622100
O	1.31916300	-1.42112200	1.73778900
O	2.15591800	-2.61721400	-0.30803300
C	3.12532600	-0.16268400	0.20008900
C	2.96813300	1.08950000	0.84466700
C	4.30728300	-0.48728000	-0.50217100
C	4.01469500	2.00581500	0.75394600
C	5.31603900	0.48484900	-0.54552100
C	5.19660200	1.72690200	0.06744200
H	3.89841100	2.96842000	1.24187800
H	6.22837800	0.24809100	-1.08271200
C	4.60167700	-1.79430700	-1.20867100
H	4.60821200	-2.63653800	-0.51615100
H	3.85918600	-2.03519300	-1.96983800
H	5.58061300	-1.72738600	-1.68606300
C	1.74534100	1.49898800	1.63196100
H	1.84891300	2.53408400	1.96014900
H	0.83912500	1.41255500	1.03039200
H	1.60830400	0.86223500	2.50667100
C	6.31174000	2.74055800	0.00944700
H	5.94192100	3.71670800	-0.31671600
H	6.76396100	2.87880200	0.99681700
H	7.09985000	2.42820400	-0.67798500

(S154)

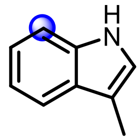

C	-0.40482700	0.90709600	-0.00000200
C	0.17332300	-0.39075300	-0.00000200
C	-0.67434100	-1.51029900	-0.00000100
C	-2.04835900	-1.31675000	0.00000100
C	-2.59905400	-0.02035200	0.00000100
C	-1.78739100	1.10639900	-0.00000100
C	1.83636500	1.12432900	0.00000100
C	1.60611900	-0.22574700	-0.00000100
H	-0.26169800	-2.51342200	-0.00000100
H	-2.71235800	-2.17374000	0.00000400
H	-3.67645600	0.10012600	0.00000200
H	-2.21498600	2.10326100	-0.00000200
H	0.53803800	2.81445200	0.00001100
H	2.77346200	1.66013900	0.00000200
N	0.63376100	1.81348000	-0.00000100
C	2.63201800	-1.31702400	0.00000100
H	2.53516600	-1.96034800	0.88119200
H	2.53518300	-1.96033700	-0.88120000
H	3.64421000	-0.90588000	0.00001300

(S155)

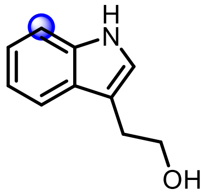

C	-1.50377000	0.85367100	0.00003900
C	-0.63845800	-0.27177500	0.00010300
C	-1.19978800	-1.55906600	0.00011900
C	-2.58079500	-1.69252100	-0.00004800
C	-3.41988200	-0.56122800	-0.00020700
C	-2.89475300	0.72408100	-0.00015000
C	0.62476000	1.59086400	0.00010600
C	0.71744600	0.22289700	0.00015400
H	-0.56403900	-2.43778300	0.00025100
H	-3.02522700	-2.68127800	-0.00005600
H	-4.49552300	-0.69639700	-0.00035500
H	-3.54386100	1.59311600	-0.00022000
H	-1.03352500	2.92845500	-0.00136700
H	1.40105400	2.33989000	0.00019400
N	-0.70644200	1.97760800	0.00032700
C	1.95234100	-0.63184500	0.00029500
H	1.94932500	-1.29155000	0.87600700
H	1.94899900	-1.29223900	-0.87489800
C	3.25993800	0.14528400	-0.00027700
H	3.31854300	0.78691900	0.88919800
H	3.31810300	0.78632900	-0.89019900
O	4.32557500	-0.80787400	-0.00021500
H	5.16441000	-0.33790600	0.00007100

(S156)

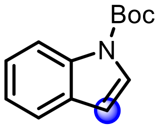

C	2.61172000	0.96296700	0.00005500
C	1.71348700	-0.13117700	0.00007600
C	2.15865700	-1.45263400	-0.00002800
C	3.53499000	-1.66205300	-0.00010800
C	4.44214500	-0.59072800	-0.00017700
C	3.99098900	0.72274300	-0.00008700
C	1.81963800	2.16916300	0.00016200
C	0.51322700	1.80587700	0.00009700
H	1.45973600	-2.27538600	-0.00004300
H	3.91154400	-2.67862100	-0.00020000
H	5.50706300	-0.79382400	-0.00028300
H	4.69216800	1.54993200	-0.00008200
H	2.18868900	3.18329400	0.00019000
H	-0.38207300	2.40163200	0.00004100
N	0.41127100	0.40433300	-0.00007900
C	-0.77271700	-0.33744100	0.00024500
O	-0.79811300	-1.54584400	0.00047800
O	-1.83097800	0.48556800	0.00024800
C	-3.22058500	-0.04110500	-0.00010400
C	-3.46783700	-0.85244200	1.27301200
H	-4.52601400	-1.12219100	1.32654600
H	-2.87537800	-1.76591100	1.28645100
H	-3.22565000	-0.25782300	2.15751900
C	-3.46717200	-0.85277500	-1.27312600
H	-2.87469100	-1.76623800	-1.28608000
H	-4.52531100	-1.12261000	-1.32703400
H	-3.22465600	-0.25838700	-2.15769300
C	-4.05289000	1.24050100	-0.00048800
H	-3.83832700	1.84036600	-0.88762000
H	-5.11660600	0.99152000	-0.00058600
H	-3.83857800	1.84074500	0.88644800

(S157)

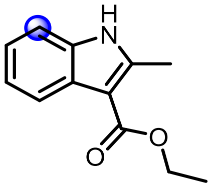

C	2.09855700	0.81879400	0.00000400
C	1.29419100	-0.34375200	-0.00001400
C	1.91847200	-1.59998000	-0.00002400
C	3.30641400	-1.65316200	-0.00000600
C	4.08648900	-0.48257700	0.00002700
C	3.49207500	0.77340900	0.00001800
C	-0.07006800	1.49114200	0.00000200
C	-0.08342200	0.10178900	0.00000700
H	1.31766800	-2.49944500	-0.00003500
H	3.80232700	-2.61726400	-0.00000200
H	5.16766300	-0.56045300	0.00004400
H	4.08990800	1.67829500	0.00002200
H	1.52233900	2.87292500	0.00001500
N	1.23753400	1.90663900	-0.00000700
C	-1.17418800	2.49830700	-0.00001400
H	-1.81353500	2.37842700	0.87666900
H	-0.77123600	3.51425900	-0.00025200
H	-1.81379400	2.37812900	-0.87647200
C	-1.23726500	-0.79048400	-0.00002700
O	-1.17454600	-2.00337200	-0.00001000
O	-2.42575300	-0.12316700	-0.00001700
C	-3.61469200	-0.94602700	0.00004000
C	-4.81528900	-0.02085100	0.00000500
H	-3.59688300	-1.59104500	0.88167000
H	-3.59690100	-1.59114700	-0.88151200
H	-5.73531300	-0.61169400	0.00005300
H	-4.82010500	0.61765000	0.88665600
H	-4.82013200	0.61755800	-0.88671000

(S158)

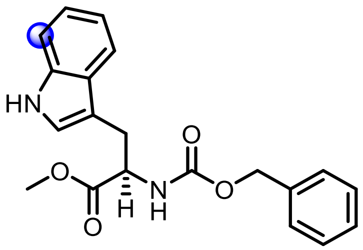

C	-3.89775800	-1.59324200	0.47131000
C	-3.05483100	-0.91852700	-0.45239900
C	-3.61140300	-0.45208200	-1.65561100
C	-4.96163800	-0.65614100	-1.90002500
C	-5.77862800	-1.32086200	-0.96445000
C	-5.25830300	-1.79856400	0.23027500
C	-1.81427800	-1.54242900	1.32265000
C	-1.72723600	-0.89618400	0.11491500
H	-2.99412900	0.05972000	-2.38562500
H	-5.39908200	-0.30252800	-2.82692800
H	-6.83063000	-1.46461000	-1.18279100
H	-5.88745300	-2.31400900	0.94779700
H	-3.43137600	-2.44943700	2.36218300
H	-1.04552100	-1.73841900	2.05372700
N	-3.11240700	-1.96092500	1.54265400
C	-0.50129500	-0.27322900	-0.48652400
H	0.36524300	-0.92876000	-0.37529500
H	-0.64548900	-0.12492200	-1.55916300
C	-0.08766300	1.09706200	0.13284300
H	0.75285600	1.47794600	-0.45252900
C	-1.21566700	2.11876100	0.04204800
O	-1.78812800	2.59847800	0.99004900
O	-1.49681100	2.41325100	-1.23637700
C	-2.59050000	3.33167300	-1.44837800
H	-3.51849500	2.90053200	-1.07199500
H	-2.39377000	4.27705100	-0.94232400
H	-2.64175800	3.47448600	-2.52520700
N	0.29979200	0.99965800	1.52871900
H	-0.35022200	1.36035400	2.21339800
C	1.51848700	0.62593900	2.00298600
O	1.82692200	0.59544700	3.17704100
O	2.35621200	0.27553600	0.98766400
C	3.69017800	-0.13152300	1.39480900
H	3.60519000	-1.00489800	2.04393200
H	4.14054300	0.67733000	1.97238700
C	4.47824200	-0.43535900	0.15120900
C	5.19691900	0.57334500	-0.49752200
C	4.49229500	-1.72640400	-0.38473000
C	5.91238100	0.29937600	-1.66100300
H	5.19710000	1.57774400	-0.08663500
C	5.20662900	-2.00469500	-1.54818700
H	3.94236500	-2.51804400	0.11393000
C	5.91738900	-0.99101700	-2.18906300
H	6.46845200	1.08990800	-2.15238500
H	5.21200900	-3.01105100	-1.95164300
H	6.47697900	-1.20661300	-3.09236000

(S159)

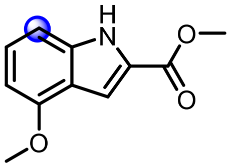

C	-0.42264100	1.21588200	0.00001300
C	-0.92608100	-0.11197000	0.00002400
C	-2.32533300	-0.31643400	0.00010000
C	-3.16215600	0.79071600	-0.00000900
C	-2.62347000	2.09809400	-0.00000600
C	-1.26273000	2.33974200	0.00008200
C	1.32105000	-0.20885000	-0.00003100
C	0.19044900	-0.99526000	0.00003700
H	-4.23681900	0.67187000	-0.00019100
H	-3.31115200	2.93625200	-0.00006600
H	-0.86724300	3.34819900	0.00015800
H	1.59996200	1.89265000	0.00004500
H	0.17909800	-2.07233900	0.00009600
C	2.72041700	-0.63199400	0.00001800
O	3.10879500	-1.77711200	0.00007900
O	3.55725000	0.44303700	0.00001200
C	4.96362400	0.14008900	-0.00008200
H	5.22956800	-0.43435500	-0.88841100
H	5.46932400	1.10336200	-0.00145000
H	5.23015600	-0.43205400	0.88958500
N	0.94568900	1.12741200	-0.00010300
O	-2.73283300	-1.61360600	0.00002300
C	-4.12881200	-1.88585500	-0.00008100
H	-4.61227300	-1.47915400	-0.89456100
H	-4.21966700	-2.97066800	-0.00009600
H	-4.61237400	-1.47915300	0.89431900

(S160)

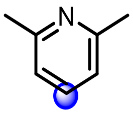

C	1.19844000	-1.13220400	0.00004900
C	1.15735000	0.26589400	0.00003400
C	-1.15734600	0.26589800	-0.00002900
C	-1.19843900	-1.13220500	-0.00004800
C	-0.00000200	-1.83723000	-0.00000100
H	2.14980600	-1.65109700	0.00008700
H	-2.14980700	-1.65109500	-0.00008900
H	-0.00000100	-2.92193500	-0.00000200
N	0.00000000	0.94118100	0.00000500
C	2.41593600	1.09471900	-0.00002400
H	2.44008100	1.74461400	0.87886100
H	3.31165500	0.47086700	0.00054100
H	2.44060100	1.74367700	-0.87959900
C	-2.41593600	1.09472000	0.00001900
H	-2.43994100	1.74477100	-0.87875200
H	-3.31165700	0.47086800	-0.00079200
H	-2.44074400	1.74351000	0.87971400

(S161)

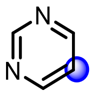

C	-1.30985400	-0.00022500	0.00001500
C	0.62221800	-1.18324600	0.00000200
C	1.35349300	0.00015500	0.00000100
C	0.62191100	1.18337300	-0.00001900
H	-2.39579600	-0.00040700	-0.00003400
H	1.11593900	-2.15107400	0.00002000
H	2.43641600	0.00053200	0.00002100
H	1.11553000	2.15127100	0.00000700
N	-0.71439600	1.19494300	0.00001000
N	-0.71398900	-1.19503800	-0.00001100

(S162)

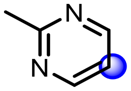

C	0.84374400	-0.00225700	-0.01163400
C	-1.10972700	-1.17870200	0.00168800
C	-1.84334000	0.00188800	0.00714300
C	-1.10551600	1.18078600	0.00182100
H	-1.60194900	-2.14770000	0.00354000
H	-2.92586100	0.00392900	0.01259300
H	-1.59503500	2.15122300	0.00334300
N	0.22829700	1.19044200	-0.00820000
N	0.22474500	-1.19246600	-0.00813100
C	2.34559800	-0.00163700	0.00414700
H	2.73267200	0.84929800	-0.55700200
H	2.70265200	0.09270100	1.03545200
H	2.73167700	-0.93575000	-0.40259600

(S163)

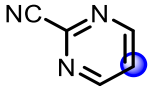

C	-0.53767100	-0.00008000	0.00002600
C	1.38886700	-1.18486800	-0.00000300
C	2.11891800	-0.00001200	-0.00001000
C	1.38893900	1.18483300	-0.00001000
H	1.87946500	-2.15343200	-0.00000200
H	3.20139200	0.00010800	-0.00001300
H	1.87958700	2.15338100	-0.00001300
N	0.05433600	1.19779400	0.00001300
N	0.05443900	-1.19767500	0.00000500
C	-1.98505500	-0.00006400	0.00000100
N	-3.13798000	0.00003600	-0.00001800

(S164)

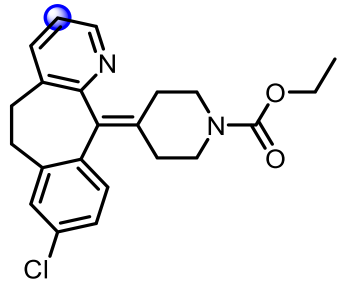

C	1.70719600	-0.55488100	-0.20810000
C	1.70046200	-1.35920000	-1.35161800
H	1.05710400	-1.09401900	-2.18256500
C	2.50808500	-2.49102900	-1.44271200
H	2.49950500	-3.10949500	-2.33105200
C	3.33079300	-2.81315200	-0.37047900
C	3.36126300	-2.02741900	0.77700600
H	4.00782500	-2.30294600	1.60187500
C	2.55181500	-0.89363100	0.86270000
C	2.71377000	2.23819600	0.69087200
C	3.36576800	3.46143600	0.49983800
H	4.21920200	3.70678500	1.12553300
C	2.93642300	4.35823700	-0.46630700
H	3.43810900	5.30566200	-0.62307000
C	1.83199200	3.99732200	-1.23261200
H	1.44898700	4.65944700	-2.00472000
N	1.19182600	2.84168100	-1.08044500
C	1.60714900	1.96342000	-0.14469200
C	0.85592700	0.66898400	-0.11073600
C	-0.49766400	0.58742200	-0.02588500
C	-1.41218300	1.78477700	0.15173100
H	-1.61899800	1.90602500	1.22311700
H	-0.94561500	2.69768800	-0.20749400
C	-2.73695000	1.59176200	-0.58480800
H	-3.42905400	2.40540800	-0.38042500
H	-2.56366600	1.55644500	-1.66680500
C	-2.55448600	-0.76851900	0.02523400
H	-3.06737300	-1.70982100	0.15072100
C	-1.20623300	-0.68282400	0.03392000
H	-0.64899100	-1.60430000	0.13331300
Cl	4.35646900	-4.24290000	-0.46222300
N	-3.38117800	0.33520200	-0.17081400
O	-5.20679500	-0.94561600	0.21819300
C	-4.76016400	0.28042400	-0.13613900
O	-5.48334900	1.22149000	-0.38804900
C	2.57019600	0.00232600	2.07438200
H	1.54414900	0.17973400	2.41232300
H	3.09638000	-0.48904300	2.89720100
C	3.26355100	1.34563300	1.79685900
H	4.31765100	1.14640400	1.56834600
H	3.26515200	1.92954400	2.72488100
C	-6.64666300	-1.09675800	0.28083400
H	-7.06678800	-0.85713300	-0.69817700
H	-7.04184700	-0.37735200	1.00104300
C	-6.93554300	-2.52645300	0.68951100
H	-6.50182400	-2.75090800	1.66660700
H	-8.01659400	-2.67702800	0.75192300
H	-6.53368500	-3.23339600	-0.03991400

(S165)

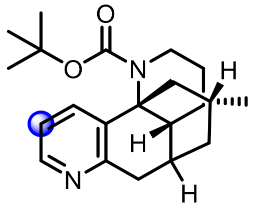

N	0.58486300	-1.11025700	0.50192600
N	-3.62315900	-0.42733700	-1.77485700
C	-0.50169900	-0.07642800	0.38544100
C	-1.30844500	-0.07196500	1.72647500
C	-2.50644900	0.88943300	1.59092900
C	0.01679400	1.37428100	0.16537900
C	-2.00579600	2.32376400	1.36615900
C	-1.09804200	2.43677600	0.13651000
C	-1.74344200	-1.45863400	2.22305800
C	-1.42308200	-0.43444900	-0.79150500
C	-3.46902900	0.41067100	0.48514100
C	-2.80210200	-0.16922400	-0.74429100
C	-0.56686900	-2.42949700	2.26239700
C	0.10246000	-2.45119500	0.89383700
C	-0.49298800	3.84002200	0.00885300
C	-0.90741500	-0.96509000	-1.97569100
C	-1.75400200	-1.22366200	-3.04553900
C	-3.10932900	-0.94029900	-2.89158000
H	-0.62348000	0.33991100	2.47868200
H	-3.05677600	0.87232600	2.53865900
H	0.57414900	1.42602200	-0.76667100
H	0.71921500	1.61588500	0.96931000
H	-2.85616100	3.00851000	1.26886700
H	-1.44641100	2.64491700	2.25516200
H	-1.70932600	2.26451800	-0.75680200
H	-2.51865400	-1.87363700	1.56971100
H	-2.19814000	-1.34700500	3.21359500
H	-4.11996000	1.22961400	0.16353300
H	-4.14692100	-0.35496900	0.87823300
H	0.16374000	-2.12584600	3.02039300
H	-0.90326000	-3.43898200	2.52052000
H	0.95540900	-3.12196300	0.87793900
H	-0.61714700	-2.79393100	0.14119200
H	0.12242600	3.92908900	-0.89124100
H	-1.27731200	4.60083000	-0.04668100
H	0.13936900	4.07609100	0.87162700
H	0.15260200	-1.18049500	-2.04915200
H	-3.81125800	-1.13309100	-3.69855700
H	-1.37827200	-1.63733400	-3.97399800
C	1.95101200	-1.02548300	0.31183600
O	2.71602900	-1.93039000	0.60381700
O	2.35914100	0.13055200	-0.25453700
C	3.79787800	0.41912600	-0.44561600
C	4.41318100	-0.57534900	-1.43377900
H	5.43400300	-0.26338400	-1.67231600
H	4.43933000	-1.58062000	-1.01808300
H	3.83958500	-0.58986800	-2.36450300
C	4.52346800	0.42643700	0.90247500
H	5.54844400	0.77972200	0.75893500
H	4.02674100	1.10793800	1.59841900
H	4.55355900	-0.56910200	1.34135600
C	3.77412900	1.82532700	-1.04882300
H	4.79469500	2.16089900	-1.24808600
H	3.21989400	1.83372700	-1.99021900
H	3.30524200	2.53472200	-0.36352800

(S166)

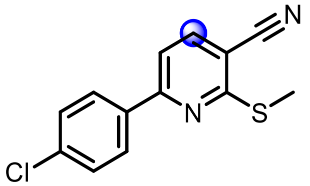

C	-0.79845900	-1.98851300	-0.13862900
C	-2.17634300	-2.14092900	-0.14751600
C	-3.00036700	-1.01719600	-0.06355200
C	-2.37219600	0.25351100	0.01698500
C	-0.26058000	-0.69849400	-0.03783800
H	-0.15853100	-2.85462400	-0.23248100
H	-2.62450400	-3.12406100	-0.22689700
N	-1.05326000	0.38749100	0.02703000
S	-3.39404800	1.69837200	0.11534600
C	-2.14413400	3.02012700	0.20793100
H	-1.55834200	3.06417600	-0.70833200
H	-2.71477200	3.94158500	0.32770100
H	-1.48949300	2.87116600	1.06422000
C	-4.41822000	-1.15866600	-0.06744800
N	-5.56695300	-1.29029100	-0.07186200
C	1.20227400	-0.45487100	-0.01693300
C	1.71048900	0.79857800	-0.38726500
C	2.11009200	-1.45123900	0.36944600
C	3.07721400	1.04806600	-0.39032300
H	1.01861500	1.57717600	-0.67929200
C	3.48008200	-1.21306300	0.37836700
H	1.75410500	-2.42121300	0.69426900
C	3.95427500	0.03699300	-0.00718300
H	3.46125700	2.01551700	-0.68759500
H	4.17218500	-1.98535200	0.68814800
Cl	5.68241000	0.34443900	-0.00398300

(S167)

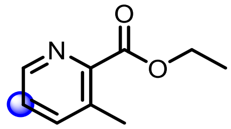

C	0.69411600	-0.36414700	0.10407900
C	1.16806600	0.95338400	0.23705000
C	2.51846500	1.15521800	-0.05887500
C	3.31142200	0.09323700	-0.47718500
C	2.72820300	-1.16678300	-0.57699800
N	1.44911100	-1.39642100	-0.28181200
H	2.94412400	2.14831500	0.04239800
H	4.35924200	0.23525700	-0.71426800
H	3.30803000	-2.02669300	-0.89909700
C	0.29961400	2.10169400	0.68850900
H	-0.39567000	2.40144800	-0.09916300
H	-0.29864900	1.83725100	1.56386600
H	0.91520700	2.96443200	0.95027600
C	-0.73080000	-0.74326300	0.43647400
O	-1.04162400	-1.60451600	1.21823000
O	-1.61555800	-0.00215800	-0.26212300
C	-3.01963400	-0.29899100	-0.04093500
H	-3.18766100	-1.35455600	-0.26427200
H	-3.24584600	-0.14178700	1.01633400
C	-3.82939700	0.61120000	-0.94088500
H	-4.89496900	0.40907100	-0.80297800
H	-3.64825100	1.66238800	-0.70517200
H	-3.58221000	0.44391200	-1.99150100

(S168)

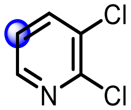

C	0.00000000	0.70090900	0.00000000
C	-0.00951200	-0.70286400	0.00000000
C	1.21420900	-1.36266100	0.00000000
C	2.38727500	-0.61447900	0.00000000
H	3.16744700	1.40166100	0.00000000
H	1.23672400	-2.44519100	0.00000000
H	3.35561700	-1.09908800	0.00000000
N	1.11008500	1.40918100	0.00000000
Cl	-1.50143700	1.60119300	0.00000000
Cl	-1.48607400	-1.62923500	0.00000000
C	2.28424700	0.77160700	0.00000000

(S169)

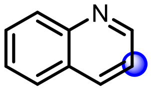

C	2.41536600	-0.66443300	0.00000000
C	-0.02629700	-0.67638600	0.00000000
C	-0.02245900	0.74988400	0.00000000
C	1.23298400	1.43836200	0.00000000
C	2.43078200	0.70734500	0.00000000
H	-1.20404300	-2.47361300	0.00000000
H	3.30204000	-1.28106900	0.00000000
H	1.19820700	-2.34180200	0.00000100
C	-1.22506800	-1.38799300	0.00000000
C	-1.27200900	1.40112100	0.00000000
H	1.24458200	2.52085000	0.00000000
H	3.38712100	1.21548600	-0.00000100
C	-2.46556400	0.68596700	0.00000000
C	-2.44556900	-0.70858800	0.00000000
H	-1.28821200	2.48580700	0.00000000
H	-3.41163800	1.21502000	0.00000000
H	-3.37220200	-1.27031400	0.00000000
N	1.20159200	-1.33457700	0.00000000

(S170)

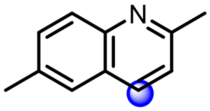

C	-2.10237500	-1.30373600	0.00000400
C	-0.77316300	-1.64817600	-0.00002600
C	0.23093200	-0.64681700	0.00000200
C	-0.17447100	0.71962100	-0.00000100
C	-1.55621200	1.03876900	0.00002800
C	-2.52122400	0.05539300	0.00002600
H	-2.85729600	-2.08404500	0.00002800
H	-0.45555000	-2.68420800	-0.00004400
C	0.85424200	1.69445800	-0.00001000
H	-1.84771800	2.08524000	0.00006500
C	2.16377300	1.29319800	-0.00006100
C	2.47473900	-0.09746800	-0.00018000
H	0.59419300	2.74845100	0.00003000
H	2.96831800	2.02027600	-0.00002300
N	1.54132600	-1.02760700	-0.00008600
C	-3.99300100	0.38569600	0.00003100
H	-4.49120300	-0.03233700	-0.88062100
H	-4.49123400	-0.03234500	0.88065700
H	-4.15975000	1.46449300	0.00002100
C	3.91407900	-0.54516900	0.00014100
H	4.44265400	-0.16604200	-0.88051200
H	4.44177500	-0.16749500	0.88195900
H	3.96261800	-1.63334300	-0.00068100

(S171)

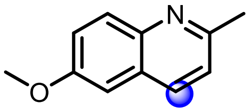

C	1.75376700	-1.17258700	-0.00000300
C	0.43352100	-1.56505400	0.00000000
C	-0.61235700	-0.61450700	0.00000000
C	-0.26736100	0.77143200	-0.00000200
C	1.08865700	1.15808100	-0.00000600
C	2.08851900	0.20474800	-0.00000700
H	2.53051900	-1.92560700	-0.00000200
H	0.16536600	-2.61482700	0.00000300
C	-1.34484100	1.69751400	0.00000400
H	1.36101500	2.20751600	-0.00000700
C	-2.63132700	1.23329500	0.00000700
C	-2.87949700	-0.17264300	0.00000200
H	-1.13551800	2.76247700	0.00000700
H	-3.46927600	1.92178100	0.00001200
N	-1.90487500	-1.05642000	0.00000000
C	-4.29734100	-0.68436400	-0.00000300
H	-4.84249700	-0.33059500	0.88122700
H	-4.84249500	-0.33058200	-0.88122900
H	-4.29678100	-1.77369600	-0.00001100
O	3.37293200	0.67066700	-0.00001200
C	4.44577100	-0.26185100	0.00001300
H	4.42972000	-0.89357400	-0.89463500
H	5.35584600	0.33586600	0.00001800
H	4.42969600	-0.89355300	0.89467500

(S172)

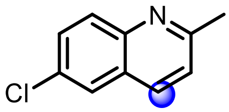

C	-1.69485300	1.40552000	0.00000100
C	-0.35279700	1.70057500	0.00000200
C	0.61885200	0.66825600	-0.00000400
C	0.17749000	-0.68793100	-0.00000400
C	-1.20950800	-0.97683900	-0.00000200
C	-2.11250900	0.05625500	-0.00000200
H	-2.43786100	2.19276300	0.00000500
H	-0.00477400	2.72638900	0.00000400
C	1.17918100	-1.69210800	0.00000100
H	-1.54793200	-2.00602500	-0.00000100
C	2.49738300	-1.32472800	-0.00000100
C	2.84643800	0.05815200	-0.00002700
H	0.89055900	-2.73823900	0.00001200
H	3.28227400	-2.07273000	0.00001600
N	1.93877600	1.01269700	-0.00000500
C	4.29688900	0.46523900	0.00001600
H	4.81355400	0.07112600	0.88105800
H	4.81351600	0.07138500	-0.88116500
H	4.37665800	1.55143300	0.00016700
Cl	-3.83746000	-0.28937300	0.00000300

(S173)

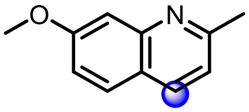

C	1.98276200	-0.33912100	-0.00019100
C	0.79417800	-1.04471500	-0.00006400
C	-0.44153000	-0.36741700	-0.00000700
C	-0.45637200	1.06219100	-0.00003300
C	0.77424000	1.75540300	-0.00022700
C	1.97369800	1.07886300	-0.00030600
H	0.80324700	-2.12704500	0.00002100
C	-1.72154100	1.69973200	0.00018700
H	0.76918100	2.84066100	-0.00033300
C	-2.85949600	0.93913300	0.00027800
C	-2.75105300	-0.48383200	0.00014900
H	-1.77436800	2.78414400	0.00028800
H	-3.84042600	1.40076200	0.00047400
N	-1.59241300	-1.10806400	-0.00001400
H	2.90019500	1.63680000	-0.00066700
C	-4.00055000	-1.32698800	-0.00014000
H	-4.61522400	-1.11495100	0.88075800
H	-4.61451000	-1.11537800	-0.88164700
H	-3.73434800	-2.38315400	0.00019100
O	3.12697700	-1.08282100	-0.00007900
C	4.38491000	-0.42091300	0.00038200
H	5.13347900	-1.21150200	0.00011300
H	4.51433000	0.19768900	-0.89415700
H	4.51403800	0.19697200	0.89551800

(S174)

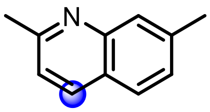

C	-0.04401000	-0.38253100	-0.00000100
C	-0.01903400	1.04295200	0.00000000
C	-1.26687200	1.71258600	0.00000000
C	-2.42438100	0.98004500	0.00000000
H	1.13283300	-2.17515000	0.00000000
C	1.18371100	-1.09201200	-0.00000100
C	1.23388300	1.70613100	0.00000000
H	-1.29298700	2.79789500	0.00000000
H	-3.39274500	1.46791600	0.00000100
C	2.40348800	0.98720500	0.00000000
C	2.39523500	-0.43503000	0.00000000
H	1.25609400	2.79138600	0.00000000
H	3.35565900	1.50834400	0.00000000
N	-1.20905800	-1.09462100	-0.00000100
C	-3.62410100	-1.25599500	0.00000100
H	-4.23343400	-1.03056600	0.88124500
H	-4.23343600	-1.03056700	-0.88124300
H	-3.38300400	-2.31820800	0.00000000
C	3.70205200	-1.18826100	0.00000100
H	4.30172200	-0.93664800	-0.88074300
H	4.30171900	-0.93665000	0.88074700
H	3.53934500	-2.26733300	-0.00000100
C	-2.35469800	-0.44310300	0.00000000

(S175)

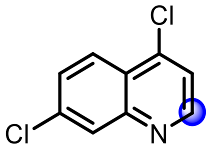

C	-2.18785800	-0.17881000	0.00000400
C	-1.52229700	1.01922300	-0.00000600
C	-0.10413300	1.03658900	0.00000400
C	0.60907800	-0.20596900	0.00000100
C	-0.12395200	-1.41892900	-0.00000200
C	-1.49655700	-1.41109700	0.00000800
H	-2.04556700	1.96589700	-0.00001100
C	2.02555500	-0.11385200	-0.00000500
H	0.40922300	-2.36051100	-0.00000300
H	-2.05575600	-2.33771900	0.00001200
C	2.64072100	1.11147600	0.00000000
C	1.82619400	2.26758900	0.00001200
H	3.71892800	1.19896600	-0.00000200
H	2.30065300	3.24579400	-0.00000500
N	0.51277600	2.25109600	-0.00000500
Cl	3.00623400	-1.56575600	-0.00000100
Cl	-3.94255300	-0.20526900	-0.00000100

(S176)

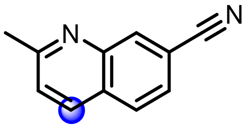

C	2.17722900	-0.14776300	0.00000000
C	1.03522700	-0.92823900	-0.00000300
C	-0.24215600	-0.32118200	-0.00000300
C	-0.33347200	1.10201300	0.00000000
C	0.85378100	1.87656500	0.00000300
C	2.08496400	1.27226600	0.00000400
H	1.09130900	-2.00927000	-0.00000600
C	-1.63563300	1.66354800	-0.00000100
H	0.77841300	2.95869300	0.00000600
C	-2.72351900	0.83396200	-0.00000500
C	-2.53598400	-0.58134600	-0.00000600
H	-1.75466700	2.74215300	0.00000000
H	-3.72995100	1.23711700	-0.00000800
N	-1.34021300	-1.13193800	-0.00000600
H	2.99333100	1.86135300	0.00000600
C	-3.73276800	-1.49514200	0.00000700
H	-4.35758900	-1.31843100	0.88121900
H	-4.35778200	-1.31822300	-0.88102400
H	-3.40662300	-2.53410600	-0.00014200
C	3.46909100	-0.76506000	0.00000000
N	4.51778400	-1.25049400	0.00000100

(S177)

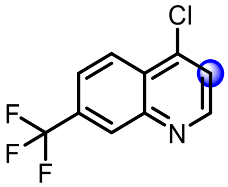

C	1.60688100	-0.10185400	-0.00000400
C	0.90273100	1.07527000	0.00000000
C	-0.51618500	1.06227500	0.00000500
C	-1.19715000	-0.19701700	0.00000700
C	-0.43609500	-1.39410900	0.00000900
C	0.93394500	-1.34950800	0.00000100
H	1.39770600	2.03695600	-0.00000500
C	-2.61626500	-0.13844800	0.00000000
H	-0.94856800	-2.34691900	0.00001300
H	1.50726100	-2.26841800	-0.00000200
C	-3.25817300	1.07277500	-0.00000300
C	-2.47281600	2.24897000	-0.00000100
H	-4.33838100	1.13415600	-0.00000900
H	-2.97007000	3.21537300	-0.00000400
N	-1.15946000	2.26168900	0.00000200
Cl	-3.56310900	-1.61187900	-0.00000300
C	3.11238300	-0.10402500	-0.00000300
F	3.60796700	-0.74846700	-1.08531700
F	3.60796500	-0.74830700	1.08540700
F	3.63802200	1.13599700	-0.00009100

(S178)

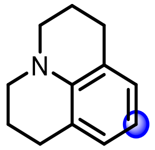

C	-1.19678800	2.21006500	0.01336900
C	-1.22124300	0.81673200	0.02562600
C	-2.55214300	0.08915800	0.05858300
C	-0.00019800	2.91983500	0.00725000
C	0.00000200	0.09956000	0.02095100
C	1.22112000	0.81687600	0.02563900
C	1.19646700	2.21024000	0.01338500
C	2.55212400	0.08949500	0.05862400
H	2.94803200	0.10587900	1.08252900
C	2.42349100	-1.36117200	-0.40533100
C	1.23054600	-2.01409900	0.28401000
C	-1.23026100	-2.01425100	0.28406700
C	-2.42329300	-1.36151400	-0.40530500
H	-3.33490200	-1.92302400	-0.18121500
H	-1.11380600	-3.04893500	-0.05119400
H	-0.00030900	4.00339700	-0.00532800
H	2.14150700	2.74557600	0.00801500
H	2.27016500	-1.40031300	-1.48863400
H	1.11426000	-3.04878600	-0.05130100
H	-2.94806700	0.10553800	1.08247800
H	-2.14187300	2.74532000	0.00801600
H	3.27988400	0.62604100	-0.55765500
H	3.33517500	-1.92257100	-0.18127200
H	1.40199000	-2.04607300	1.37528900
H	-1.40167600	-2.04618900	1.37535100
H	-2.26996700	-1.40069400	-1.48860600
H	-3.27994400	0.62559300	-0.55774400
N	0.00008500	-1.30375900	-0.03627700

(S179)

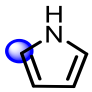

C	-0.00005000	-0.33116500	1.12485100
C	-0.00005000	0.98244800	0.71237000
C	-0.00005000	0.98244800	-0.71237000
C	-0.00005000	-0.33116500	-1.12485100
N	0.00010400	-1.12183600	0.00000000
H	0.00044000	-2.12788400	0.00000000
H	0.00008100	-0.76296800	2.11233600
H	-0.00006000	1.84563200	1.35946500
H	-0.00006000	1.84563200	-1.35946500
H	0.00008100	-0.76296800	-2.11233600

(S180)

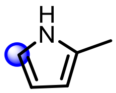

C	1.46554100	-0.70208900	0.00011700
C	1.53639100	0.67045700	0.00016800
C	0.19819600	1.16454400	-0.00007500
C	-0.65669800	0.08238700	-0.00014100
N	0.13137800	-1.04851700	-0.00029200
H	-0.21808200	-1.99265300	0.00023700
H	2.23822600	-1.45352100	0.00020600
H	2.44285700	1.25552200	0.00004700
H	-0.10766600	2.19985800	-0.00046100
C	-2.14913600	0.00781400	0.00014700
H	-2.53578000	-0.51217900	-0.88379100
H	-2.53538600	-0.51060000	0.88519100
H	-2.56957900	1.01451000	-0.00067800

(S181)

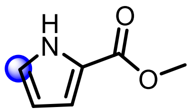

C	0.47652400	-0.01105700	-0.00002000
C	1.16142900	-1.21775200	0.00000200
C	2.54248700	-0.91729600	-0.00004200
C	2.66538900	0.46320400	0.00007700
N	1.41379800	0.99777100	-0.00000300
H	1.16630500	1.97590800	-0.00002700
H	0.70278800	-2.19327200	-0.00000500
H	3.35948200	-1.62152900	-0.00007600
H	3.54293200	1.08956100	0.00013300
C	-0.93655300	0.34121200	-0.00004900
O	-1.34866800	1.48623200	-0.00002800
O	-1.73962500	-0.74315200	0.00000100
C	-3.15260000	-0.47595800	0.00004200
H	-3.63091600	-1.45295100	0.00035800
H	-3.43537000	0.08982500	0.88895100
H	-3.43551100	0.08930100	-0.88915900

(S182)

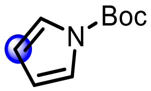

C	1.90381700	-1.19828500	0.00000900
C	3.26930500	-1.18614500	0.00000500
C	3.69539300	0.18430600	-0.00001100
C	2.57685500	0.96699000	-0.00001400
N	1.46778000	0.12603200	-0.00000200
H	1.19482800	-2.00641600	0.00002400
H	3.90415700	-2.05870200	0.00001300
H	4.71293600	0.54288700	-0.00002000
H	2.44018700	2.03433000	-0.00002500
C	0.14508400	0.60006800	0.00000200
O	-0.12251500	1.77689800	0.00002600
O	-0.71124400	-0.42495500	-0.00002600
C	-2.18241500	-0.20788400	-0.00000400
C	-2.59569900	0.53258100	1.27281700
H	-2.21057600	1.55105100	1.28571600
H	-3.68694000	0.57239100	1.32571500
H	-2.23325900	0.00337600	2.15765500
C	-2.59572600	0.53272200	-1.27273400
H	-3.68696900	0.57247100	-1.32564100
H	-2.21066100	1.55121600	-1.28549400
H	-2.23324100	0.00365400	-2.15763500
C	-2.71996000	-1.63827700	-0.00007400
H	-3.81234300	-1.62321500	-0.00003200
H	-2.38125300	-2.17857700	-0.88675400
H	-2.38118800	-2.17868700	0.88651400

(S183)

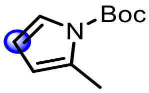

C	1.46069900	-1.59468500	-0.00001400
C	2.80526900	-1.80734900	0.00003600
C	3.44702000	-0.52617000	0.00004200
C	2.48642100	0.44841600	-0.00002100
N	1.24183600	-0.21150900	-0.00008800
H	0.62857000	-2.27436200	-0.00002900
H	3.28820400	-2.77228400	0.00005500
H	4.51023000	-0.33921800	0.00004200
C	-0.01445200	0.41402000	0.00002100
O	-0.16257100	1.61265800	0.00017100
O	-0.98560200	-0.50785600	-0.00006600
C	-2.42051700	-0.12376100	-0.00000900
C	-2.74846800	0.65842000	1.27298400
H	-2.25439000	1.62863700	1.28340700
H	-3.82891600	0.81600100	1.32870400
H	-2.44304100	0.09335700	2.15726800
C	-2.74859100	0.65885000	-1.27271000
H	-3.82909200	0.81606500	-1.32845100
H	-2.25482200	1.62922400	-1.28275900
H	-2.44291200	0.09423400	-2.15719600
C	-3.11942200	-1.48302700	-0.00023500
H	-4.20294400	-1.34275900	-0.00006300
H	-2.84570500	-2.05865600	-0.88718500
H	-2.84547300	-2.05905200	0.88638400
C	2.66232900	1.93158000	-0.00010200
H	2.20969300	2.40116600	-0.87650800
H	2.20945900	2.40129300	0.87612300
H	3.73194700	2.15074600	0.00003500

(S184)

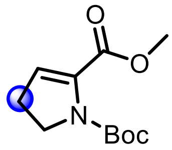

C	-1.08409600	2.67482300	0.02689200
C	-2.41878100	2.60713900	0.33540200
C	-2.77198800	1.23295400	0.37728800
C	-1.64361700	0.48896800	0.09733300
N	-0.59616600	1.39134000	-0.11990200
H	-0.41388500	3.51200700	-0.07182200
H	-3.06928600	3.44974500	0.50876400
H	-3.75240100	0.81523100	0.54074200
C	0.80834000	1.16421500	-0.23676800
O	1.52510200	1.96954800	-0.77516600
O	1.14633500	0.03888300	0.36558800
C	2.50981400	-0.54934500	0.21419500
C	2.80938300	-0.76584300	-1.26900100
H	2.92937500	0.17970500	-1.79686500
H	3.73855200	-1.33407100	-1.36371000
H	2.00440600	-1.33705800	-1.73556200
C	3.53037400	0.35646500	0.90293500
H	4.50725200	-0.13461600	0.89584000
H	3.61913000	1.31510400	0.39368000
H	3.24685600	0.53083200	1.94398300
C	2.36625400	-1.88232600	0.94554100
H	3.31592600	-2.42184500	0.91740300
H	2.09032300	-1.72154000	1.99017100
H	1.59894300	-2.49549200	0.47010400
C	-1.58877000	-0.93835700	-0.24280600
O	-0.79390200	-1.47201000	-0.98427600
O	-2.61267900	-1.60231300	0.34643600
C	-2.74764700	-2.98676400	-0.01561400
H	-1.86017700	-3.54870900	0.27895700
H	-3.62100500	-3.34310200	0.52648100
H	-2.89528700	-3.09001700	-1.09189100

7. References for Supporting Information

(1) Paul, S.; Chotana, G. A.; Holmes, D.; Reichle, R. C.; Maleczka, Robert E.; Smith, M. R. Ir-Catalyzed Functionalization of 2-Substituted Indoles at the 7-Position: Nitrogen-Directed Aromatic Borylation. *J. Am. Chem. Soc.* **2006**, *128*, 15552–15553.

(2) Su, B.; Hartwig, J. F. Iridium-Catalyzed, Silyl-Directed, Peri-Borylation of C−H Bonds in Fused Polycyclic Arenes and Heteroarenes. *Angew. Chem. Int. Ed.* **2018**, *57*, 10163–10167.

(3) Joliton, A.; Carreira, E. M. Ir-Catalyzed Preparation of SF5-Substituted Potassium Aryl Trifluoroborates via C–H Borylation and Their Application in the Suzuki–Miyaura Reaction. *Org. Lett.* **2013**, *15*, 5147–5149.

(4) Oeschger, R.; Su, B.; Yu, I.; Ehinger, C.; Romero, E.; He, S.; Hartwig, J. Diverse Functionalization of Strong Alkyl C–H Bonds by Undirected Borylation. *Science* **2020**, *368*, 736–741.

(5) Chattopadhyay, B.; Dannatt, J. E.; Andujar-De Sanctis, I. L.; Gore, K. A.; Maleczka, R. E., Jr.; Singleton, D. A.; Smith, M. R., III. Ir-Catalyzed Ortho-Borylation of Phenols Directed by Substrate–Ligand Electrostatic Interactions: A Combined Experimental/in Silico Strategy for Optimizing Weak Interactions. *J. Am. Chem. Soc.* **2017**, *139*, 7864–7871.

(6) Sasaki, I.; Taguchi, J.; Hiraki, S.; Ito, H.; Ishiyama, T. Catalyst-Controlled Regiodivergent C–H Borylation of Multifunctionalized Heteroarenes by Using Iridium Complexes. *Chem. Eur. J.* **2015**, *21*, 9236–9241.

(7) Lawrence, J. D.; Takahashi, M.; Bae, C.; Hartwig, J. F. Regiospecific Functionalization of Methyl C−H Bonds of Alkyl Groups in Reagents with Heteroatom Functionality. *J. Am. Chem. Soc.* **2004**, *126*, 15334–15335.

(8) Gayler, K. M.; Kong, K.; Reisenauer, K.; Taube, J. H.; Wood, J. L. Staurosporine Analogs Via C–H Borylation. *ACS Med. Chem. Lett.* **2020**, *11*, 2441–2445.

(9) He, Z.-T.; Li, H.; Haydl, A. M.; Whiteker, G. T.; Hartwig, J. F. Trimethylphosphate as a Methylating Agent for Cross Coupling: A Slow-Release Mechanism for the Methylation of Arylboronic Esters. *J. Am. Chem. Soc.* **2018**, *140*, 17197–17202.

(10) Lo, W. F.; Kaiser, H. M.; Spannenberg, A.; Beller, M.; Tse, M. K. A Highly Selective Ir-Catalyzed Borylation of 2-Substituted Indoles: A New Access to 2,7- and 2,4,7-Substituted Indoles. *Tetrahedron Lett.* **2007**, *48*, 371–375.

(11) Preshlock, S. M.; Plattner, D. L.; Maligres, P. E.; Krska, S. W.; Maleczka Jr., R. E.; Smith III, M. R. A Traceless Directing Group for C–H Borylation. *Angew. Chem. Int. Ed.* **2013**, *52*, 12915–12919.

(12) Meyer, F.-M.; Liras, S.; Guzman-Perez, A.; Perreault, C.; Bian, J.; James, K. Functionalization of Aromatic Amino Acids via Direct C−H Activation: Generation of Versatile Building Blocks for Accessing Novel Peptide Space. *Org. Lett.* **2010**, *12*, 3870–3873.

(13) Nippa, D. F.; Atz, K.; Hohler, R.; Müller, A. T.; Marx, A.; Bartelmus, C.; Wuitschik, G.; Marzuoli, I.; Jost, V.; Wolfard, J.; Binder, M.; Stepan, A. F.; Konrad, D. B.; Grether, U.; Martin, R. E.; Schneider, G. Enabling Late-Stage Drug Diversification by High-Throughput Experimentation with Geometric Deep Learning. *Nat. Chem.* **2023**, *16*, 239–248.

(14) Caldeweyher, E.; Elkin, M.; Gheibi, G.; Johansson, M.; Sköld, C.; Norrby, P.-O.; Hartwig, J. F. Hybrid Machine Learning Approach to Predict the Site Selectivity of Iridium-Catalyzed Arene Borylation. *J. Am. Chem. Soc.* **2023**, *145*, 17367–17376.

(15) Shi, F.; Smith, M. R.; Maleczka, R. E. Aromatic Borylation/Amidation/Oxidation: A Rapid Route to 5-Substituted 3-Amidophenols. *Org. Lett.* **2006**, *8*, 1411–1414.

(16) Tajuddin, H.; Harrisson, P.; Bitterlich, B.; Collings, J. C.; Sim, N.; Batsanov, A. S.; Cheung, M. S.; Kawamorita, S.; Maxwell, A. C.; Shukla, L.; Morris, J.; Lin, Z.; Marder, T. B.; Steel, P. G. Iridium-Catalyzed C–H Borylation of Quinolines and Unsymmetrical 1,2-Disubstituted Benzenes: Insights into Steric and Electronic Effects on Selectivity. *Chem. Sci.* **2012**, *3*, 3505–3515.

(17) Shockley, S. E.; Holder, J. C.; Stoltz, B. M. A Catalytic, Enantioselective Formal Synthesis of (+)-Dichroanone and (+)-Taiwaniaquinone H. *Org. Lett.* **2014**, *16*, 6362–6365.
(18) Zeng, J.; Naito, M.; Torigoe, T.; Yamanaka, M.; Kuninobu, Y. Iridium-Catalyzed Ortho-C–H Borylation of Thioanisole Derivatives Using Bipyridine-Type Ligand. *Org. Lett.* **2020**, *22*, 3485–3489.
(19) Myeong, I.-S.; Avci, N. H.; Movassaghi, M. Total Synthesis of (–)-Kopsifoline A and (+)-Kopsifoline E. *Org. Lett.* **2021**, *23*, 9118–9122.
(20) Harmer, R.; Fan, H.; Lloyd, K.; Doble, S.; Avenoso, J.; Yan, H.; Rego, L. G. C.; Gundlach, L.; Galoppini, E. Synthesis and Properties of Perylene-Bridge-Anchor Chromophoric Compounds. *J. Phys. Chem. A* **2020**, *124*, 6330–6343.
(21) Kawamorita, S.; Ohmiya, H.; Sawamura, M. Ester-Directed Regioselective Borylation of Heteroarenes Catalyzed by a Silica-Supported Iridium Complex. *J. Org. Chem.* **2010**, *75*, 3855–3858.
(22) Murphy, J. M.; Lawrence, J. D.; Kawamura, K.; Incarvito, C.; Hartwig, J. F. Ruthenium-Catalyzed Regiospecific Borylation of Methyl C−H Bonds. *J. Am. Chem. Soc.* **2006**, *128*, 13684–13685.
(23) Ohmura, T.; Torigoe, T.; Suginome, M. Catalytic Functionalization of Methyl Group on Silicon: Iridium-Catalyzed C(Sp^3)–H Borylation of Methylchlorosilanes. *J. Am. Chem. Soc.* **2012**, *134*, 17416–17419.
(24) Liskey, C. W.; Hartwig, J. F. Iridium-Catalyzed Borylation of Secondary C–H Bonds in Cyclic Ethers. *J. Am. Chem. Soc.* **2012**, *134*, 12422–12425.
(25) Li, Q.; Liskey, C. W.; Hartwig, J. F. Regioselective Borylation of the C–H Bonds in Alkylamines and Alkyl Ethers. Observation and Origin of High Reactivity of Primary C–H Bonds Beta to Nitrogen and Oxygen. *J. Am. Chem. Soc.* **2014**, *136*, 8755–8765.
(26) Miyamura, S.; Araki, M.; Suzuki, T.; Yamaguchi, J.; Itami, K. Stereodivergent Synthesis of Arylcyclopropylamines by Sequential C–H Borylation and Suzuki–Miyaura Coupling. *Angew. Chem. Int. Ed. Engl.* **2015**, *54*, 846–851.
(27) Boebel, T. A.; Hartwig, John. F. Silyl-Directed, Iridium-Catalyzed Ortho-Borylation of Arenes. A One-Pot Ortho-Borylation of Phenols, Arylamines, and Alkylarenes. *J. Am. Chem. Soc.* **2008**, *130*, 7534–7535.
(28) Cho, S. H.; Hartwig, J. F. Iridium-Catalyzed Borylation of Secondary Benzylic C–H Bonds Directed by a Hydrosilane. *J. Am. Chem. Soc.* **2013**, *135*, 8157–8160.
(29) Kawamorita, S.; Ohmiya, H.; Hara, K.; Fukuoka, A.; Sawamura, M. Directed Ortho Borylation of Functionalized Arenes Catalyzed by a Silica-Supported Compact Phosphine−Iridium System. *J. Am. Chem. Soc.* **2009**, *131*, 5058–5059.
(30) Preshlock, S. M.; Ghaffari, B.; Maligres, P. E.; Krska, S. W.; Maleczka, R. E. Jr.; Smith, M. R. I. High-Throughput Optimization of Ir-Catalyzed C–H Borylation: A Tutorial for Practical Applications. *J. Am. Chem. Soc.* **2013**, *135*, 7572–7582.
(31) Cho, J.-Y.; Iverson, C. N.; Smith, M. R. Steric and Chelate Directing Effects in Aromatic Borylation. *J. Am. Chem. Soc.* **2000**, *122*, 12868–12869.
(32) Holmes, D.; Chotana, G. A.; Maleczka, R. E.; Smith, M. R. One-Pot Borylation/Amination Reactions: Syntheses of Arylamine Boronate Esters from Halogenated Arenes. *Org. Lett.* **2006**, *8*, 1407–1410.
(33) Bisht, R.; Chattopadhyay, B. Formal Ir-Catalyzed Ligand-Enabled Ortho and Meta Borylation of Aromatic Aldehydes via in Situ-Generated Imines. *J. Am. Chem. Soc.* **2016**, *138*, 84–87.
(34) Roosen, P. C.; Kallepalli, V. A.; Chattopadhyay, B.; Singleton, D. A.; Maleczka, R. E., Jr.; Smith, M. R., III. Outer-Sphere Direction in Iridium C–H Borylation. *J. Am. Chem. Soc.* **2012**, *134*, 11350–11353.
(35) Murphy, J. M.; Liao, X.; Hartwig, J. F. Meta Halogenation of 1,3-Disubstituted Arenes via Iridium-Catalyzed Arene Borylation. *J. Am. Chem. Soc.* **2007**, *129*, 15434–15435.
(36) Crawford, A. G.; Liu, Z.; Mkhalid, I. A. I.; Thibault, M.-H.; Schwarz, N.; Alcaraz, G.; Steffen, A.; Collings, J. C.; Batsanov, A. S.; Howard, J. A. K.; Marder, T. B. Synthesis of 2- and 2,7-Functionalized Pyrene Derivatives: An Application of Selective C–H Borylation. *Chem. Eur. J.* **2012**, *18*, 5022–5035.

(37) Takagi, J.; Sato, K.; Hartwig, J. F.; Ishiyama, T.; Miyaura, N. Iridium-Catalyzed C–H Coupling Reaction of Heteroaromatic Compounds with Bis(pinacolato)diboron: Regioselective Synthesis of Heteroarylboronates. *Tetrahedron Lett.* **2002**, *43*, 5649–5651.
(38) Ishiyama, T.; Takagi, J.; Yonekawa, Y.; Hartwig, J. F.; Miyaura, N. Iridium-Catalyzed Direct Borylation of Five-Membered Heteroarenes by Bis(pinacolato)diboron: Regioselective, Stoichiometric, and Room Temperature Reactions. *Adv. Synth. Catal.* **2003**, *345*, 1103–1106.
(39) Chotana, G. A.; Kallepalli, V. A.; Maleczka, R. E.; Smith, M. R. Iridium-Catalyzed Borylation of Thiophenes: Versatile, Synthetic Elaboration Founded on Selective C–H Functionalization. *Tetrahedron* **2008**, *64*, 6103–6114.
(40) Kallepalli, V. A.; Shi, F.; Paul, S.; Onyeozili, E. N.; Maleczka, R. E. Jr.; Smith, M. R. I. Boc Groups as Protectors and Directors for Ir-Catalyzed C−H Borylation of Heterocycles. *J. Org. Chem.* **2009**, *74*, 9199–9201.
(41) Larsen, M. A.; Hartwig, J. F. Iridium-Catalyzed C–H Borylation of Heteroarenes: Scope, Regioselectivity, Application to Late-Stage Functionalization, and Mechanism. *J. Am. Chem. Soc.* **2014**, *136*, 4287–4299.
(42) Shinamura, S.; Sugimoto, R.; Yanai, N.; Takemura, N.; Kashiki, T.; Osaka, I.; Miyazaki, E.; Takimiya, K. Orthogonally Functionalized Naphthodithiophenes: Selective Protection and Borylation. *Org. Lett.* **2012**, *14*, 4718–4721.
(43) Loach, R. P.; Fenton, O. S.; Amaike, K.; Siegel, D. S.; Ozkal, E.; Movassaghi, M. C7-Derivatization of C3-Alkylindoles Including Tryptophans and Tryptamines. *J. Org. Chem.* **2014**, *79*, 11254–11263.
(44) Fischer, D. F.; Sarpong, R. Total Synthesis of (+)-Complanadine A Using an Iridium-Catalyzed Pyridine C−H Functionalization. *J. Am. Chem. Soc.* **2010**, *132*, 5926–5927.
(45) Hoque, M. E.; Bisht, R.; Haldar, C.; Chattopadhyay, B. Noncovalent Interactions in Ir-Catalyzed C–H Activation: L-Shaped Ligand for Para-Selective Borylation of Aromatic Esters. *J. Am. Chem. Soc.* **2017**, *139*, 7745–7748.
(46) Sadler, S. A.; Tajuddin, H.; Mkhalid, I. A. I.; Batsanov, A. S.; Albesa-Jove, D.; Cheung, M. S.; Maxwell, A. C.; Shukla, L.; Roberts, B.; Blakemore, D. C.; Lin, Z.; Marder, T. B.; Steel, P. G. Iridium-Catalyzed C–H Borylation of Pyridines. *Org. Biomol. Chem.* **2014**, *12*, 7318–7327.
(47) Oeschger, R. J.; Larsen, M. A.; Bismuto, A.; Hartwig, J. F. Origin of the Difference in Reactivity between Ir Catalysts for the Borylation of C–H Bonds. *J. Am. Chem. Soc.* **2019**, *141*, 16479–16485.
(48) Gensch, T.; dos Passos Gomes, G.; Friederich, P.; Peters, E.; Gaudin, T.; Pollice, R.; Jorner, K.; Nigam, A.; Lindner-D'Addario, M.; Sigman, M. S.; Aspuru-Guzik, A. A Comprehensive Discovery Platform for Organophosphorus Ligands for Catalysis. *J. Am. Chem. Soc.* **2022**, *144*, 1205–1217.
(49) Zhong, R.-L.; Sakaki, S. Methane Borylation Catalyzed by Ru, Rh, and Ir Complexes in Comparison with Cyclohexane Borylation: Theoretical Understanding and Prediction. *J. Am. Chem. Soc.* **2020**, *142*, 16732–16747.
(50) Gaussian 09, Revision D.01: Frisch, M. J.; Trucks, G. W.; Schlegel, H. B.; Scuseria, G. E.; Robb, M. A.; Cheeseman, J. R.; Scalmani, G.; Barone, V.; Mennucci, B.; Petersson, G. A.; Nakatsuji, H.; Caricato, M.; Li, X.; Hratchian, H. P.; Izmaylov, A. F.; Bloino, J.; Zheng, G.; Sonnenberg, J. L.; Hada, M.; Ehara, M.; Toyota, K.; Fukuda, R.; Hasegawa, J.; Isheda, M.; Nakajima, T.; Honda, Y.; Kitao, O.; Nakai, H.; Vreven, T.; Montgomery, J. A., Jr.; Peralta, J. E.; Ogliaro, F.; Bearpark, M.; Heyd, J. J.; Brothers, E.; Kudin, K. N.; Staroverov, V. N.; Kobayashi, R.; Normand, J.; Raghavachari, K.; Rendell, A.; Burant, J. C.; Iyengar, S. S.; Tomasi, J.; Cossi, M.; Rega, N.; Millam, N. J.; Klene, M.; Knox, J. E.; Cross, J. B.; Bakken, V.; Adamo, C.; Jaramillo, J.; Gomperts, R.; Stratmann, R. E.; Yazyev, O.; Austin, A. J.; Cammi, R.; Pomelli, C.; Ochterski, J. W.; Martin, R. L.; Morokuma, K.; Zakrzewski, V. G.; Voth, G. A.; Salvador, P.; Dannenberg, J. J.; Dapprich, S.; Daniels, A. D.; Farkas, Ő.; Foresman, J. B.; Ortiz, J. V.; Cioslowski, J.; Fox, D. J. Gaussian, Inc.: Wallingford, CT, 2013.

(51) Dunning, Thom. H.; Hay, P. J. Gaussian Basis Sets for Molecular Calculations. In *Methods of Electronic Structure Theory*; Schaefer, H. F., Ed.; Modern Theoretical Chemistry; Springer US: Boston, MA, 1977; pp 1–27.

(52) Becke, A. D. Density-functional Thermochemistry. III. The Role of Exact Exchange. *J. Chem. Phys* **1993**, *98*, 5648–5652.

(53) Lee, C.; Yang, W.; Parr, R. G. Development of the Colle-Salvetti Correlation-Energy Formula into a Functional of the Electron Density. *Phys. Rev. B Condens. Matter* **1988**, *37*, 785–789.

(54) Vosko, S. H.; Wilk, L.; Nusair, M. Accurate Spin-Dependent Electron Liquid Correlation Energies for Local Spin Density Calculations: A Critical Analysis. *Can. J. Phys.* **1980**, *58*, 1200–1211.

(55) Stephens, P. J.; Devlin, F. J.; Chabalowski, C. F.; Frisch, M. J. Ab Initio Calculation of Vibrational Absorption and Circular Dichroism Spectra Using Density Functional Force Fields. *J. Phys. Chem.* **1994**, *98*, 11623–11627.

(56) Hirshfeld, F. L. Bonded-Atom Fragments for Describing Molecular Charge Densities. *Theoret. Chim. Acta* **1977**, *44*, 129–138.

(57) Marenich, A. V.; Jerome, S. V.; Cramer, C. J.; Truhlar, D. G. Charge Model 5: An Extension of Hirshfeld Population Analysis for the Accurate Description of Molecular Interactions in Gaseous and Condensed Phases. *J. Chem. Theory Comput.* **2012**, *8*, 527–541.

(58) Breneman, C. M.; Wiberg, K. B. Determining Atom-Centered Monopoles from Molecular Electrostatic Potentials. The Need for High Sampling Density in Formamide Conformational Analysis. *J. Comput. Chem.* **1990**, *11*, 361–373.

(59) Reed, A. E.; Weinstock, R. B.; Weinhold, F. Natural Population Analysis. *J. Chem. Phys.* **1985**, *83*, 735–746.

(60) Mulliken, R. S. Electronic Population Analysis on LCAO–MO Molecular Wave Functions. I. *J. Chem. Phys.* **1955**, *23*, 1833–1840.

(61) ChemAxon. JChem for Excel [For Windows], version 21.15.704. **2020**. https://chemaxon/. (accessed 2024-08-01).

(62) Moriwaki, H.; Tian, Y.-S.; Kawashita, N.; Takagi, T. Mordred: A Molecular Descriptor Calculator. *J. Cheminform.* **2018**, *10*, 4.

(63) *How to visualize decision tree*. http://explained.ai/decision-tree-viz/index.html (accessed 2023-04-22).
